# Licochalcone A-Inspired Chalcones: Synthesis and Their Antiproliferative Potential in Prostate Cancer Cells

**DOI:** 10.3390/molecules29246023

**Published:** 2024-12-20

**Authors:** Roxana Gonzalez Dorado, Esveidy Isabel Oceguera Nava, Guanglin Chen, Qiang Zhang, Guangdi Wang, Qiao-Hong Chen

**Affiliations:** 1 Department of Chemistry & Biochemistry, California State University, Fresno, CA 93740, USA; 2 Department of Chemistry and RCMI Cancer Research Center, Xavier University of Louisiana, New Orleans, LA 70125, USA

**Keywords:** licochalcone A, prostate cancer, androgen receptor, cell proliferation, chalcones

## Abstract

Prostate cancer remains a significant global health concern, prompting ongoing exploration of novel therapeutic agents. Licochalcone A, a natural product in the chalcone family isolated from licorice root, is characterized by its enone structure and demonstrates antiproliferative activity in the micromolar range across various cell lines, including prostate cancer. Building on our prior success in enhancing curcumin’s antiproliferative potency by replacing the substituted phenol with a 1-alkyl-1H-imizadol-2-yl moiety, we applied a similar approach to design a new class of licochalcone A-inspired chalcones. The synthesis of these target chalcones involved key [3,3]-sigmatropic rearrangement of aryl prenyl ethers and Claisen–Schmidt condensations, yielding three derivative series. These compounds were evaluated for antiproliferative activity in both androgen receptor (AR)-positive and AR-null prostate cancer cell models using WST-1 cell proliferation assay. Systematic evaluation of licochalcone A across four prostate cancer cell lines indicated a modest advantage over enzalutamide, an FDA-approved AR antagonist, in suppressing 22Rv1 cell proliferation. Interestingly, three ester derivatives by replacing the phenol next to the carbonyl with an alkoxide demonstrated similar antiproliferative potency to licochalcone A in both AR-positive and AR-negative prostate cancer cell lines. This suggests that the phenol moiety on licochalcone A may be a promising site for chemical manipulations to enhance anti-prostate cancer activity. Among the synthesized chalcones, nine derivatives showed improved selectivity for AR-positive LNCaP and 22RV1 cells relative to AR-negative PC-3 and DU145 cells, surpassing licochalcone A in selectivity. Additionally, the antiproliferative potency was highly dependent on the R group attached to the imidazole. Most of the derivatives showed antiproliferative potency against androgen receptor-positive LNCaP and 22Rv1 cells, comparable to that of enzalutamide and licochalcone A. These findings suggest that optimization of licochalcone A-inspired chalcones as potential anti-prostate cancer agents warrants further investigation.

## 1. Introduction

Prostate cancer remains a significant health issue, representing an estimated 29% of all new cancer diagnoses in American men in 2024. In the United States alone, around 299,000 new cases of prostate cancer are expected, leading to over 35,000 deaths this year [1]. Of particular concern is the 3% annual increase in prostate cancer incidence since 2014 [1]. Castration-resistant prostate cancer (CRPC) represents an advanced, lethal form of prostate cancer that continues to progress despite extremely low levels of serum androgens [2]. The androgen receptor (AR) signaling pathway, activated by androgens, is the primary driver of prostate cancer development and progression [3]. AR remains a promising therapeutic target for CRPC, as the disease’s progression is largely driven by the reactivation of AR signaling [2]. As a ligand-dependent transcription factor, AR regulates genes associated with prostate cancer growth and metastasis [4]. This insight led to the design and FDA approval of three second-generation non-steroidal AR antagonists: enzalutamide [5], apalutamide [6], and darolutamide [7]. Additionally, proxalutamide, a novel AR antagonist, has recently advanced to late-stage clinical trials [8]. These four AR antagonists are notable for their impact on patient survival, enhanced binding affinity to the AR ligand-binding domain (LBD), and increased specificity for AR over other steroid hormone receptors. However, resistance to these drugs remains a significant challenge, often due to AR gene amplification, mutations in the LBD, and the emergence of AR splice variants lacking the LBD. Given that most resistance mechanisms focus on the LBD, developing drugs that target other functional domains of AR offers a promising approach for treating lethal CRPC. For instance, targeting the intrinsically disordered yet constitutively active *N*-terminal (NTD) of AR could circumvent some of these resistance mechanisms [9]. The AR NTD is essential for the transcriptional activity of both full-length AR and AR variants lacking the LBD, making it an attractive yet challenging drug target. Therefore, pursuing novel AR antagonists that specifically target the NTD represents a promising alternative strategy for overcoming resistance to current FDA-approved AR antagonists [10]. Due to the intrinsically disordered nature of the AR NTD, rational structure-based drug design is challenging for developing AR NTD antagonists. However, natural products present a promising source of potential antagonists, as demonstrated by their historical success in drug discovery [11].

**Figure 1:**
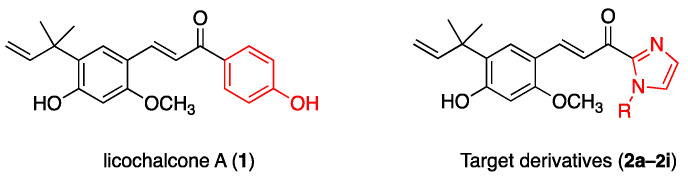
Chemical structures of licochalcone A (**1**) and target derivatives (**2a**–**2i**). **2a**, R = methyl; **2b**, R = ethyl; **2c**, R = propyl; **2d**, R = butyl; **2e**, R = *sec*butyl; **2f**, R = isobutyl; **2g**, R = isopentyl; **2h**, R = pentan-2-yl; **2i**, R = pentan-3-yl.

Licochalcone A (**1**, Figure 1) is a naturally occurring compound originally isolated from licorice root (*Glycyrrhiza species*), one of the oldest and most widely used traditional medicinal plants globally. Structurally, licochalcone A (**1**) is part of a large group of natural compounds known as chalcones (1, 3-diaryl-2-propen-1-one), characterized by a central three-carbon *α*,*β*-unsaturated ketone (enone) linker and two terminal aromatic rings. Chalcones are recognized as a privileged scaffold in medicinal chemistry due to their diverse biological activities [12].

PC-SPES is a herbal mixture formerly marketed as a complementary treatment for prostate cancer that includes extracts from *Glycyrrhiza uralensis*, the source of licochalcone A. Screening of the bioactivities of PC-SPES component herbs revealed that extracts from *G. uralensis* could suppress prostate cancer cell proliferation and reduce the expression of the androgen receptor and prostate-specific antigen [13]. Further purification of *G. uralensis* extracts led to the isolation and characterization of licochalcone A [14].

Notably, licochalcone A (**1**), as an oxygenated chalcone derived from PC-SPES composition herb licorice root, demonstrates a broad spectrum of biological activities [14,15,16]. Additionally, *Glycyrrhiza* root has been shown to decrease serum testosterone in men [14,17,18]. Licochalcone A exhibits antiproliferative effects in the micromolar range across several cancer cell lines, including prostate cancer. Specifically, it demonstrates significant antiproliferative activity in both androgen receptor-positive LNCaP [19] and androgen receptor-negative PC-3 prostate cancer cell line [20]. When PC-3 cells were treated with 25 μM licochalcone A for two to three days, it resulted in 55–83% inhibition of PC-3 cell proliferation [20]. However, only a few studies have reported on the in vitro antiproliferative activities of licochalcone A in prostate cancer cell models, with no systematic exploration of structure–activity relationships or in vivo antitumor efficacy. This paper aims to increase the selectivity of licochalcone A (**1**) in suppressing androgen receptor-positive prostate cancer cell proliferation through targeted chemical modifications. Additionally, it will investigate its structure–activity relationships and antiproliferative potency across four prostate cancer cell lines.

## 2. Results and Discussion

### 2.1. Design and Retrosynthetic Analysis of Licochalcone A-Inspired Chalcones

#### 2.1.1. Design of Licochalcone A-Inspired Chalcones

Building on our previous success in enhancing the antiproliferative potency of curcumin by substituting the phenol moiety with a 1-alkyl-1H-imizadol-2-yl group [21], we applied this strategy to design a new series of licochalcone-inspired chalcones. 1-Alkyl-1H-imizadol-2-yl moiety has been established as a promising bioisotere for the phenol moiety in curcumin in prostate cancer cell models [21]. Consequently, our initial goal was to synthesize a series of chalcones with the general chemical structure (**2a**–**2i**) depicted in Figure 1.

#### 2.1.2. Retrosynthetic Analysis of Licochalcone A-Inspired Chalcones

**Figure 2:**
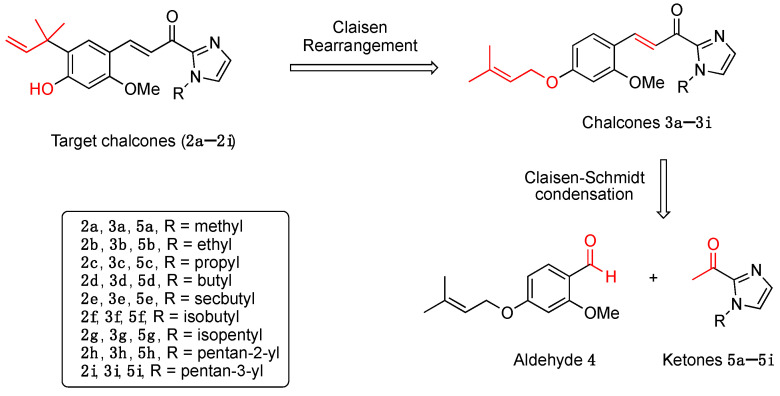
Retrosynthetic analysis of target chalcones **2a**–**2i.**

As outlined in Figure 2, the [3,3]-sigmatropic rearrangement of an aryl prenyl ether in chalcones **3a**–**3i** and the Claisen–Schmidt condensation of aldehyde **4** and ketone **5a**–**5i** were identified as the key reactions in the synthetic strategy for the target chalcones. The [3,3]-sigmatropic rearrangement is crucial for introducing the same substituents on the aromatic ring adjacent to the double bound in licochalcone A. Meanwhile, the Claisen–Schmidt condensation is essential for constructing the *α*,*β*-unsaturated ketone moiety that constitutes the backbone of the chalcone structure.

### 2.2. Synthesis and Antiproliferation Evaluation of Chalcones ***3a**–**3i***

#### 2.2.1. Synthesis of Chalcones

**Scheme 1:**
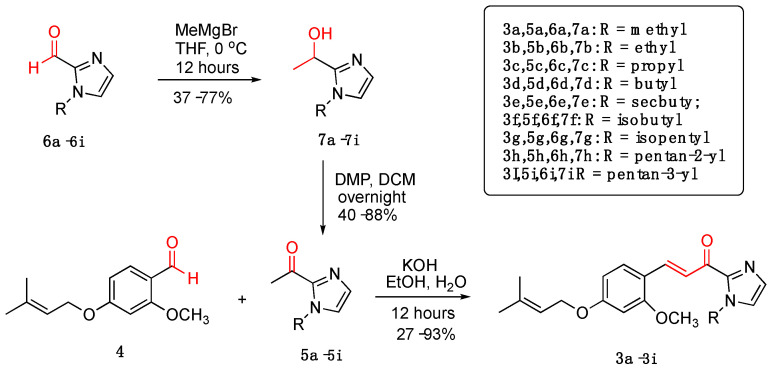
Synthesis of chalcones **3a**–**3i**

Chalcones **3a**–**3i**, with an isoprenyloxy group, were synthesized following the sequence outlined in Scheme 1. In this process, 1-alkyl-1*H*-imidazole-2-carboxaldehydes (**6a**–**6i**) were first converted into 1-(1-alkyl-1*H*-imidazol-2-yl)ethanones via a Grignard reaction, followed by oxidation using Dess–Martin periodinane. Subsequently, the aldol condensation of these 1-(1-alkyl-1*H*-imidazol-2-yl)ethanones (**5a**–**5i**) with 2-methoxy-4-[3-methyl-2-buten-1-yl]oxy]benzaldehyde (**4**) produced the desired chalcones **3a**–**3i**.

#### 2.2.2. Antiproliferative Evaluation of Licochalcone A and Chalcones **3a**–**3i**

To begin investigating the structure–activity relationship of licochalcone A-inspired chalcones, the antiproliferative potency of these chalcones (**3a**–**3i**) and parent licochalcone A (**1**) was evaluated in both androgen receptor-positive (LNCaP and 22RV1) and androgen receptor-negative (PC-3 and DU145) prostate cancer cell lines. In addition to licochalcone A, enzalutamide, a current FDA-approved androgen receptor antagonist, was included as a positive control. The WST-1 cell proliferation assay was chosen for this study due to the water solubility and stability of its tetrazolium dye [22,23]. The IC_50_ values were calculated from concentration–response (% inhibition) curves generated using five different concentrations (100 µM, 50 µM, 25 µM, 12.5 µM, and 6.25 µM) of each compound, tested across four prostate cancer cell lines. The curves were fitted using a nonlinear regression model, and data from three independent experiments were used for each cell line. Reported IC_50_ values are presented as mean ± standard deviation.

The preliminary data are summarized in Table 1. Licochalcone A demonstrates comparable potency across all four prostate cancer cell lines, with IC_50_ values ranging from 15.73 to 23.35 μM. Notably, licochalcone A exhibits slightly greater potency against the 22Rv1 cell line, which lacks a ligand-binding domain and androgen responsiveness.

Among the chalcones in this series, six derivatives (**3d**, **3e**, **3f**, **3g**, **3h**, and **3i**) displayed similar antiproliferative activity to licochalcone A in androgen receptor-positive LNCaP and 22RV1 cells. However, these compounds exhibited lower antiproliferative activity than licochalcone A in androgen receptor-negative PC-3 and DU145 cells, resulting in greater selectivity for androgen receptor-positive cells over androgen receptor-negative cells.

### 2.3. Synthesis of Esters ***8**–**10*** via Clasen Rearrangement of Chalcones ***3a**–**3i***

**Scheme 2:**
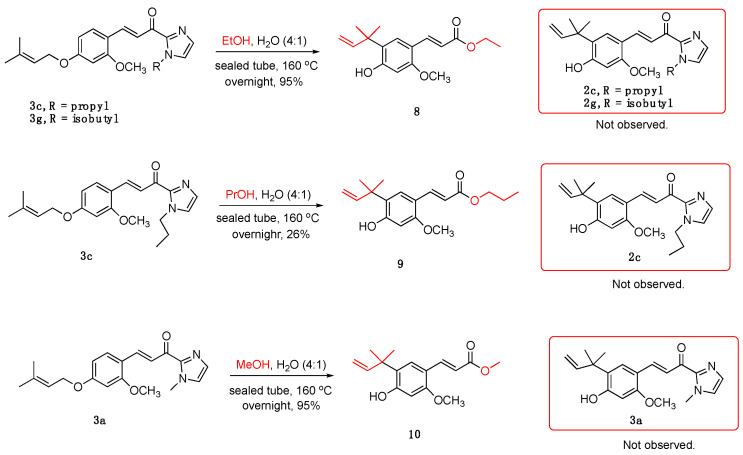
Claisen rearrangement of chalcones **3a**, **3c**, and **3g**.

Heating chalcones **3c** and **3g** at 160 °C in a sealed tube resulted in the expected [3,3]-sigmatropic rearrangement of the aryl prenyl ether portion. However, the desired imidazole moiety was not observed in product **8** (Scheme 2). The NMR data indicated the absence of the 1-alkylimidazole moiety and the presence of an ethoxy group. Additionally, the ^13^C NMR data confirmed the presence of an ester carbonyl group instead of a ketone carbonyl. The HMBC spectrum showed the correlation signal between the CH_2_ of the ethyl group and the ester carbonyl carbon. The chemical structure was further confirmed by the 2D-NMR spectra and HRMS.

It is believed that the reaction proceeds via nucleophilic attack of ethanol on the ketone carbonyl carbon, followed by reformation of the pi bond and elimination of the imidazole moiety as a leaving group under the high temperature. Similarly, heating **3c** and **3a** under PrOH:H_2_O (4:1) and MeOH:H_2_O (4:1, *v/v*), respectively, afforded esters **9** and **10** (Scheme 2).

**Figure 3:**
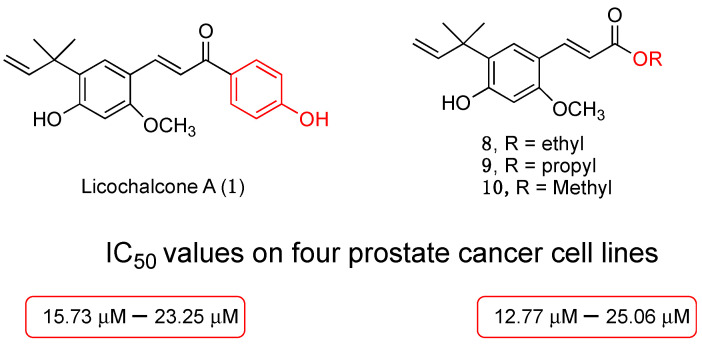
Comparison of IC_50_ values of ester derivatives **8**–**10** and licochalcone A (**1**) in prostate cancer cell lines.

Interestingly, the three esters **8**–**10** demonstrated similar antiproliferative potency to licochalcone A (**1**) in both androgen receptor-positive and androgen receptor-negative prostate cancer cell lines (Table 2 and Figure 3). This suggests that the phenol moiety next to the carbonyl group on licochalcone A (**1**) may serve as an effective site for chemical manipulations to develop more potent anti-prostate cancer agents. 

### 2.4. Synthesis and Antiproliferative Evaluation of Chalcones ***13a**–**13i***

**Scheme 3:**
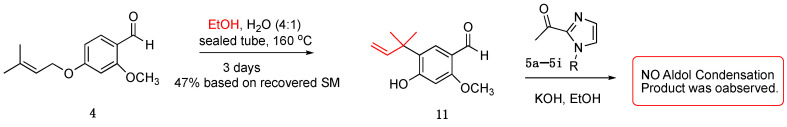
Attempt to synthesize chalcones **2a**–**2i**.

**Scheme 4:**
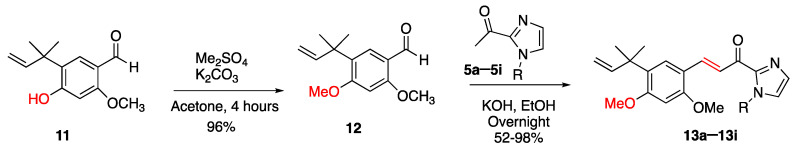
Synthesis of chalcones **13a**–**13i**. **5a** and **13a**, R = methyl; **5b** and **13b**, R = ethyl; **5c** and **13c**, R = propyl; **5d** and **13d**, R = butyl; **5e** and **13e**, R = *sec*butyl; **5f** and **13f**, R = isobutyl; **5g** and **13g**, R = isopentyl; **5h** and **13h**, R = pentan-2-yl; **5i** and **13i**, R = pentan-3-yl.

Since the imidazole moiety in chalcones **3a**–**3i** cannot withstand the high temperatures required for Claisen condensation, we reconsidered the synthetic approach. Instead, 2-methoxy-4-[(3-methyl-2-buten-1-yl)oxy]benzaldehyde (**4**), an aryl prenyl ether, underwent a [3,3]-sigmatropic rearrangement to yield the desired aldehyde **11** at 160 °C in a sealed tube, following a previously reported procedure [24]. This reaction yielded the desired aldehyde **11** with a 47% yield based on recovered starting material. However, attempts to perform an aldol condensation of aldehyde **11** with 1-(1-alkyl-1H-imidazol-2-yl)ethenones (**5a**–**5i**) were unsuccessful (Scheme 3). We attribute this failure to the presence of the phenolic hydroxyl group. To address this, 5-(1,1-dimethyl-2-propen-1-yl)-4-hydroxy-2-methoxybenzaldehyde (**11**) was methylated using dimethylsulfate in the presence of potassium carbonate, yielding **12** [25]. A series of chalcones (**13a**–**13i**) were then successfully synthesized via aldol condensation of aldehyde (**12**) and 1-(1-alkyl-1H-imidazol-2-yl)ethenones (**5a**–**5i**), mediated by potassium hydroxide (Scheme 4).

The antiproliferative potency of this series is highly dependent on the R group on the imidazole ring (Table 3). For example, derivatives **13a** and **13g**, which contain a methyl and an isopentyl group, respectively, exhibit IC_50_ values above 100 μM across all four tested prostate cancer cell lines. In contrast, the other seven derivatives in this series display antiproliferative potency against androgen receptor-positive LNCaP and 22Rv1 cells, comparable to that of enzalutamide and licochalcone A. Three derivatives (**13b**, **13f**, and **13i**) demonstrate significant selectivity in suppressing AR-positive LNCaP and 22Rv1 cell proliferation over AR-negative PC-3 and 22Rv1 prostate cancer proliferation (Table 3).

## 3. Materials and Methods

### 3.1. General Procedures

IR spectra were measured on a Nicolet Nexus 470 FTIR spectrophotometer (Thermo Nicolet, Waltham, MA, USA). HRMS data were obtained using an Orbitrap mass spectrometer (Thermo Scientific, Waltham, MA, USA) with electrospray ionization (ESI). NMR spectra were recorded on a Bruker Fourier 300 spectrometer (Bruker, Billerica, MA, USA) and a 600 MHz JEOL NMR spectrometer (JOEL, Tokyo, Japan) in CDCl_3_. Chemical shifts are recorded in ppm, referenced to the respective solvent peaks, with coupling constants given in Hz. The multiplicity of each signal in the ^1^H NMR spectra is described as singlet (s), doublet (d), doublet of doublets (dd), quartet (q), or multiplet (m). The solvent peaks were calibrated as 7.26 ppm for the ^1^H NMR spectrum and 77.16 ppm for the ^13^C NMR spectrum. THF and dichloromethane were purified using the PureSolv MD 7 Solvent Purification System from (Innovative Technologies, Herndon, VA, USA). All other reagents and solvents were purchased from commercial sources and used without further purification. Silica gel column chromatography was performed with silica gel (32–63 μm). Preparative thin-layer chromatography (PTLC) separations were conducted on silica gel 60 GF254 plates (EMD Millipore Corporation, Berlington, MA, USA). Licochalcone A (98%) was purchased from AmBeed (Arlington Heights, IL, USA). 1-Alkyl-1*H*-imidazole-2-carboxaldehydes (**6a**–**6i**) were synthesized from 1*H*-imidazole-2-carboxaldehyde following the previously described procedure [21]. 1-Methyl-1*H*-imidazole-2-carboxaldehyde was purchased from Fisher Scientific (Portland, OR, USA). All NMR spectra and high-resolution mass spectra were included in Supplementary Materials.

### 3.2. Synthesis of 1-(1-Alkyl-1H-imidazole-2-yl)ethanol (***7a**–**7i***) 

Methylmagnesium bromide (1M, 5.25 mL) was added dropwise to a solution of 1-alkyl-1*H*-imidazole-2-carboxaldehyde (2.53 mmol) in THF (0.21 mL) at 0 °C under argon. The subsequent reaction mixture was allowed to stir for 12 h at room temperature under argon. The reaction completion was monitored by TLC prior to being quenched with sodium bicarbonate (15 mL). The mixture was extracted with ethyl acetate (20 mL × 3), and the combined organic layers were dried over anhydrous sodium sulfate and concentrated on the rotary evaporator. The crude product was subjected to PTLC purification eluting with 5% methanol in dichloromethane (*v/v*) to yield the respective 1-(1-alkyl-1*H*-imidazole-2-yl)ethanol (**7a**–**7i**). Their physical and spectral data are summarized below.

#### 3.2.1. 1-(1-Methyl-1H-imidazole-2-yl)ethanol (**7a**)

Yield, 68%; light yellow oil. ^1^H NMR (300 MHz, CDCl_3_) *δ* 6.80 (d, *J* = 1.2 Hz, 1H, imidazole H), 6.76 (d, *J* = 1.2 Hz, 1H, imidazole H), 4.87 (q, *J* = 6.7 Hz, 1H, *CH*CH_3_), 3.97 (s, 1H, OH), 3.69 (s, 3H, *NCH_3_*), 1.54 (d, *J* = 6.7 Hz, 3H, CH*CH_3_*). ^13^C NMR (75 MHz, CDCl_3_) *δ* 150.20, 126.17, 121.59, 62.53, 33.05, 21.89.

#### 3.2.2. 1-(1-Ethyl-1H-imidazole-2-yl)ethanol (**7b**)

Yield, 53%; light yellow oil. ^1^H NMR (300 MHz, CDCl_3_) *δ* 6.79 (d, *J* = 1.2 Hz, 1H, imidazole H), 6.76 (d, *J* = 1.2 Hz, 1H, imidazole H), 4.82 (q, *J* = 6.6 Hz, 1H, *CH*CH_3_), 4.15–3.91 (m, 2H, *CH_2_*CH_3_), 1.51 (d, *J* = 6.9 Hz, 3H, *CH_3_*CH), 1.35 (t, *J* = 7.3 Hz, 3H, CH_2_*CH_3_*). ^13^C NMR (75 MHz, CDCl_3_) *δ* 149.75, 126.46, 119.19, 62.39, 40.70, 22.03, 16.40.

#### 3.2.3. 1-(1-Propyl-1H-imidazole-2-yl)ethanol (**7c**)

Yield, 72%; light yellow oil. ^1^H NMR (300 MHz, CDCl_3_) *δ* 6.88 (d, *J* = 1.2 Hz, 1H, imidazole H), 6.82 (d, *J* = 1.2 Hz, 1H, imidazole H), 4.88 (q, *J* = 6.6 Hz, 1H, *CH*CH_3_), 4.06–3.83 (m, 2H, *NCH_2_*), 1.88–1.67 (m, 2H, *CH_2_*), 1.58 (d, *J* = 6.6 Hz, 3H, *CH_3_*CH), 0.93 (t, *J* = 7.4 Hz, 3H, CH_2_*CH_3_*). ^13^C NMR (75 MHz, CDCl_3_) *δ* 150.00, 126.43, 119.86, 62.35, 47.55, 24.36, 22.41, 11.17.

#### 3.2.4. 1-(1-Butyl-1H-imidazole-2-yl)ethanol (**7d**)

Yield, 37%; light yellow oil. ^1^H NMR (300 MHz, CDCl_3_) *δ* 6.86 (d, *J* = 1.2 Hz, 1H, imidazole H), 6.82 (d, *J* = 1.2 Hz, 1H, imidazole H), 4.87 (q, *J* = 6.6 Hz, 1H, *CH*CH_3_), 4.10–3.86 (m, 2H, *NCH_2_*), 1.73 (quintet, *J* = 7.5 Hz, 2H, CH_2_*CH_2_*CH_2_), 1.56 (d, *J* = 6.6 Hz, 3H, *CH_3_*CH), 1.34 (sextet, *J* = 7.5 Hz, 2H, CH_2_*CH_2_*CH_3_), 0.93 (t, *J* = 7.3 Hz, 3H, CH_2_*CH_3_*). ^13^C NMR (75 MHz, CDCl_3_) *δ* 150.01, 126.30, 119.83, 62.32, 45.78, 33.13, 22.39, 19.92, 13.68.

#### 3.2.5. 1-(1-secButyl-1H-imidazole-2-yl)ethanol (**7e**)

Yield, 73%; colorless solid. ^1^H NMR (300 MHz, CDCl_3_) *δ* 6.94 (d, *J* = 1.5 Hz, 1H, imidazole H), 6.88 (d, *J* = 1.5 Hz, 1H, imidazole H), 4.91 (q, *J* = 6.6 Hz, 1H, *CH*CH_3_), 4.48–4.24 (m, 1H, *NCH*), 1.88–1.68 (m, 2H, *CH_2_*CH_3_), 1.58 (d, *J* = 6.6 Hz, 3H, *CH_3_*CH), 1.40 (d, *J* = 6.6 Hz, 3H, *CH_3_*CH), 0.83 (t, *J* = 8.3 Hz, 3H, CH_2_*CH_3_*). ^13^C NMR (75 MHz, CDCl_3_) *δ* 149.97, 126.82, 115.92, 62.65, 52.98, 30.69, 22.83, 21.95, 10.72.

#### 3.2.6. 1-(1-Isobutyl-1H-imidazole-2-yl)ethanol (**7f**)

Yield, 76%; colorless oil. ^1^H NMR (300 MHz, CDCl_3_) *δ* 6.91 (d, *J* = 1.2 Hz, 1H, imidazole H), 6.81 (d, *J* = 1.2 Hz, 1H, imidazole H), 4.86 (q, *J* = 6.6 Hz, 1H, *CH*CH_3_), 3.83 (dd, *J* = 14.1, 7.8 Hz, 1H, *NCH_2_*), 3.71 (dd, *J* = 13.8, 7.2 Hz, 1H, *NCH_2_*), 3.30 (br.s, 1H, OH), 2.16–1.96 [m, 1H, *CH*(CH_3_)_2_], 1.60 (d, *J* = 6.6 Hz, 3H, *CH_3_*CH), 0.93 (0.91) [d, *J* = 6.6 Hz, 6H, CH*(CH_3_)_2_*]. ^13^C NMR (75 MHz, CDCl_3_) *δ* 150.28, 126.66, 120.28, 62.27, 53.29, 29.99, 22.59, 20.00.

#### 3.2.7. 1-(1-Isopentyl-1H-imidazole-2-yl)ethanol (**7g**)

Yield, 73%; light yellow oil. ^1^H NMR (300 MHz, CDCl_3_) *δ* 6.79 (s, 2H, imidazole H), 4.83 (q, *J* = 6.6 Hz, 1H, *CH_3_*CH), 4.09–3.86 (m, 2H, *NCH_2_*), 1.72–1.57 (m, 3H, *N*CH_2_*CH_2_CH*), 1.53 (d, *J* = 6.6 Hz, 3H, CH*CH_3_*), 0.91 [d, *J* = 6.3 Hz, 6H, CH*(CH_3_)_2_*]. ^13^C NMR (75 MHz, CDCl_3_) *δ* 149.95, 126.41, 119.66, 62.32, 44.31, 39.99, 25.80, 22.36, 22.21.

#### 3.2.8. 1-[1-(Pentyl-2-yl)-1H-imidazole-2-yl]ethanol (**7h**)

Yield, 74%; colorless oil. ^1^H NMR (300 MHz, CDCl_3_) *δ* 6.83 (s, 2H, imidazole H), 4.84 (q, *J* = 6.6 Hz, 1H, *CH*CH_3_), 4.51–4.39 (m, 2H, *NCH* and OH), 1.79–1.57 (m, 2H), 1.53 (d, *J* = 6.6 Hz, 3H, *CH_3_*CH), 1.33 (d, *J* = 6.9 Hz, 3H, *CH_3_*CH), 1.30–1.01 (m, 2H, *CH_2_*), 0.89–0.79 (m, 3H, *CH_3_*). ^13^C NMR (75 MHz, CDCl_3_) δ 149.91, 126.81, 115.77, 62.60, 51.29, 39.76, 22.35, 22.31, 19.47, 13.81.

#### 3.2.9. 1-[1-(Pentan-3-yl)-1H-imidazole-2-yl]ethanol (**7i**)

Yield, 77%; light yellow solid. ^1^H NMR (300 MHz, CDCl_3_) *δ* 6.95 (d, *J* = 1.5 Hz, 1H, imidazole H), 6.83 (d, *J* = 1.2 Hz, 1H, imidazole H), 4.87 (q, *J* = 6.6 Hz, 1H, *CH*CH_3_), 4.15–3.99 (m, 1H, *NCH*), 1.89–1.61 (m, 4H, 2 x *CH_2_*CH_3_), 1.59 (d, *J* = 6.6 Hz, 3H, *CH_3_*CH), 0.86–0.72 (m, 6H, 2 × CH_2_*CH_3_*). ^13^C NMR (75 MHz, CDCl_3_) *δ* 150.81, 126.85, 115.81, 62.37, 59.05, 29.05, 22.74, 10.59.

### 3.3. Synthesis of 1-(1-Alkyl-1H-imidazol-2-yl)ethenone (***5a**–**5i***)

Dess–Martin periodinane (1.65 mmol) was added to a solution of 1-(1-alkyl-1*H*-imidazole-2-yl)ethanol (**7a**–**7i*,*** 1.09 mmol) in dichloromethane (11.8 mL) at room temperature. The reaction mixture was stirred at room temperature for 30 min before a second batch of Dess–Martin periodinane (1.65 mmol) was added. The mixture was then stirred at room temperature overnight and quenched with sodium thiosulfate (10 mL) and sodium bicarbonate (10 mL). The mixture was extracted with dichloromethane (20 mL × 3), and the combined organic extracts were dried over anhydrous sodium sulfate and concentrated using a rotary evaporator. The crude product was subjected to PTLC purification using 3% methanol in dichloromethane as the eluent to yield the desired 1-(1-alkyl-1*H*-imidazol-2-yl)ethenone (**5a**-**5i**). Their physical and spectral data are summarized below.

#### 3.3.1. 1-(1-Methyl-1H-imidazol-2-yl)ethenone (**5a**)

Yield, 88%; light yellow syrup. ^1^H NMR (300 MHz, CDCl_3_) *δ* 7.12 (s, 1H, imidazole H), 7.01 (s, 1H, imidazole H), 3.98 (s, 3H, *NCH_3_*), 2.64 (s, 3H, C(O)*CH_3_*). ^13^C NMR (75 MHz, CDCl_3_) *δ* 190.57, 143.10, 128.94, 126.95, 27.25, 27.19.

#### 3.3.2. 1-(1-Ethyl-1H-imidazol-2-yl)ethenone (**5b**)

Yield, 57%; light yellow syrup. ^1^H NMR (300 MHz, CDCl_3_) *δ* 7.08 (d, *J* = 1.2 Hz, 1H, imidazole H), 7.04 (d, *J* = 0.9 Hz, 1H, imidazole H), 4.36 (q, *J* = 7.2 Hz, 2H, *NCH_2_*), 2.60 (s, 3H, C(O)*CH_3_*), 1.34 (t, *J* = 7.2 Hz, 3H, CH_2_*CH_3_*). ^13^C NMR (75 MHz, CDCl_3_) *δ* 190.38, 142.51, 129.19, 125.25, 43.79, 27.39, 16.39.

#### 3.3.3. 1-(1-Propyl-1H-imidazol-2-yl)ethenone (**5c**)

Yield, 69%; colorless syrup. ^1^H NMR (300 MHz, CDCl_3_) *δ* 7.12 (d, *J* = 1.0 Hz, 1H, imidazole H), 7.04 (d, *J* = 1.0 Hz, 1H, imidazole H), 4.32 (t, *J* = 7.3 Hz, 2H, *NCH_2_*), 2.63 (s, 3H, C(O)*CH_3_*), 1.76 (sextet, *J* = 7.4 Hz, 2H, *CH_2_*), 0.89 (t, *J* = 7.5 Hz, 3H, CH_2_*CH_3_*). ^13^C NMR (75 MHz, CDCl_3_) *δ* 190.40, 142.64, 129.02, 126.01, 50.27, 27.50, 24.30, 10.93.

#### 3.3.4. 1-(1-Butyl-1H-imidazol-2-yl)ethenone (**5d**)

Yield, 40%; light yellow syrup. ^1^H NMR (300 MHz, CDCl_3_) *δ* 7.13 (d, *J* = 1.0 Hz, 1H, imidazole H), 7.05 (d, *J* = 1.0 Hz, 1H, imidazole H), 4.36 (t, *J* = 7.4 Hz, 2H, *NCH_2_*), 2.65 (s, 3H, C(O)*CH_3_*), 1.73 (quintet, *J* = 7.4 Hz, 2H, *CH_2_*), 1.33 (sextet, *J* = 7.5 Hz, 2H, *CH_2_*), 0.93 (t, *J* = 7.3 Hz, 3H, CH_2_*CH_3_*). ^13^C NMR (75 MHz, CDCl_3_) *δ* 190.47, 142.71, 129.21, 125.85, 48.53, 47.67, 33.11, 27.49, 19.71.

#### 3.3.5. 1-(1-secButyl-1H-imidazol-2-yl)ethenone (**5e**)

Yield, 79%; light yellow syrup. ^1^H NMR (300 MHz, CDCl_3_) *δ* 7.17 (d, *J* = 0.9 Hz, 1H, imidazole H), 7.12 (d, *J* = 1.2 Hz, 1H, imidazole H), 5.36 (sextet, *J* = 6.9 Hz, 1H, N*CH*), 2.61 (s, 3H, C(O)*CH_3_*), 1.69 (quintet, *J* = 7.4 Hz, 2H, CH*CH_2_*CH_3_), 1.35 (d, *J* = 7.4Hz, 3H, CH*CH_3_*), 0.77 (t, *J* = 7.5 Hz, 3H, CH_2_*CH_3_*). ^13^C NMR (75 MHz, CDCl_3_) *δ* 190.95, 142.88, 129.66, 121.38, 54.38, 30.76, 27.95, 21.53, 10.36.

#### 3.3.6. 1-(1-Isobutyl-1H-imidazol-2-yl)ethenone (**5f**)

Yield, 76%; light yellow syrup. ^1^H NMR (300 MHz, CDCl_3_) *δ* 7.12 (d, *J* = 0.9 Hz, 1H, imidazole H), 7.02 (d, *J* = 1.2 Hz, 1H, imidazole H), 4.17 (d, *J* = 7.5 Hz, 2H, *NCH_2_*CH), 2.64 (s, 3H, C(O)*CH_3_*), 2.14–1.94 [m, 1H, *CH*(CH_3_)_2_], 0.88 [d, *J* = 6.9 Hz, 6H, CH*(CH_3_)_2_*]. ^13^C NMR (75 MHz, CDCl_3_) *δ* 190.50, 142.81, 128.89, 126.58, 55.80, 29.87, 27.57, 19.74.

#### 3.3.7. 1-(1-Isopentyl-1H-imidazol-2-yl)ethenone (**5g**)

Yield, 75%; light yellow syrup. ^1^H NMR (300 MHz, CDCl_3_) *δ* 7.10 (d, *J* = 0.9 Hz, 1H, imidazole H), 7.04 (d, *J* = 0.9 Hz, 1H, imidazole H), 4.35 (t, *J* = 7.3 Hz, 2H, *NCH_2_*CH_2_), 2.62 (s, 3H, C(O)*CH_3_*), 1.68–1.47 (m, 3H, *N*CH_2_*CH_2_CH*), 0.91 [d, *J* = 6.3 Hz, 6H, CH*(CH_3_)_2_*]. ^13^C NMR (75 MHz, CDCl_3_) *δ* 190.35, 142.59, 129.07, 125.76, 47.18, 39.98, 27.45, 25.68, 22.34.

#### 3.3.8. 1-[1-(Pentan-2-yl)-1H-imidazol-2-yl]ethenone (**5h**)

Yield, 74%; light yellow syrup. ^1^H NMR (300 MHz, CDCl_3_) *δ* 7.19 (d, *J* = 1.2 Hz, 1H, imidazole H), 7.13 (d, *J* = 0.9 Hz, 1H, imidazole H), 5.48 (sextet, *J* = 6.9 Hz, 1H, N*CH*), 2.63 (s, 3H, C(O)*CH_3_*), 1.76–1.53 (m, 2H, *CH_2_*), 1.36 (d, *J* = 6.9 Hz, 3H, CH*CH_3_*), 1.26–0.96 (m, 2H, *CH_2_*), 0.84 (t, *J* = 7.2 Hz, 3H, CH_2_*CH_3_*). ^13^C NMR (75 MHz, CDCl_3_) *δ* 190.93, 142.77, 129.68, 121.37, 52.79, 39.89, 27.94, 22.02, 19.13, 13.66.

#### 3.3.9. 1-[1-(Pentan-3-yl)-1H-imidazol-2-yl]ethenone (**5i**)

Yield, 67%; light yellow syrup. ^1^H NMR (300 MHz, CDCl_3_) *δ* 7.19 (d, *J* = 1.1 Hz, 1H, imidazole H), 7.16 (d, *J* = 1.1 Hz, 1H, imidazole H), 5.42–5.26 (m, 1H, N*CH*), 2.66 (s, 3H, C(O)*CH_3_*), 1.90–1.54 (m, 4H, 2 × CH_2_*CH_3_*), 0.75 (t, *J* = 7.5 Hz, 6H, 2 × CH_2_*CH_3_*). ^13^C NMR (75 MHz, CDCl_3_) *δ* 190.96, 143.47, 129.64, 121.34, 60.02, 29.05, 28.22, 10.23.

### 3.4. Synthesis of (E)-3(2-Methoxy-4-[(3-methyl-2-buten-1-yl)oxy]phenyl-1-(1-alkyl-1H-imidazol-2-yl)-2-propen-1-one (***3a**–**3i***)

Potassium hydroxide (0.67 mmol) and 2-methoxy-4-[(3-methyl-2-buten-1-yl)oxy]benzaldehyde (**4**, 0.67 mmol) were sequentially added to a solution of 1-(1-alkyl-1*H*-imidazol-2-yl)ethenone (**5a**–**5i**, 0.335 mmol) in ethanol (3.35 mL). The reaction was stirred at room temperature for 12 h and monitored by TLC for completion, after which it was quenched with water (5 mL). The resulting mixture was extracted with ethyl acetate (5 mL × 3). The combined organic layers were dried over anhydrous sodium sulfate and concentrated in vacuo. The crude product was purified by multiple PTLC runs, eluting with 1% methanol in dichloromethane and hexane/ethyl acetate (4:1, *v/v*), to yield the desired chalcones **3a–3i**. Their physical and spectral properties are summarized below.

#### 3.4.1. (E)-3-(2-Methoxy-4-[(3-methyl-2-buten-1-yl)oxy]phenyl-1-(1-methyl-1H-imidazol-2-yl)-2-propen-1-one (**3a**)

Yield, 59%; bright yellow oil. ^1^H NMR (300 MHz, CDCl_3_) *δ* 8.18 (d, *J* = 16.1 Hz, 1H, *trans*-vinylic H), 7.99 (d, *J* = 16.0 Hz, 1H, *trans*-vinylic H), 7.72 (d, *J* = 8.6 Hz, 1H, aromatic H), 7.21 (d, *J* = 1.0 Hz, 1H, imidazole H), 7.05 (d, *J* = 1.0 Hz, 1H, imidazole H), 6.52 (dd, *J* = 8.6, 2.4 Hz, 1H, aromatic H), 6.46 (d, *J* = 2.3 Hz, 1H, aromatic H), 5.51–5.46 (m, 1H, vinylic H), 4.54 (d, *J* = 6.9 Hz, 2H, OCH_2_), 4.09 (s, 3H), 3.87 (s, 3H), 1.78(s, 3H, CH_3_), 1.75 (s, 3H, CH_3_). ^13^C NMR (75 MHz, CDCl_3_) *δ* 180.71, 162.60, 160.40, 144.04, 138.98, 130.38, 130.24, 128.44, 126.87, 120.06, 119.07, 117.04, 105.94, 99.04, 64.98, 55.67, 36.61, 25.94, 18.31. IR (film) *ν*_max_: 3110, 2926, 1651, 1580, 1501 cm^−1^. HRMS: *m/z* calculated for C_19_H_23_N_2_O_3_ [M + H]^+^: 327.1709. Found: 327.1715.

#### 3.4.2. (E)-3-(2-Methoxy-4-[(3-methyl-2-buten-1-yl)oxy]phenyl-1-(1-ethyl-1H-imidazol-2-yl)-2-propen-1-one (**3b**)

Yield, 57%; bright yellow oil. ^1^H NMR (300 MHz, CDCl_3_) *δ* 8.19 (d, *J* = 16.0 Hz, 1H, *trans*-vinylic H), 8.02 (d, *J* = 16.0 Hz, 1H, *trans*-vinylic H), 7.74 (d, *J* = 8.6 Hz, 1H, aromatic H), 7.23 (d, *J* = 1.0 Hz, 1H, imidazole H), 7.12 (d, *J* = 1.0 Hz, 1H, imidazole H), 6.52 (dd, *J* = 8.6, 2.4 Hz, 1H, aromatic H), 6.46 (d, *J* = 2.3 Hz, 1H, aromatic H), 5.55–5.42 (m, 1H, vinylic H), 4.54 (d, *J* = 7.2 Hz, 2H, OCH_2_), 4.54 (q, *J* = 7.2 Hz, 2H, *NCH_2_*CH_3_), 3.87 (s, 3H, OCH_3_), 1.80 (s, 3H, CH_3_), 1.75 (s, 3H, CH_3_), 1.46 (t, *J* = 7.2 Hz, 3H, CH_2_*CH_3_*). ^13^C NMR (75 MHz, CDCl_3_) *δ* 180.41, 162.58, 160.38, 143.38, 138.97, 134.54, 130.18, 128.51, 125.01, 120.32, 119.08, 117.07, 106.02, 99.05, 64.98, 55.71, 44.15, 25.84, 18.21, 16.54. IR (film) *ν*_max_: 3106, 2871, 2934, 1651, 1578, 1500 cm^−1^. HRMS: *m/z* calculated for C_20_H_25_N_2_O_3_ [M + H]^+^: 341.1865. Found: 341.1873.

#### 3.4.3. (E)-3-(2-Methoxy-4-[(3-methyl-2-buten-1-yl)oxy]phenyl-1-(1-propyl-1H-imidazol-2-yl)-2-propen-1-one (**3c**)

Yield, 69%; bright yellow oil. ^1^H NMR (300 MHz, CDCl_3_) *δ* 8.17 (d, *J* = 16.2 Hz, 1H, *trans*-vinylic H), 8.01 (d, *J* = 15.9 Hz, 1H, *trans*-vinylic H), 7.71 (d, *J* = 8.7 Hz, 1H, aromatic H), 7.20 (d, *J* = 0.9 Hz, 1H, imidazole H), 7.09 (d, *J* = 0.9 Hz, 1H, imidazole H), 6.52 (dd, *J* = 8.4, 2.4 Hz, 1H, aromatic H), 6.47 (d, *J* = 2.4 Hz, 1H, aromatic H), 5.67–5.41 (m, 1H, vinylic H), 4.55 (d, *J* = 6.9 Hz, 2H, OCH_2_), 4.45 (t, *J* = 7.2 Hz, 2H, *NCH_2_*CH_2_), 3.86 (s, 3H, OCH_3_), 1.87 (sextet, *J* = 7.5 Hz, 2H, *N*CH_2_*CH_2_*CH_3_), 1.80 (s, 3H, CH_3_), 1.75 (s, 3H, CH_3_), 0.95 (t, *J* = 7.5 Hz, 3H, CH_2_*CH_3_*). ^13^C NMR (151 MHz, CDCl_3_) *δ* 180.64, 162.61, 160.43, 138.92, 132.22, 130.25, 128.67, 125.92, 120.48, 119.20, 117.20, 106.07, 104.83, 99.09, 65.05, 55.63, 50.62, 25.91, 24.57, 18.30, 11.10. IR (film) *ν*_max_: 3105, 2965, 2933, 2875, 1651, 1607, 1578, 1500 cm^−1^. HRMS: *m/z* calculated for C_21_H_27_N_2_O_3_ [M + H]^+^: 355.2022. Found: 355.2029.

#### 3.4.4. (E)-3-(2-Methoxy-4-[(3-methyl-2-buten-1-yl)oxy]phenyl-1-(1-butyl-1H-imidazol-2-yl)-2-propen-1-one (**3d**)

Yield, 41%; bright yellow oil. ^1^H NMR (300 MHz, CDCl_3_) *δ* 8.17 (d, *J* = 16.2 Hz, 1H, *trans*-vinylic H), 8.00 (d, *J* = 16.2 Hz, 1H, *trans*-vinylic H), 7.70 (d, *J* = 8.7 Hz, 1H, aromatic H), 7.19 (d, *J* = 0.9 Hz, 1H, imidazole H), 7.08 (d, *J* = 0.9 Hz, 1H, imidazole H), 6.51 (dd, *J* = 8.7, 2.4 Hz, 1H, aromatic H), 6.46 (d, *J* = 2.4 Hz, 1H, aromatic H), 5.69–5.37 (m, 1H, vinylic H), 4.54 (d, *J* = 6.9 Hz, 2H, OCH_2_), 4.48 (t, *J* = 7.4 Hz, 2H, *NCH_2_*CH_2_), 3.86 (s, 3H, OCH_3_), 1.85–1.74 (overlapped, 2H, *N*CH_2_*CH_2_*CH_2_), 1.80 (s, 3H, CH_3_), 1.75 (s, 3H, CH_3_), 1.37 (sextet, *J* = 7.5 Hz, 2H, CH_2_*CH_2_*CH_3_), 0.94 (t, *J* = 7.4 Hz, 3H, CH_2_*CH_3_*). ^13^C NMR (75 MHz, CDCl_3_) *δ* 180.95, 162.55, 160.42, 144.02, 139.05, 138.60, 130.24, 129.12, 126.04, 120.63, 119.25, 117.27, 106.02, 99.10, 65.09, 55.73, 48.87, 33.44, 25.98, 19.96, 18.35, 13.82. IR (film) *ν*_max_: 3106, 2958, 2931, 2872, 1651, 1607, 1579, 1501 cm^−1^. HRMS: *m/z* calculated for C_22_H_29_N_2_O_3_ [M + H]^+^: 369.2178. Found: 369.2187.

#### 3.4.5. (E)-3-(2-Methoxy-4-[(3-methyl-2-buten-1-yl)oxy]phenyl-1-(1-secbutyl-1H-imidazol-2-yl)-2-propen-1-one (**3e**)

Yield, 27%; bright yellow oil. ^1^H NMR (300 MHz, CDCl_3_) *δ* 8.17 (d, *J* = 16.2 Hz, 1H, *trans*-vinylic H), 8.03 (d, *J* = 16.2 Hz, 1H, *trans*-vinylic H), 7.72 (d, *J* = 8.4 Hz, 1H, aromatic H), 7.24 (s, 2H, imidazole H), 6.52 (dd, *J* = 8.7, 2.4 Hz, 1H, aromatic H), 6.47 (d, *J* = 2.4 Hz, 1H, aromatic H), 5.63 (sextet, *J* = 6.9 Hz, 1H, *N*CH), 5.56–5.42 (m, 1H, vinylic H), 4.55 (d, *J* = 6.9 Hz, 2H, OCH_2_), 3.87 (s, 3H, OCH_3_), 1.85–1.71 (overlapped, 2H, *CH_2_*CH_3_), 1.81 (s, 3H, CH_3_), 1.74 (s, 3H, CH_3_), 1.46 (d, *J* = 6.9 Hz, 3H, CH*CH_3_*), 0.86 (t, *J* = 7.4 Hz, 3H, CH_2_*CH_3_*). IR (film) *ν*_max_: 3106, 2966, 2933, 2876, 1651, 1577, 1501 cm^−1^. HRMS: *m/z* calculated for C_22_H_29_N_2_O_3_ [M + H]^+^: 369.2178. Found: 369.2188.

#### 3.4.6. (E)-3-(2-Methoxy-4-[(3-methyl-2-buten-1-yl)oxy]phenyl-1-(1-isobutyl-1H-imidazol-2-yl)-2-propen-1-one (**3f**)

Yield, 38%; bright yellow oil. ^1^H NMR (300 MHz, CDCl_3_) *δ* 8.17 (d, *J* = 16.1 Hz, 1H, *trans*-vinylic H), 8.02 (d, *J* = 16.1 Hz, 1H, *trans*-vinylic H), 7.71 (d, *J* = 8.6 Hz, 1H, aromatic H), 7.20 (d, *J* = 1.0 Hz, 1H, imidazole H), 7.06 (d, *J* = 1.0 Hz, 1H, imidazole H), 6.52 (dd, *J* = 8.6, 2.4 Hz, 1H, aromatic H), 6.46 (d, *J* = 2.3 Hz, 1H, aromatic H), 5.55–5.42 (m, 1H, vinylic H), 4.54 (d, *J* = 7.1 Hz, 2H, OCH_2_), 4.29 (d, *J* = 7.3 Hz, 2H, *NCH_2_*CH), 3.86 (s, 3H, OCH_3_), 2.22–2.09 [m, 1H, CH_2_*CH*(CH_3_)_2_], 1.80 (s, 3H, CH_3_), 1.75 (s, 3H, CH_3_), 0.92 [d, *J* = 6.7 Hz, 6H, CH*(CH_3_)_2_*]. ^13^C NMR (75 MHz, CDCl_3_) *δ* 180.80, 162.60, 160.45, 143.94, 139.02, 138.78, 130.20, 128.60, 126.57, 120.55, 119.22, 117.22, 106.02, 99.07, 65.08, 56.18, 30.02, 26.02, 25.94, 19.90, 18.40. IR (film) *ν*_max_: 3106, 2933, 2871, 1651, 1607, 1579, 1500 cm^−1^. HRMS: *m/z* calculated for C_22_H_29_N_2_O_3_ [M + H]^+^: 369.2178. Found: 369.2186.

#### 3.4.7. (E)-3-(2-Methoxy-4-[(3-methyl-2-buten-1-yl)oxy]phenyl-1-(1-isopentyl-1H-imidazol-2-yl)-2-propen-1-one (**3g**)

Yield, 93%; bright yellow oil. ^1^H NMR (300 MHz, CDCl_3_) *δ* 8.18 (d, *J* = 16.2 Hz, 1H, *trans*-vinylic H), 8.01 (d, *J* = 15.9 Hz, 1H, *trans*-vinylic H), 7.71 (d, *J* = 8.7 Hz, 1H, aromatic H), 7.20 (d, *J* = 0.9 Hz, 1H, imidazole H), 7.09 (d, *J* = 0.9 Hz, 1H, imidazole H), 6.51 (dd, *J* = 8.6, 2.3 Hz, 1H, aromatic H), 6.46 (d, *J* = 2.4 Hz, 1H, aromatic H), 5.54–5.43 (m, 1H, vinylic H), 4.54 (d, *J* = 6.9 Hz, 2H, OCH_2_), 4.49 (t, *J* = 8.1 Hz, 2H, *NCH_2_*CH_2_), 3.86 (s, 3H, OCH_3_), 1.80 (s, 3H, CH_3_), 1.75 (s, 3H, CH_3_), 1.73–1.68 [overlapped, 3H, *CH_2_CH*(CH_3_)_2_], 0.97 [d, *J* = 6.6 Hz, 6H, CH_2_CH*(CH_3_)_2_*]. ^13^C NMR (75 MHz, CDCl_3_) *δ* 180.63, 162.48, 160.33, 143.70, 138.89, 138.66, 130.14, 128.86 125.65, 120.46, 119.12, 117.13, 105.93, 98.99, 64.96, 55.57, 47.42, 40.14, 25.88, 25.82, 22.43, 18.22. IR (film) *ν*_max_: 3105, 2955, 2931, 2869, 1651, 1607, 1580, 1501 cm^−1^. HRMS: *m/z* calculated for C_23_H_31_N_2_O_3_ [M + H]^+^: 383.2335. Found: 383.2342.

#### 3.4.8. (E)-3-(2-Methoxy-4-[(3-methyl-2-buten-1-yl)oxy]phenyl-1-[1-(pentan-2-yl)-1H-imidazol-2-yl]-2-propen-1-one (**3h**)

Yield, 72%; bright yellow oil. ^1^H NMR (300 MHz, CDCl_3_) *δ* 8.16 (d, *J* = 15.9 Hz, 1H, *trans*-vinylic H), 8.02 (d, *J* = 15.9 Hz, 1H, *trans*-vinylic H), 7.71 (d, *J* = 8.4 Hz, 1H, aromatic H), 7.25 (d, *J* = 1.2 Hz, 1H, imidazole H), 7.23 (d, *J* = 1.2 Hz, 1H, imidazole H), 6.52 (dd, *J* = 8.4, 2.4 Hz, 1H, aromatic H), 6.46 (d, *J* = 2.4 Hz, 1H, aromatic H), 5.75 (sextet, *J* = 6.9 Hz, 1H, *N*CH), 5.54–5.42 (m, 1H, vinylic H), 4.54 (d, *J* = 6.9 Hz, 2H, OCH_2_), 3.86 (s, 3H, OCH_3_), 1.80 (s, 3H, CH_3_), 1.75 (s, 3H, CH_3_), 1.74–1.62 (m, 2H, CH*CH_2_*CH_2_), 1.44 (d, *J* = 6.9 Hz, 3H, CH*CH_3_*), 1.33–1.17 (m, 2H, CH_2_*CH_2_*CH_3_), 0.88 (t, *J* = 7.7 Hz, 3H, CH_2_*CH_3_*).^13^C NMR (75 MHz, CDCl_3_) *δ* 181.26, 162.55, 160.41, 144.03, 139.01, 138.73, 130.26, 129.53, 121.23, 121.15, 119.24, 117.29, 106.03, 99.11, 65.08, 55.70, 53.03, 40.13, 25.96, 22.23, 19.37, 18.37, 13.89. IR (film) *ν*_max_: 3105, 2960, 2931, 2872, 1651, 1607, 1577, 1502 cm^−1^. HRMS: *m/z* calculated for C_23_H_31_N_2_O_3_ [M + H]^+^: 383.2335. Found: 383.2344.

#### 3.4.9. (E)-3-(2-Methoxy-4-[(3-methyl-2-buten-1-yl)oxy]phenyl-1-(1-(pentan-3-yl)-1H-imidazol-2-yl)-2-propen-1-one (**3i**)

Yield, 92%; bright yellow oil. ^1^H NMR (300 MHz, CDCl_3_) *δ* 8.16 (d, *J* = 15.9 Hz, 1H, *trans*-vinylic H), 8.03 (d, *J* = 15.9 Hz, 1H, *trans*-vinylic H), 7.71 (d, *J* = 8.7 Hz, 1H, aromatic H), 7.26 (d, *J* = 1.2 Hz, 1H, imidazole H), 7.19 (d, *J* = 1.2 Hz, 1H, imidazole H), 6.52 (dd, *J* = 8.4, 2.4 Hz, 1H, aromatic H), 6.46 (d, *J* = 2.4 Hz, 1H, aromatic H), 5.63–5.52 (m, 1H, *N*CH), 5.52–5.43 (m, 1H, vinylic H), 4.54 (d, *J* = 6.9 Hz, 2H, OCH_2_), 3.86 (s, 3H, OCH_3_), 1.96–1.62 (m, 4H, 2 × *CH_2_*CH_3_), 1.80 (s, 3H, CH_3_), 1.75 (s, 3H, CH_3_), 0.81 (t, *J* = 7.4 Hz, 6H, 2 × CH_2_*CH_3_*). ^13^C NMR (75 MHz, CDCl_3_) *δ* 181.29, 162.55, 160.40, 144.84, 139.02, 138.75, 130.23, 129.65, 121.23, 121.19, 119.21, 117.25, 106.03, 99.11, 65.06, 60.20, 55.71, 29.25, 26.00, 18.33, 10.46. IR (film) *ν*_max_: 3105, 2965, 2933, 2876, 1652, 1579, 1502 cm^−1^. HRMS: *m/z* calculated for C_23_H_31_N_2_O_3_ [M + H]^+^: 383.2335. Found: 383.2345.

### 3.5. Claisen Rearrangement of (E)-3(2-Methoxy-4-[(3-methyl-2-buten-1-yl)oxy]phenyl-1-(1-alkyl-1H-imidazol-2-yl)-2-propen-1-one

#### 3.5.1. Conversion of Chalcones **3c** and **3g** to Ester **8**

Chalcone **3c** (28 mg) or chalcone **3g** (28 mg) was dissolved in ethanol and water (5 mL, 4:1 *v/v*) in a sealed tube. The solution was heated at 160 °C overnight and then allowed to cool to room temperature. After cooling, the mixture was diluted with brine (10 mL) and extracted with dichloromethane (5 mL × 3). The combined organic layers were dried over anhydrous sodium sulfate and concentrated under reduced pressure to yield ester **8** as a yellow-green syrup in 95% yield. ^1^H NMR (300 MHz, CDCl_3_) δ 7.89 (d, J = 16.2 Hz, 1H, trans-vinylic H), 7.38 (s, 1H), 6.45 (d, J = 15.6 Hz, 1H, trans-vinylic H), 6.42 (s, 1H), 6.22 (br.s, 1H, OH), 6.18 (dd, J = 18.0, 10.8 Hz, 1H, vinylic H), 5.37 (dd, J = 18.0, 0.9 Hz, 1H, vinylic H), 5.33 (dd, J = 10.2, 0.9 Hz, 1H, vinylic H), 4.25 (q, J = 7.2 Hz, 2H, OCH_2_CH_3_), 3.84 (s, 3H, OCH_3_), 1.43 (s, 6H, 2 × CH_3_), 1.33 (t, J = 7.2 Hz, 3H, OCH_2_*CH_3_*). ^13^C NMR (75 MHz, CDCl_3_) δ 168.17 (C), 159.14 (C), 158.04 (C), 147.95 (CH), 140.61 (CH), 127.89 (CH), 124.50 (C), 116.08 (C), 116.06 (C), 114.10 (CH_2_), 101.17 (CH), 60.30 (CH_2_), 55.68 (CH_3_), 39.89 (C), 27.22 (CH_3_), 14.56 (CH_3_). C, CH, CH_2_, and CH_3_ were assigned based on DEPT. IR (film) *ν*_max_: 3342, 2964, 1702, 1678, 1600, 1504, 1463, 1156 cm^−1^. HRMS: *m/z* calculated for C_17_H_23_O_4_ [M + H]^+^: 291.1596. Found: 291.1593.

#### 3.5.2. Conversion of Chalcone **3c** to Ester **9**

Chalcone **3c** was converted to ester **9** under the Claisen rearrangement conditions using 1-propanol–water (4:1 *v/v*) as solvent. Yield: 26%; Yellow-green syrup. ^1^H NMR (300 MHz, CDCl_3_) δ 7.90 (d, J = 16.2 Hz, 1H, trans-vinylic H), 7.38 (s, 1H, aromatic H), 6.46 (d, J = 16.2 Hz, 1H, trans-vinylic H), 6.42 (s, 1H, aromatic H), 6.22 (br.s., 1H, OH), 6.18 (dd, J = 17.7, 10.5 Hz, 1H, vinylic H), 5.38 (dd, J = 17.7, 0.9 Hz, 1H, vinylic H), 5.34 (dd, J = 10.5, 0.9 Hz, 1H, vinylic H), 4.15 (t, J = 6.9 Hz, 2H, O*CH_2_*CH_2_CH_3_), 3.84 (s, 3H, OCH_3_), 1.73 (sextet, J = 7.2 Hz, 2H, OCH_2_*CH*_2_CH_3_), 1.43 (s, 6H, 2 × CH_3_), 0.99 (t, J = 7.2 Hz, 3H, OCH_2_CH_2_*CH_3_*). ^13^C NMR (75 MHz, CDCl_3_) δ 168.30, 159.12, 158.02, 147.96, 140.62, 127.75, 124.43, 116.05, 115.95, 114.18, 101.20, 66.00, 55.81, 39.88, 27.24, 22.31, 10.61. IR (film) *ν*_max_: 3325, 2964, 1703, 1676, 1600, 1504, 1446, 1158 cm^−1^. HRMS: *m/z* calculated for C_18_H_25_O_4_ [M + H]^+^: 305.1753. Found: 305.1749.

#### 3.5.3. Conversion of Chalcone **3a** to Ester **10**

Chalcone **3a** was converted to ester **10** under the Claisen rearrangement conditions using methanol–water (4:1 *v/v*) as solvent. Yield: 36%; Yellow-green syrup. ^1^H NMR (300 MHz, CDCl_3_) δ 7.90 (d, J = 15.9 Hz, 1H, trans-vinylic H), 7.37 (s, 1H, aromatic H), 6.46 (d, J = 15.9 Hz, 1H, trans-vinylic H), 6.42 (s, 1H), 6.23 (br.s., 1H, OH), 6.17 (dd, J = 17.7, 10.5 Hz, 1H, vinylic H), 5.38 (dd, J = 17.7, 0.9 Hz, 1H, vinylic H), 5.34 (dd, J = 10.5, 1.2 Hz, 1H, vinylic H), 3.84 (s, 3H, OCH_3_), 3.79 (s, 3H, OCH_3_), 1.42 (s, 6H, 2 × CH_3_). ^13^C NMR (75 MHz, CDCl_3_) δ 168.61, 159.16, 158.10, 147.92, 140.94, 127.76, 124.46, 115.94, 115.48, 114.19, 101.22, 55.83, 51.77, 39.87, 27.22. IR (film) *ν*_max_: 3329, 2964, 1702, 1682, 1604, 1506, 1446, 1290, 1165. HRMS: *m/z* calculated for C_16_H_21_O_4_ [M + H]^+^: 277.1440. Found: 277.1435.

### 3.6. Synthesis of 5-(1,1,-Dimethyl-2-propen-1-yl)-4-hydroxy-2-methoxybenzaldehyde (***11***)

2-Methoxy-4-[(3-methyl-2-buten-1-yl)oxy]benzaldehyde (**4**, 696 mg, 3.16 mmol) was dissolved in ethanol and water (50 mL, 4:1 *v/v*) in a sealed tube. The solution was heated at 169 °C for three days and then allowed to cool to room temperature. After cooling, the mixture was diluted with brine (100 mL) and extracted with dichloromethane (50 mL × 3). The combined organic layers were dried over anhydrous sodium sulfate and concentrated in vacuo. The crude product was purified by PTLC using 2% methanol in dichloromethane as the eluent, yielding 170 mg of the Claisen rearrangement product as a pale-yellow wax in 24% yield alongside 338 mg of the starting material. The yield, based on recovered starting material, is 47%. The ^1^H NMR data are consistent with those previously reported in the literature [24].

### 3.7. Synthesis of Aldehyde ***12*** via Methylation of Aldehyde ***11***

A three-neck round bottom flask was charged with 5-(1,1,-dimethyl-2-propen-1-yl)-4-hydroxy-2-methoxybenzaldehyde (**11**, 56 mg, 0.254 mmol) and potassium carbonate (72 mg, 0.52 mmol). The flask was evacuated three times under argon before acetone (2 mL) was added. The reaction mixture was refluxed for 15 min, after which dimethylsulfate (64 uL, 0.68 mmol) was added via a needle. The mixture was then refluxed for an additional 4 h, with completion monitored by TLC. After cooling to room temperature, saturated ammonium chloride (5 mL) was added to quench the reaction. The resulting mixture was extracted with ethyl acetate (5 mL × 3), and the combined organic layers were washed with brine (3 mL), dried over anhydrous sodium sulfate, and concentrated in vacuo. The crude product was purified by PTLC using hexane/ethyl acetate as the eluent, yielding the desired aldehyde **12** as an off-white powder in 96% yield. ^1^H NMR (300 MHz, CDCl_3_) *δ* 10.29 (s, 1H, aldehyde H), 7.77 (s, 1H, aromatic H), 6.40 (s, 1H, aromatic H), 6.10 (dd, *J* = 17.4, 10.5 Hz, 1H, vinylic H), 4.93 (dd, *J* = 10.8, 1.2 Hz, 1H, vinylic H), 4.89 (dd, *J* = 17.4, 1.2 Hz, 1H, vinylic H), 3.92 (s, 3H, OCH_3_), 3.87 (s, 3H, OCH_3_), 1.42 (s, 6H, 2 × CH_3_). ^13^C NMR (75 MHz, CDCl_3_) *δ* 188.70, 164.90, 162.89, 147.57, 129.49, 127.66, 117.54, 110.31, 95.29, 55.91, 55.52, 40.15, 27.52.

### 3.8. Synthesis of Chalcones ***13a**–**13i***

Potassium hydroxide (0.18 mmol) was added to a solution of 1-(1-alkyl-1*H*-imidazol-2-yl)ethenone (**5a**–**5i**, 0.083 mmol) in ethanol (0.5 mL). The solution was stirred for 10 min before a solution of aldehyde **12** (0.67 mmol) in ethanol (0.5 mL) was added. The reaction was stirred at room temperature overnight and monitored by TLC for completion, after which it was quenched with water (5 mL). The resulting mixture was extracted with ethyl acetate (5 mL × 3). The combined organic layers were dried over anhydrous sodium sulfate and concentrated in vacuo. The crude product was purified by multiple PTLC runs, eluting with hexane/ethyl acetate (4:1, *v/v*), to yield the desired chalcones **13a**–**13i**. Their physical and spectral properties are summarized below.

#### 3.8.1. Chalcone **13a**

Yield: 98%; yellow syrup. ^1^H NMR (300 MHz, CDCl_3_) *δ* 8.17 (d, *J* = 16.2 Hz, 1H, *trans*-vinylic H), 7.95 (d, *J* = 16.2 Hz, 1H, *trans*-vinylic H), 7.61 (s, 1H, aromatic H), 7.18 (d, *J* = 1.2 Hz, 1H, imidazole H), 7.04 (d, *J* = 1.2 Hz, 1H, imidazole H), 6.41 (s, 1H, aromatic H), 6.14 (dd, *J* = 17.4, 10.8 Hz, 1H, vinylic H), 4.93 (dd, *J* = 10.8, 1.2 Hz, 1H, vinylic H), 4.90 (dd, *J* = 17.4, 1.2 Hz, 1H, vinylic H), 4.06 (s, 3H, *NCH_3_*), 3.89 (s, 3H, O*CH_3_*), 3.83 (s, 3H, O*CH_3_*), 1.44 (s, 6H, 2 × *CH_3_*). ^13^C NMR (151 MHz, CDCl_3_) 181.08, 161.78, 159.35, 147.86, 144.29, 139.72, 129.16, 128.72, 128.14, 126.84, 120.02, 115.64, 110.07, 95.97, 55.79, 55.28, 40.11, 36.48, 27.57. IR (film) *ν*_max_: 3079, 2961, 2837, 1651, 1579, 1498, 1274 cm^−1^. HRMS: *m/z* calculated for C_20_H_25_N_2_O_3_ [M + H]^+^: 341.1865. Found: 341.1870.

#### 3.8.2. Chalcone **13b**

Yield: 93%; yellow syrup. ^1^H NMR (300 MHz, CDCl_3_) *δ* 8.18 (d, *J* = 16.2 Hz, 1H*, trans*-vinylic H), 7.98 (d, *J* = 15.9 Hz, 1H, *trans*-vinylic H), 7.62 (s, 1H, aromatic H), 7.21 (s, 1H, imidazole H), 7.11 (s, 1H, imidazole H), 6.42 (s, 1H), aromatic H), 6.14 (dd, *J* = 17.4, 10.8 Hz, 1H, vinylic H), 4.93 (dd, *J* = 10.8, 1.2 Hz, 1H, vinylic H), 4.91 (dd, *J* = 17.4, 1.2 Hz, 1H, vinylic H), 4.53 (q, *J* = 7.2 Hz, 2H, *NCH_2_*CH_3_), 3.90 (s, 3H, OCH_3_), 3.84 (s, 3H, OCH_3_), 1.45 (t, *J* = 7.2 Hz, 3H, CH_2_*CH_3_*)1.44 (s, 6H, 2 × CH_3_). ^13^C NMR (151 MHz, CDCl_3_) *δ* 180.94, 161.71, 159.31, 147.87, 143.77, 139.52, 129.14, 129.02, 128.10, 125.13, 120.27, 115.70, 110.06, 95.97, 55.78, 55.28, 44.07, 40.11, 27.57, 16.66. IR (film) *ν*_max_: 2966, 2837, 1652, 1581, 1499, 1408 cm^−1^. HRMS: *m/z* calculated for C_21_H_27_N_2_O_3_ [M + H]^+^: 355.2021. Found: 355.2027.

#### 3.8.3. Chalcone **13c**

Yield: 72%; yellow syrup. ^1^H NMR (300 MHz, CDCl_3_) *δ* 8.20 (d, *J* = 15.9 Hz, 1H, *trans*-vinylic H), 8.04 (d, *J* = 15.9 Hz, 1H, *trans*-vinylic H), 7.66 (s, 1H, aromatic H), 7.26 (d, *J* = 1.2 Hz, 1H, imidazole H), 7.12 (d, *J* = 1.2 Hz, 1H, imidazole H), 6.42 (s, 1H, aromatic H), 6.15 (dd, *J* = 17.1, 10.5 Hz, 1H, vinylic H), 4.94 (dd, *J* = 10.8, 1.2 Hz, 1H, vinylic H), 4.91 (dd, *J* = 17.1, 1.2 Hz, 1H, vinylic H), 4.47 (t, *J* = 7.5 Hz, 2H, *NCH_2_*CH_2_), 3.92 (s, 3H, OCH_3_), 3.85 (s, 3H, OCH_3_), 1.86 (sextet, *J* = 7.5 Hz, 2H, CH_2_*CH_2_*CH_3_), 1.45 (s, 6H, 2 x CH_3_), 0.95 (t, *J* = 7.5 Hz, 3H, CH_2_*CH_3_*). ^13^C NMR (151 MHz, CDCl_3_) *δ* 180.40, 162.02, 159.58, 147.93, 143.29, 140.36, 129.31, 128.49, 127.96, 125.80, 120.08, 115.70, 110.11, 95.96, 55.86, 55.34, 50.83, 40.19, 27.65, 24.60, 11.13. IR (film) *ν*_max_: 2964, 2875, 1652, 1581, 1499, 1407, 1275 cm^−1^. HRMS: *m/z* calculated for C_22_H_29_N_2_O_3_ [M + H]^+^: 369.2178. Found: 369.2184.

#### 3.8.4. Chalcone **13d**

Yield: 52%; yellow-green syrup. ^1^H NMR (300 MHz, CDCl_3_) *δ* 8.20 (d, *J* = 15.9 Hz, 1H, *trans*-vinylic H), 8.03 (d, *J* = 15.9 Hz, 1H, *trans*-vinylic H), 7.64 (s, 1H, aromatic H), 7.24 (d, *J* = 0.9 Hz, 1H, imidazole H), 7.11 (d, *J* = 1.2 Hz, 1H, imidazole H), 6.42 (s, 1H, aromatic H), 6.14 (dd, *J* = 17.4, 10.8 Hz, 1H, vinylic H), 4.93 (dd, *J* = 10.8, 1.5 Hz, 1H, vinylic H), 4.91 (dd, *J* = 17.4, 1.5 Hz, 1H, vinylic H), 4.49 (t, *J* = 7.5 Hz, 2H, *N*CH_2_), 3.91 (s, 3H, OCH_3_), 3.85 (s, 3H, OCH_3_), 1.86—1.76 (m, 2H, CH_2_), 1.45 (s, 6H, 2 × CH_3_), 1.41—1.31 (m, 2H, CH_2_), 0.94 (t, *J* = 7.2 Hz, 3H, CH_3_). ^13^C NMR (75 MHz, CDCl_3_) *δ* 180.83, 161.98, 159.55, 147.93, 143.23, 140.33, 129.20, 128.56, 128.37, 125.72, 119.97, 115.60, 110.11, 95.80, 55.71, 55.44, 49.09, 40.16, 33.35, 27.65, 19.91, 13.77. IR (film) *ν*_max_: 2960, 2873, 1652, 1583, 1499, 1277, 1024 cm^−1^. HRMS: *m/z* calculated for C_23_H_31_N_2_O_3_ [M + H]^+^: 383.2334. Found: 383.2334.

#### 3.8.5. Chalcone **13e**

Yield: 84%; yellow syrup. ^1^H NMR (300 MHz, CDCl_3_) *δ* 8.19 (d, *J* = 15.9 Hz, 1H, *trans*-vinylic H), 8.04 (d, *J* = 15.9 Hz, 1H, *trans*-vinylic H), 7.65 (s, 1H, aromatic H), 7.29 (d, *J* = 1.2 Hz, 1H, imidazole H), 7.26 (d, *J* = 1.2 Hz, 1H, imidazole H), 6.42 (s, 1H, aromatic H), 6.15 (dd, *J* = 17.1, 10.8 Hz, 1H, vinylic H), 5.63 (Sextet, *J* = 6.9 Hz, 1H, *NCH*), 4.94 (dd, *J* = 10.8, 1.2 Hz, 1H, vinylic H), 4.91 (dd, *J* = 17.4, 1.2 Hz, 1H, vinylic H), 3.92 (s, 3H, OCH_3_), 3.85 (s, 3H, OCH_3_), 1.87—1.72 (m, 2H, CH_2_), 1.46 (d, *J* = 6.9 Hz, 3H, CH*CH_3_*), 1.45 (s, 6H, 2 × CH_3_), 0.85 (t, *J* = 7.2 Hz, 3H, CH_2_*CH_3_*). ^13^C NMR (75 MHz, CDCl_3_) *δ* 180.84, 161.94, 159.50, 147.93, 143.52, 140.28, 129.18, 128.47, 128.33, 121.04, 120.52, 115.63, 110.09, 95.90, 55.40, 54.88, 54.82, 40.15, 30.98, 27.63, 21.68, 10.55. IR (film) *ν*_max_: 3079, 2964, 1652, 1580, 1499, 1403, 1389, 1275, 1025 cm^−1^. HRMS: *m/z* calculated for C_23_H_31_N_2_O_3_ [M + H]^+^: 383.2334. Found: 383.2340.

#### 3.8.6. Chalcone **13f**

Yield: 98%; yellow syrup. ^1^H NMR (300 MHz, CDCl_3_) *δ* 8.20 (d, *J* = 15.9 Hz, 1H, *trans*-vinylic H), 8.07 (d, *J* = 15.9 Hz, 1H, *trans*-vinylic H), 7.66 (s, 1H, aromatic H), 7.28 (s, 1H, imidazole H), 7.09 (S, 1H, imidazole H), 6.42 (s, 1H, aromatic H), 6.15 (dd, *J* = 17.1, 10.8 Hz, 1H, vinylic H), 4.94 (dd, *J* = 10.8, 1.2 Hz, 1H, vinylic H), 4.91 (dd, *J* = 17.4, 1.2 Hz, 1H, vinylic H), 4.32 (d, *J* = 7.5 Hz, 2H, *NCH_2_*), 3.93 (s, 3H, OCH_3_), 3.85 (s, 3H, OCH_3_), 2.23–2.09 (m, 1H, CH), 1.46 (s, 6H, 2 × CH_3_), 0.93 [d, 6H, CH*(CH_3_)_2_*].^13^C NMR (75 MHz, CDCl_3_) *δ* 179.95, 162.15, 159.68, 147.87, 142.81, 140.83, 129.25, 128.60, 127.33, 126.38, 119.80, 115.49, 110.08, 95.82, 56.39, 55.87, 55.25, 40.14, 30.00, 27.58, 19.83. IR (film) *ν*_max_: 3079, 2960, 2871, 1650, 1580, 1498, 1406, 1273, 1021 cm^−1^. HRMS: *m/z* calculated for C_23_H_31_N_2_O_3_ [M + H]^+^: 383.2334. Found: 383.2341.

#### 3.8.7. Chalcone **13g**

Yield: 76%; yellow-green syrup. ^1^H NMR (300 MHz, CDCl_3_) *δ* 8.18 (d, *J* = 15.9 Hz, 1H, *trans*-vinylic H), 8.00 (d, *J* = 15.9 Hz, 1H, trans-vinylic H), 7.62 (s, 1H, aromatic H), 7.21 (d, *J* = 1.2 Hz, 1H, imidazole H), 7.10 (d, *J* = 0.9 Hz, 1H, imidazole H), 6.42 (s, 1H, aromatic H), 6.14 (dd, *J* = 17.4, 10.8 Hz, 1H, vinylic H), 4.94 (dd, *J* = 10.8, 1.5 Hz, 1H, vinylic H), 4.91 (dd, *J* = 17.4, 1.5 Hz, 1H, vinylic H), 4.50 (t, *J* = 7.5 Hz, 2H, *N*-CH_2_), 3.91 (s, 3H, OCH_3_), 3.85 (s, 3H, OCH_3_), 1.75–1.60 (m, 3H), 1.14 (s, 6H, 2 × CH_3_), 0.97 (d, *J* = 6.3 Hz, 6H, 2 × CH_3_). ^13^C NMR (75 MHz, CDCl_3_) *δ* 180.83, 161.76, 159.38, 147.91, 143.71, 139.74, 129.08, 128.69, 128.19, 125.78, 120.21, 115.63, 110.11, 95.92, 56.22, 55.65, 47.57, 40.23, 40.13, 27.62, 25.92, 22.53. IR (film) *ν*_max_: 2959, 2871, 1653, 1585, 1499, 1277 cm^−1^. HRMS: *m/z* calculated for C_24_H_33_N_2_O_3_ [M + H]^+^: 397.2491. Found: 397.2490.

#### 3.8.8. Chalcone **13h**

Yield: 68%; yellow syrup. ^1^H NMR (300 MHz, CDCl_3_) *δ* 8.18 (d, *J* = 15.9 Hz, 1H, *trans*-vinylic H), 8.01 (d, *J* = 15.9 Hz, 1H, *trans*-vinylic H), 7.62 (s, 1H, aromatic H), 7.25 (s, 2H, imidazole H), 6.42 (s, 1H, aromatic H), 6.15 (dd, *J* = 17.4, 10.8 Hz, 1H, vinylic H), 5.72 (sextet, *J* = 8.1 Hz, 1H, *NCH*), 4.94 (dd, *J* = 10.8, 1.5 Hz, 1H, vinylic H), 4.91 (dd, *J* = 17.4, 1.5 Hz, 1H, vinylic H), 3.91 (s, 3H, OCH_3_), 3.85 (s, 3H, OCH_3_), 1.79–1.66 (m, 2H, *CH_2_*CH_2_CH_3_), 1.45 (s, 6H, 2 × CH_3_), 1.45 (d, *J* = 6.9 Hz, 3H, *CH_3_*CH), 1.31–1.15 (m, 2H, CH_2_*CH_2_*CH_3_), 0.89 (t, *J* = 7.2 Hz, 3H, CH_2_CH_2_*CH_3_*). ^13^C NMR (75 MHz, CDCl_3_) *δ* 181.27, 161.77, 159.37, 147.96, 143.90, 139.74, 129.12, 128.34, 128.13, 121.09, 120.76, 115.71, 110.11, 95.86, 55.69, 55.44, 53.04, 40.15, 27.64, 27.57, 22.26, 19.36, 13.84. IR (film) *ν*_max_: 3079, 2960, 2932, 2872, 1652, 1580, 1499, 1456, 1437, 1403, 1276 cm^−1^. HRMS: *m/z* calculated for C_24_H_33_N_2_O_3_ [M + H]^+^: 397.2491. Found: 397.2496.

#### 3.8.9. Chalcone **13i**

Yield: 85%; yellow syrup. ^1^H NMR (300 MHz, CDCl_3_) *δ* 8.17 (d, *J* = 16.2 Hz, 1H, *trans*-vinylic H), 8.02 (d, *J* = 16.2 Hz, 1H, *trans*-vinylic H), 7.62 (s, 1H, aromatic H), 7.27 (d, *J* = 0.9 Hz, 1H, imidazole H), 7.20 (d, *J* = 0.9 Hz, 1H, imidazole H), 6.42 (s, 1H, aromatic H), 6.14 (dd, *J* = 17.1, 10.8 Hz, 1H, vinylic H), 5.60–5.51 (m, 1H, *N*CH), 4.93 (dd, *J* = 10.8, 1.2 Hz, 1H, vinylic H), 4.91 (dd, *J* = 17.1, 1.2 Hz, 1H, vinylic H), 3.90 (s, 3H, OCH_3_), 3.84 (s, 3H, OCH_3_), 1.90–1.63 (m, 4H, 2 × *CH_2_*), 1.44 (s, 6H, 2 × CH_3_), 0.80 (t, *J* = 7.5 Hz, 6H, 2 × CH_2_*CH_3_*). ^13^C NMR (75 MHz, CDCl_3_) *δ* 181.45, 161.70, 159.30, 147.95, 147.82, 144.88, 139.52, 129.07, 128.23, 128.02, 121.12, 115.69, 110.09, 95.95, 60.20, 55.92, 55.42, 40.13, 29.26, 27.63, 10.40. IR (film) *ν*_max_: 2965, 2935, 2877, 1654, 1584, 1500, 1459, 1291 cm^−1^. HRMS: *m/z* calculated for C_24_H_33_N_2_O_3_ [M + H]^+^: 397.2491. Found: 397.2495.

### 3.9. Cell Culture

All prostate cancer cell lines were obtained from the American Type Culture Collection (ATCC, Manassas, VA, USA). PC-3, LNCaP, and 22Rv1 cells were cultured in RPMI-1640 medium supplemented with 10% FBS and 1% penicillin/streptomycin. DU145 cells were cultured in Eagle’s Minimum Essential Medium (EMEM) with 10% FBS and 1% penicillin/streptomycin. All cultures were maintained at 37 °C in a humidified environment with 5% carbon dioxide.

### 3.10. WST-1 Cell Proliferation Assay

PC-3, DU145, and LNCaP cells were seeded in 96-well plates at a density of 3200 cells per well in 100 µL of culture medium. The 22Rv1 cells were seeded at a higher density of 6400 cells per well in 100 µL of culture medium. After 16 h of incubation, the cells were treated with enzalutamide or licochalcone A (as positive controls) or synthesized derivatives at five different concentrations for 3 days. An equal volume of DMSO (0.25% in medium) was used as the vehicle control. The cells were further incubated in a CO_2_ incubator at 37 °C for three days.

Following treatment, 10 µL of the WST-1 cell proliferation reagent (Cayman Chemical Company, Ann Arbor, MI, USA) was added to each well. The plates were gently mixed on an orbital shaker for 1 min, then incubated for an additional 3 h at 37 °C. Absorbance was measured at 430 nm using a microplate reader (Synergy HT, BioTek, Winooski, VT, USA). The IC_50_ value, defined as the concentration required to inhibit cell proliferation by 50%, was calculated based on at least five different doses of each compound. Each IC_50_ value represents the average from triplicate experiments, which were statistically significant and reproducible.

### 3.11. Statistical Analysis

Data are expressed as mean ± standard derivation from the specified number of experiments. Statistical differences between treatment and control groups were assessed using Student’s *t*-test during the data analysis phase, with significance set at *p* < 0.05.

## 4. Conclusions

As part of our ongoing research to search for new anti-prostate cancer agents, this study chose naturally occurring licochalcone A as a lead compound. By drawing from our previous success in modifying curcumin, we synthesized three series derivatives through a targeted synthetic strategy involving [3,3]-sigmatropic rearrangement and Claisen–Schmidt condensation. Systematic evaluation of these derivatives revealed key trends in their structure–-activity relationships:

- Chalcones Series **3a**–**3i**: Among this series, six chalcone derivatives (**3d**, **3e, 3f**, **3g**, **3h**, and **3i**) demonstrated antiproliferative activity similar to licochalcone A in androgen receptor-positive LNCaP and 22Rv1 cells. However, these compounds exhibited minimal activity in androgen receptor-negative PC-3 and DU145 cells, even at concentrations up to 100 µM. This suggests that modifications to the chalcone scaffold may enhance specificity toward androgen receptor-positive cells, likely by improving interactions with the androgen receptor or related signaling pathways.
- Ester Series **8**–**10:** The ester derivatives (**8**–**10**) demonstrated similar potency to licochalcone A in both androgen receptor-positive and androgen receptor-negative prostate cancer cell lines, suggesting that this modification does not significantly alter the compound’s broad-spectrum efficacy. These results highlight the phenol moiety adjacent to the carbonyl group in licochalcone A as a promising site for chemical modification.
- Chalcone Series **13a**–**13g:** The antiproliferative potency of this series confirmed that the phenol moiety adjacent to the carbonyl group in licochalcone A can be replaced by the substituted imidazole moiety. The potency of these derivatives was highly dependent on the structure of the R group on the imidazole ring. For example, compounds **13a** and **13g**, with methyl and isopentyl groups, respectively, exhibited IC_50_ values above 100 µM across all four tested prostate cancer cell lines, indicating reduced activity. In contrast, seven other derivatives in this series exhibited strong antiproliferative activity in AR-positive LNCaP and 22Rv1 cells, comparable to or greater than licochalcone A and enzalutamide. Notably, derivatives **13b**, **13f**, and **13i** showed significant selectivity, suppressing AR-positive cell proliferation while exhibiting less effect on androgen receptor-negative PC-3 and DU145 cells. These findings underscore the critical role of the R groups on the imidazole ring in optimizing both the antiproliferative potency and selectivity of these compounds.

The observed selectivity and potency of certain chalcone derivatives highlight the potential for developing more effective anti-prostate cancer agents by optimizing the structure of licochalcone A. Future work will focus on refining these derivatives and investigating their in vivo efficacy, paving the way for novel therapeutic options to overcome the resistance to current treatments. Additionally, the observed selective antiproliferative activity of the licochalcone A analogs in AR-positive versus AR-negative cells suggests that the androgen receptor may be a potential target. However, future investigation is needed to confirm this hypothesis, particularly through techniques such as Western blotting and surface plasma resonance.

## Figures and Tables

**Table 1:** Antiproliferative activities of chalcones **3a**–**3i** against prostate cancer cell lines.

Compounds	IC_50_ (µM) ^1^			
	PC-3 ^2^	DU145 ^2^	LNCaP ^3^	22Rv1 ^3^
**3a**	>100	>100	>100	>100
**3b**	27.56 ± 1.21	27.59 ± 2.21	13.82 ± 0.64	24.57 ± 2.18
**3c**	>100	>100	27.04 ± 1.81	>100
**3d**	>100	>100	21.52 ± 3.99	20.14 ± 0.57
**3e**	>100	>100	25.48 ± 3.69	36.77 ± 2.72
**3f**	>100	>100	21.26 ± 2.94	21.68 ± 2.48
**3g**	>100	>100	41.88 ± 4.37	33.97 ± 1.20
**3h**	>100	>100	23.99 ± 2.01	22.21 ± 0.46
**3i**	>100	>100	13.74 ± 2.35	21.70 ± 0.74
Enzalutamide	>100	36.43 ± 1.34	21.75 ± 4.37	67.54 ± 1.41
Licochalcone A	15.73 ± 1.32	23.35 ± 1.41	22.04 ± 2.28	17.97 ± 2.23

^1^ IC_50_ represents the half-maximal inhibitory concentration, determined using the WST-1 cell proliferation assay after a 3-day exposure. The data are presented as mean ± standard deviation. ^2^ Human AR-negative prostate cancer cell line. ^3^ Human AR-positive prostate cancer cell line.

**Table 2:** Antiproliferative activities of esters **8**–**10** against prostate cancer cell lines.

Compounds	IC_50_ (µM) ^1^			
	PC-3 ^2^	DU145 ^2^	LNCaP ^3^	22Rv1 ^3^
**8**	23.33 ± 1.78	26.19 ± 1.14	17.97 ± 1.11	24.09 ± 2.23
**9**	20.89 ± 0.61	25.06 ± 1.37	12.77 ± 1.40	21.11 ± 0.90
**10**	21.85 ± 1.38	20.57 ± 1.86	12.83 ± 1.19	21.88 ± 1.83
Enzalutamide	>100	36.43 ± 1.34	21.75 ± 4.37	67.54 ± 1.41
Licochalcone A	15.73 ± 1.32	23.35 ± 1.41	22.04 ± 2.28	17.97 ± 2.23

^1^ IC_50_ represents the half-maximal inhibitory concentration, determined using the WST-1 cell proliferation assay after a 3-day exposure. The data are presented as mean ± standard deviation. ^2^ Human AR-negative prostate cancer cell line. ^3^ Human AR-positive prostate cancer cell line.

**Table 3:** Antiproliferative activities of chalcones **13a**–**13i** against prostate cancer cell lines.

Compounds	IC_50_ (µM) ^1^			
	PC-3 ^2^	DU145 ^2^	LNCaP ^3^	22Rv1 ^3^
**13a**	>100	>100	>100	>100
**13b**	>100	>100	28.13 ± 3.24	14.78 ± 0.57
**13c**	43.86 ± 1.49	33.87 ± 3.07	20.51 ± 2.41	39.46 ± 1.69
**13d**	52.12 ± 2.88	47.03 ± 4.15	18.33 ± 2.69	52.19 ± 2.90
**13e**	54.05 ± 6.00	54.72 ± 2.30	18.90 ± 0.17	31.27 ± 2.50
**13f**	>100	>100	18.60 ± 1.71	33.25 ± 1.44
**13g**	>100	>100	>100	>100
**13h**	47.05 ± 7.46	86.24 ± 1.42	9.76 ± 1.61	28.85 ± 1.87
**13i**	>100	74.03 ± 0.59	9.43 ± 2.25	28.42 ± 1.91
Enzalutamide	>100	36.43 ± 1.34	21.75 ± 4.37	67.54 ± 1.41
Licochalcone A	15.73 ± 1.32	23.35 ± 1.41	22.04 ± 2.28	17.97 ± 2.23

^1^ IC_50_ represents the half-maximal inhibitory concentration, determined using the WST-1 cell proliferation assay after a 3-day exposure. The data are presented as mean ± standard deviation. ^2^ Human AR-negative prostate cancer cell line. ^3^ Human AR-positive prostate cancer cell line.

## Data Availability

All critical data have been included in the Supplementary Materials.

## Supplementary Materials

The NMR spectra and high-resolution mass spectra can be downloaded at: https://www.mdpi.com/article/10.3390/molecules29246023/s1.
