# Supplementary material for: Licochalcone A-Inspired Chalcones: Synthesis and Their Antiproliferative Potential in Prostate Cancer Cells
Source: Molecules. 2024 Dec 20;29(24):6023. doi: 10.3390/molecules29246023 (PMC11679503; doi:10.3390/molecules29246023)
Supplement: Supplementary file 1 [file molecules-29-06023-s001.zip › molecules-3316805-supplementary.pdf]

Supplementary Materials

Licochalcone A-Inspired Chalcones: Synthesis and Their Antiproliferative Potential in Prostate Cancer Cells

Roxana Gonzalez Dorado [1], Esveidy Isabel Oceguera Nava [1], Guanglin Chen [1], Qiang Zhang [2], Guangdi Wang [2], and Qiao-Hong Chen [1,*]

[1] Department of Chemistry & Biochemistry, California State University, Fresno, CA 93740, USA

[2] Department of Chemistry and RCMI Cancer Research Center, Xavier University of Louisiana, New Orleans, LA 70125, USA

[*] *Correspondence: qchen@csufresno.edu*

List of Contents:

Figure S1: ^{1}H NMR spectrum of **7a** in $CDCl_3$……S4
Figure S2: ^{13}C NMR spectrum of **7a** in $CDCl_3$……S5
Figure S3: ^{1}H NMR spectrum of **7b** in $CDCl_3$……S6
Figure S4: ^{13}C NMR spectrum of **7b** in $CDCl_3$……S7
Figure S5: ^{1}H NMR spectrum of **7c** in $CDCl_3$……S8
Figure S6: ^{13}C NMR spectrum of **7c** in $CDCl_3$……S9
Figure S7: ^{1}H NMR spectrum of **7d** in $CDCl_3$……S10
Figure S8: ^{13}C NMR spectrum of **7d** in $CDCl_3$……S11
Figure S9: ^{1}H NMR spectrum of **7e** in $CDCl_3$……S12
Figure S10: ^{13}C NMR spectrum of **7e** in $CDCl_3$……S13
Figure S11: ^{1}H NMR spectrum of **7f** in $CDCl_3$……S14
Figure S12: ^{13}C NMR spectrum of **7f** in $CDCl_3$……S15
Figure S13: ^{1}H NMR spectrum of **7g** in $CDCl_3$……S16
Figure S14: ^{13}C NMR spectrum of **7g** in $CDCl_3$……S17
Figure S15: ^{1}H NMR spectrum of **7h** in $CDCl_3$……S18
Figure S16: ^{13}C NMR spectrum of **7h** in $CDCl_3$……S19
Figure S17: ^{1}H NMR spectrum of **7i** in $CDCl_3$……S20
Figure S18: ^{13}C NMR spectrum of **7i** in $CDCl_3$……S21
Figure S19: ^{1}H NMR spectrum of **5a** in $CDCl_3$……S22
Figure S20: ^{13}C NMR spectrum of **5a** in $CDCl_3$……S23
Figure S21: ^{1}H NMR spectrum of **5b** in $CDCl_3$……S24
Figure S22: ^{13}C NMR spectrum of **5b** in $CDCl_3$……S25
Figure S23: ^{1}H NMR spectrum of **5c** in $CDCl_3$……S26
Figure S24: ^{13}C NMR spectrum of **5c** in $CDCl_3$……S27
Figure S25: ^{1}H NMR spectrum of **5d** in $CDCl_3$……S28
Figure S26: ^{13}C NMR spectrum of **5d** in $CDCl_3$……S29
Figure S27: ^{1}H NMR spectrum of **5e** in $CDCl_3$……S30

Figure S28: ^{13}C NMR spectrum of **5e** in $CDCl_3$……S31
Figure S29: ^{1}H NMR spectrum of **5f** in $CDCl_3$……S32
Figure S30: ^{13}C NMR spectrum of **5f** in $CDCl_3$……S33
Figure S31: ^{1}H NMR spectrum of **5g** in $CDCl_3$……S34
Figure S32: ^{13}C NMR spectrum of **5g** in $CDCl_3$……S35
Figure S33: ^{1}H NMR spectrum of **5h** in $CDCl_3$……S36
Figure S34: ^{13}C NMR spectrum of **5h** in $CDCl_3$……S37
Figure S35: ^{1}H NMR spectrum of **5i** in $CDCl_3$……S38
Figure S36: ^{13}C NMR spectrum of **5i** in $CDCl_3$……S39
Figure S37: ^{1}H NMR spectrum of **3a** in $CDCl_3$……S40
Figure S38: ^{13}C NMR spectrum of **3a** in $CDCl_3$……S41
Figure S39: High resolution mass spectrum of **3a**……S42
Figure S40: ^{1}H NMR spectrum of **3b** in $CDCl_3$……S43
Figure S41: ^{13}C NMR spectrum of **3b** in $CDCl_3$……S44
Figure S42: High resolution mass spectrum of **3b**……S45
Figure S43: ^{1}H NMR spectrum of **3c** in $CDCl_3$……S46
Figure S44: ^{13}C NMR spectrum of **3c** in $CDCl_3$……S47
Figure S45: High resolution mass spectrum of **3c**……S48
Figure S46: ^{1}H NMR spectrum of **3d** in $CDCl_3$……S49
Figure S47: ^{13}C NMR spectrum of **3d** in $CDCl_3$……S50
Figure S48: High resolution mass spectrum of **3d**……S51
Figure S49: ^{1}H NMR spectrum of **3e** in $CDCl_3$……S52
Figure S50: High resolution mass spectrum of **3e**……S53
Figure S51: ^{1}H NMR spectrum of **3f** in $CDCl_3$……S54
Figure S52: ^{13}C NMR spectrum of **3f** in $CDCl_3$……S55
Figure S53: High resolution mass spectrum of **3f**……S56
Figure S54: ^{1}H NMR spectrum of **3g** in $CDCl_3$……S57
Figure S55: ^{13}C NMR spectrum of **3g** in $CDCl_3$……S58
Figure S56: High resolution mass spectrum of **3g**……S59
Figure S57: ^{1}H NMR spectrum of **3h** in $CDCl_3$……S60
Figure S58: ^{13}C NMR spectrum of **3h** in $CDCl_3$……S61
Figure S59: High resolution mass spectrum of **3h**……S62
Figure S60: ^{1}H NMR spectrum of **3i** in $CDCl_3$……S63
Figure S61: ^{13}C NMR spectrum of **3i** in $CDCl_3$……S64
Figure S62: High resolution mass spectrum of **3i**……S65
Figure S63: ^{1}H NMR spectrum of **8** in $CDCl_3$……S66
Figure S64: ^{13}C NMR spectrum of **8** in $CDCl_3$……S67
Figure S65: High resolution mass spectrum of **8**……S68
Figure S66: ^{1}H NMR spectrum of **9** in $CDCl_3$……S69
Figure S67: ^{13}C NMR spectrum of **9** in $CDCl_3$……S70

Figure S68: High resolution mass spectrum of **9**……...S71
Figure S69: ^{1}H NMR spectrum of **10** in $CDCl_3$……S72
Figure S70: ^{13}C NMR spectrum of **10** in $CDCl_3$……...S73
Figure S71: High resolution mass spectrum of **10**……...S74
Figure S72: ^{1}H NMR spectrum of **12** in $CDCl_3$……...S75
Figure S73: ^{13}C NMR spectrum of **12** in $CDCl_3$……....S76
Figure S74: ^{1}H NMR spectrum of **13a** in $CDCl_3$……...S77
Figure S75: ^{13}C NMR spectrum of **13a** in $CDCl_3$……...S78
Figure S76: High resolution mass spectrum of **13a**……...S79
Figure S77: ^{1}H NMR spectrum of **13b** in $CDCl_3$……..S80
Figure S78: ^{13}C NMR spectrum of **13b** in $CDCl_3$……..S81
Figure S79: High resolution mass spectrum of **13b**……...S82
Figure S80: ^{1}H NMR spectrum of **13c** in $CDCl_3$……...S83
Figure S81: ^{13}C NMR spectrum of **13c** in $CDCl_3$……...S84
Figure S82: High resolution mass spectrum of **13c**……........S85
Figure S83: ^{1}H NMR spectrum of **13d** in $CDCl_3$……...S86
Figure S84: ^{13}C NMR spectrum of **13d** in $CDCl_3$……......S87
Figure S85: High resolution mass spectrum of **13d**……...S88
Figure S86: ^{1}H NMR spectrum of **13e** in $CDCl_3$……...S89
Figure S87: ^{13}C NMR spectrum of **13e** in $CDCl_3$……...S90
Figure S88: High resolution mass spectrum of **13e**……....S91
Figure S89: ^{1}H NMR spectrum of **13f** in $CDCl_3$……...S92
Figure S90: ^{13}C NMR spectrum of **13f** in $CDCl_3$……...S93
Figure S91: High resolution mass spectrum of **13f**……...S94
Figure S92: ^{1}H NMR spectrum of **13g** in $CDCl_3$……...S95
Figure S93: ^{13}C NMR spectrum of **13g** in $CDCl_3$……...S96
Figure S94: High resolution mass spectrum of **13g**……...S97
Figure S95: ^{1}H NMR spectrum of **13h** in $CDCl_3$……...S98
Figure S96: ^{13}C NMR spectrum of **13h** in $CDCl_3$……...S99
Figure S97: High resolution mass spectrum of **13h**……...S100
Figure S98: ^{1}H NMR spectrum of **13i** in $CDCl_3$……....S101
Figure S99: ^{13}C NMR spectrum of **13i** in $CDCl_3$……...S102
Figure S100: High resolution mass spectrum of **13i**……...S103

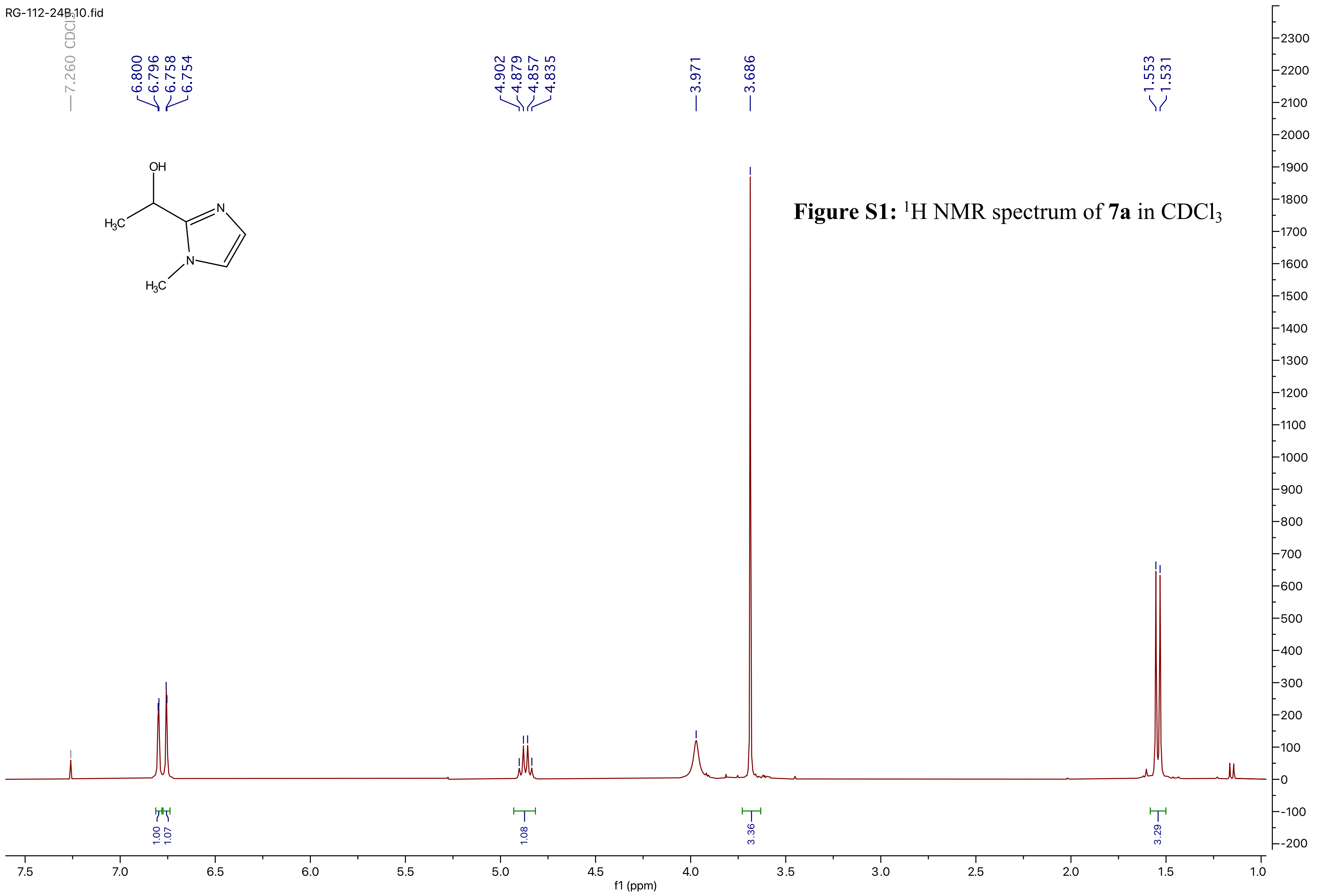

Figure S1: ^{1}H NMR spectrum of **7a** in $CDCl_3$

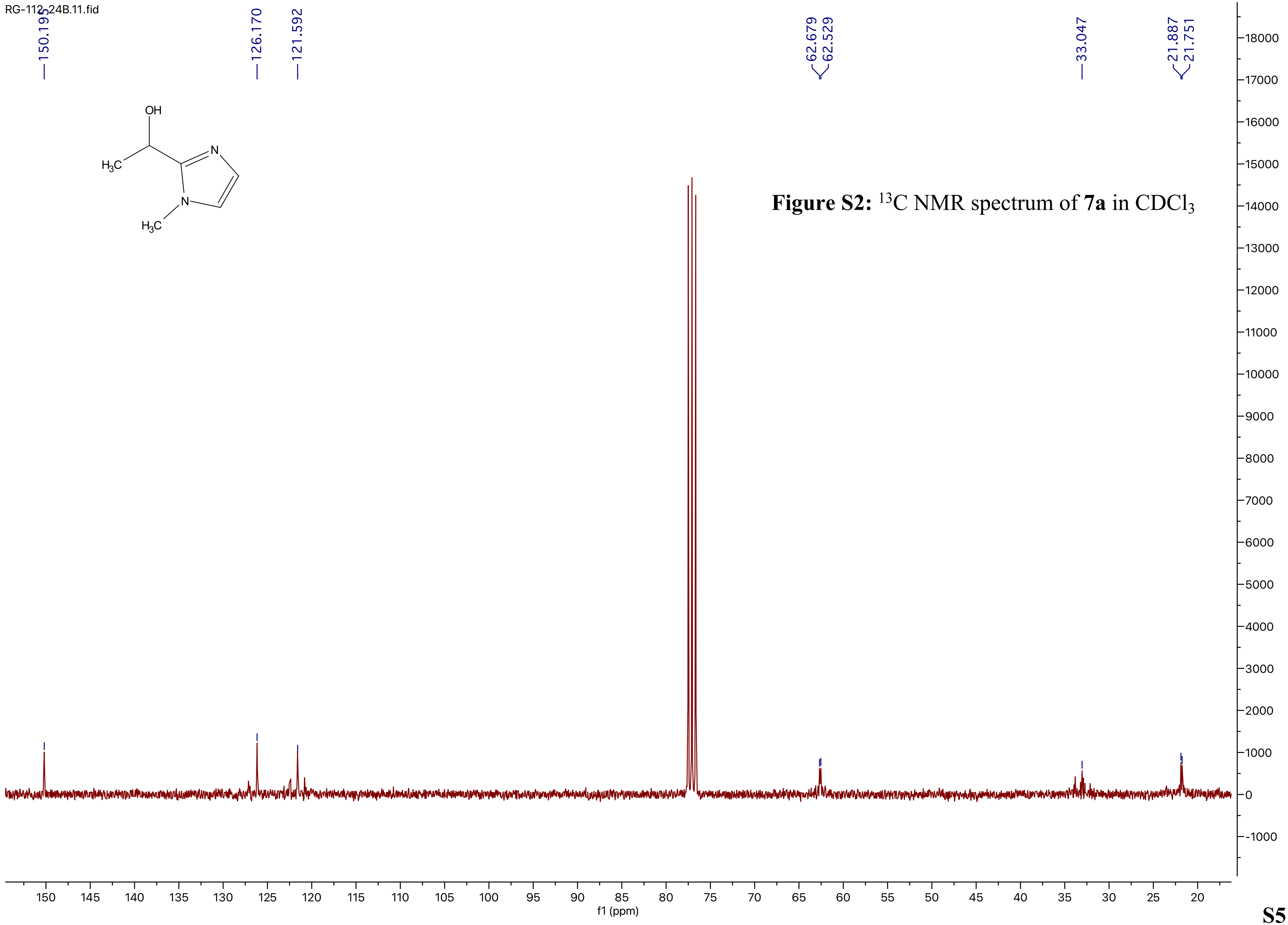

Figure S2: ^{13}C NMR spectrum of **7a** in $CDCl_3$

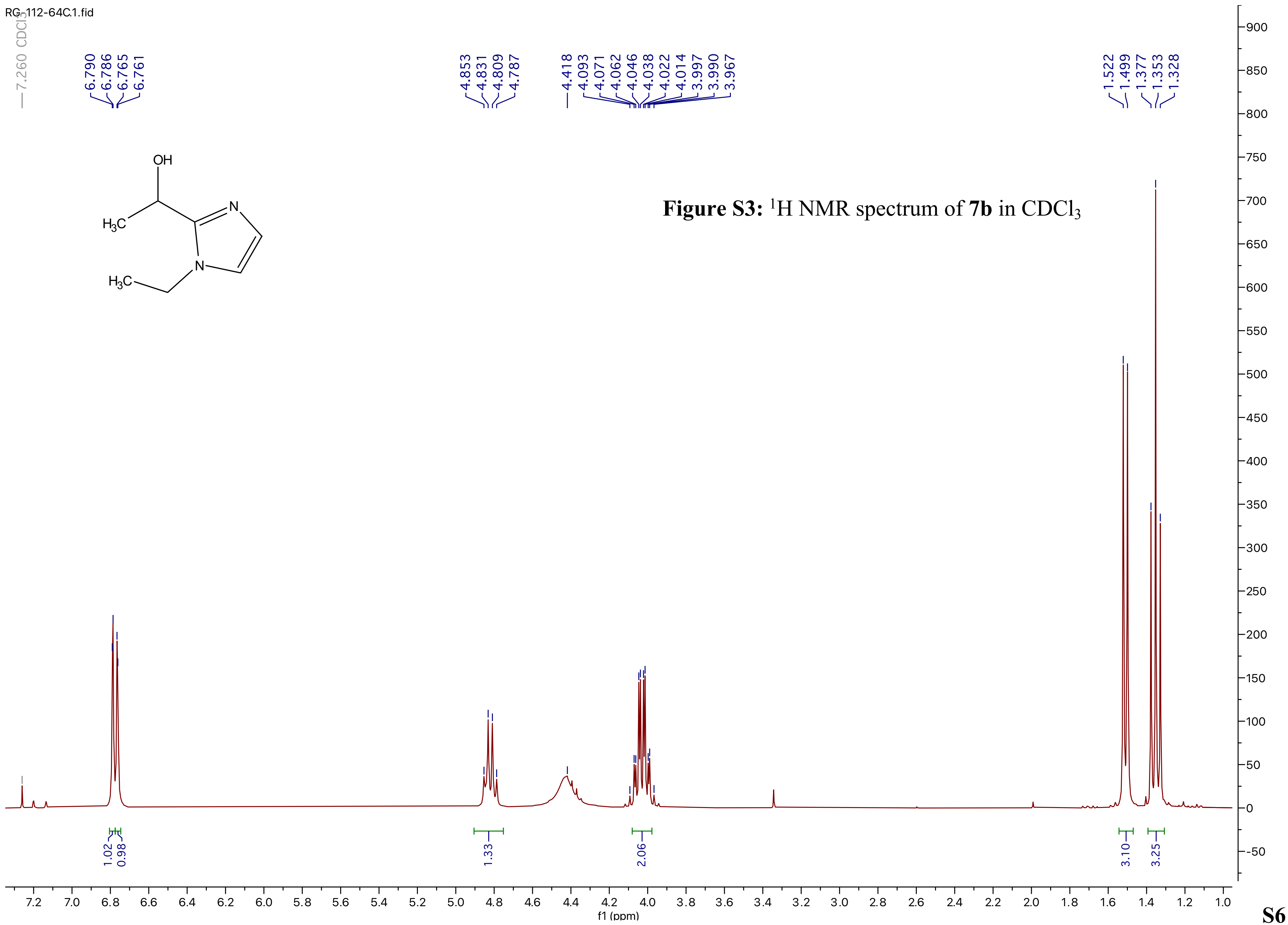

Figure S3: ^{1}H NMR spectrum of **7b** in $CDCl_3$

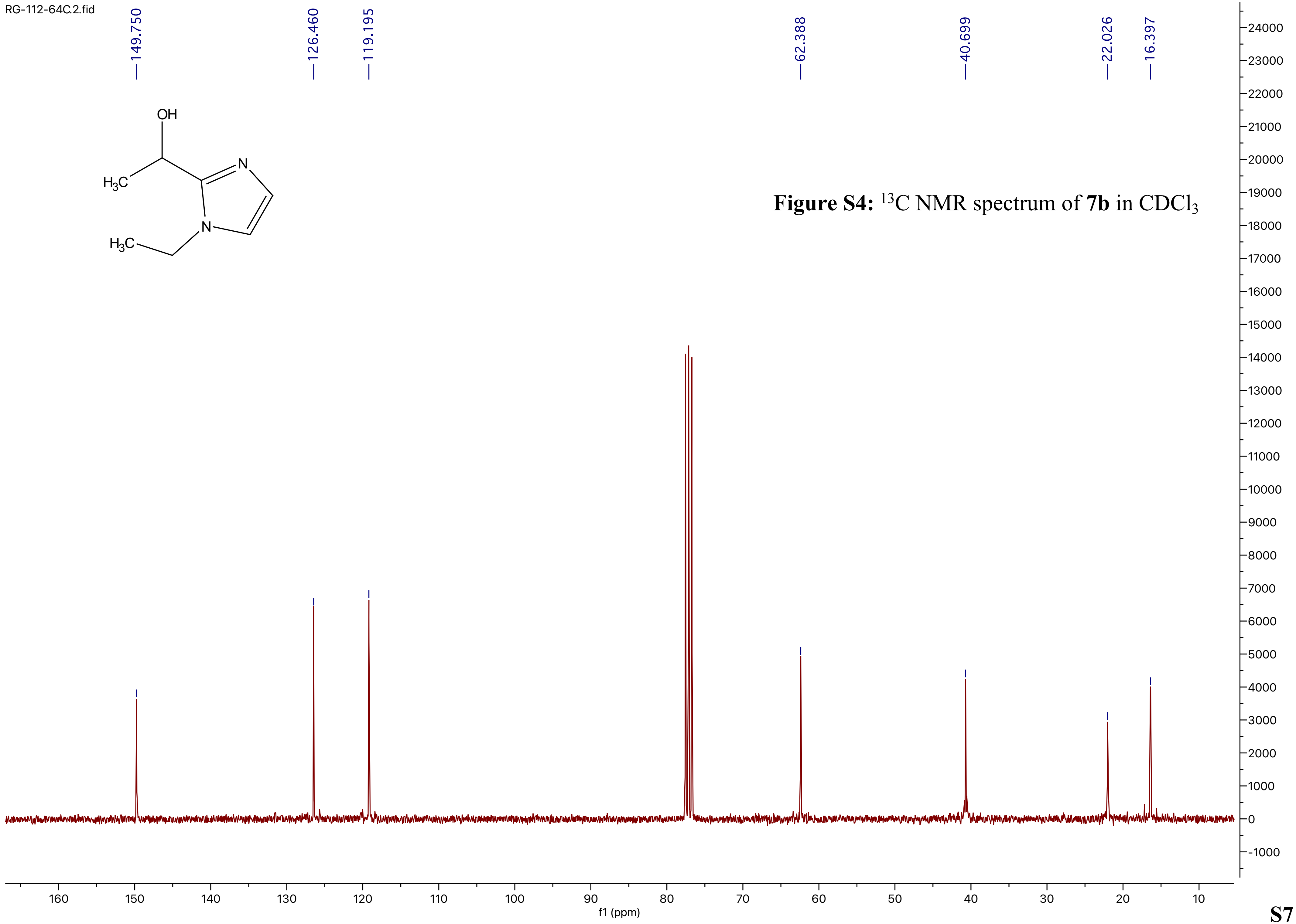

Figure S4: ^{13}C NMR spectrum of **7b** in $CDCl_3$

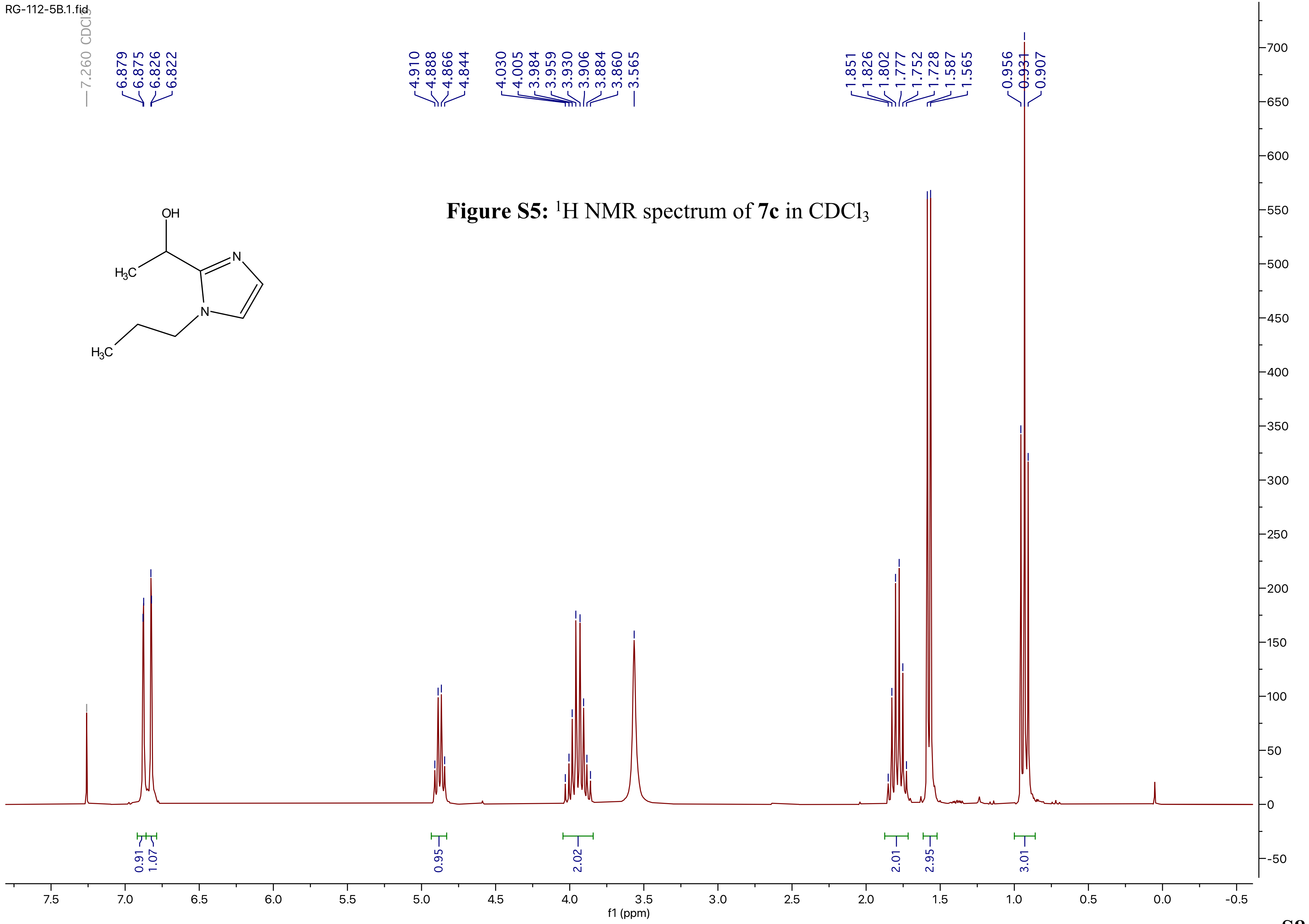

Figure S5: ^{1}H NMR spectrum of **7c** in $CDCl_3$

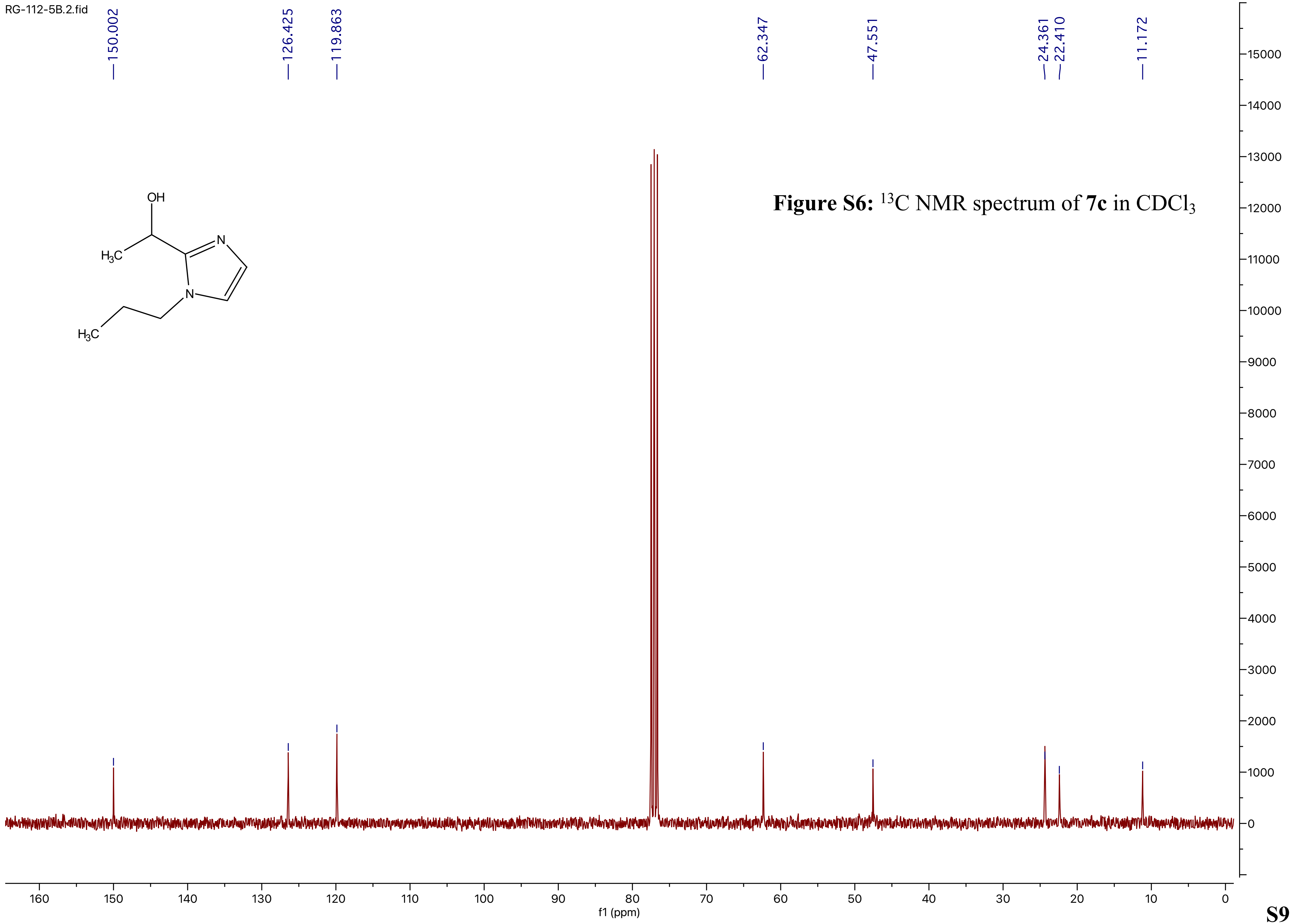

Figure S6: ^{13}C NMR spectrum of **7c** in $CDCl_3$

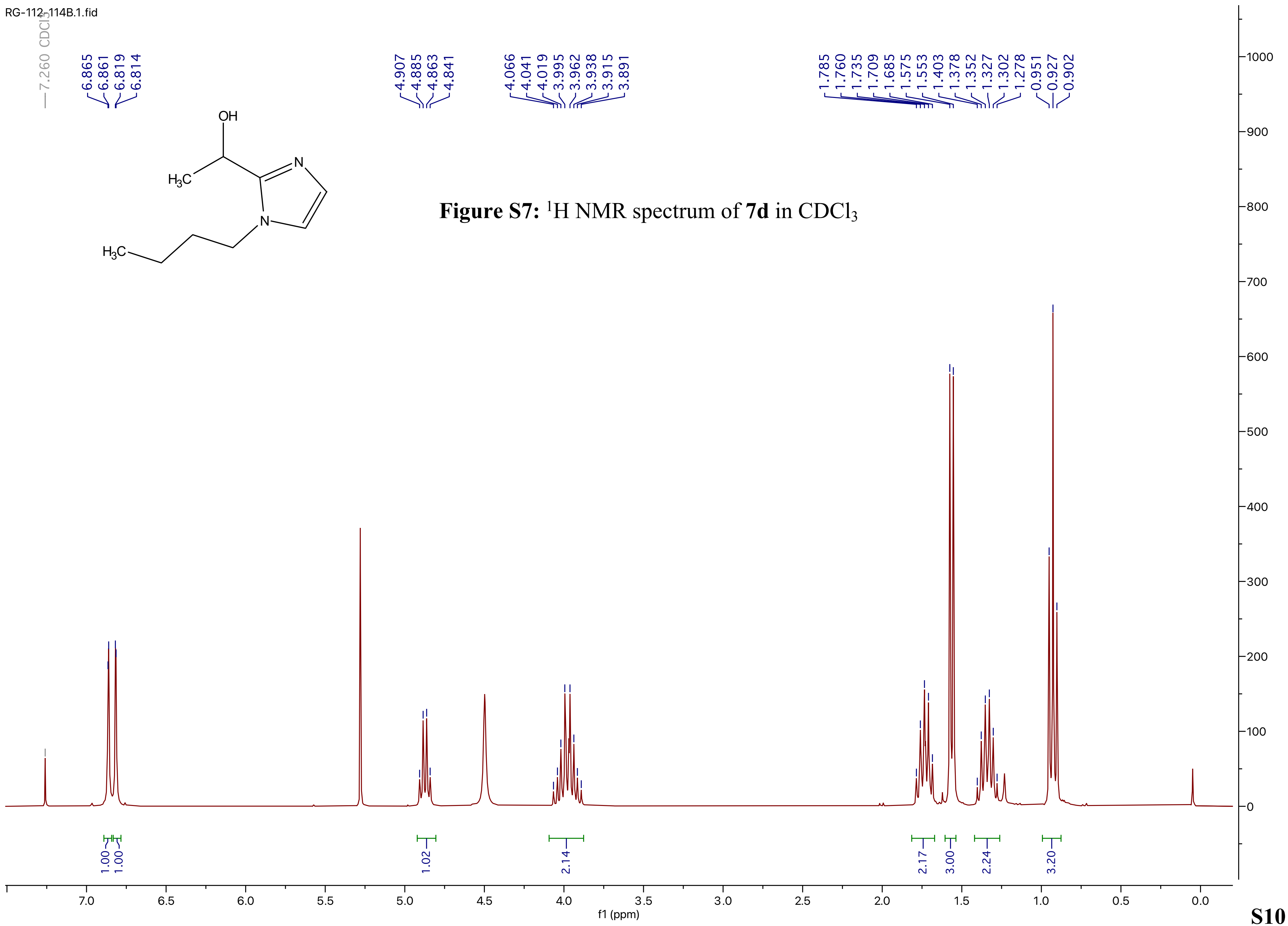

Figure S7: ^{1}H NMR spectrum of **7d** in $CDCl_3$

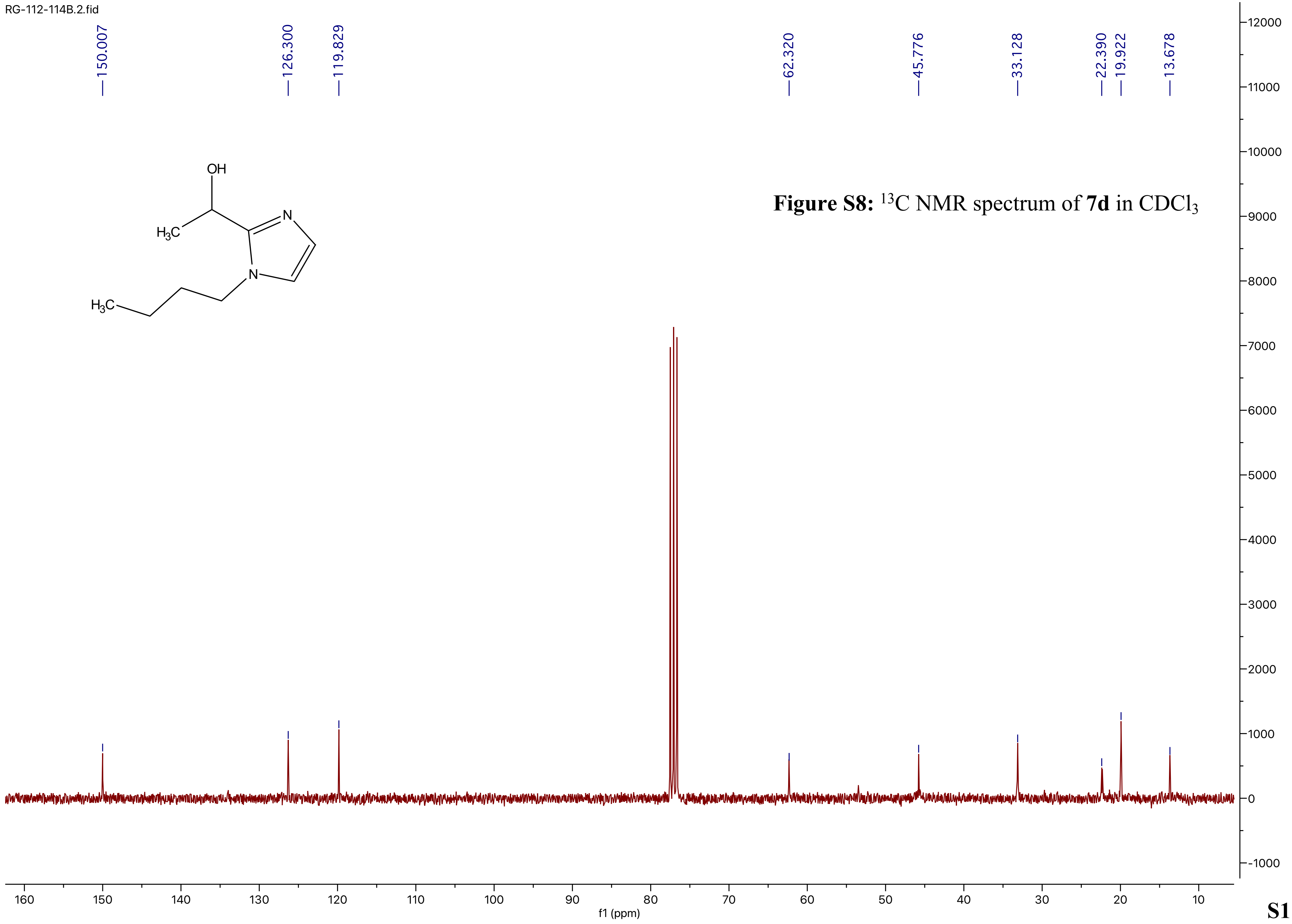

Figure S8: ^{13}C NMR spectrum of **7d** in $CDCl_3$

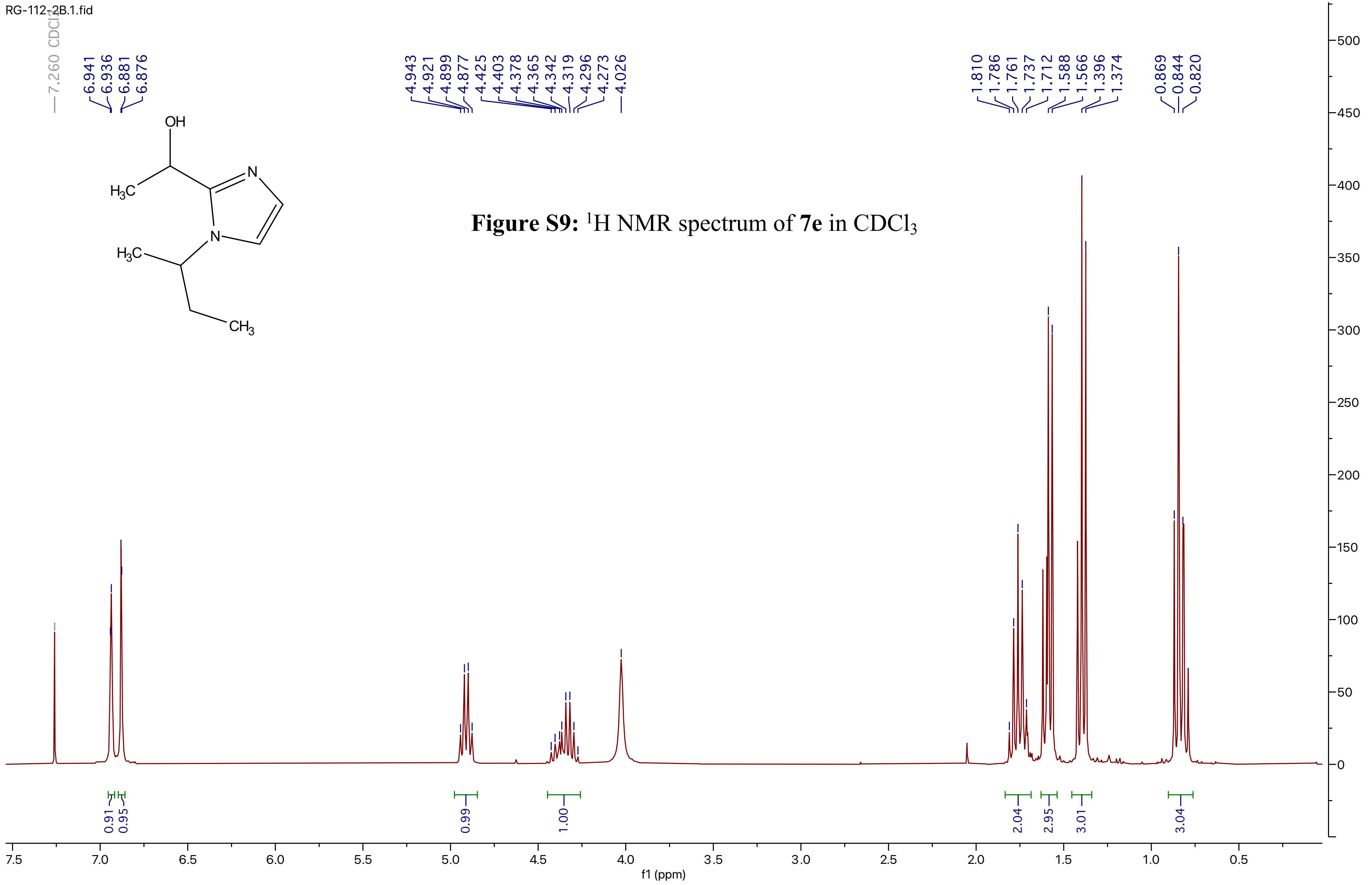

Figure S9: ^{1}H NMR spectrum of **7e** in $CDCl_3$

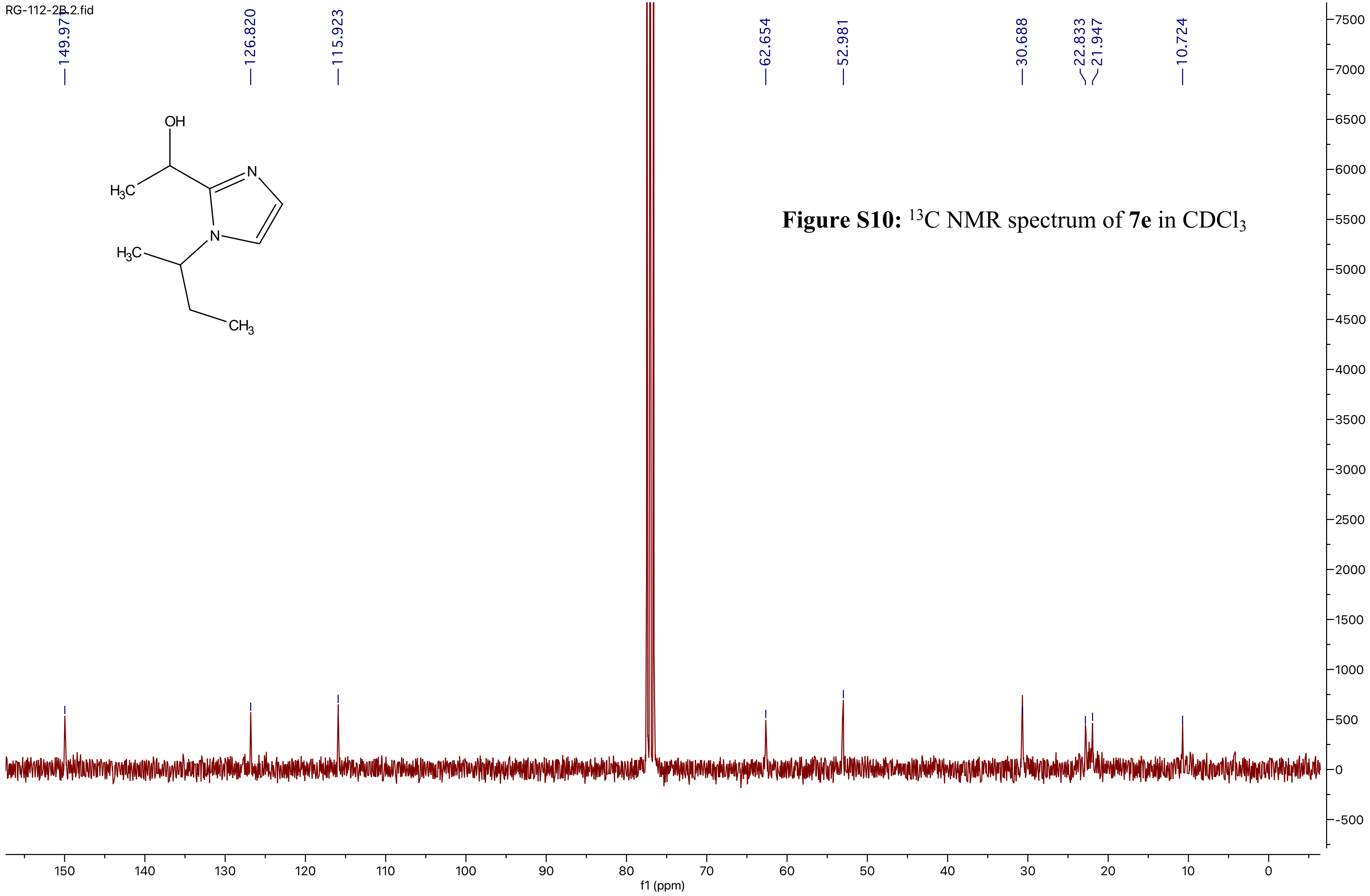

Figure S10: ^{13}C NMR spectrum of **7e** in $CDCl_3$

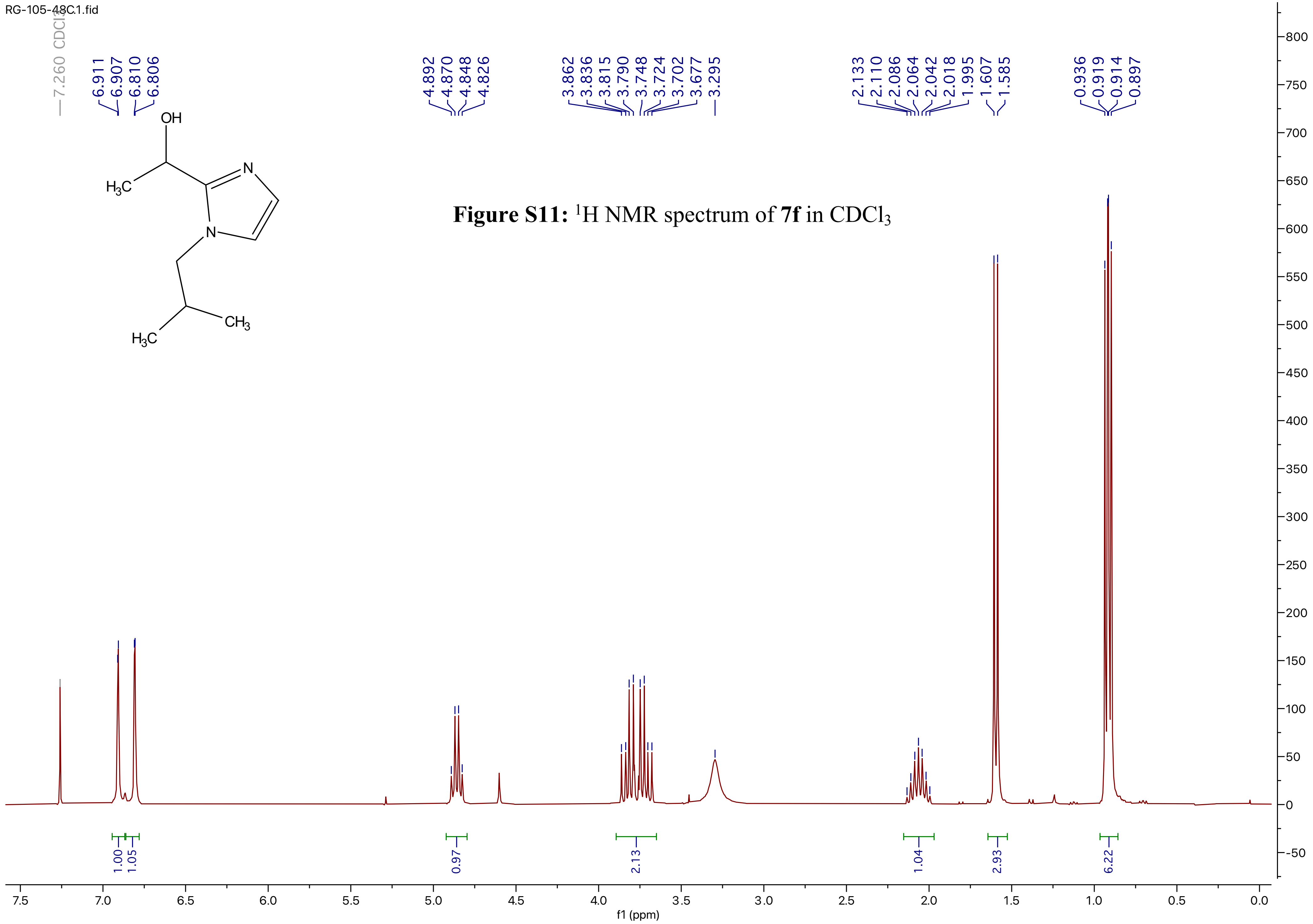

Figure S11: ^{1}H NMR spectrum of **7f** in $CDCl_3$

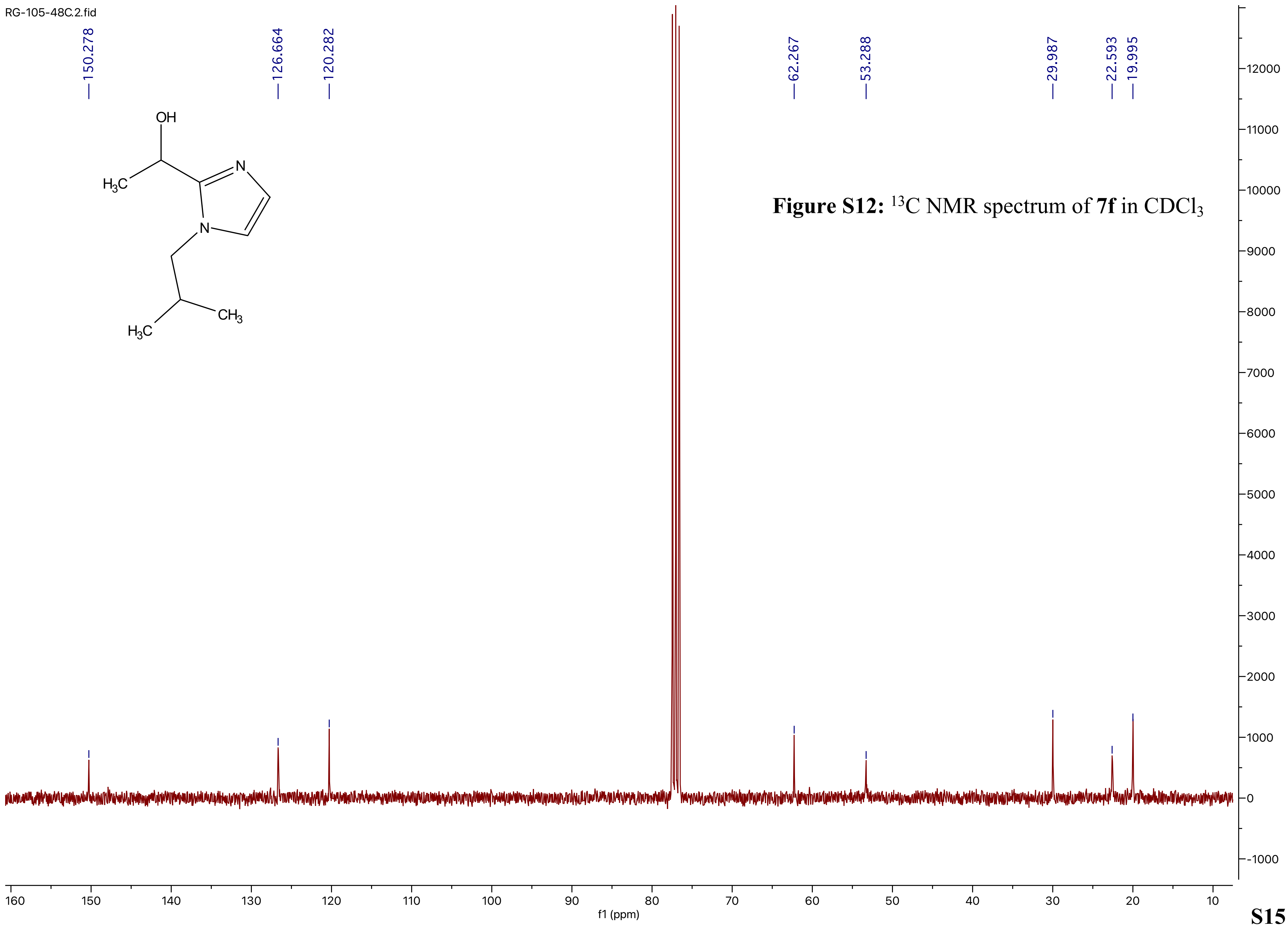

Figure S12: ^{13}C NMR spectrum of **7f** in $CDCl_3$

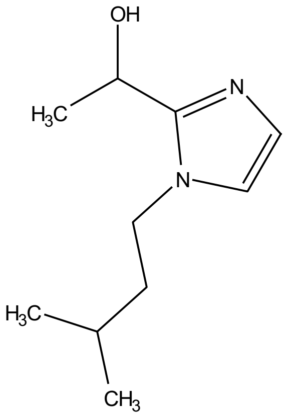

Figure S13: ^{1}H NMR spectrum of **7g** in $CDCl_3$

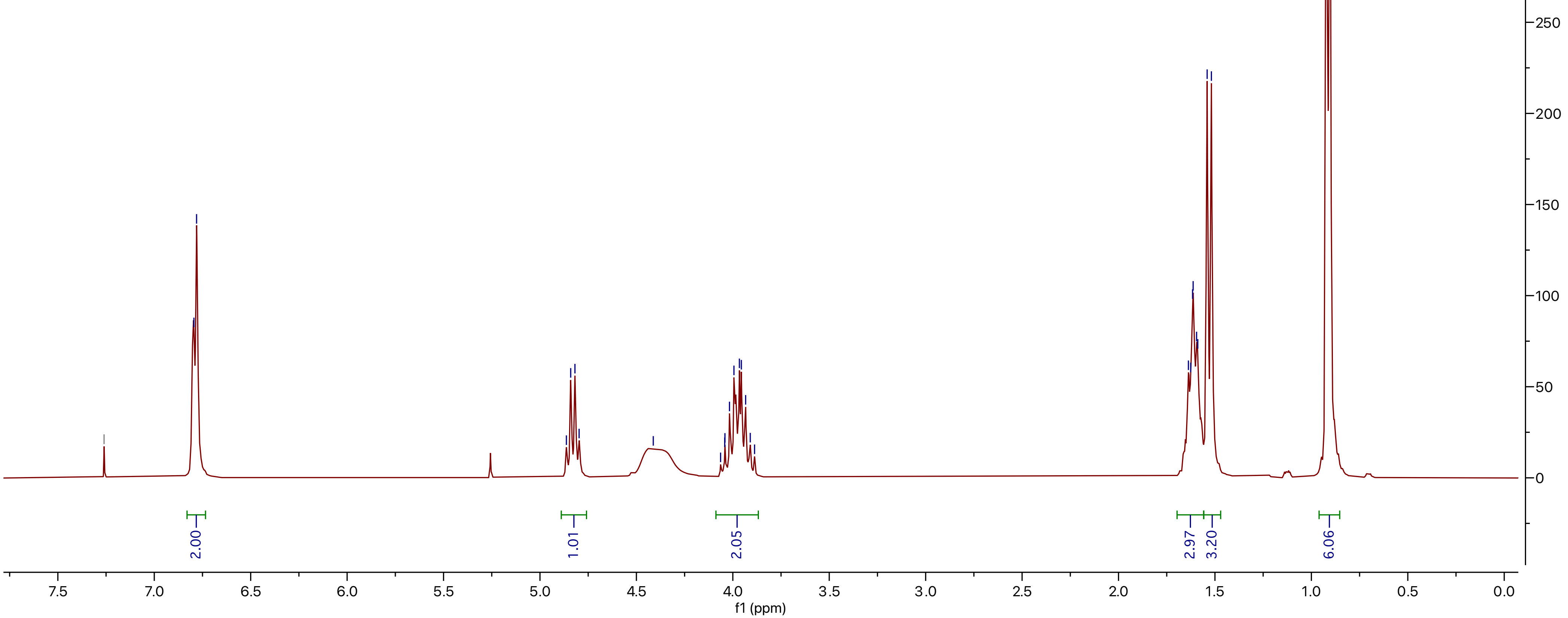

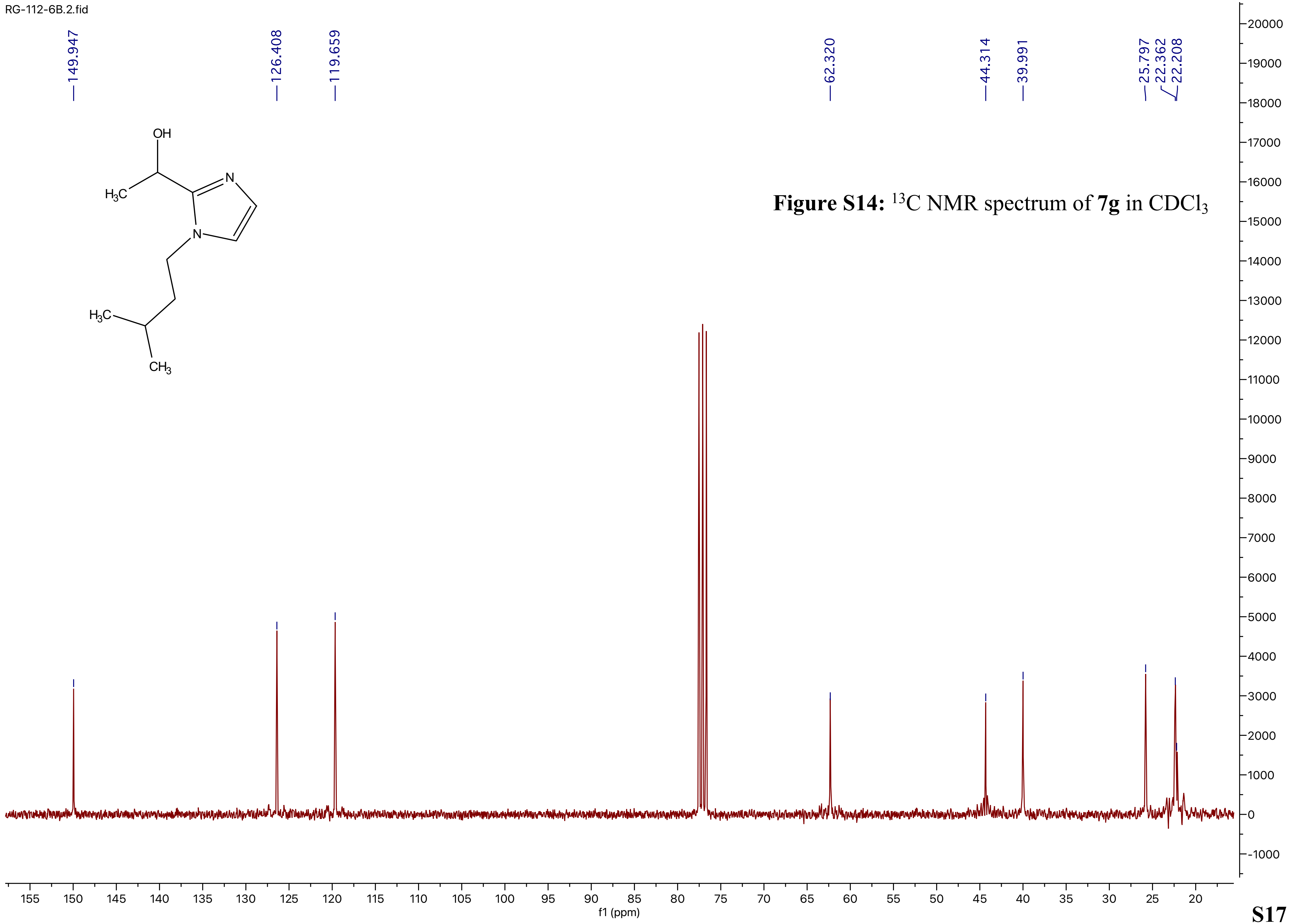

Figure S14: ^{13}C NMR spectrum of **7g** in $CDCl_3$

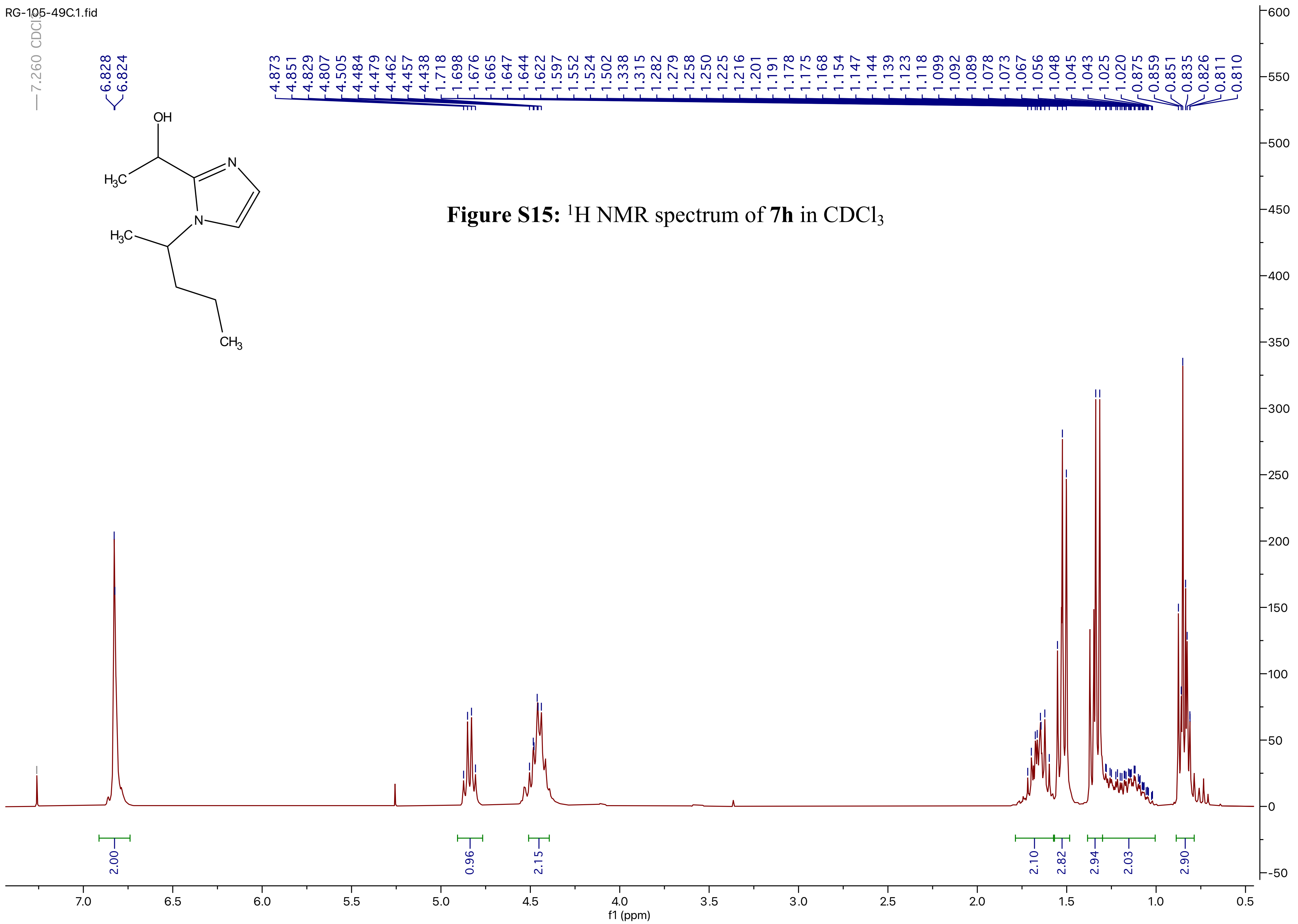

Figure S15: ^{1}H NMR spectrum of **7h** in $CDCl_3$

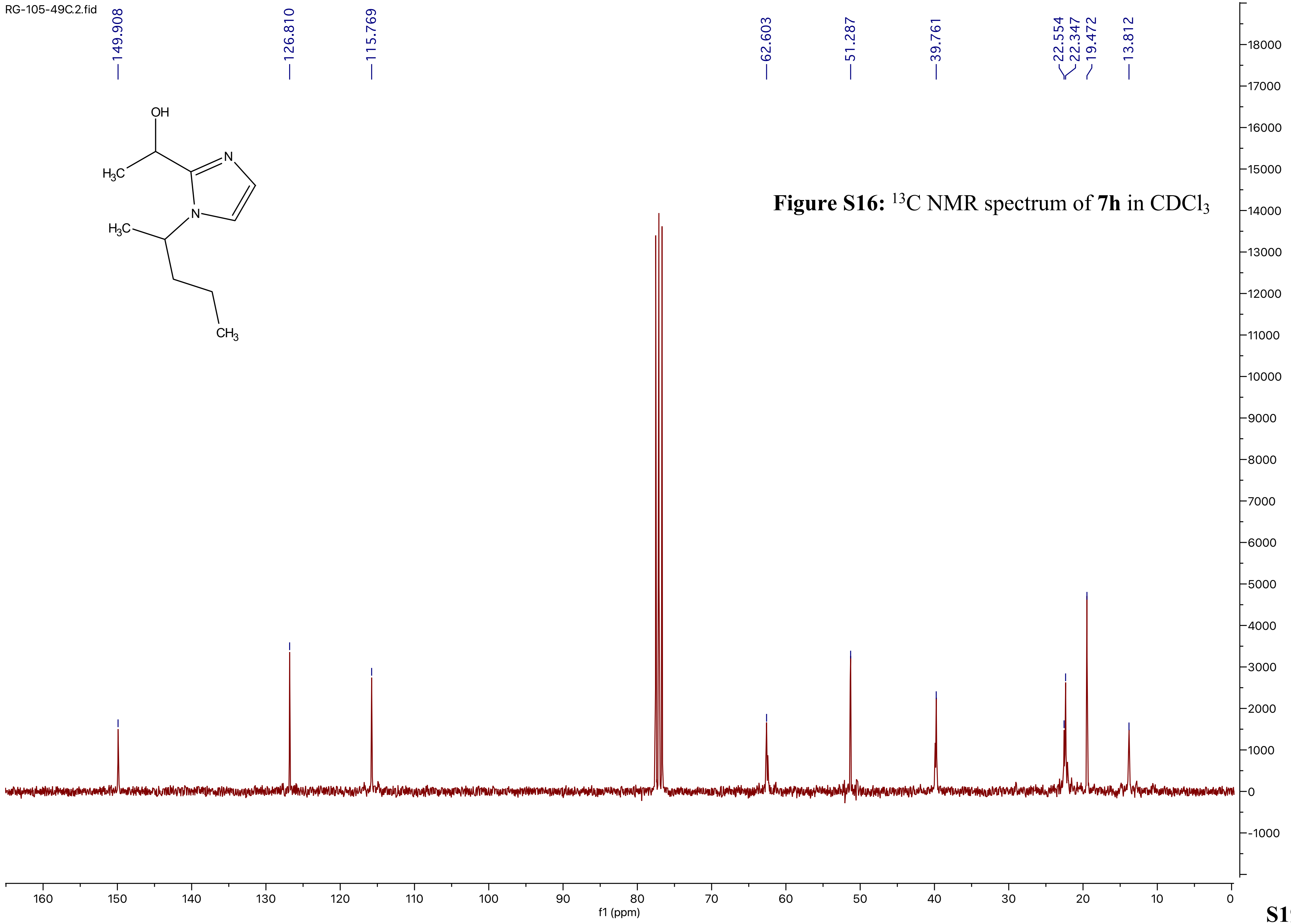

Figure S16: ^{13}C NMR spectrum of **7h** in $CDCl_3$

Figure S17: ^{1}H NMR spectrum of **7i** in $CDCl_3$

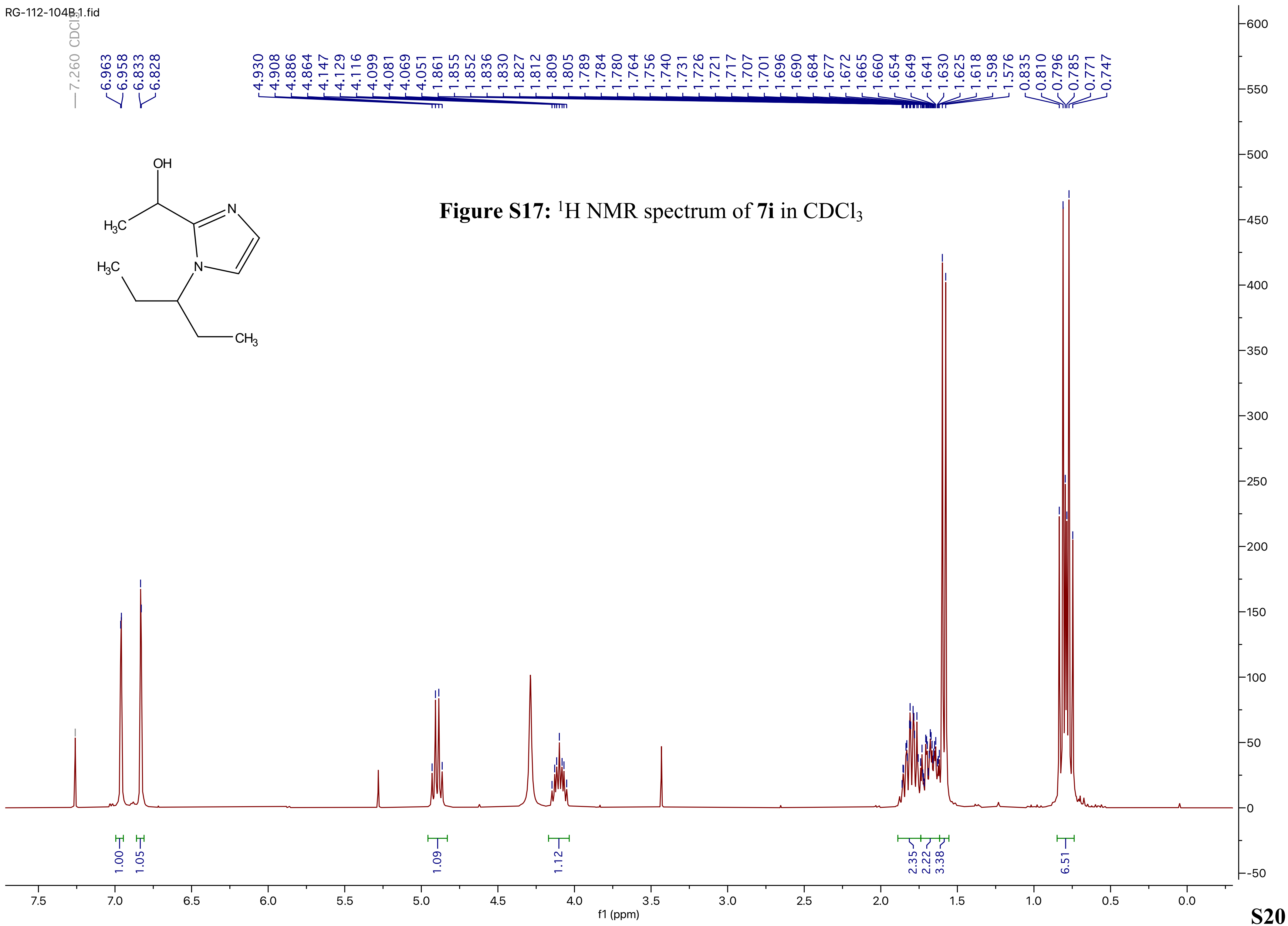

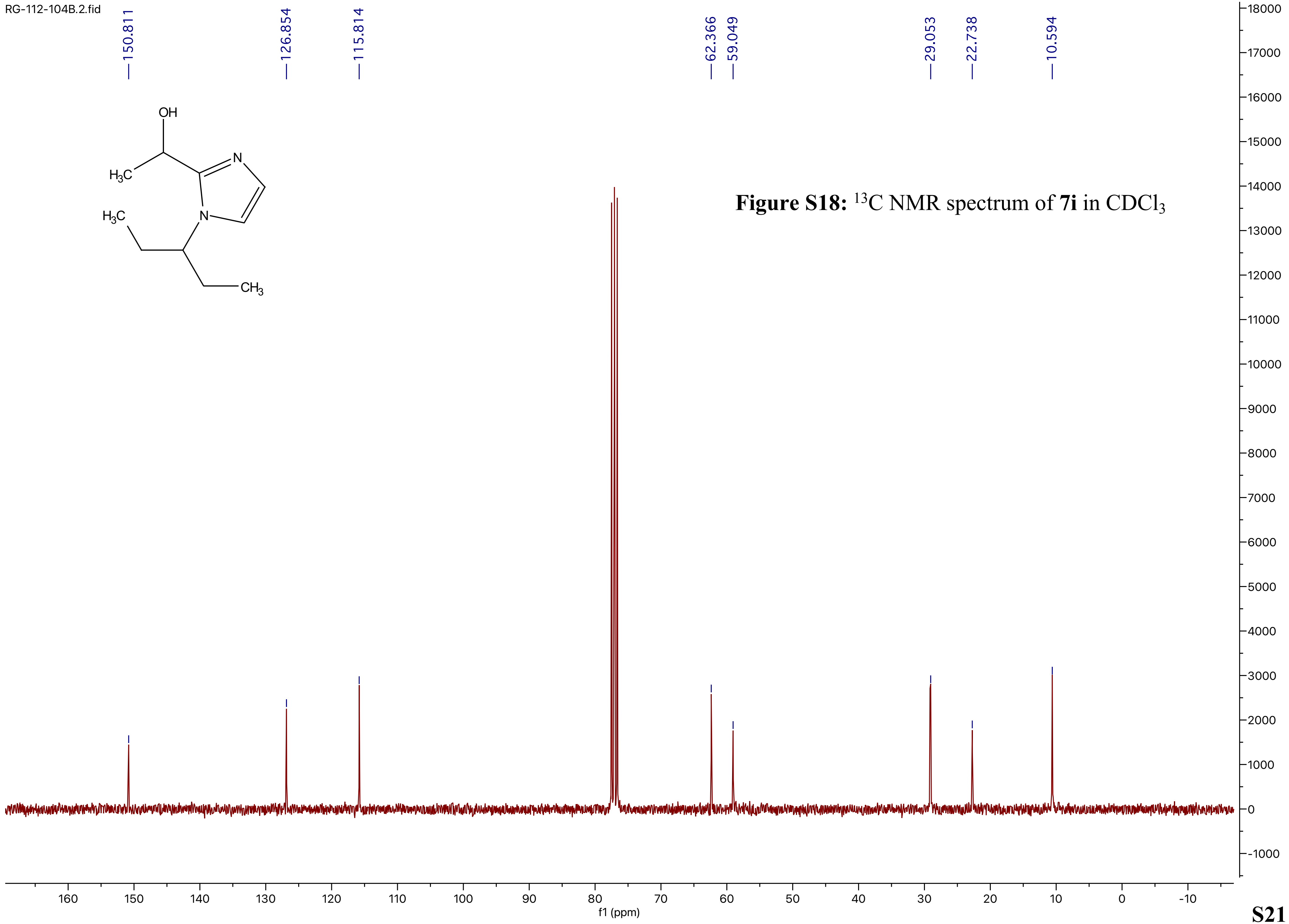

Figure S18: ^{13}C NMR spectrum of **7i** in $CDCl_3$

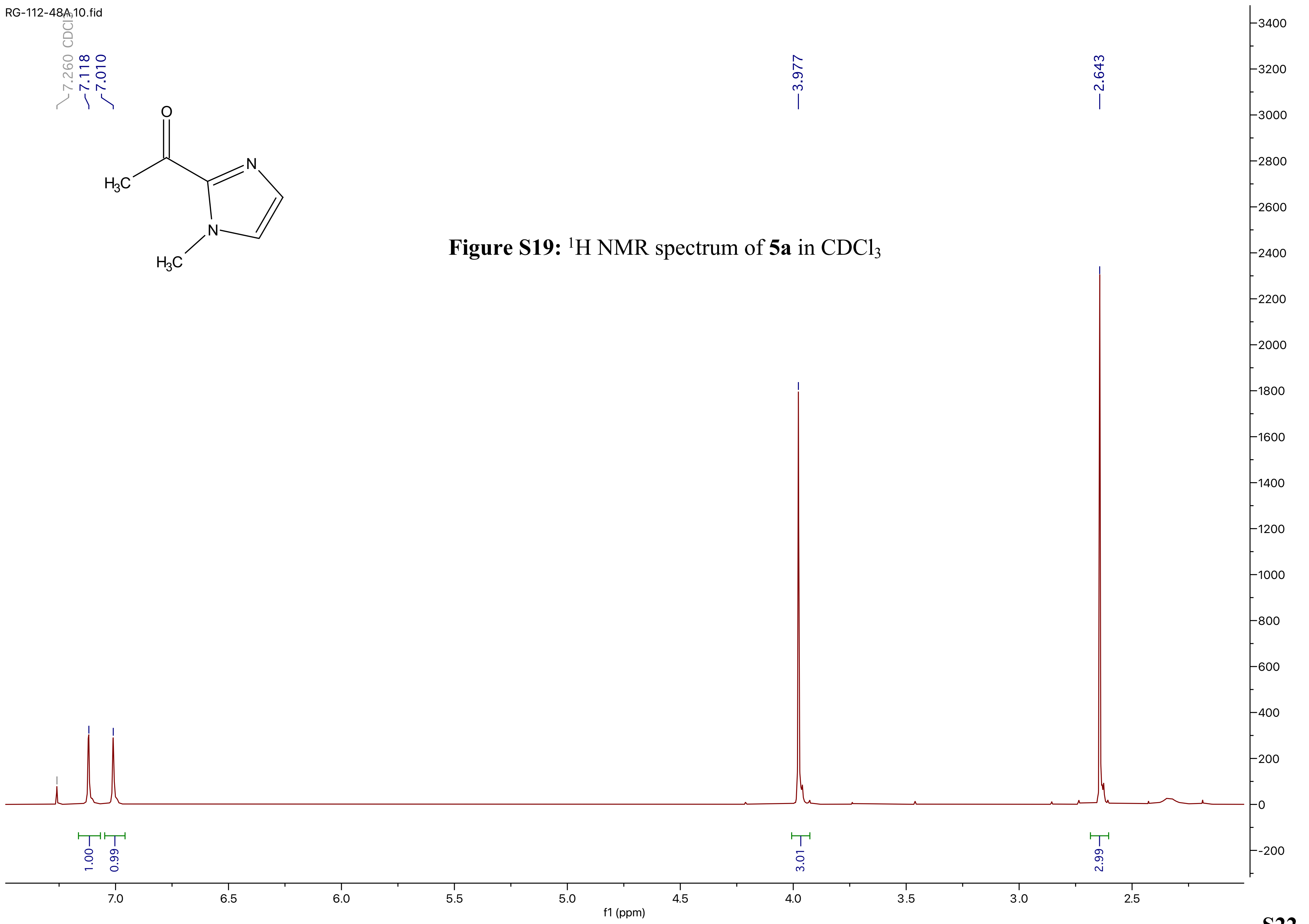

Figure S19: ^{1}H NMR spectrum of **5a** in $CDCl_3$

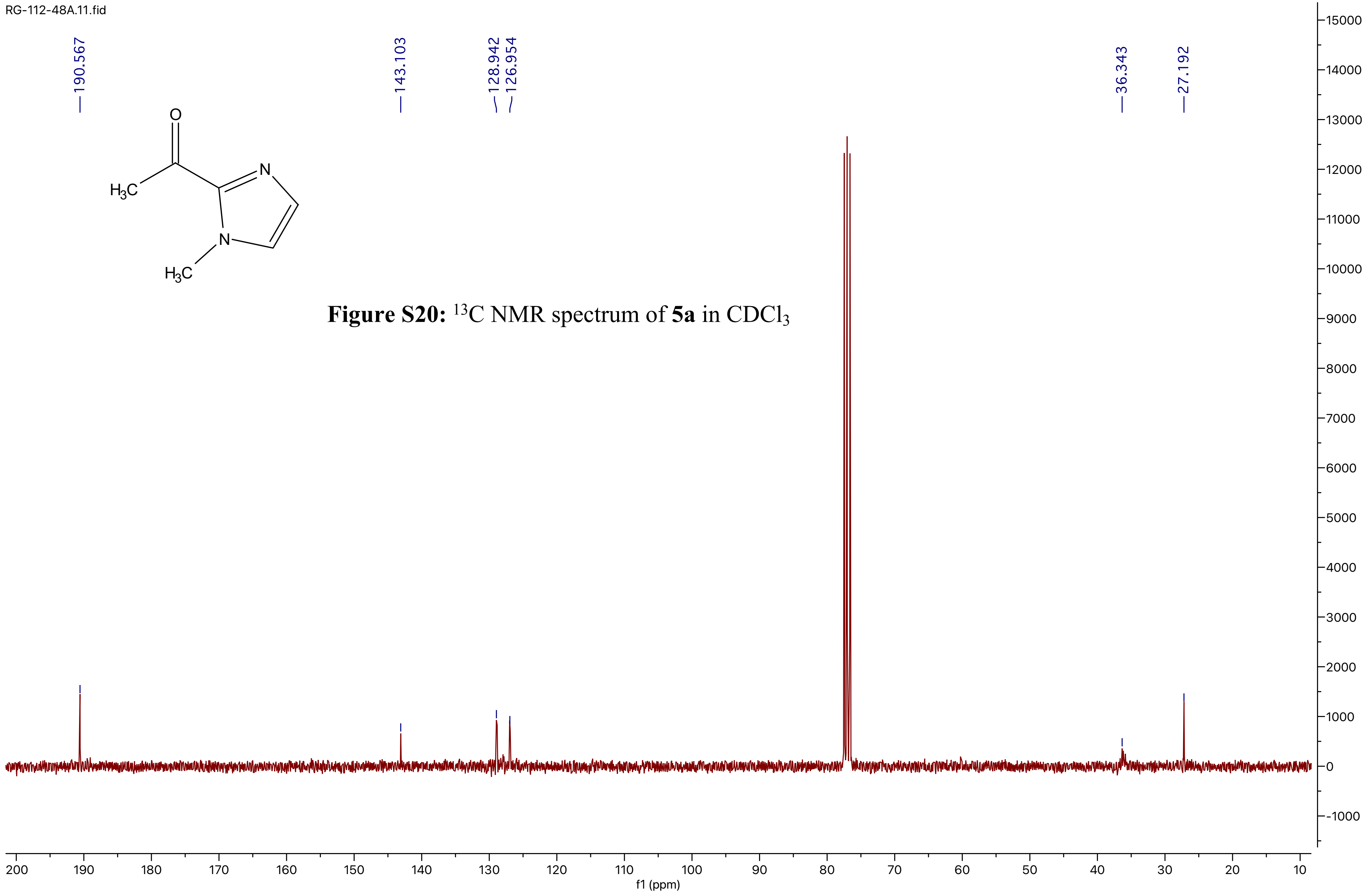

Figure S20: ^{13}C NMR spectrum of **5a** in $CDCl_3$

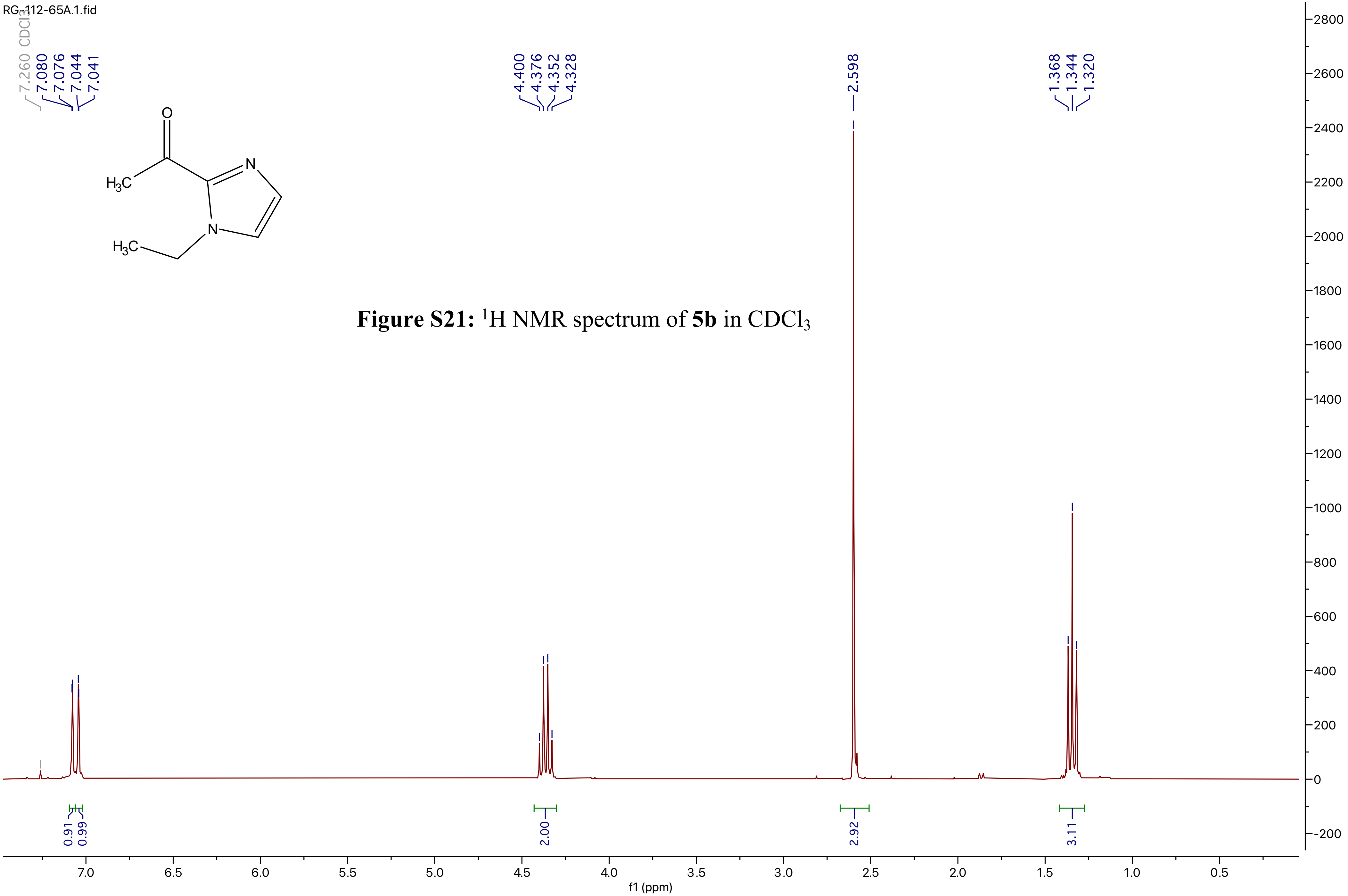

Figure S21: ^{1}H NMR spectrum of **5b** in $CDCl_3$

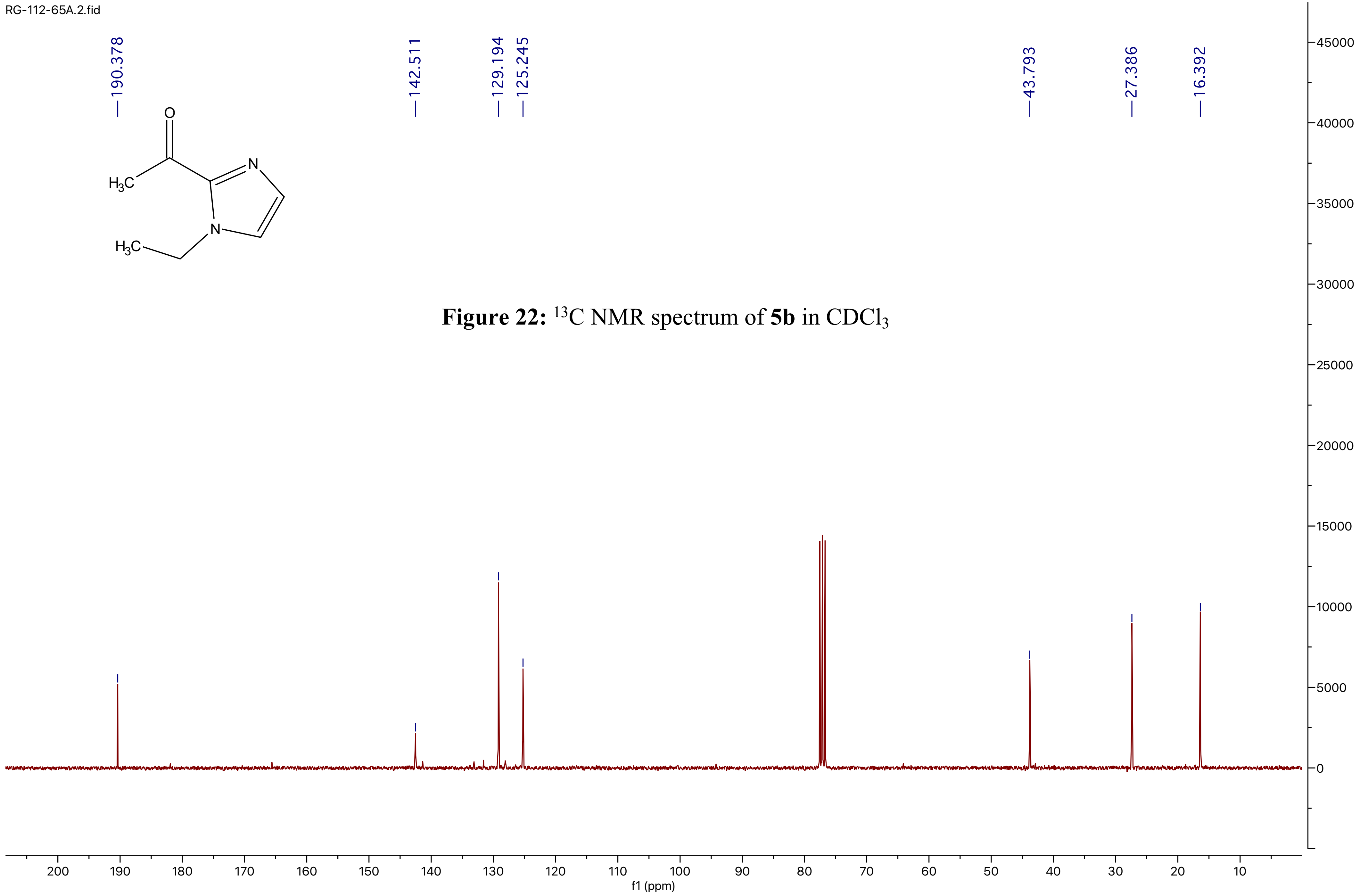

Figure 22: ^{13}C NMR spectrum of **5b** in $CDCl_3$

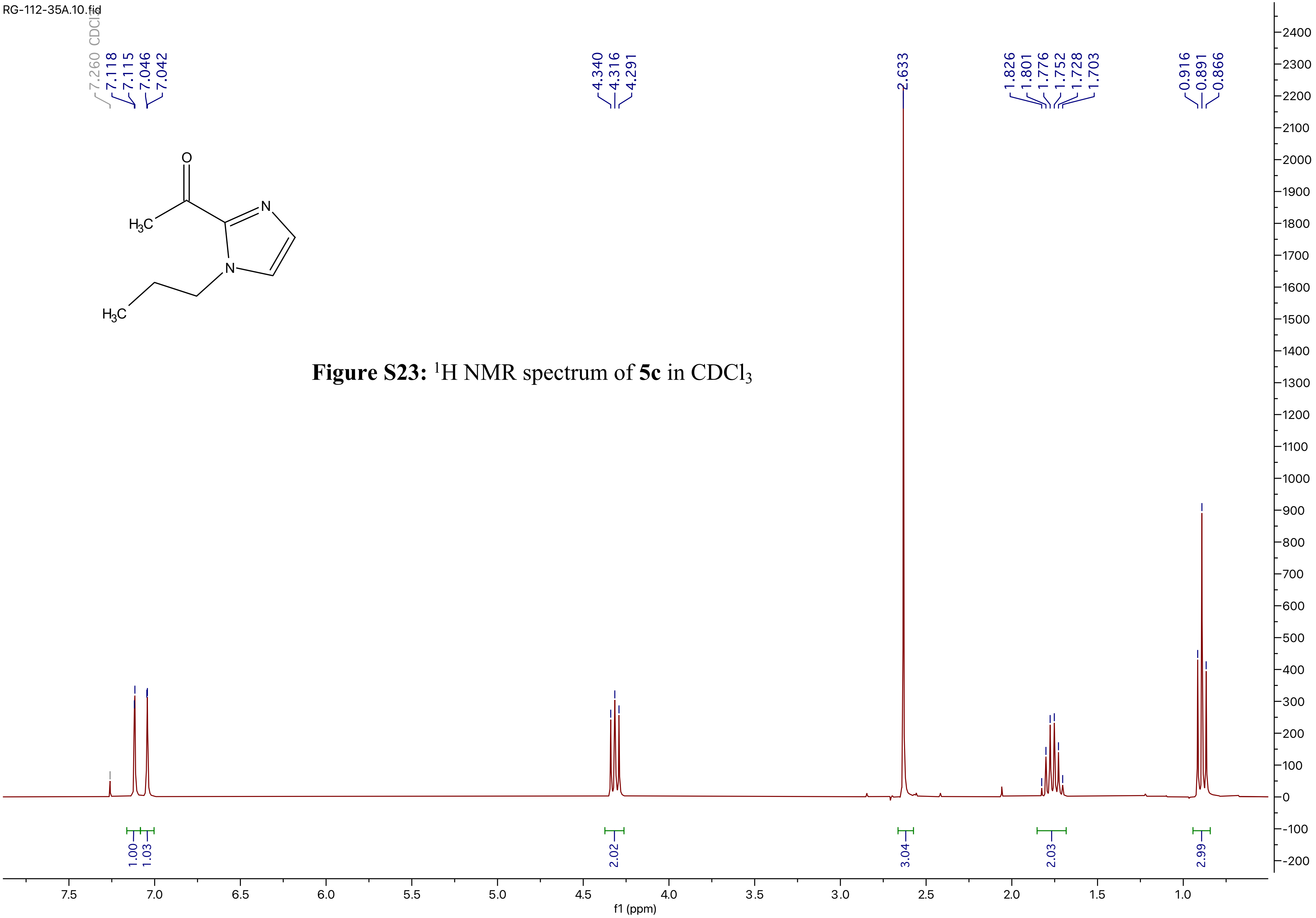

Figure S23: ^{1}H NMR spectrum of **5c** in $CDCl_3$

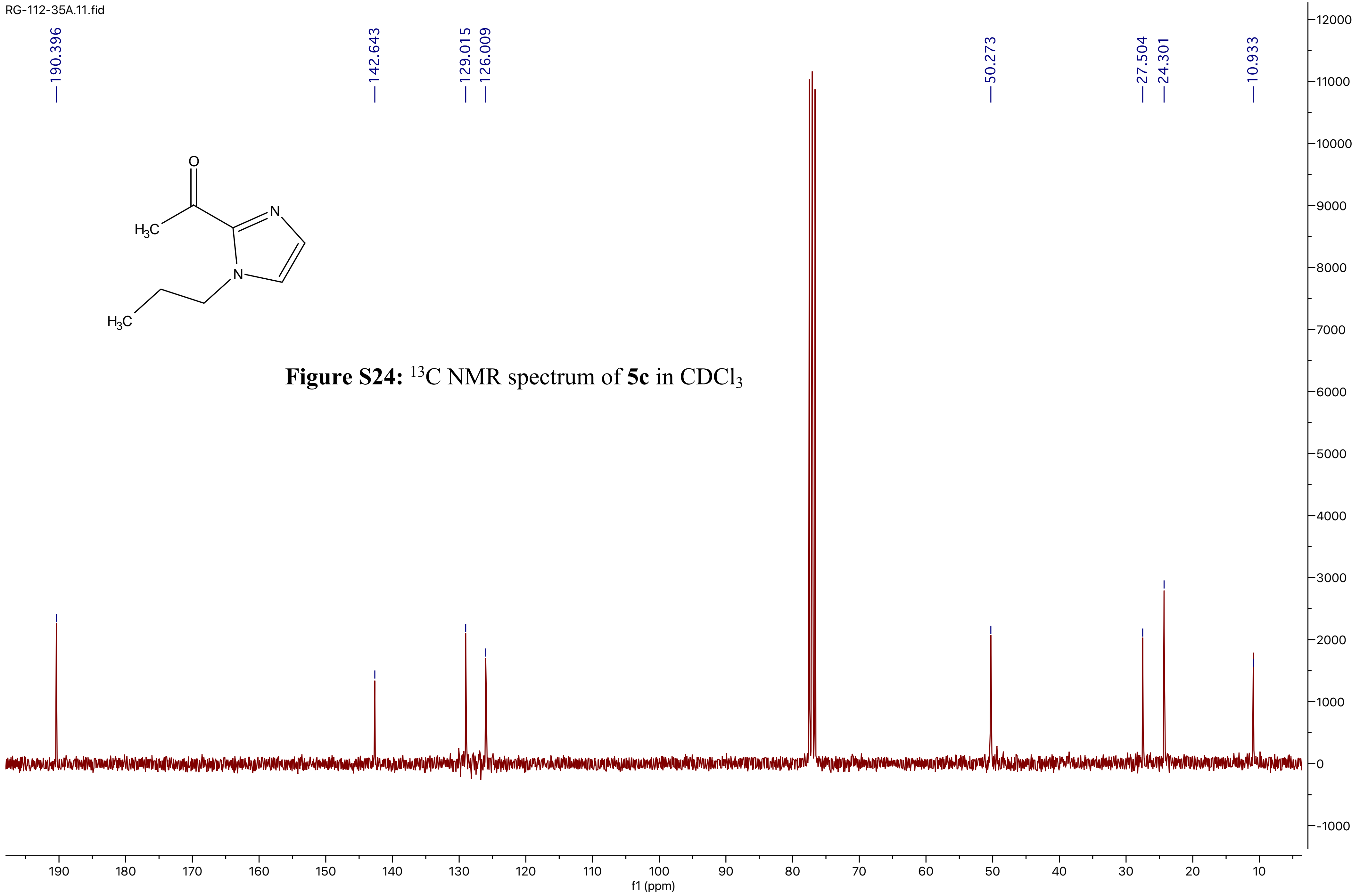

Figure S24: ^{13}C NMR spectrum of **5c** in $CDCl_3$

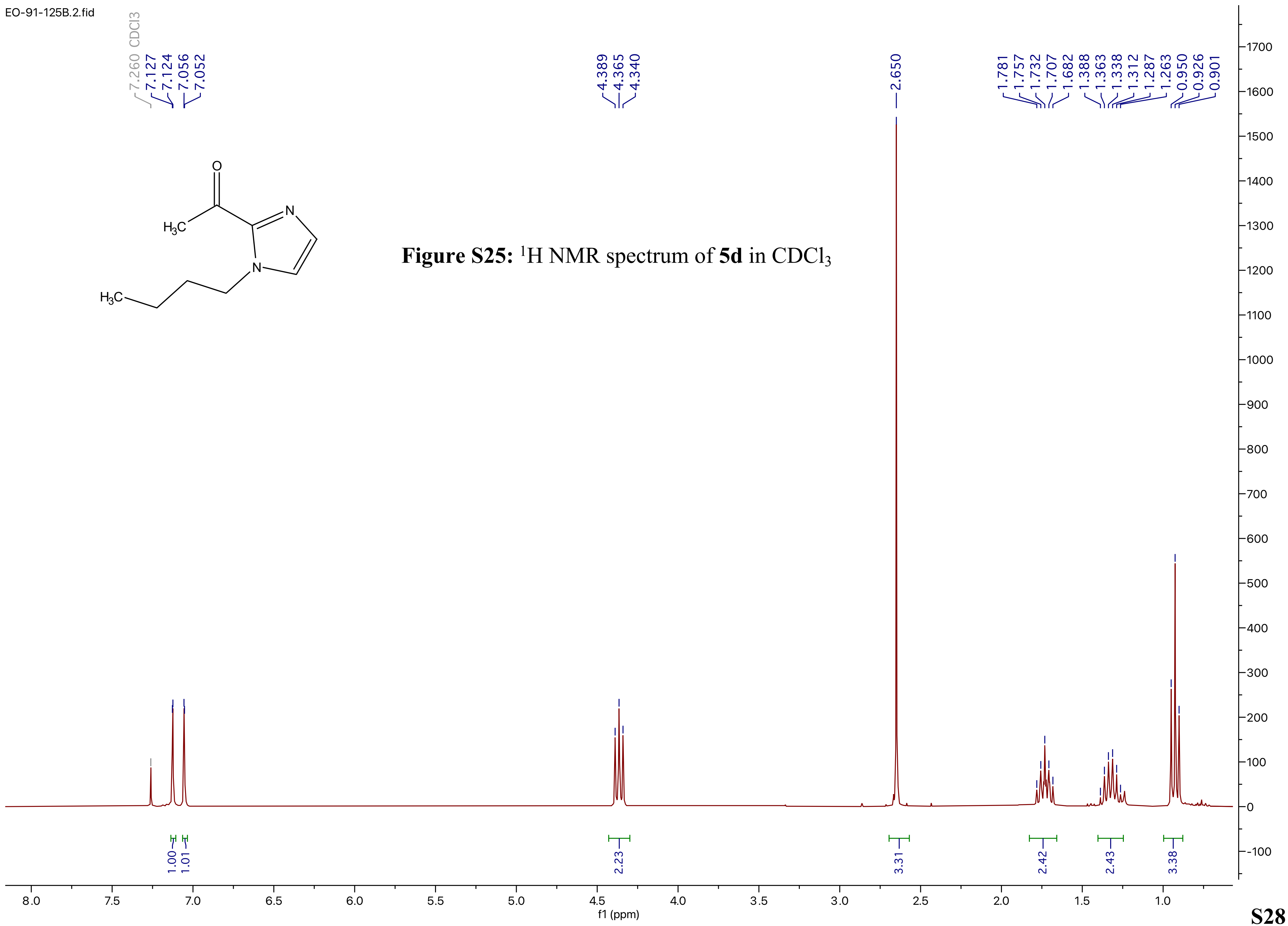

Figure S25: ^{1}H NMR spectrum of **5d** in $CDCl_3$

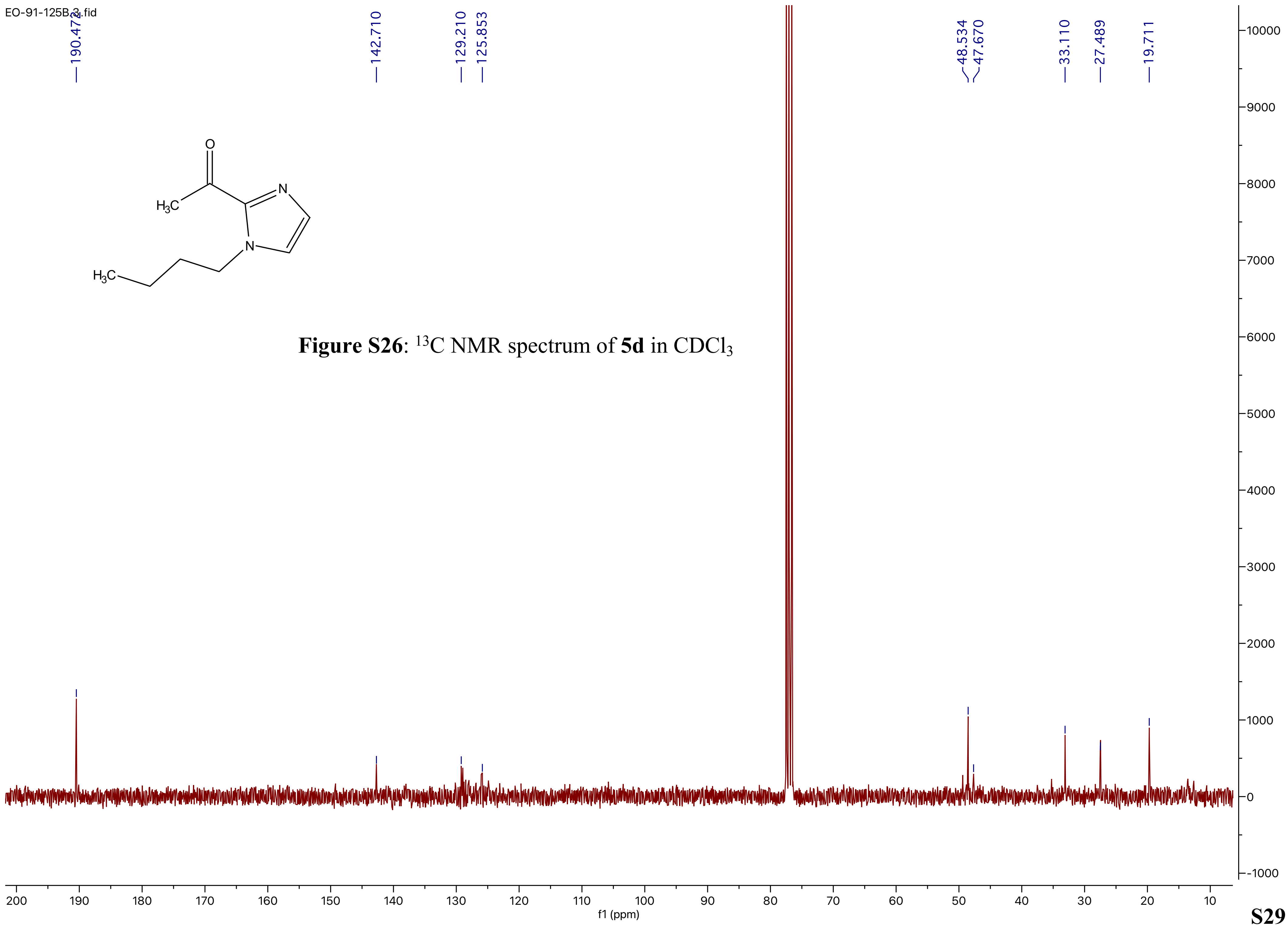

Figure S26: ^{13}C NMR spectrum of **5d** in $CDCl_3$

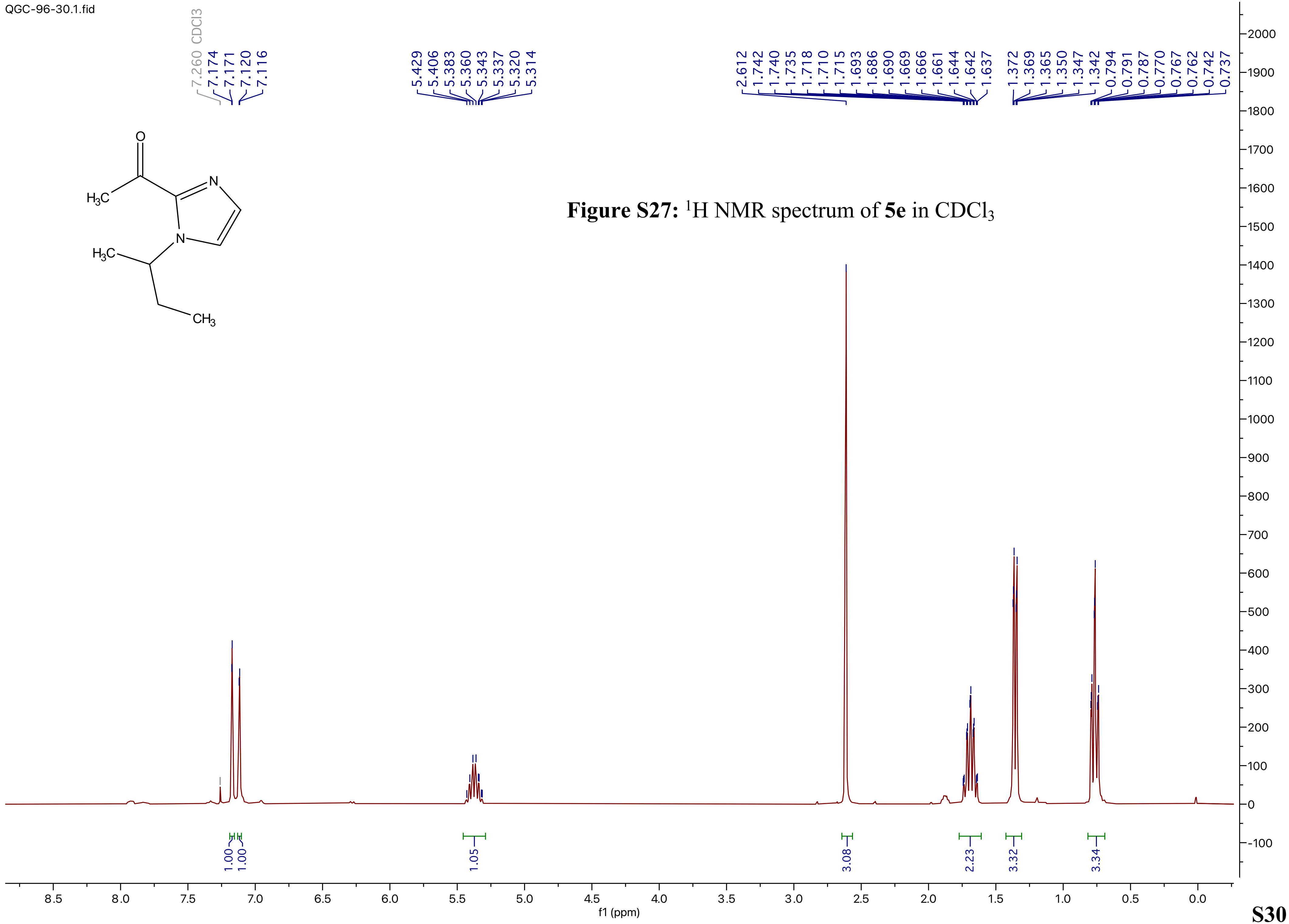

Figure S27: ^{1}H NMR spectrum of **5e** in $CDCl_3$

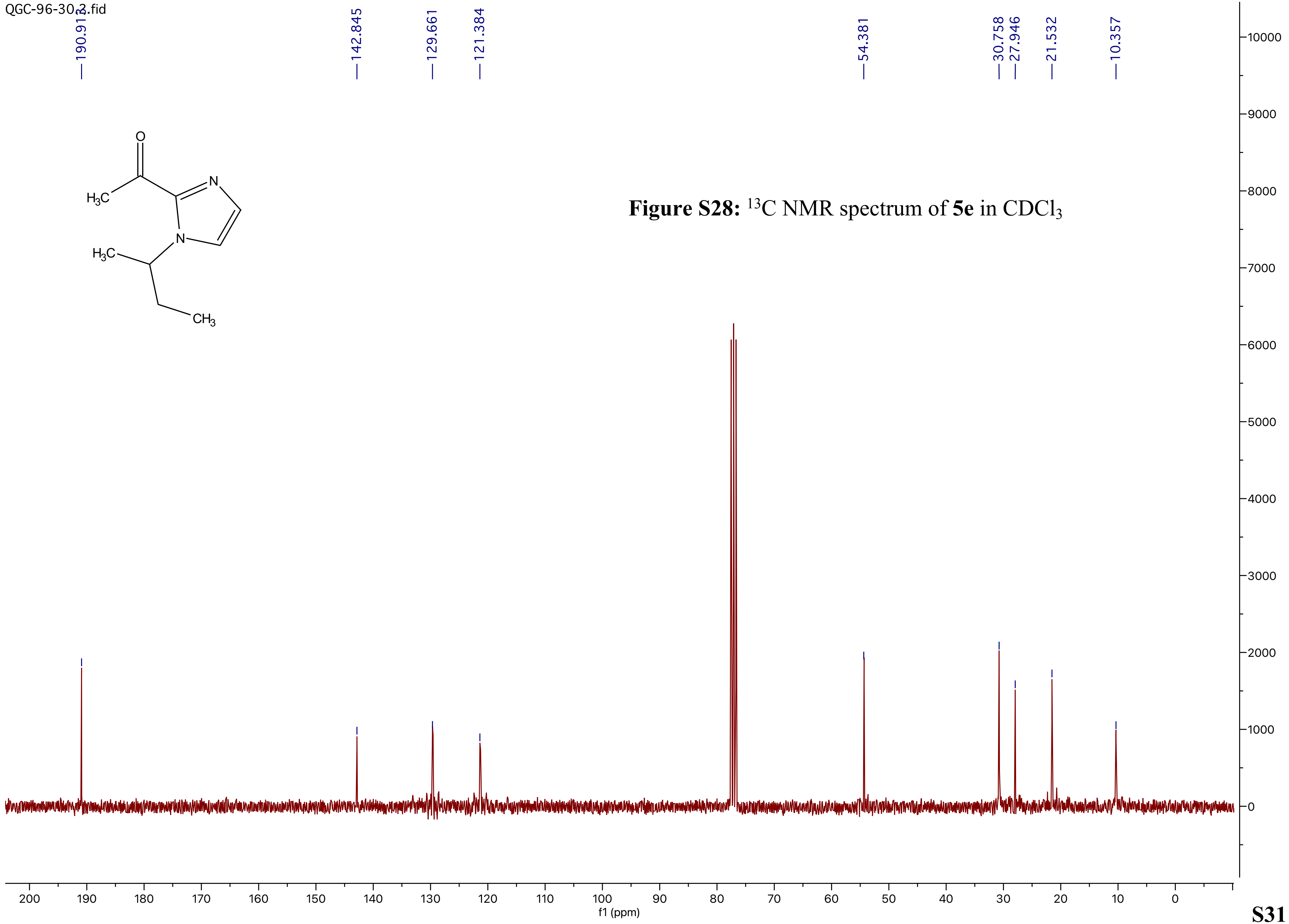

Figure S28: ^{13}C NMR spectrum of **5e** in $CDCl_3$

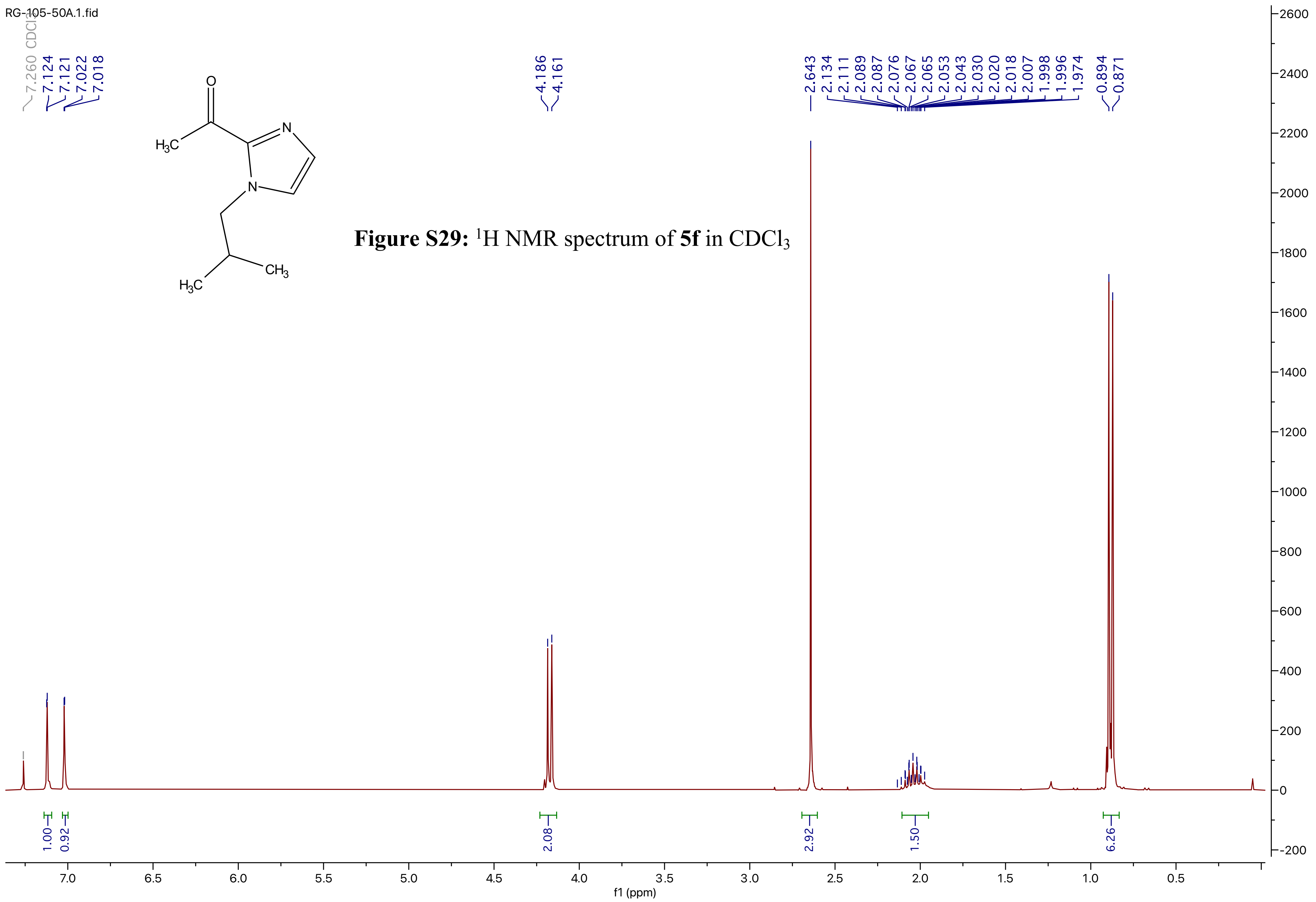

Figure S29: ^{1}H NMR spectrum of **5f** in $CDCl_3$

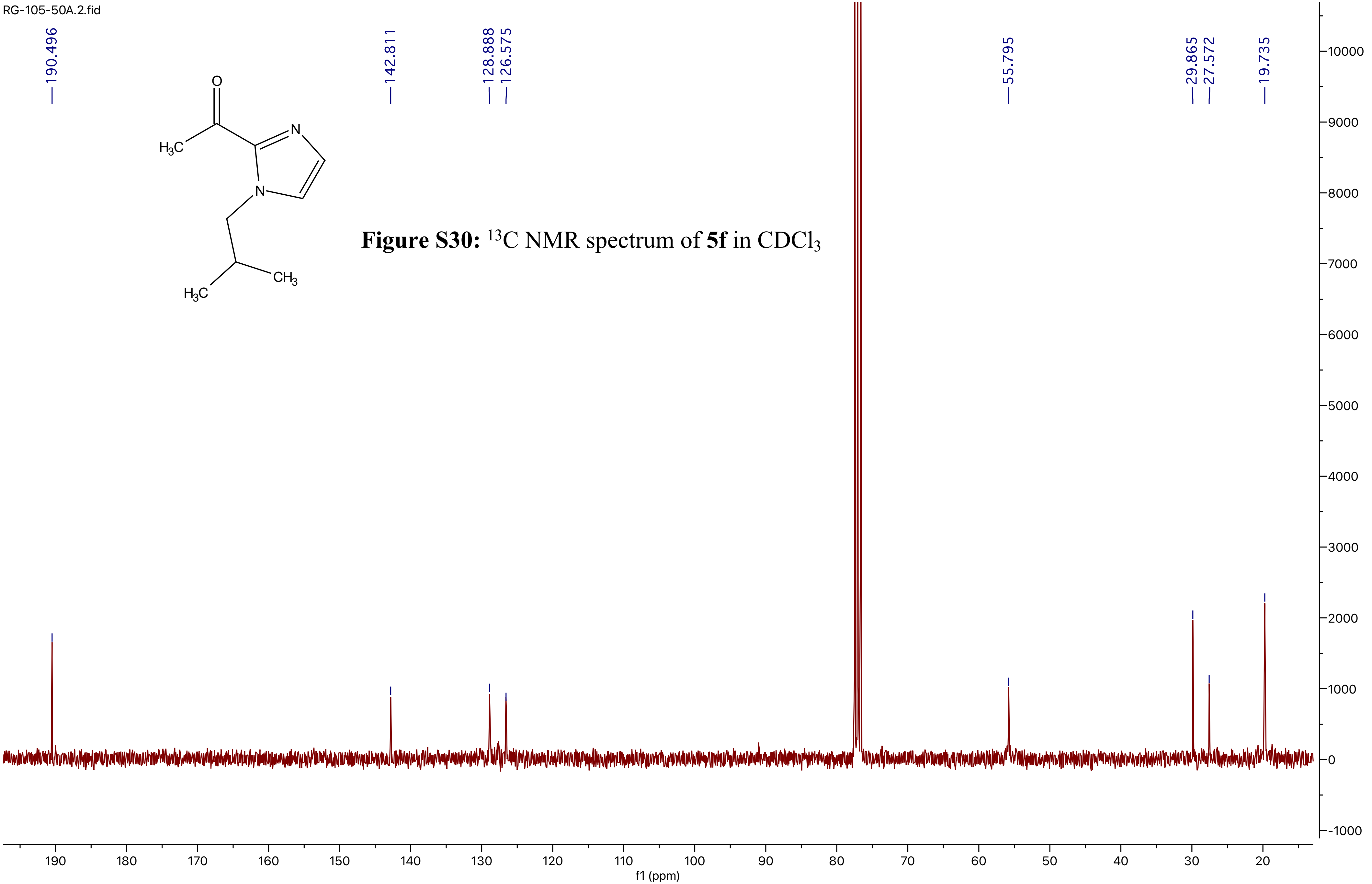

Figure S30: ^{13}C NMR spectrum of **5f** in $CDCl_3$

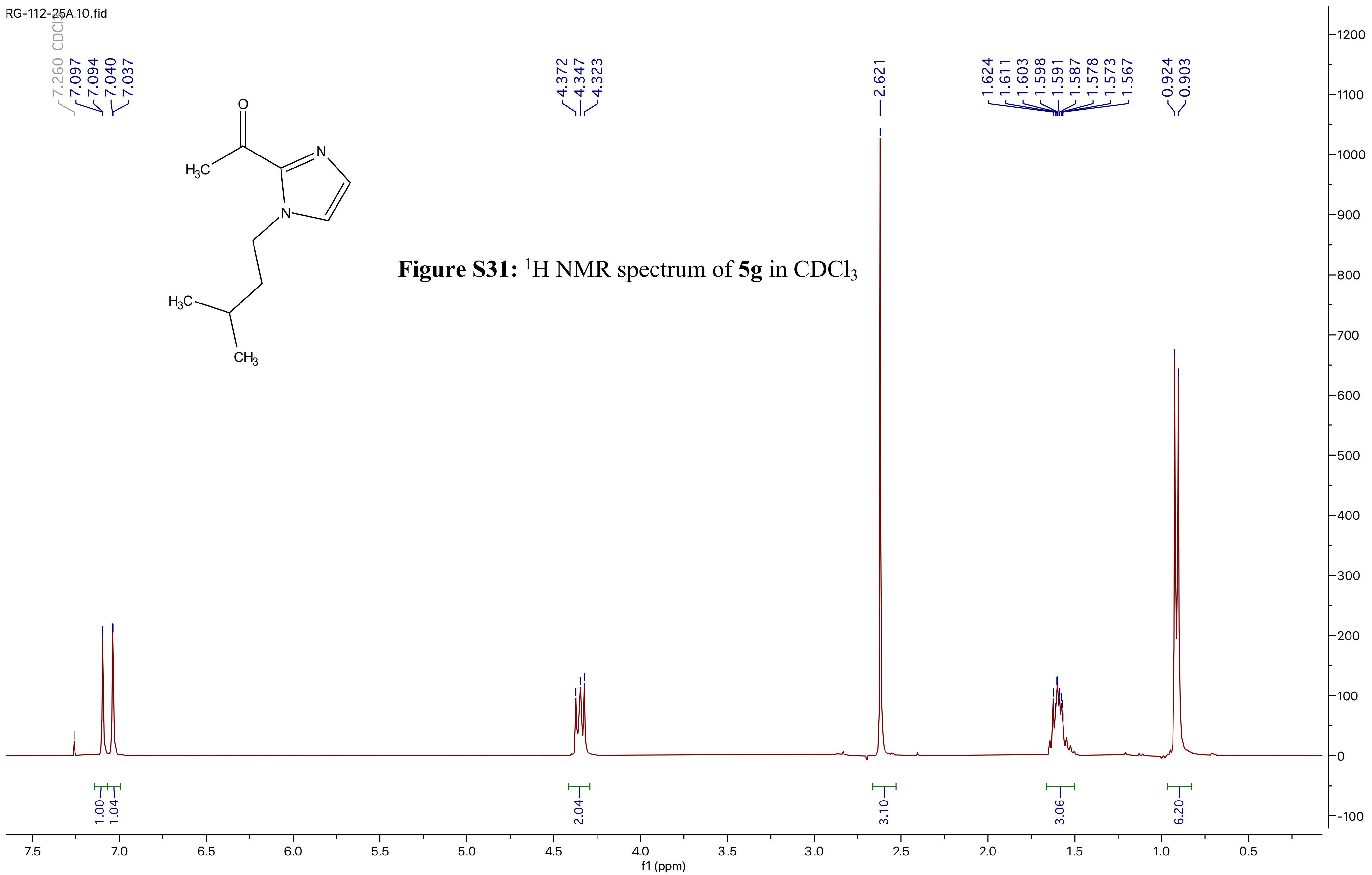

Figure S31: ^{1}H NMR spectrum of **5g** in $CDCl_3$

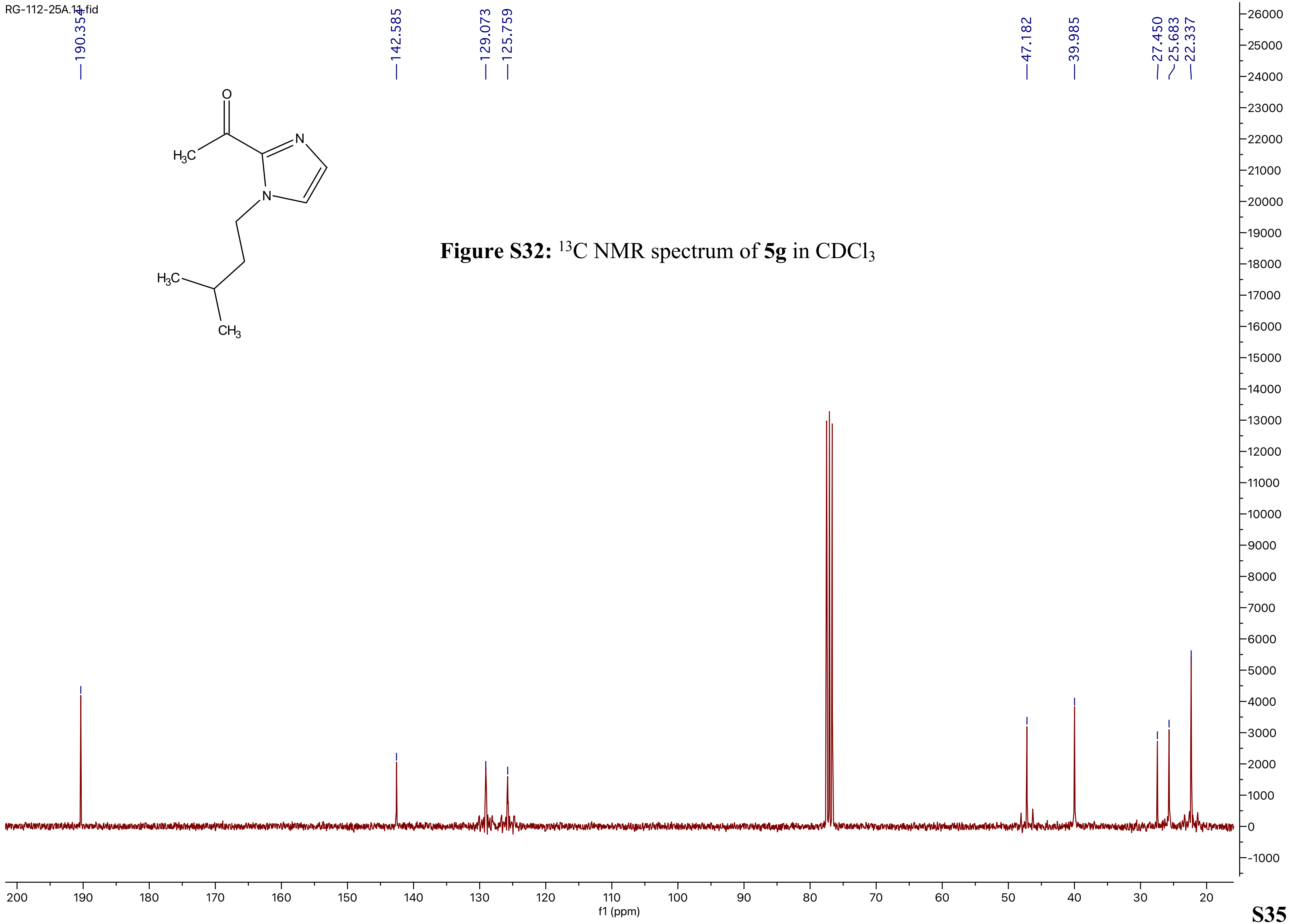

Figure S32: ^{13}C NMR spectrum of **5g** in $CDCl_3$

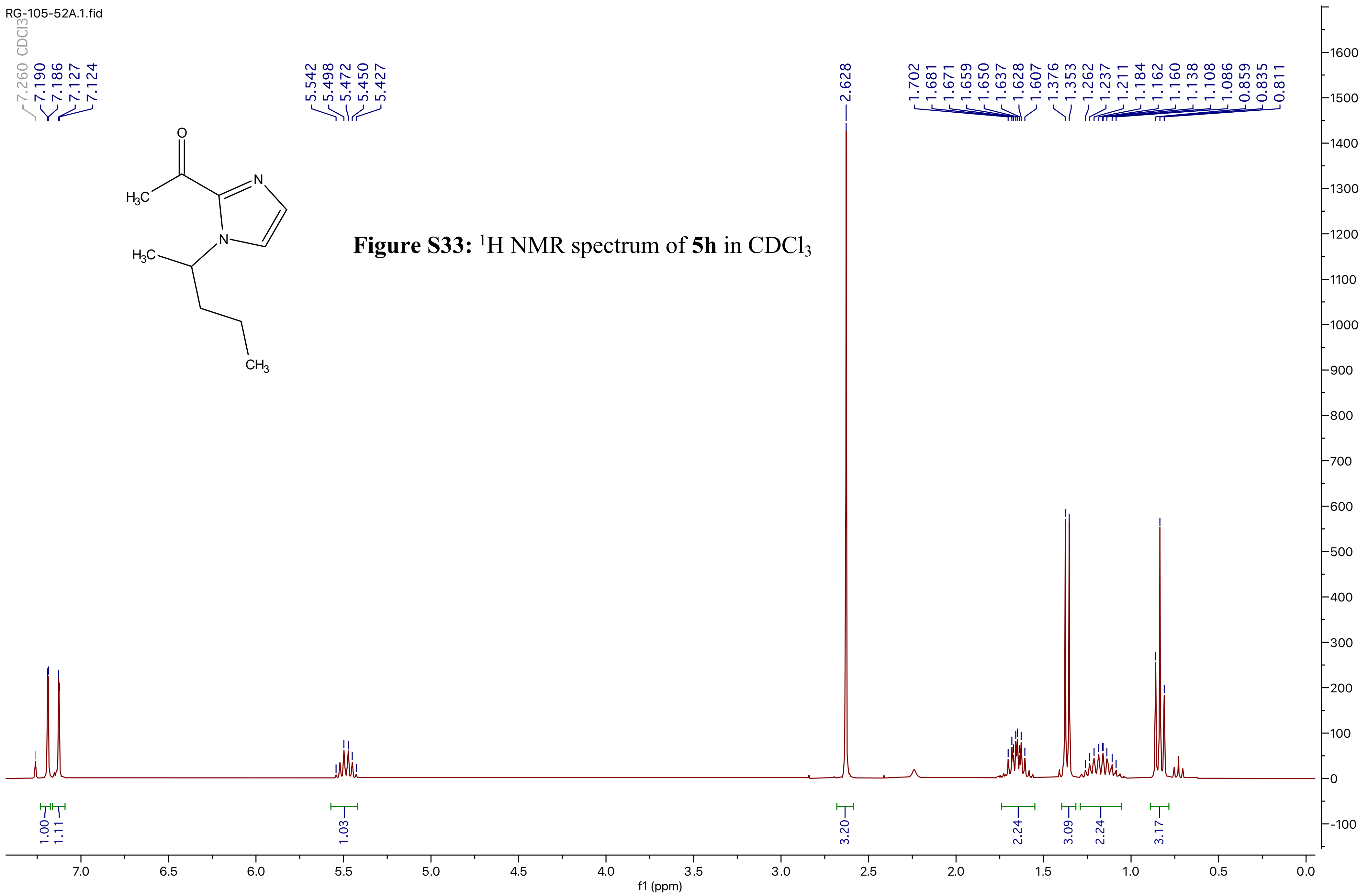

Figure S33: ^{1}H NMR spectrum of **5h** in $CDCl_3$

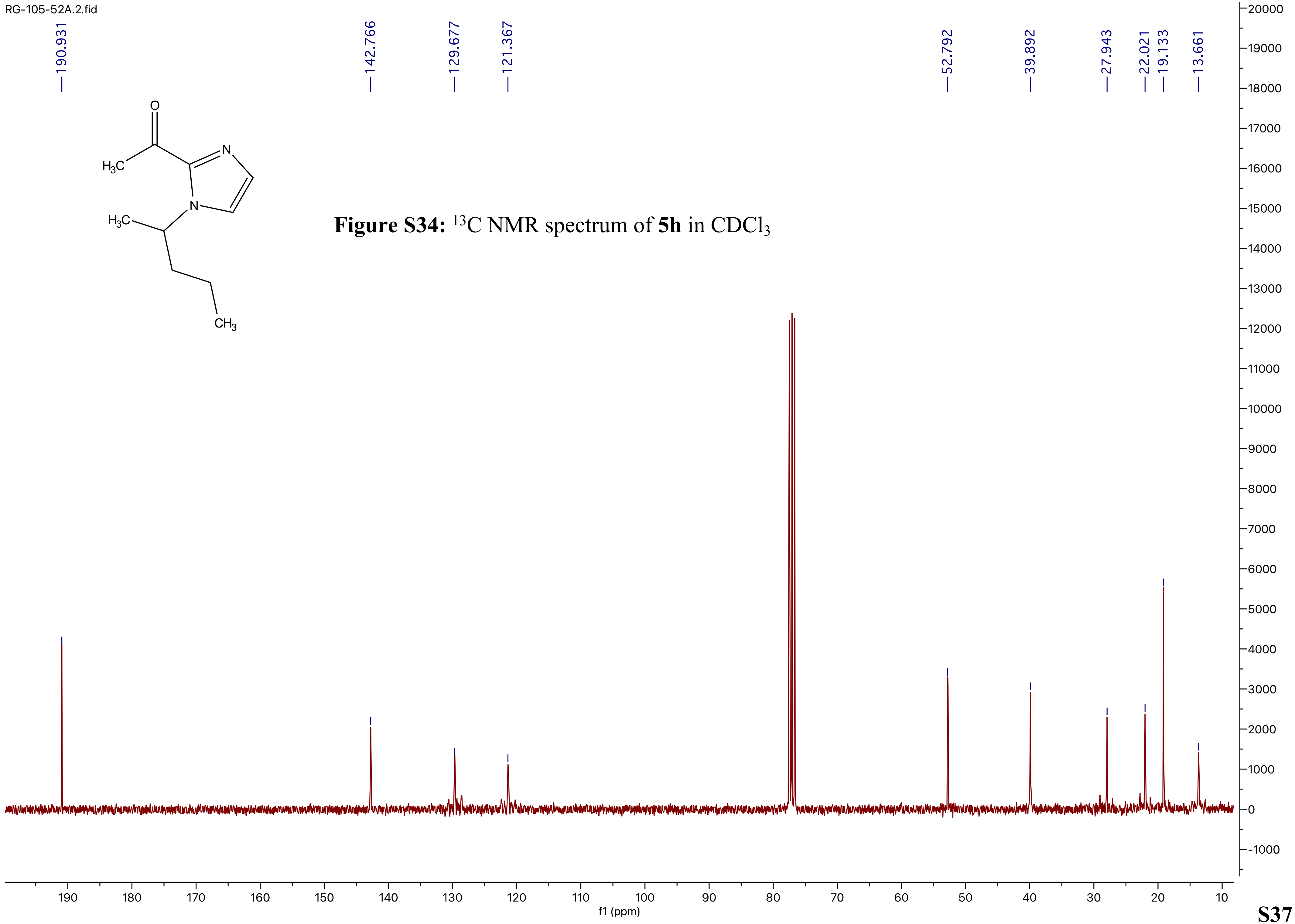

Figure S34: ^{13}C NMR spectrum of **5h** in $CDCl_3$

Figure S35: ^{1}H NMR spectrum of **5i** in $CDCl_3$

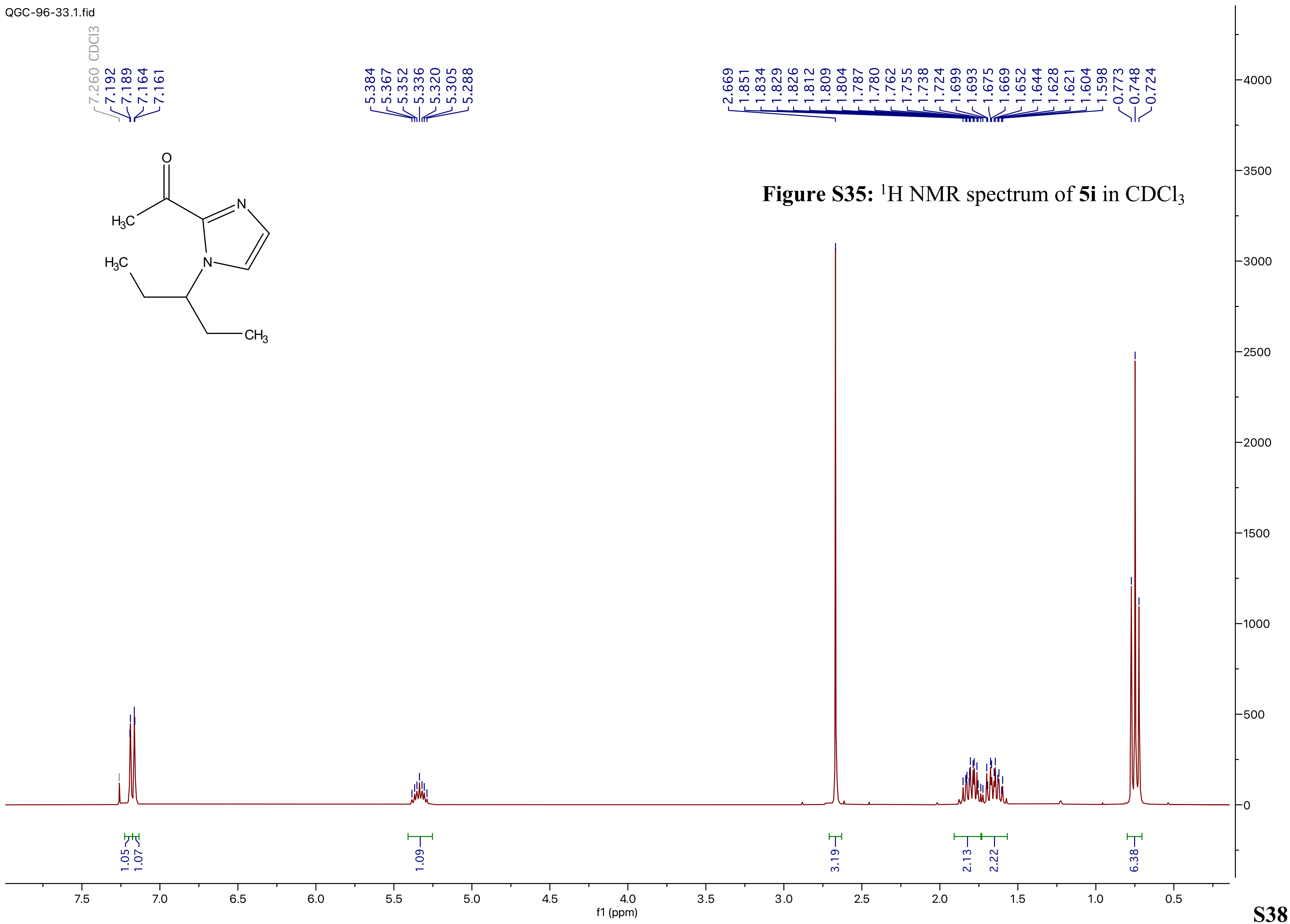

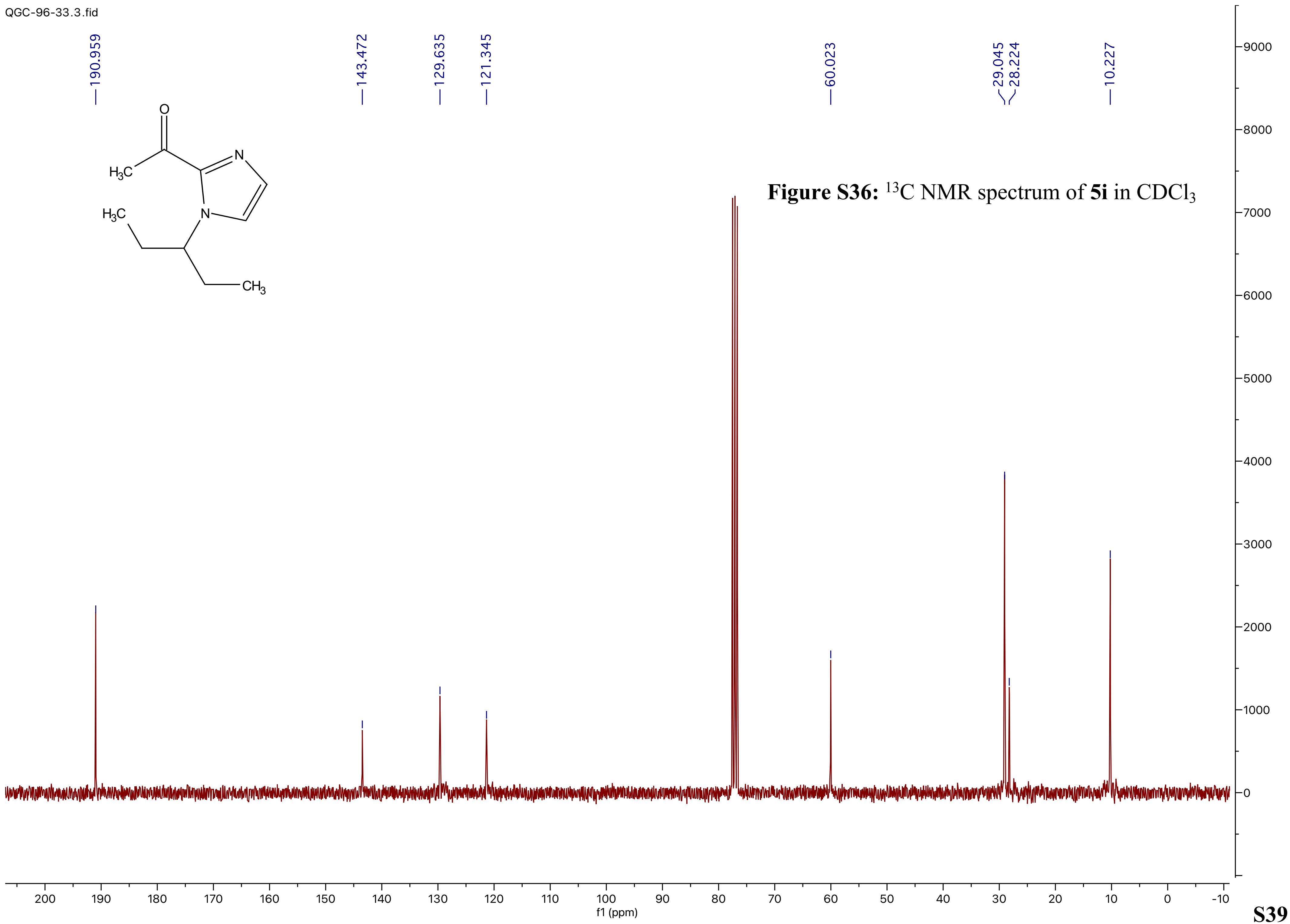

Figure S36: ^{13}C NMR spectrum of **5i** in $CDCl_3$

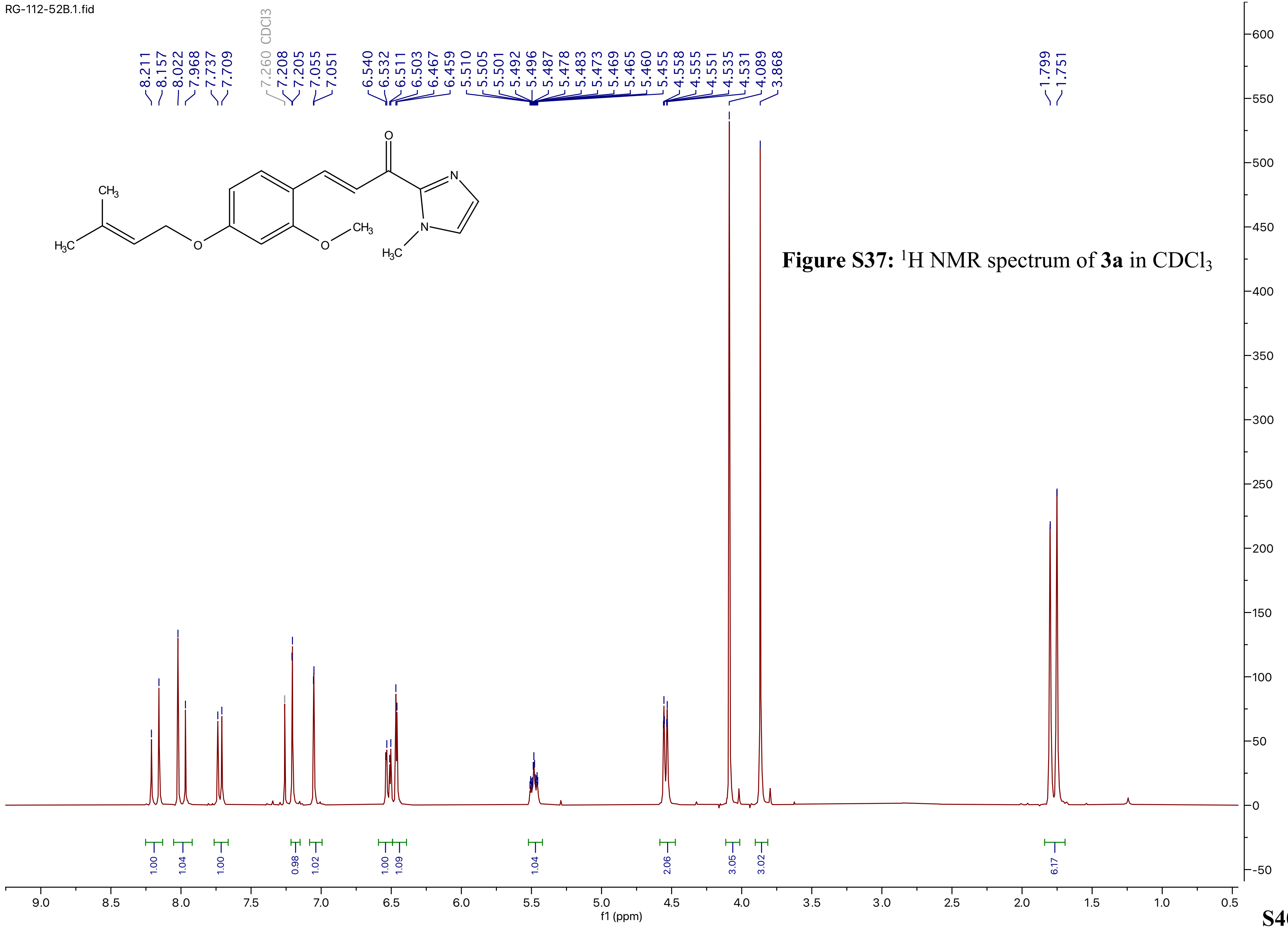

Figure S37: ^{1}H NMR spectrum of **3a** in $CDCl_3$

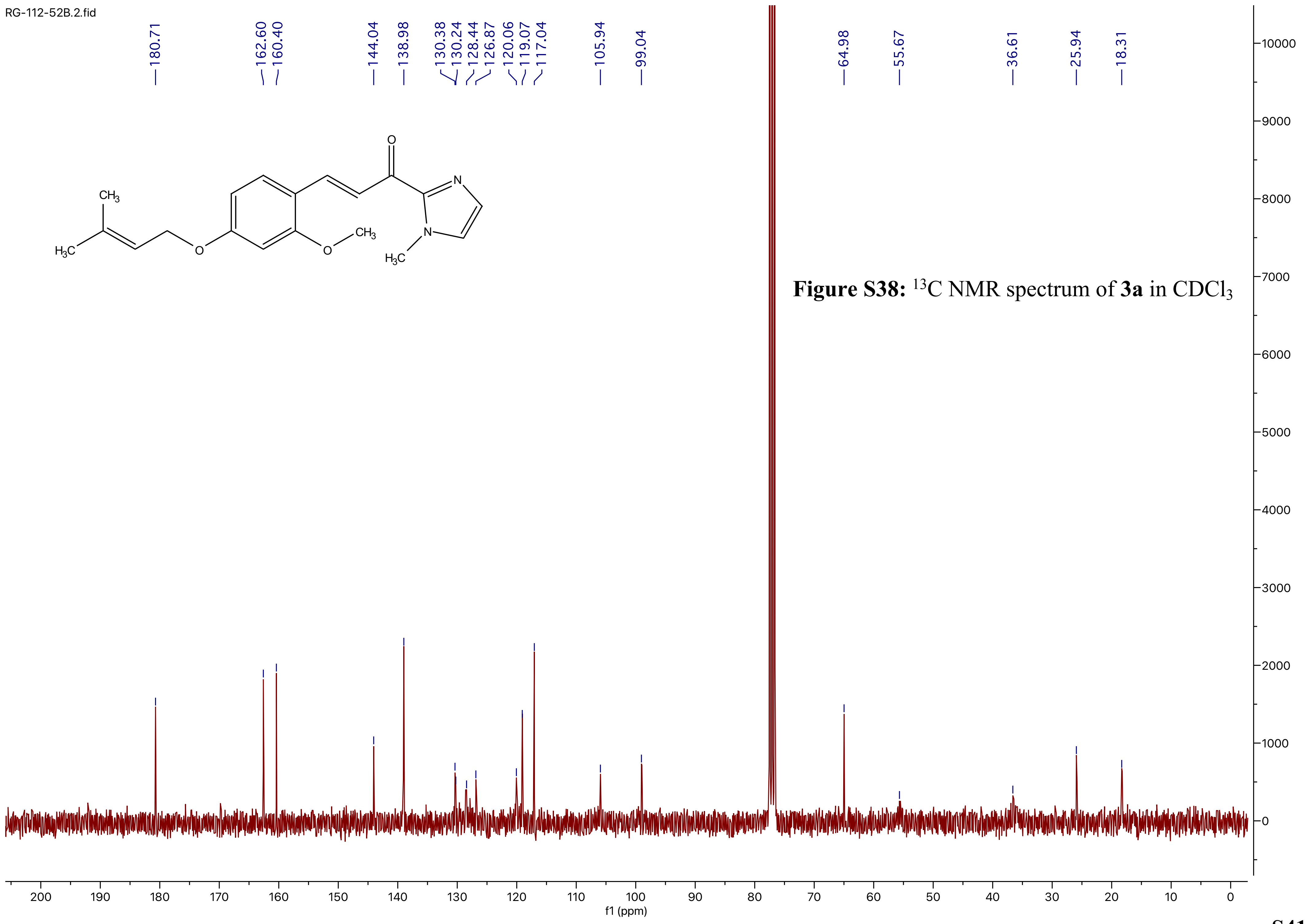

Figure S38: ^{13}C NMR spectrum of **3a** in $CDCl_3$

Smple Name	Mol Fomla	MW	M+H	obsved	dlta	ppm
RG-112-52B	C19H22N2O3	326.16306	327.1709	327.1715	0.0006	1.96

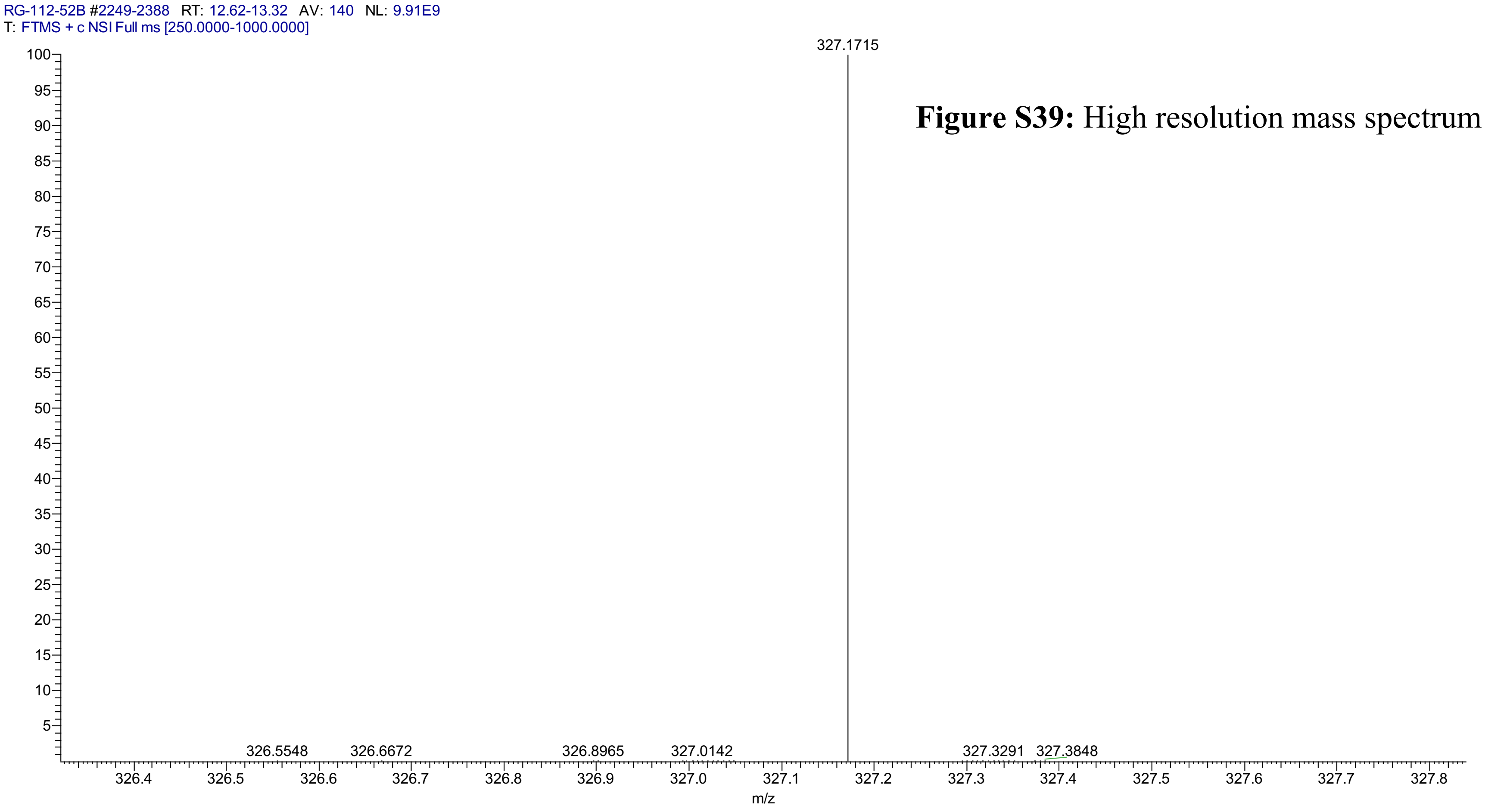

Figure S39: High resolution mass spectrum of **3a**

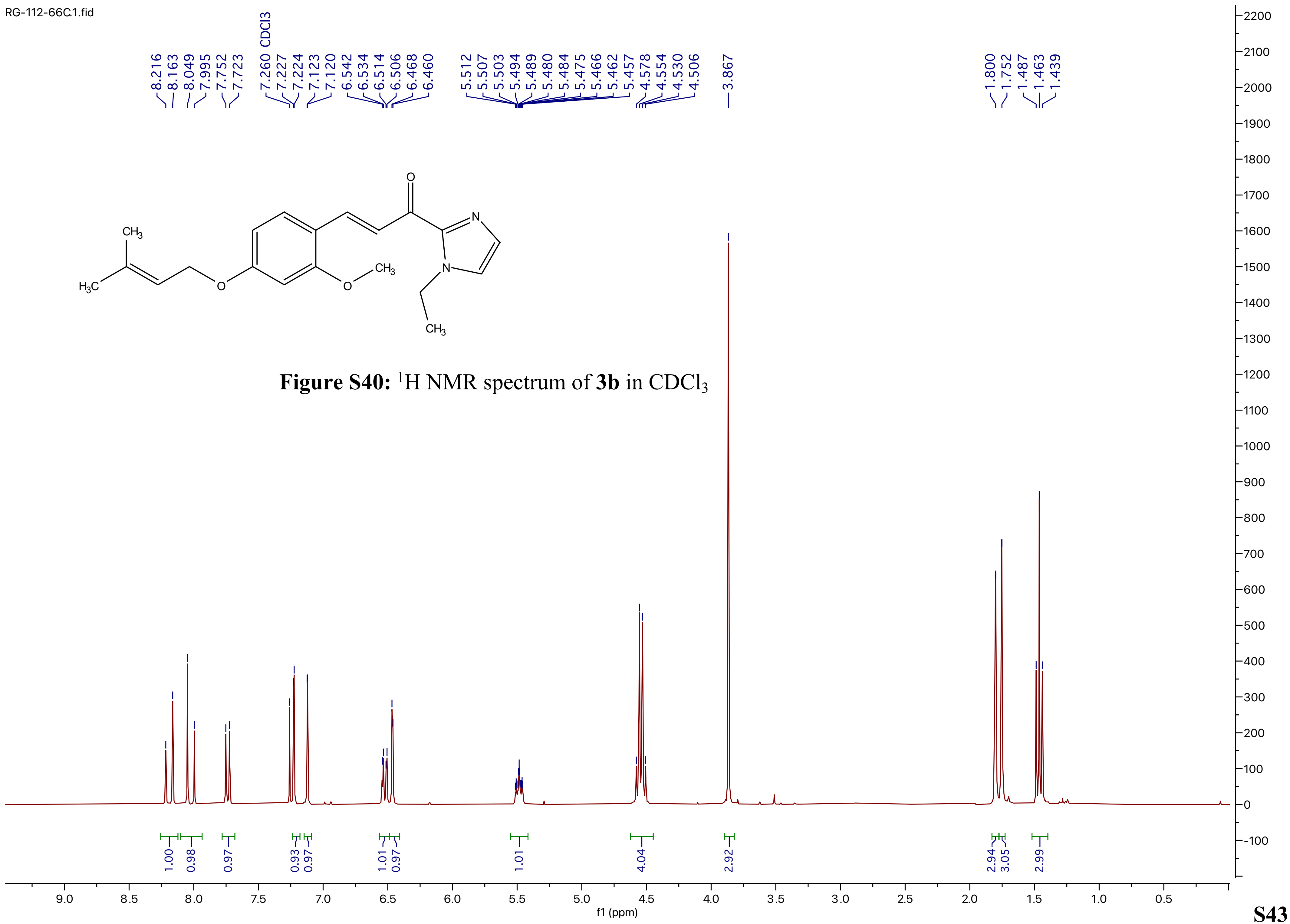

Figure S40: ^{1}H NMR spectrum of **3b** in $CDCl_3$

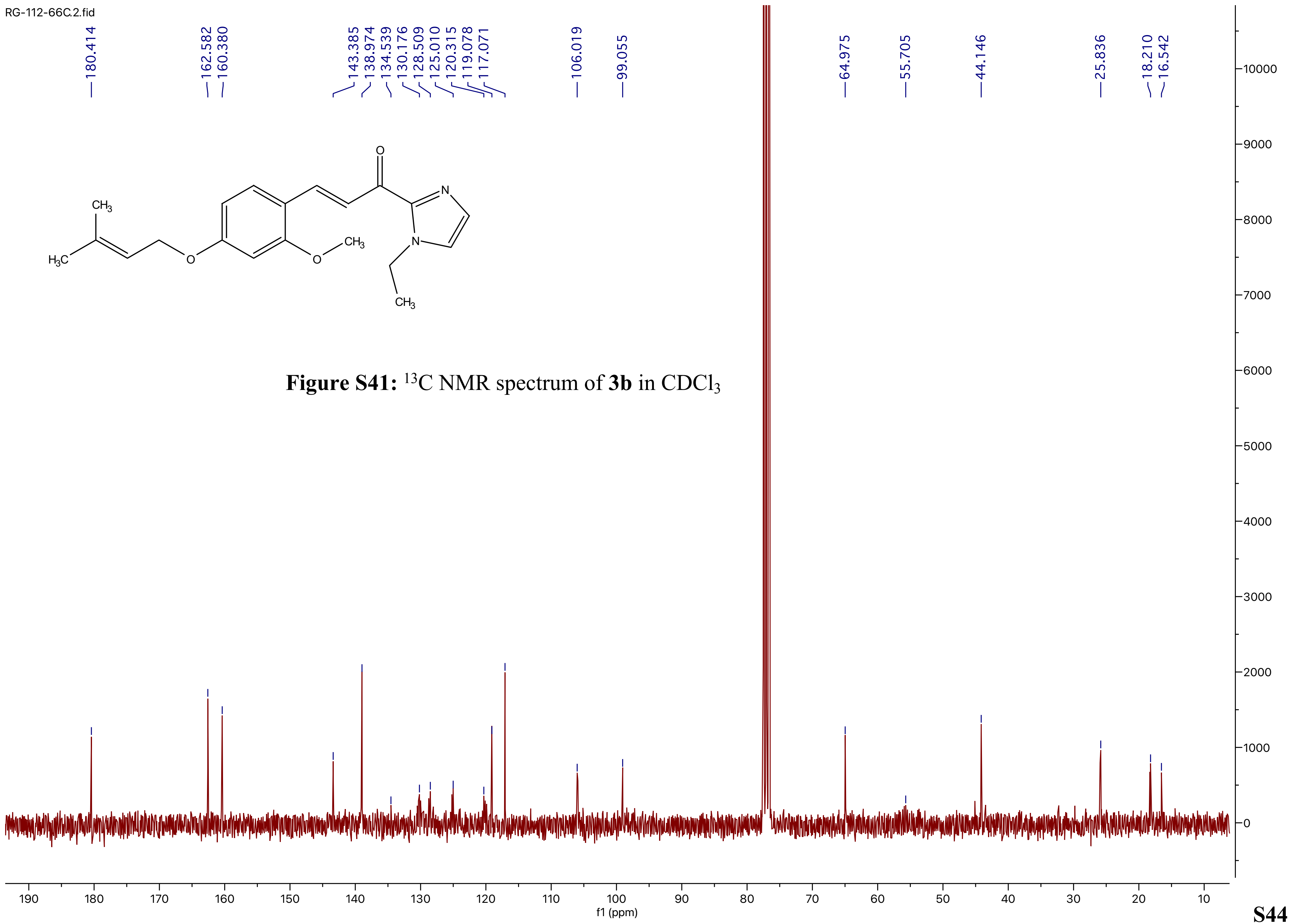

Figure S41: ^{13}C NMR spectrum of **3b** in $CDCl_3$

Smple Name	Mol Fomla	MW	M+H	obsved	dlta	ppm
RG-112-66C	C20H24N2O3	340.17871	341.1865	341.1873	0.0008	2.32

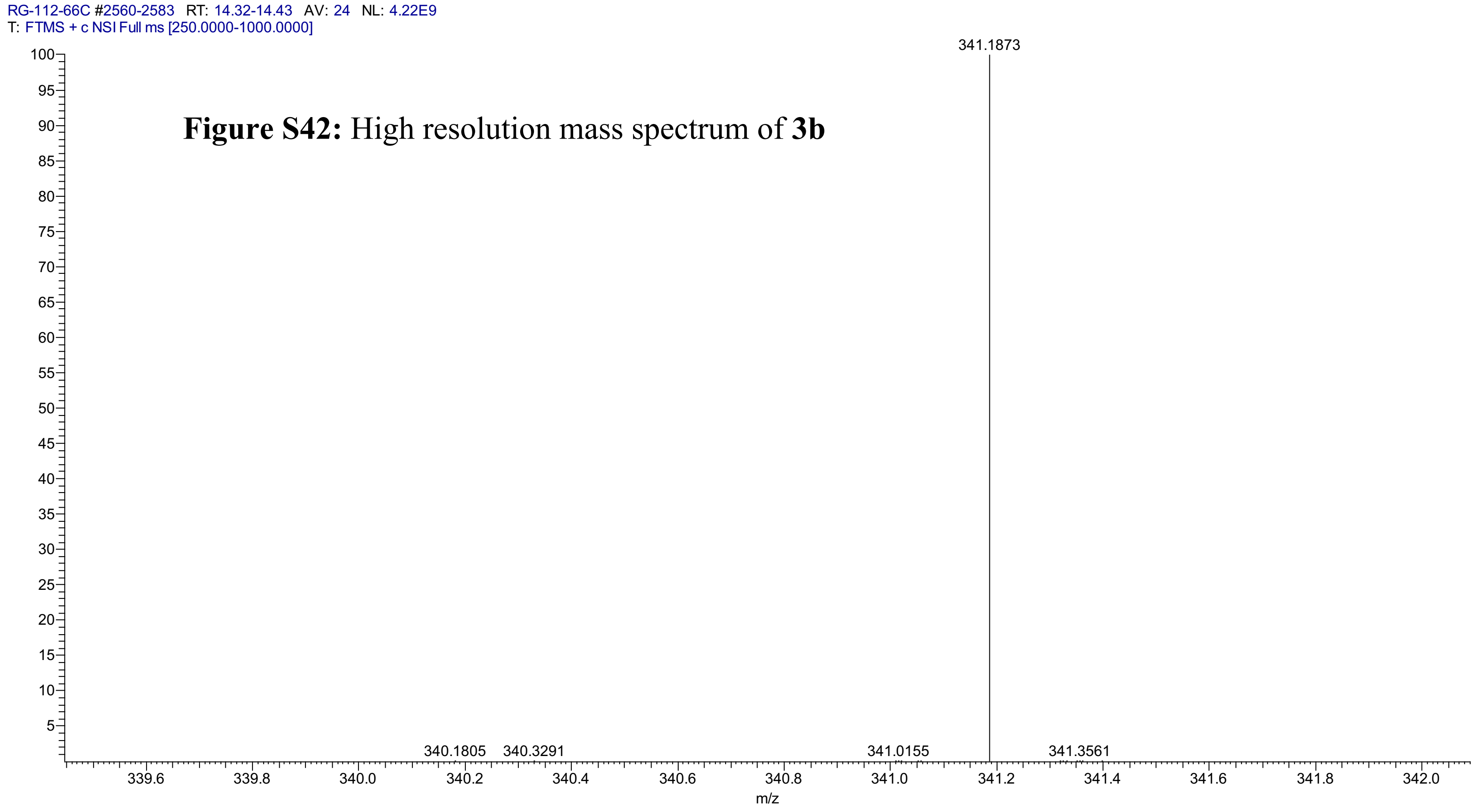

Figure S42: High resolution mass spectrum of **3b**

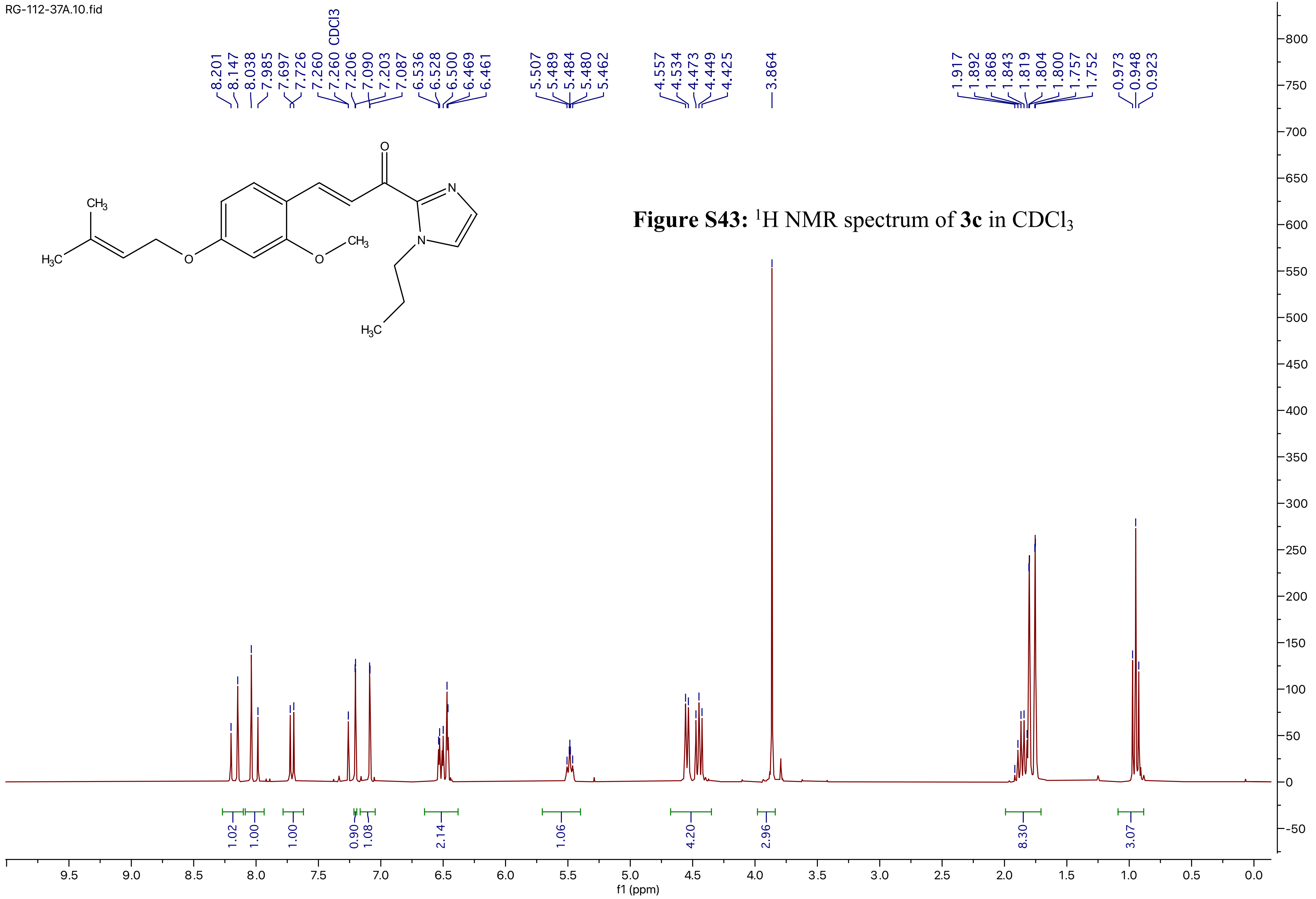

Figure S43: ^{1}H NMR spectrum of **3c** in $CDCl_3$

RG-112-37A

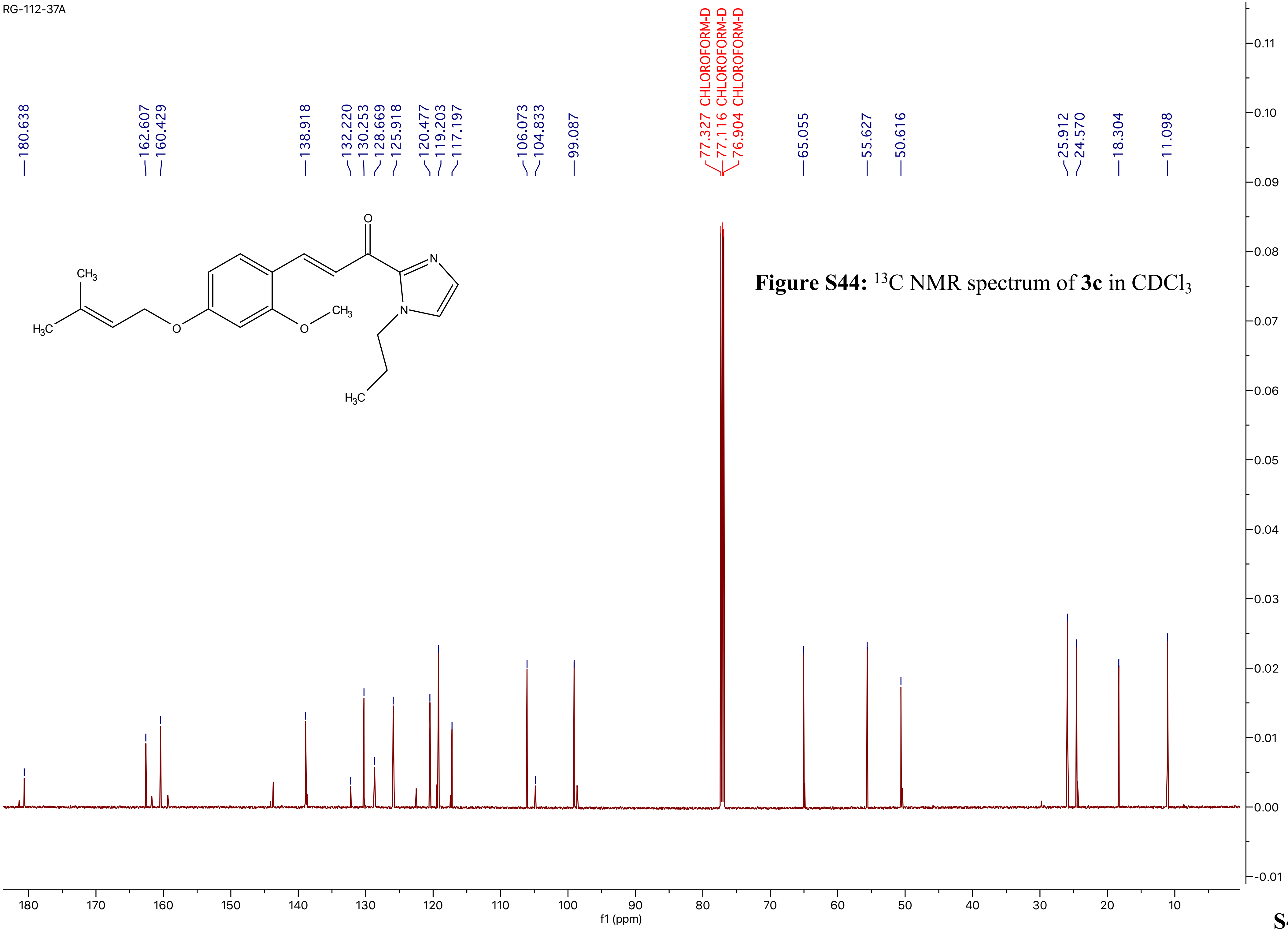

Figure S44: ^{13}C NMR spectrum of **3c** in $CDCl_3$

Smple Name	Mol Fomla	MW	M+H	obsved	dlta	ppm
RG-112-37A	C21H26N2O3	354.19436	355.2022	355.2029	0.0007	2.08

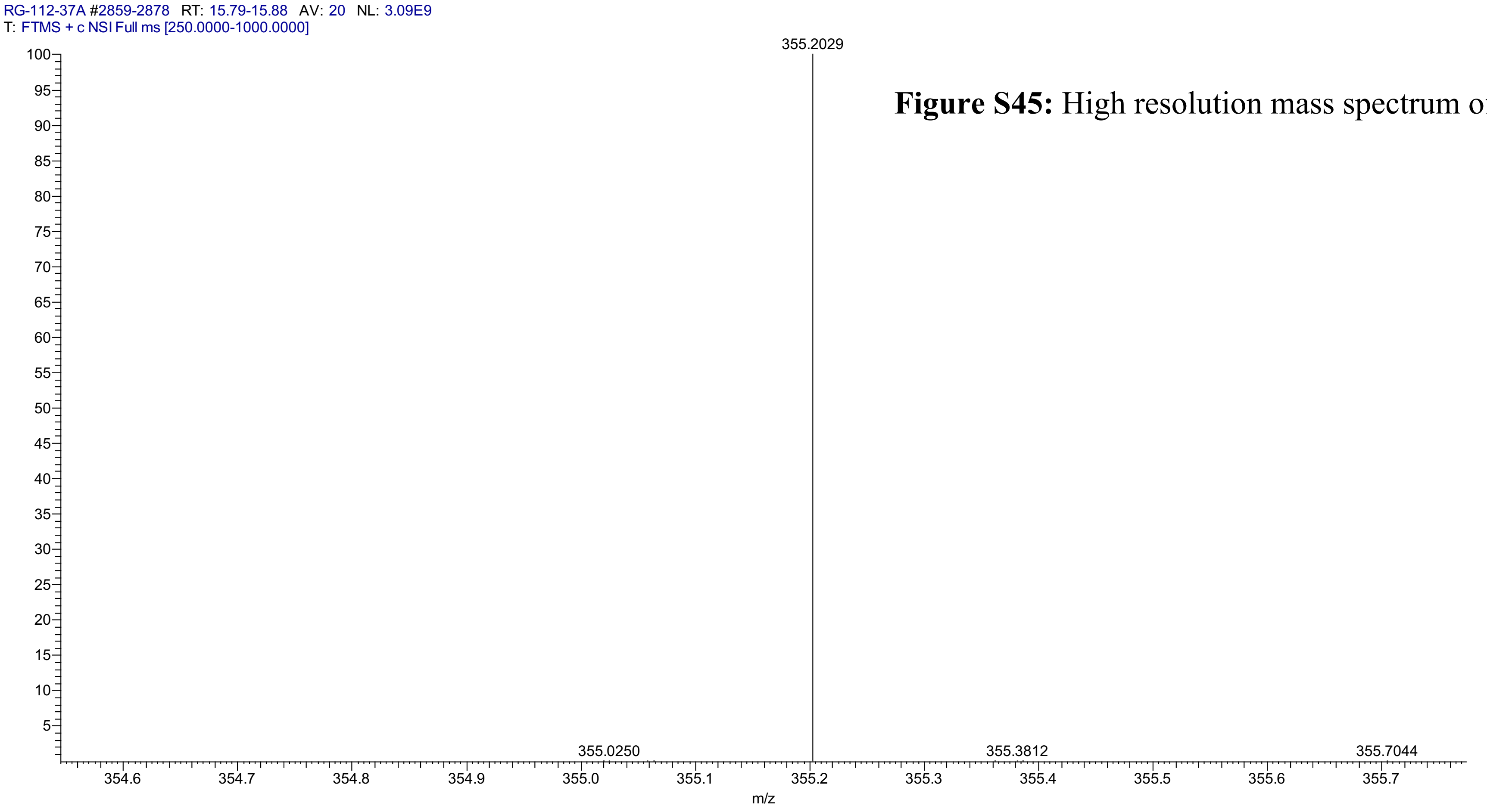

Figure S45: High resolution mass spectrum of **3c**

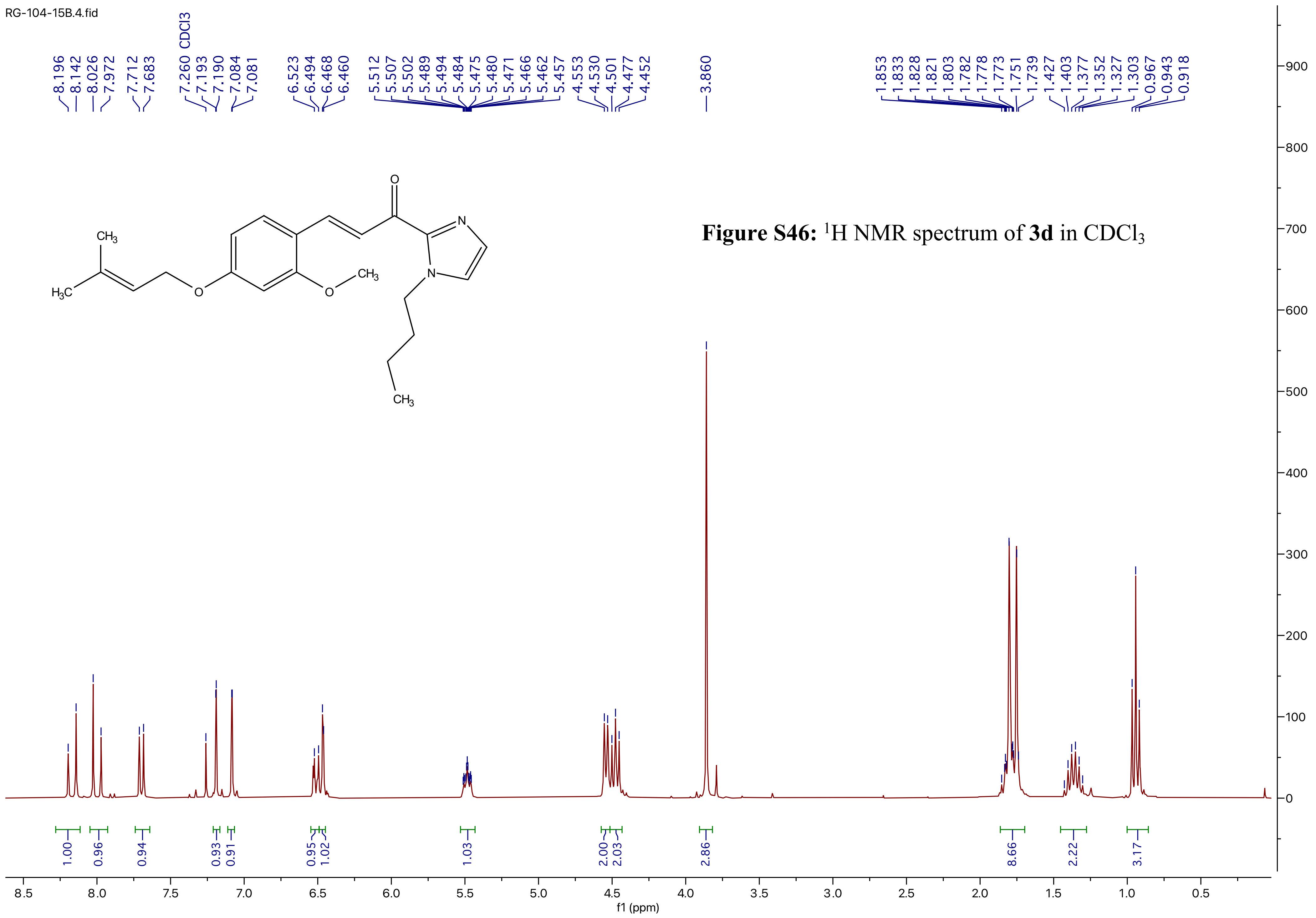

Figure S46: ^{1}H NMR spectrum of **3d** in $CDCl_3$

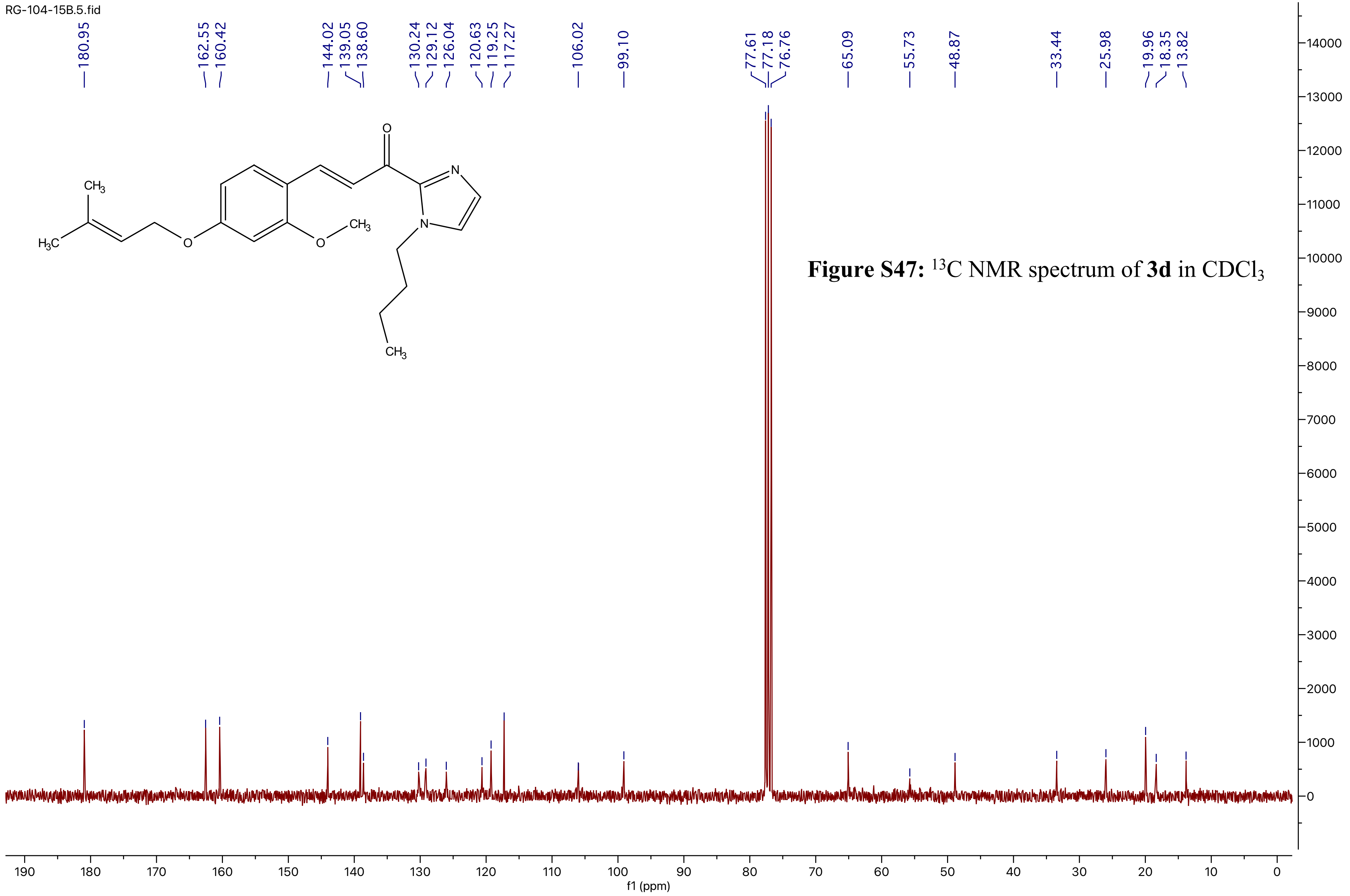

Figure S47: ^{13}C NMR spectrum of **3d** in $CDCl_3$

Smple Name	Mol Fomla	MW	M+H	obsved	dlta	ppm
RG-105-15B	C22H28N2O3	368.21001	369.2178	369.2187	0.0009	2.41

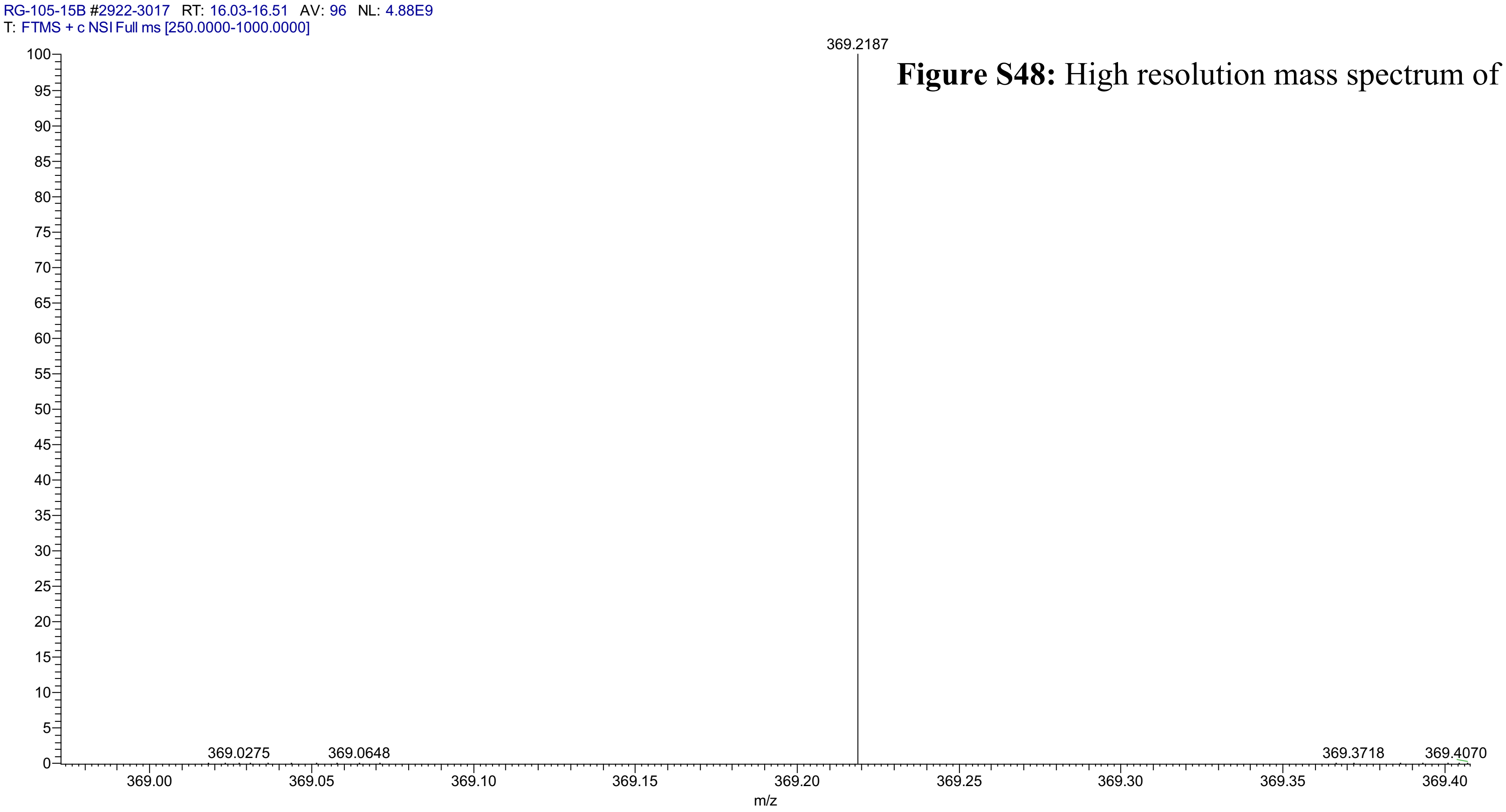

Figure S48: High resolution mass spectrum of **3d**

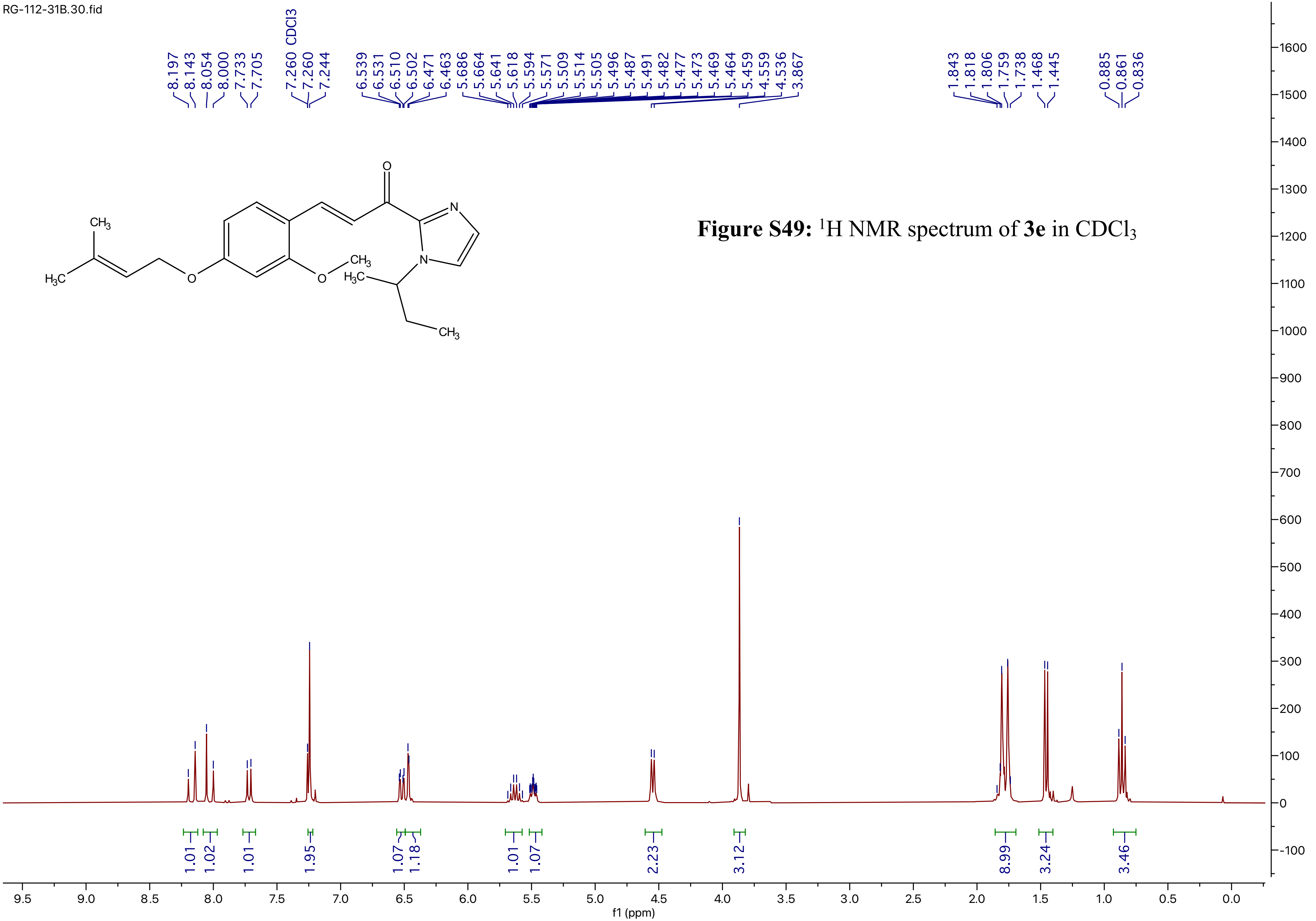

Figure S49: ^{1}H NMR spectrum of **3e** in $CDCl_3$

Smple Name	Mol Fomla	MW	M+H	obsved	dlta	ppm
RG-112-31B	C22H28N2O3	368.21001	369.2178	369.2188	0.0010	2.68

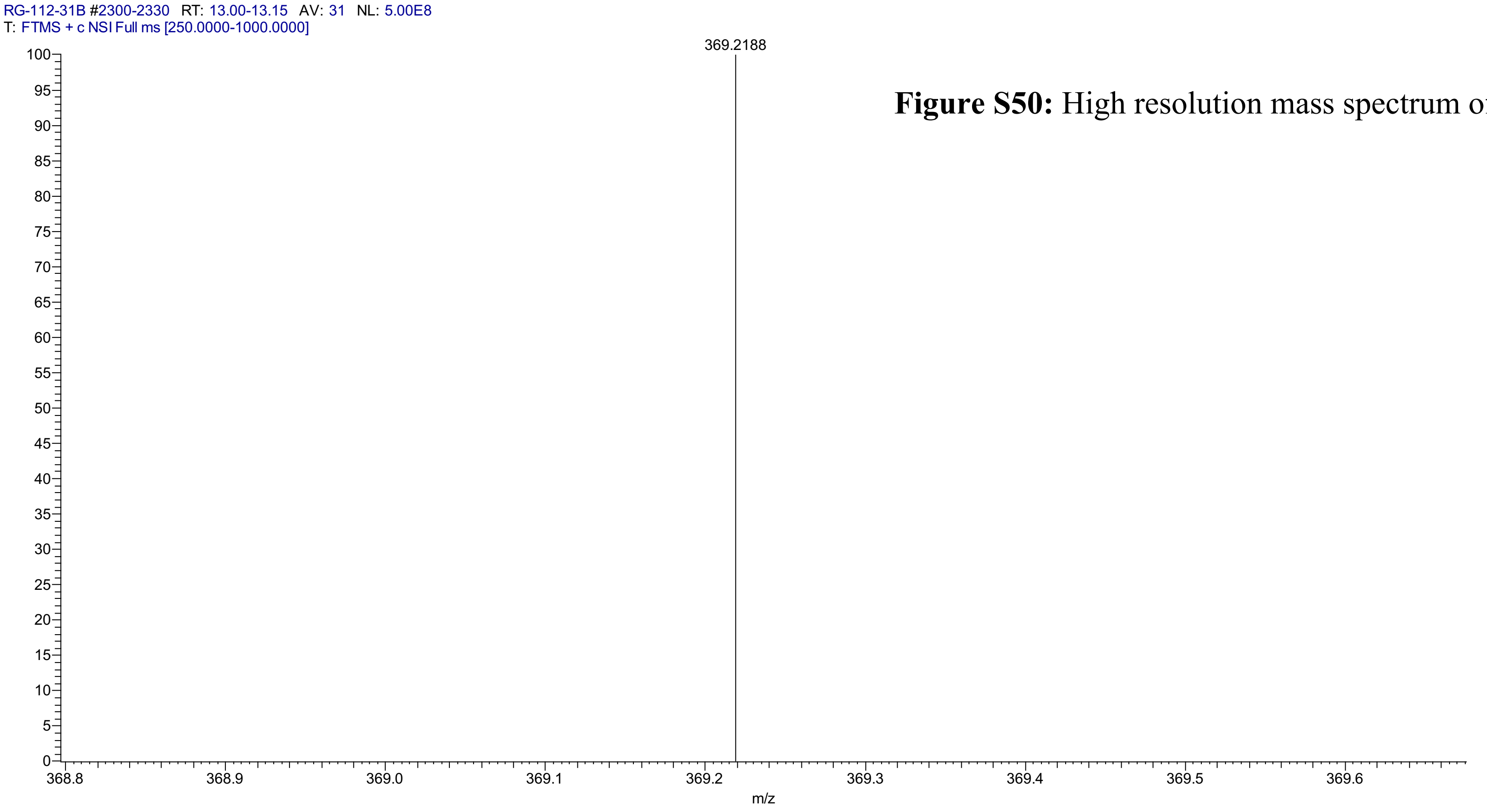

Figure S50: High resolution mass spectrum of **3e**

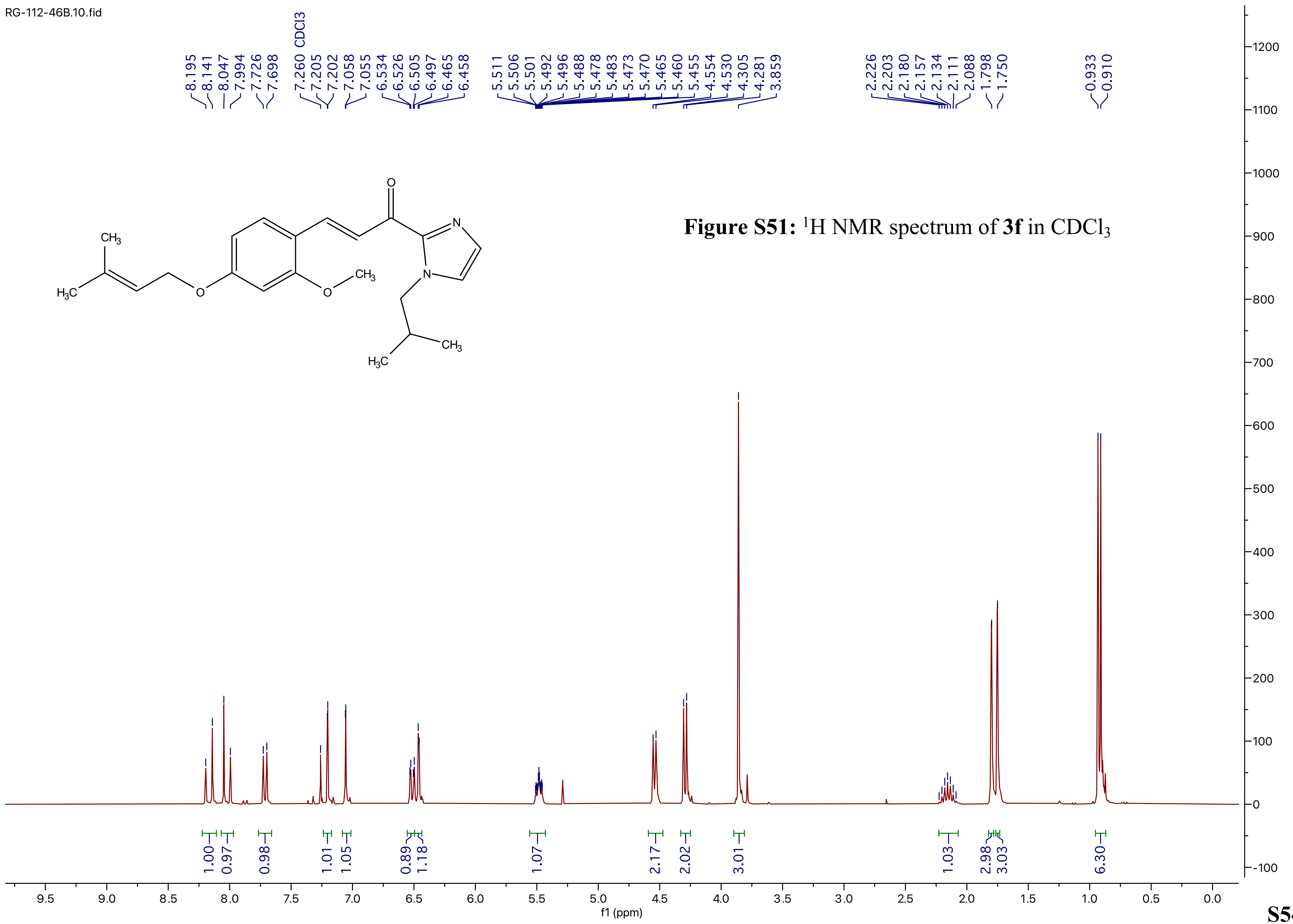

Figure S51: ^{1}H NMR spectrum of **3f** in $CDCl_3$

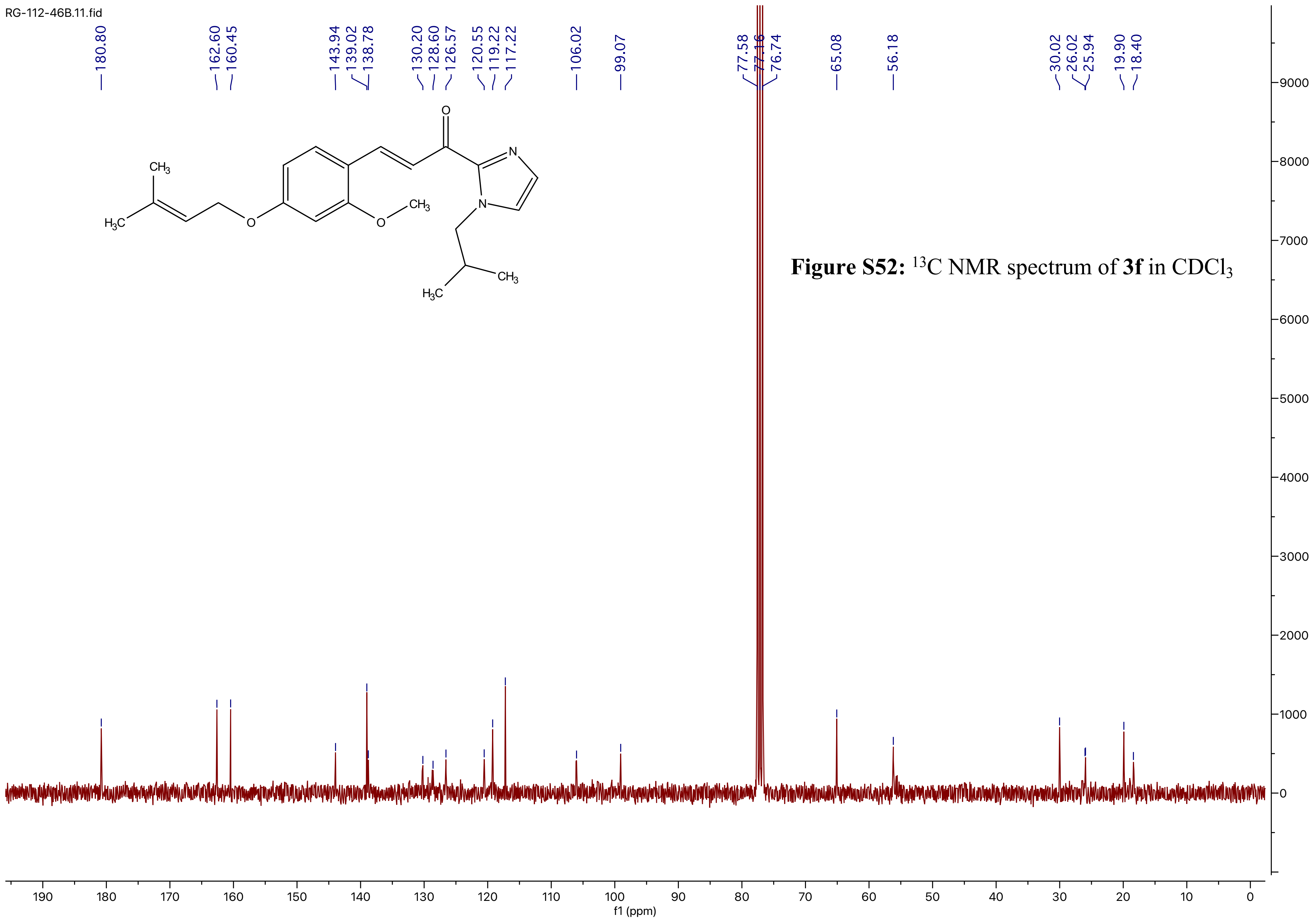

Figure S52: ^{13}C NMR spectrum of **3f** in $CDCl_3$

Smple Name	Mol Fomla	MW	M+H	obsved	dlta	ppm
RG-112-46B	C22H28N2O3	368.21001	369.2178	369.2186	0.0008	2.14

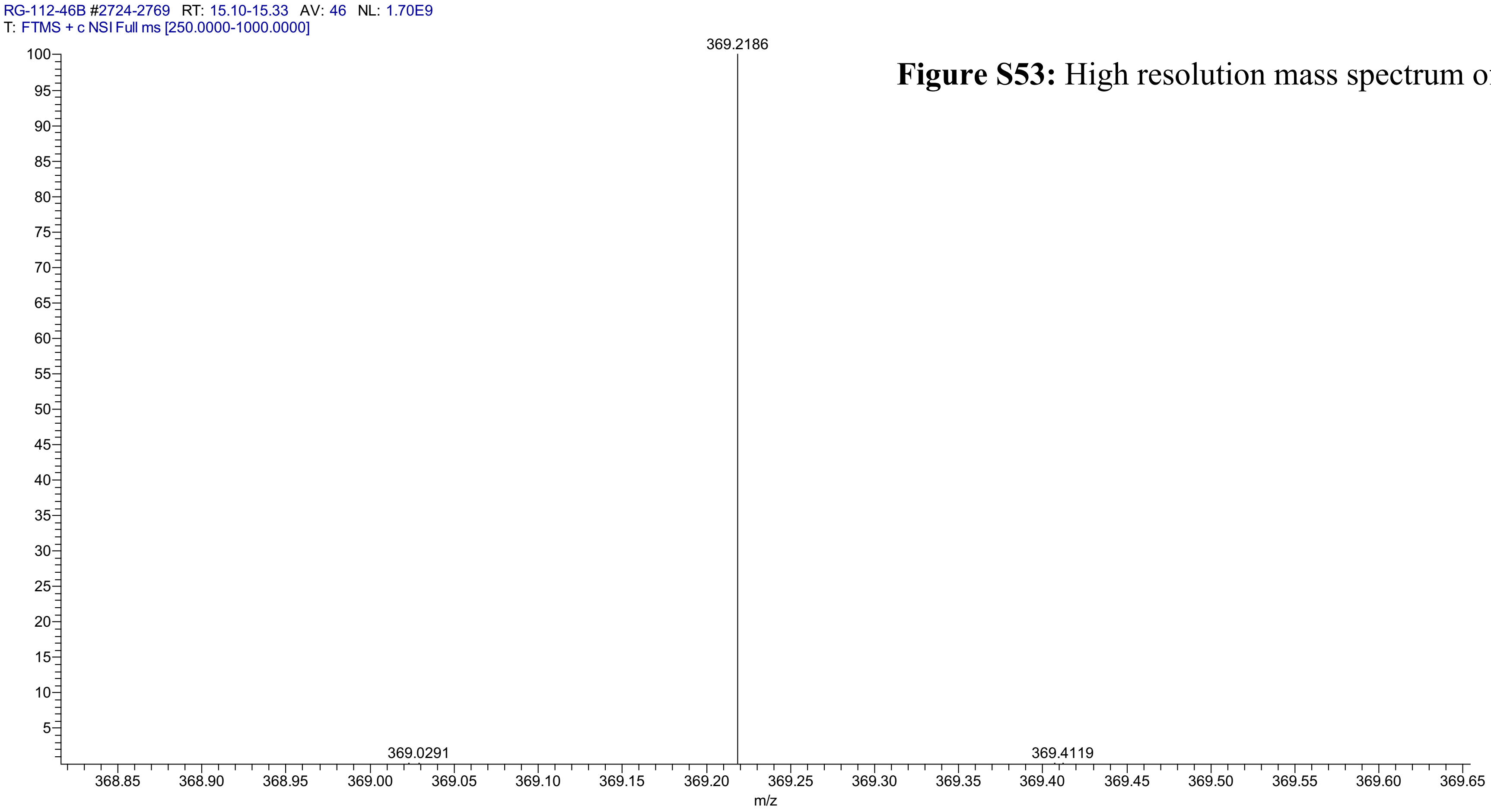

Figure S53: High resolution mass spectrum of **3f**

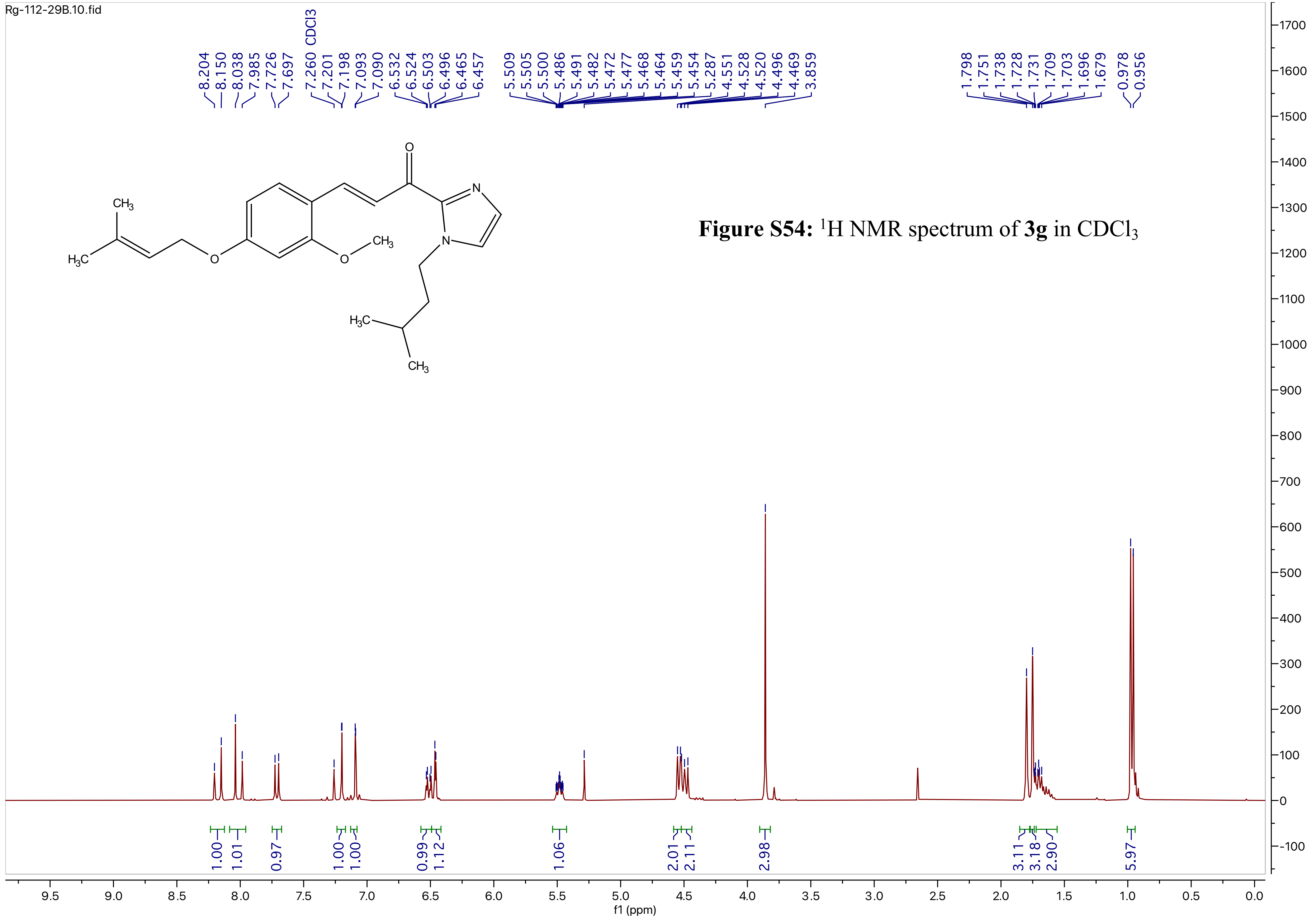

Figure S54: ^{1}H NMR spectrum of **3g** in $CDCl_3$

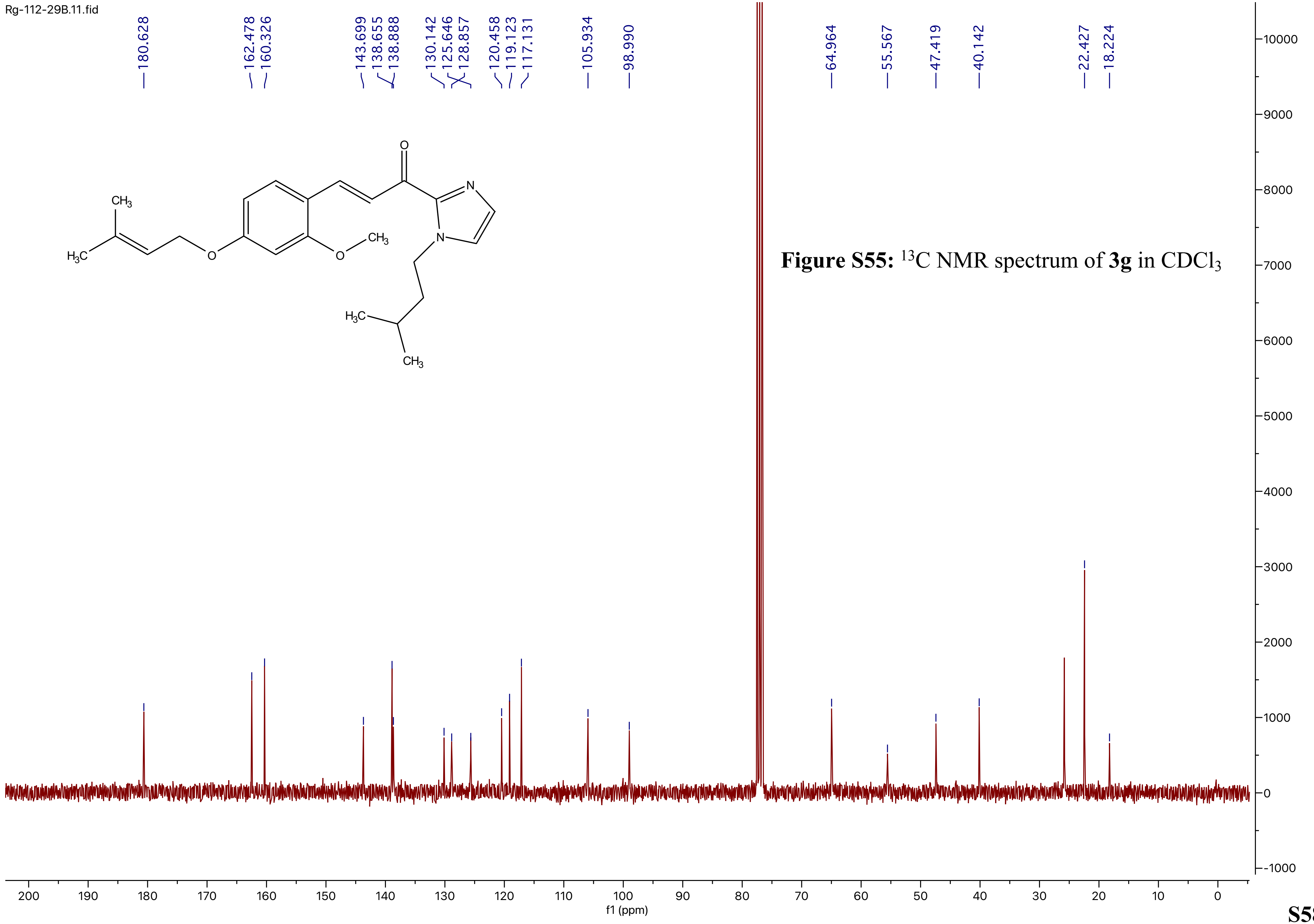

Figure S55: ^{13}C NMR spectrum of **3g** in $CDCl_3$

Smple Name	Mol Fomla	MW	M+H	obsved	dlta	ppm
RG-112-29B	C23H30N2O3	382.22566	383.2335	383.2342	0.0007	1.93

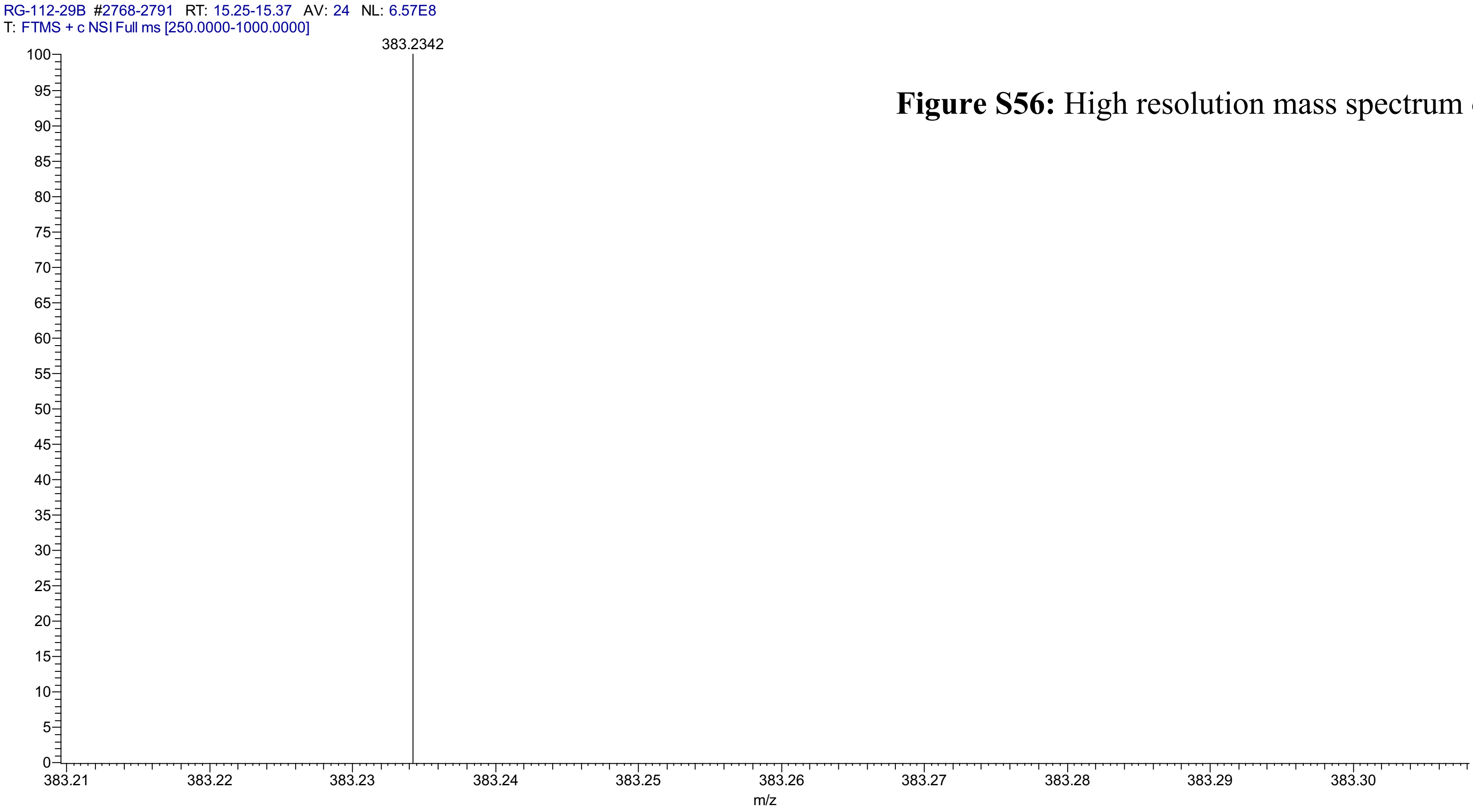

Figure S56: High resolution mass spectrum of **3g**

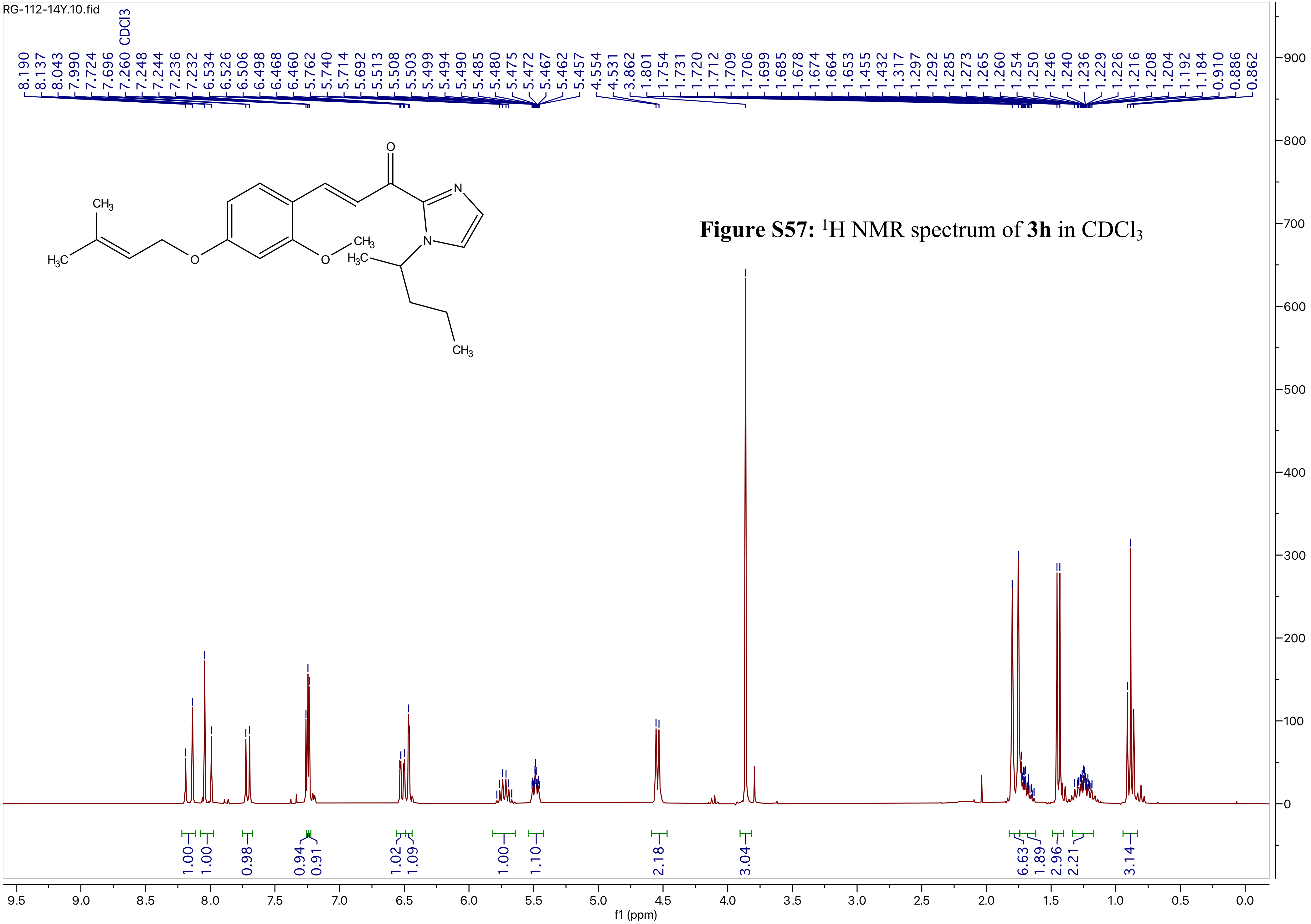

Figure S57: ^1H NMR spectrum of **3h** in $CDCl_3$

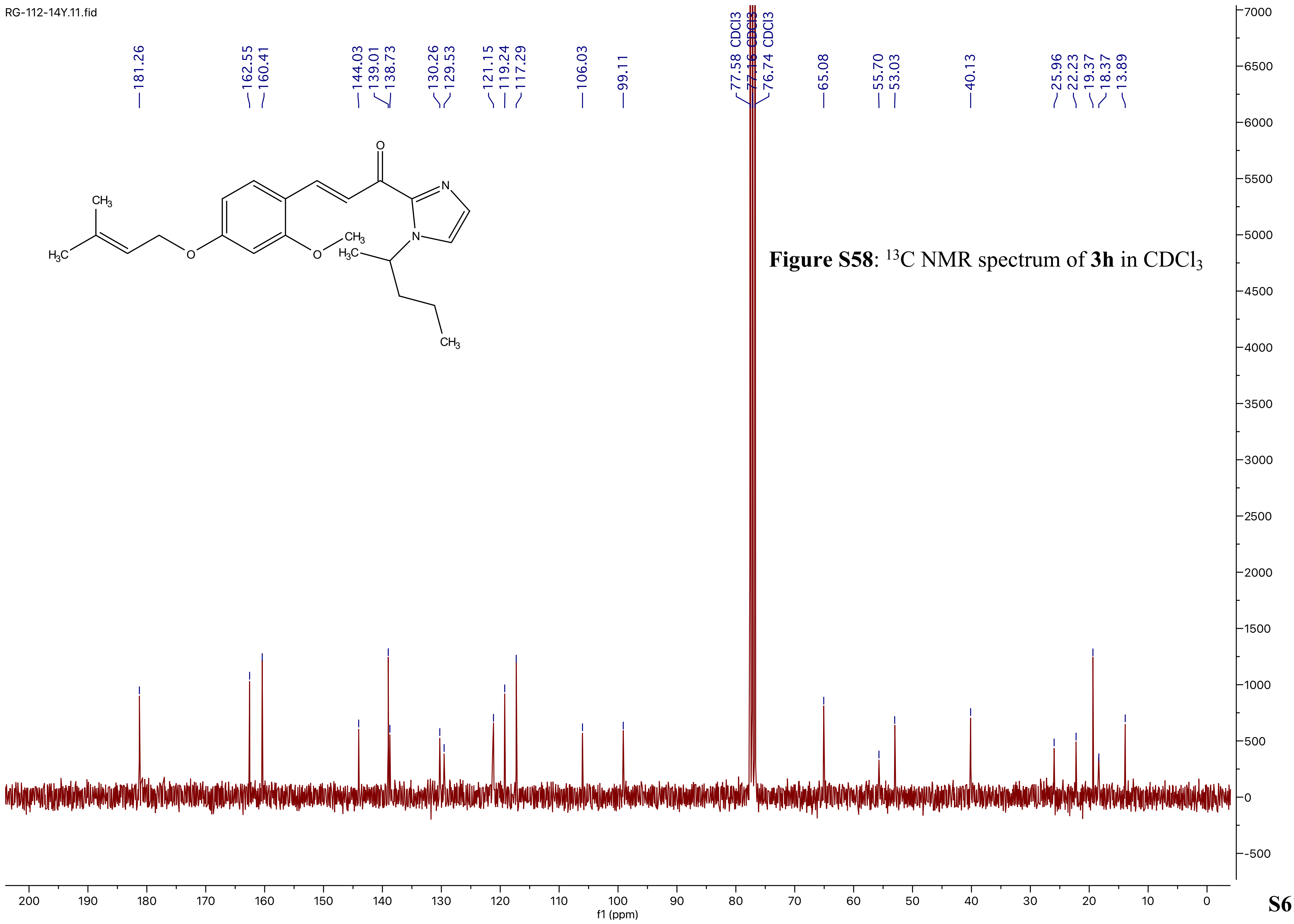

Figure S58: ^{13}C NMR spectrum of **3h** in $CDCl_3$

Smple Name	Mol Fomla	MW	M+H	obsved	dlta	ppm
RG-112-14AY	C23H30N2O3	382.22566	383.2335	383.2344	0.0009	2.45

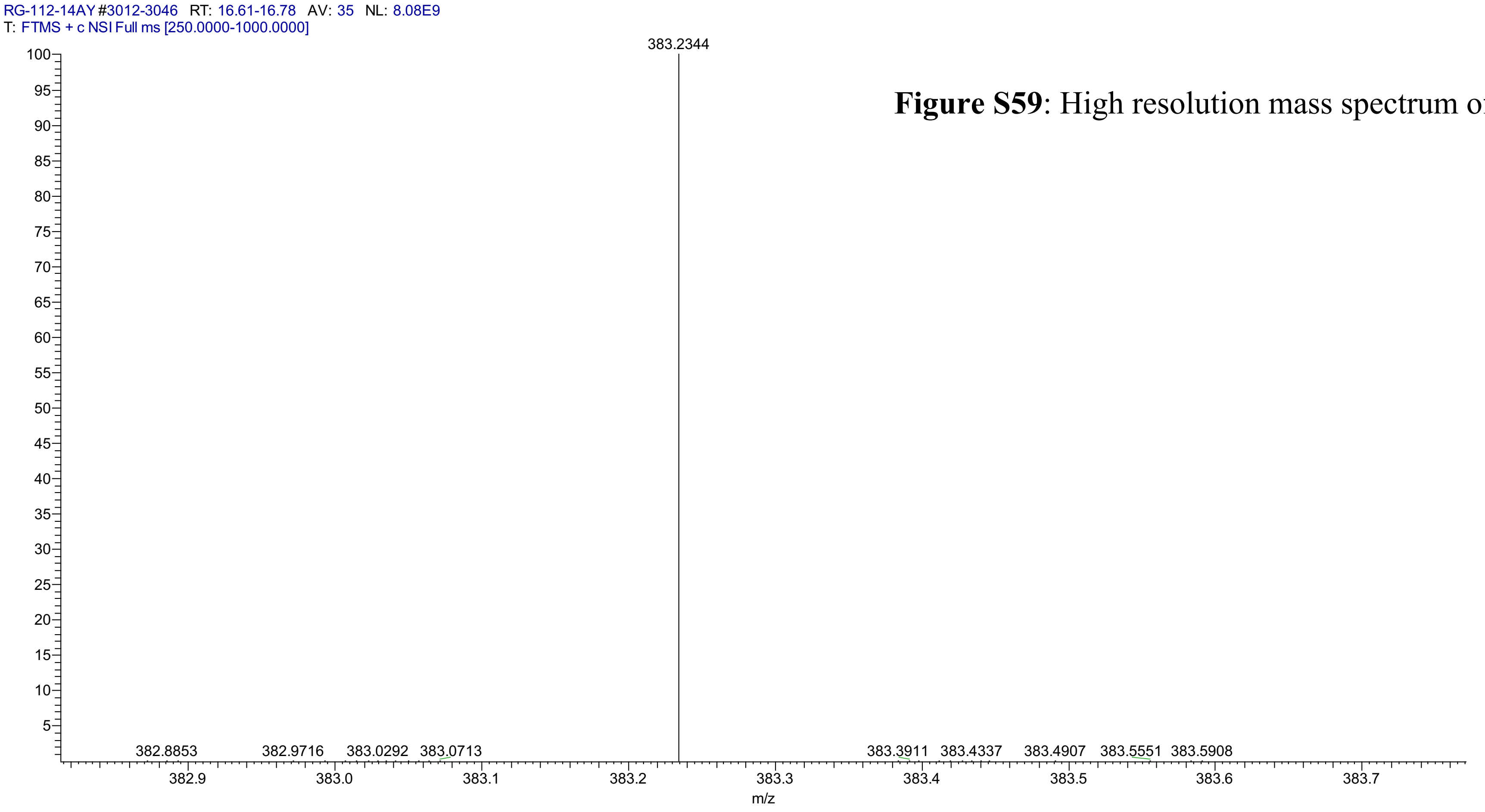

Figure S59: High resolution mass spectrum of **3h**

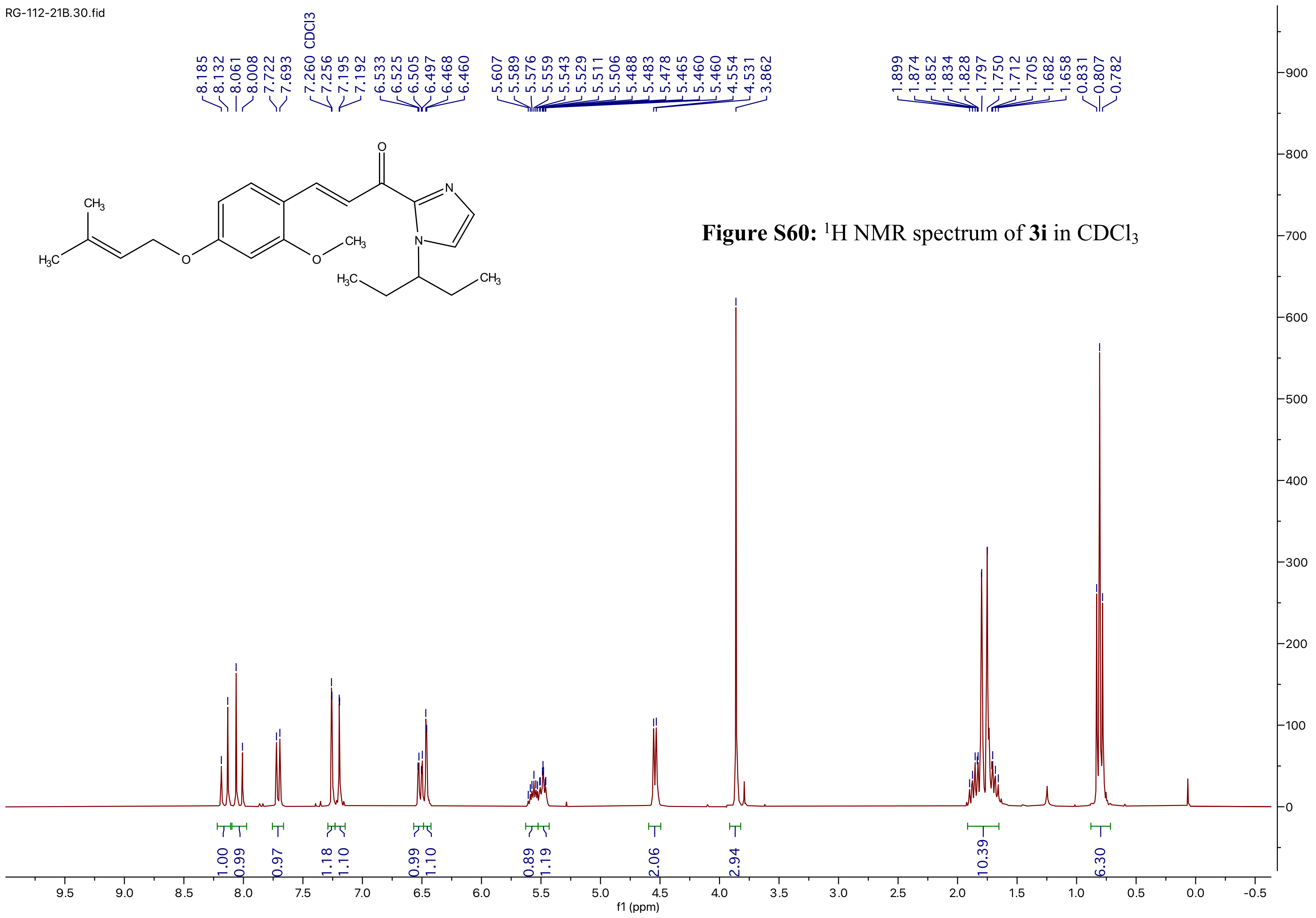

Figure S60: ^{1}H NMR spectrum of **3i** in $CDCl_3$

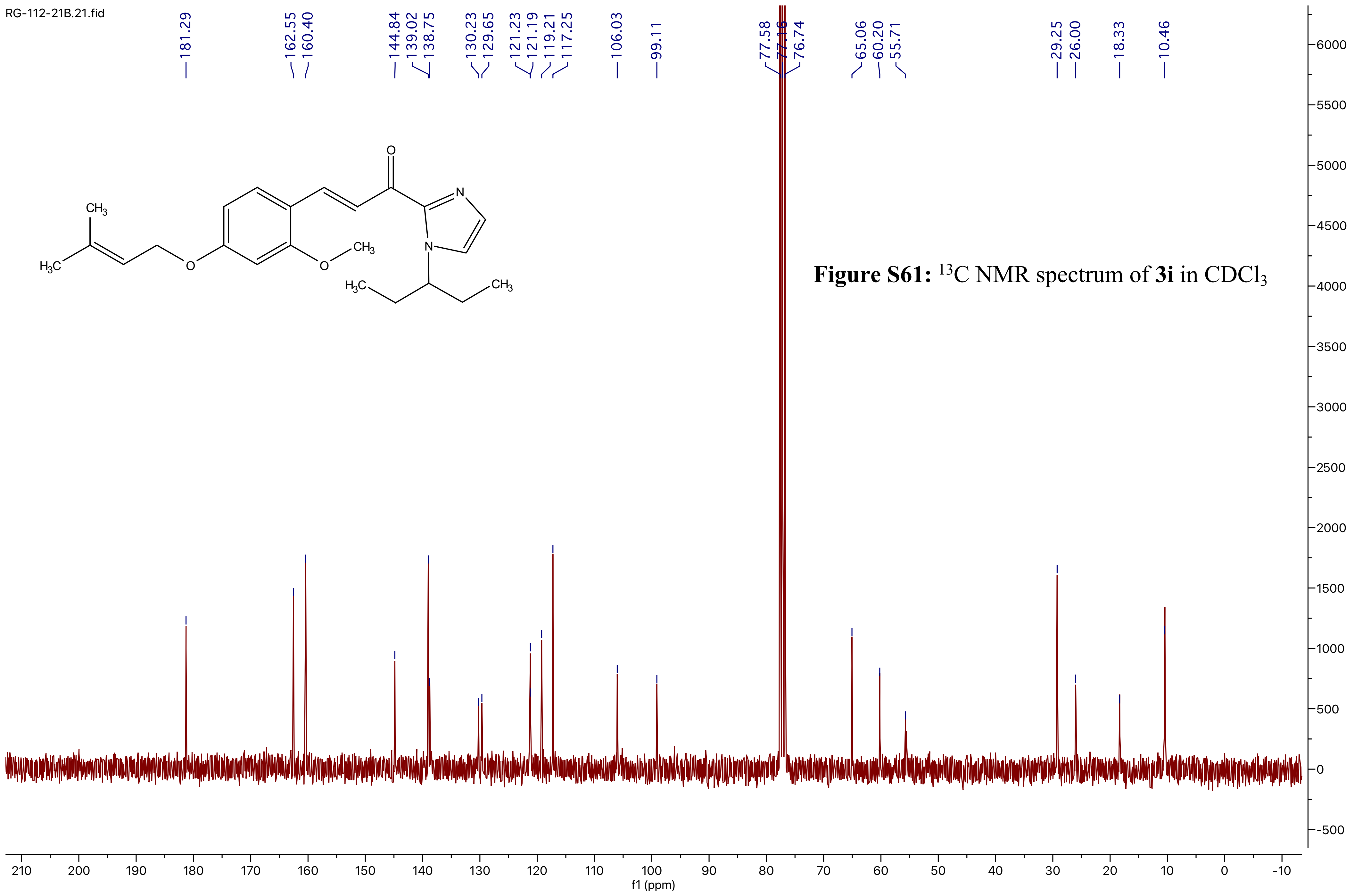

Figure S61: ^{13}C NMR spectrum of **3i** in $CDCl_3$

Smple Name	Mol Fomla	MW	M+H	obsved	dlta	ppm
RG-112-21B	C23H30N2O3	382.22566	383.2335	383.2345	0.0010	2.71

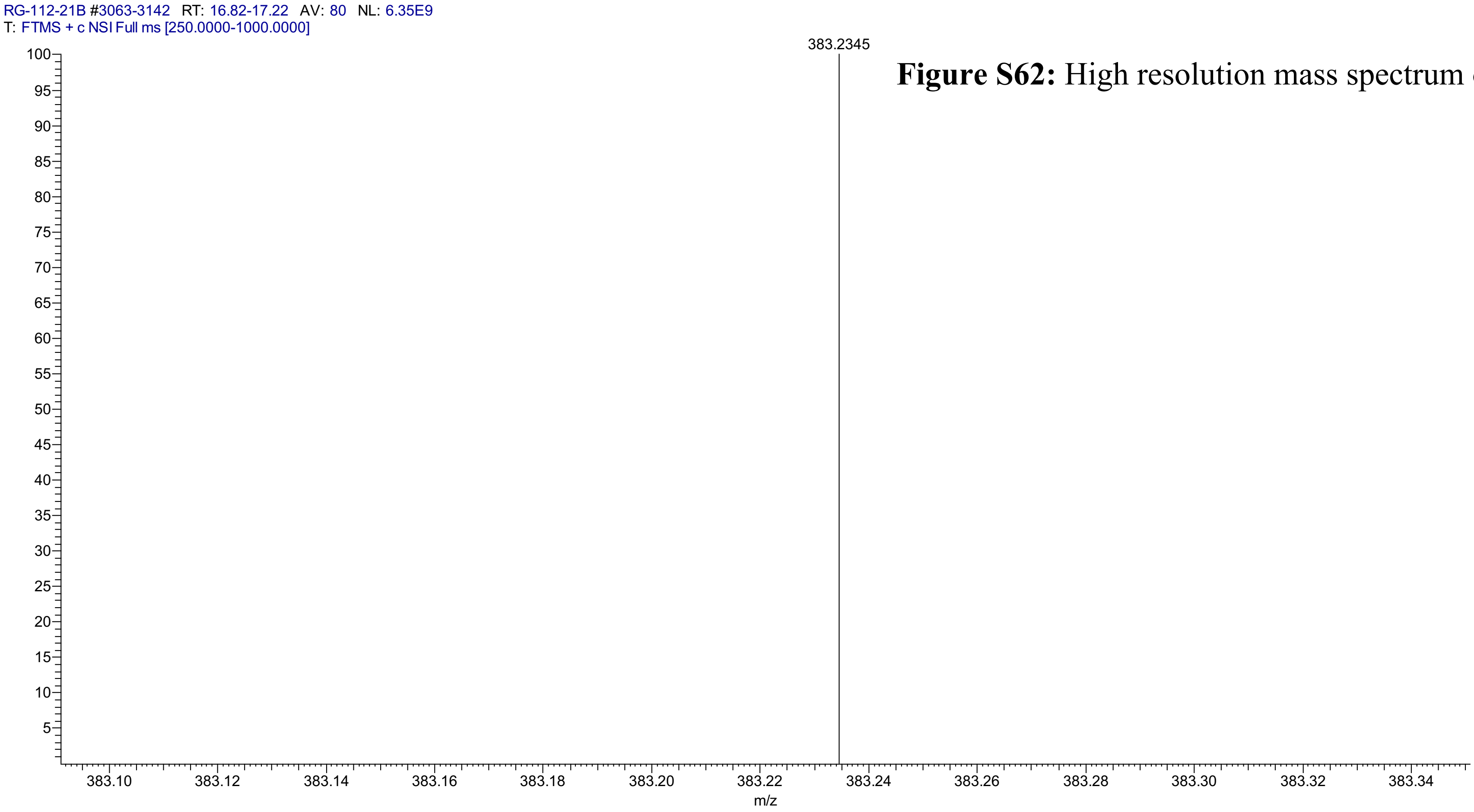

Figure S62: High resolution mass spectrum of **3i**

QGC-96-23

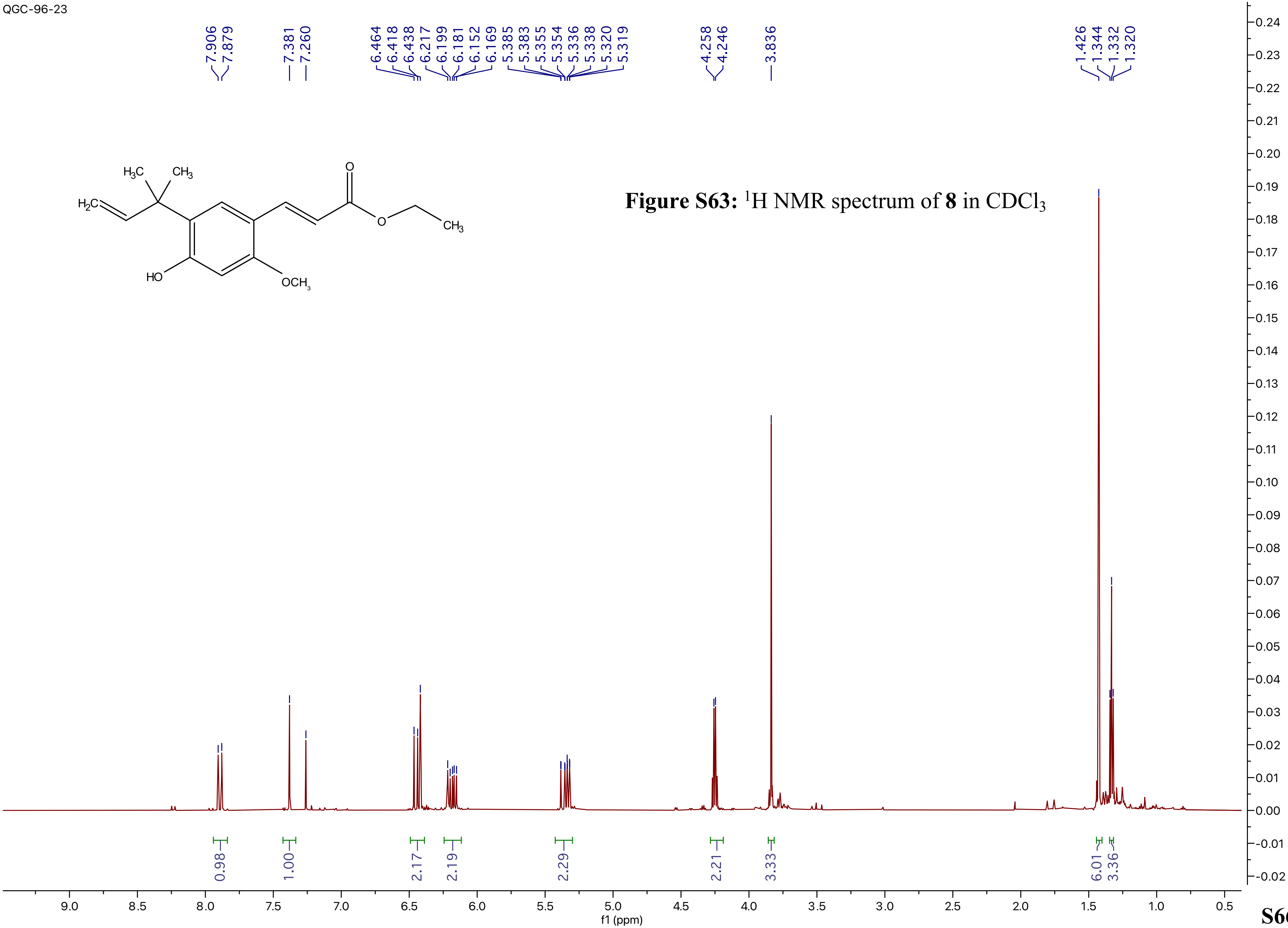

Figure S63: ^{1}H NMR spectrum of **8** in $CDCl_3$

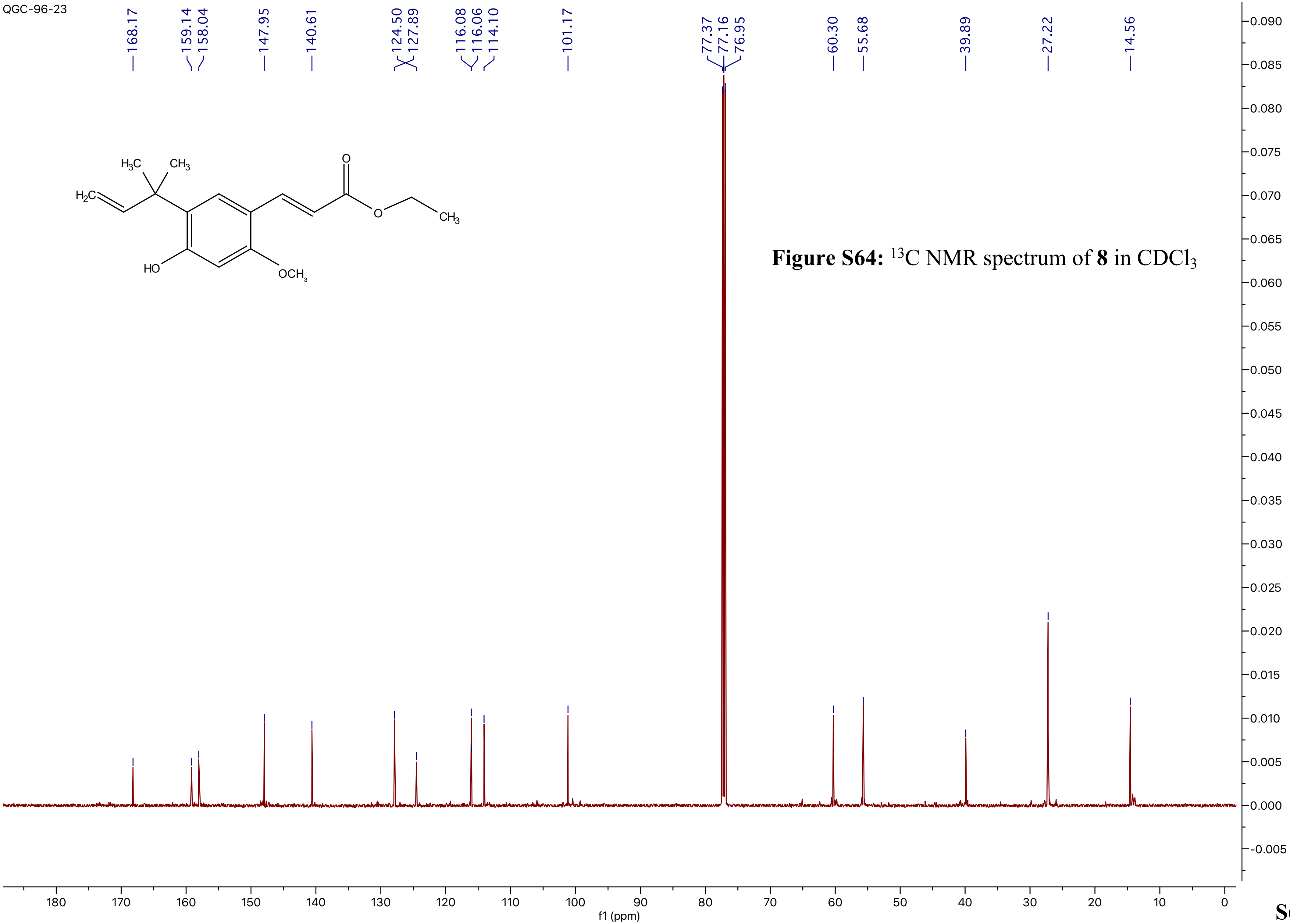

Figure S64: ^{13}C NMR spectrum of **8** in $CDCl_3$

Smple Name	Mol Fomla	MW	M+H	obsved	dlta	ppm
QGC-96-23	C17H22O4	290.1518	291.1596	291.1593	-0.0003	-1.03

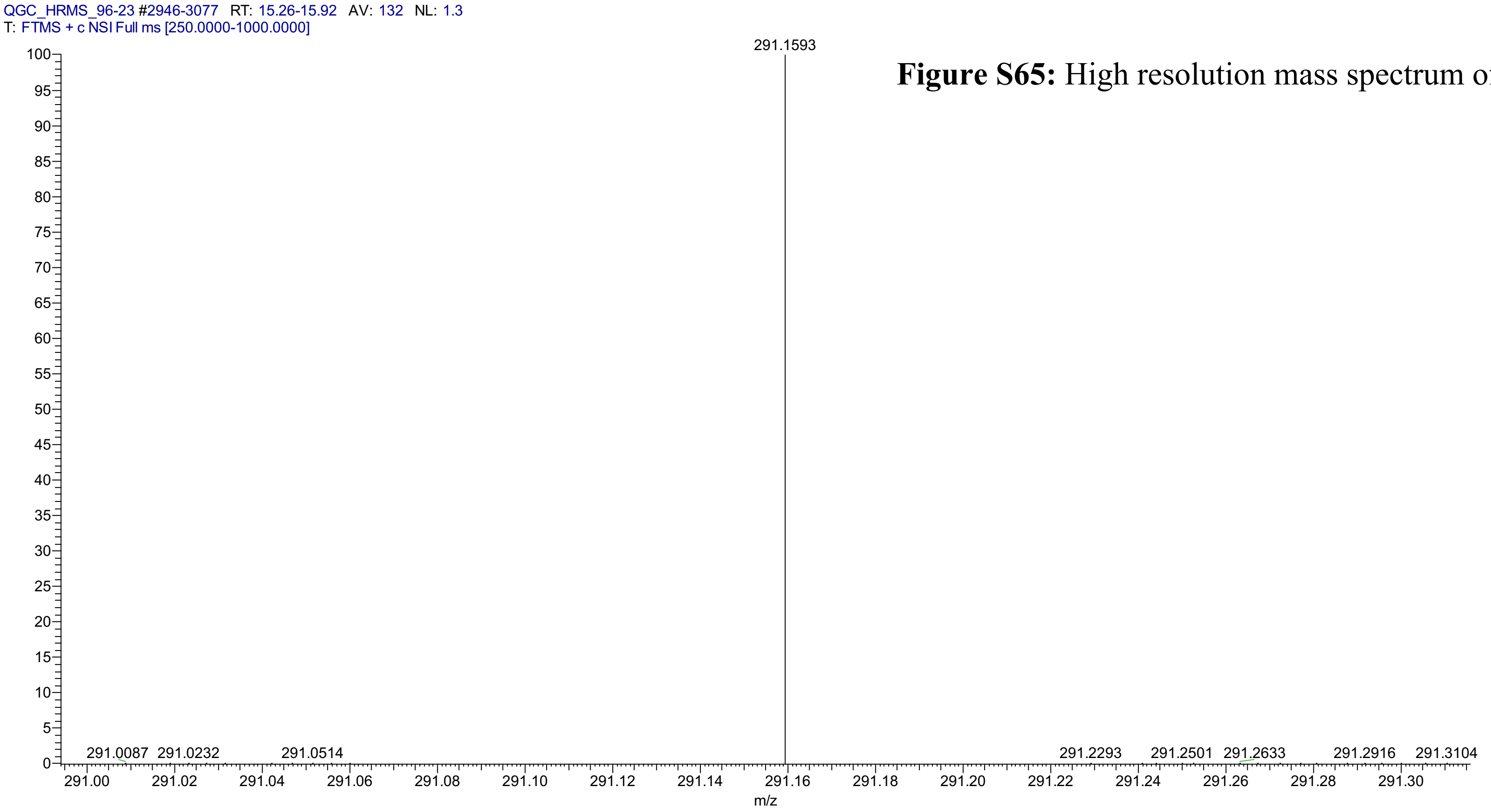

Figure S65: High resolution mass spectrum of **8**

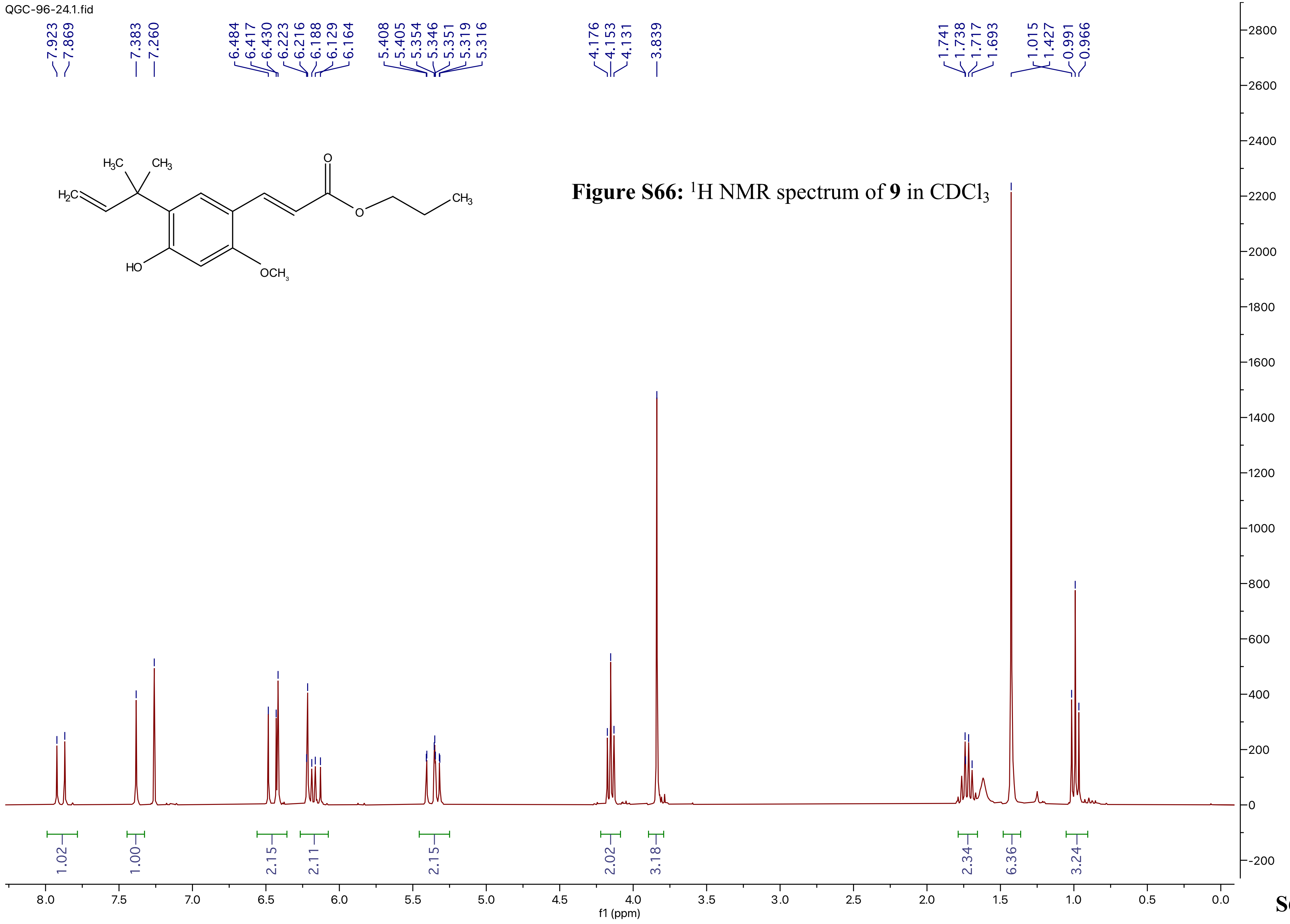

Figure S66: ^{1}H NMR spectrum of **9** in $CDCl_3$

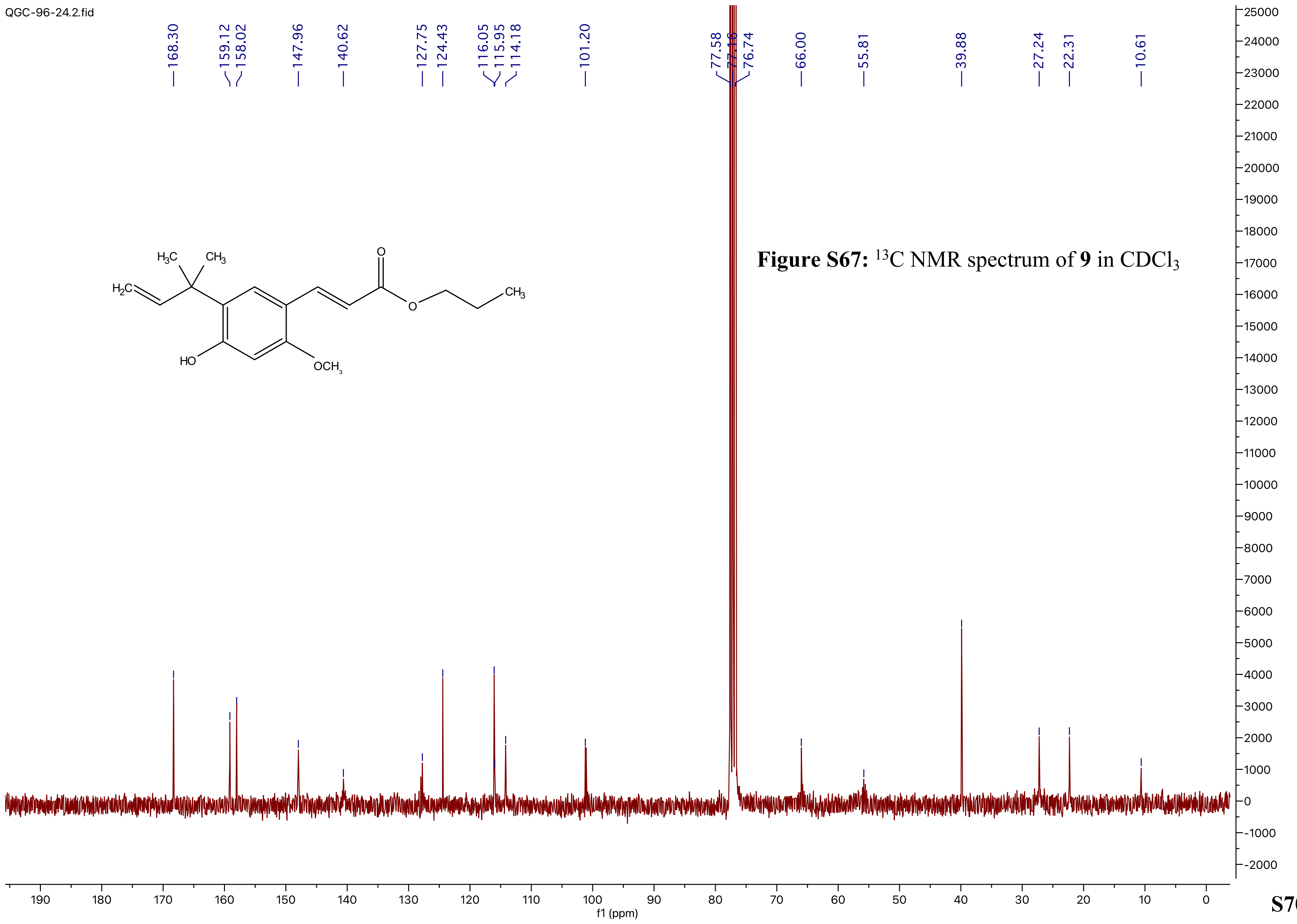

Figure S67: ^{13}C NMR spectrum of **9** in $CDCl_3$

Smple Name	Mol Fomla	MW	M+H	obsved	dlta	ppm
QGC-96-24	C18H24O4	304.1675	305.1753	305.1749	-0.0004	-1.31

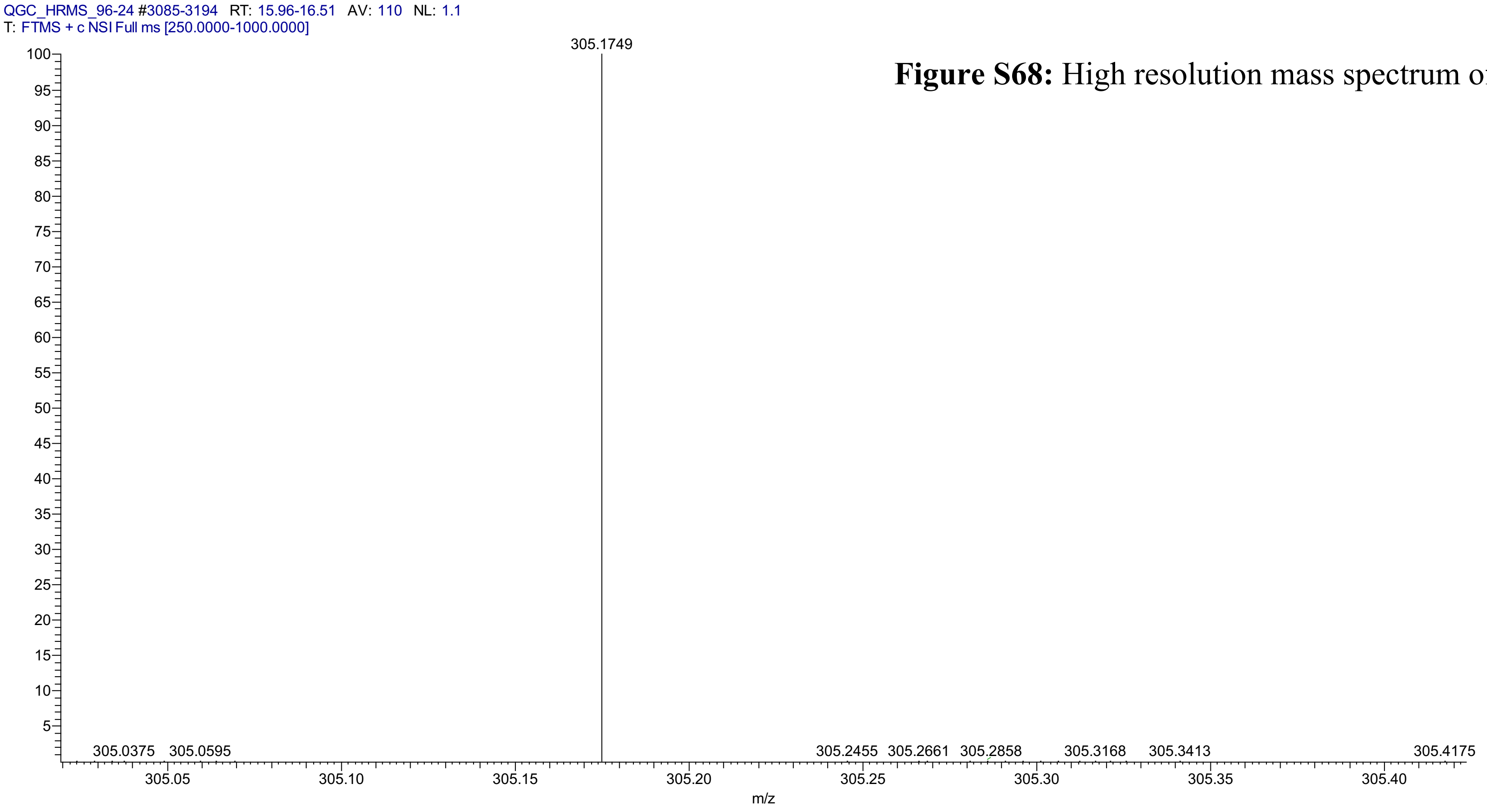

Figure S68: High resolution mass spectrum of **9**

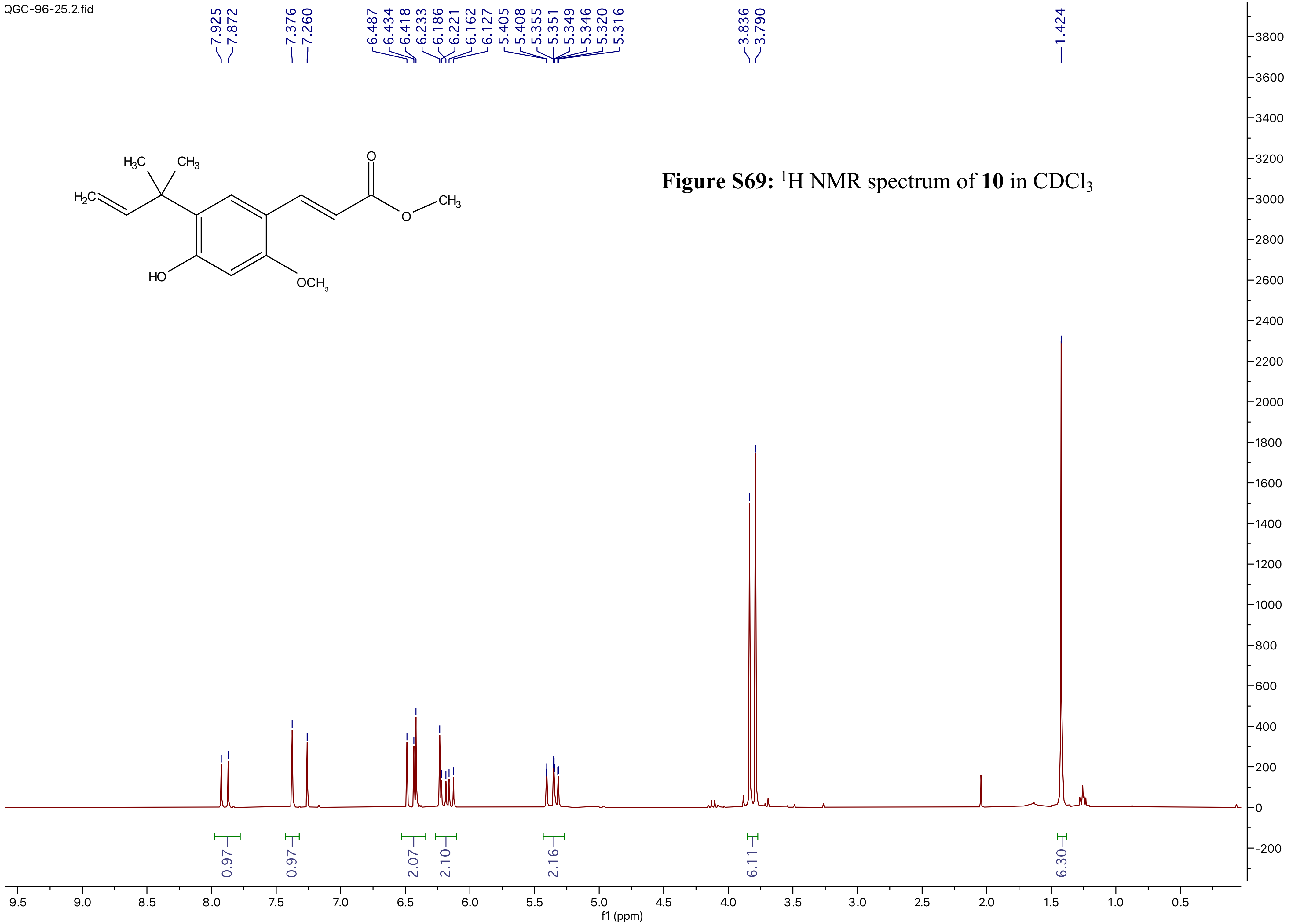

Figure S69: ^{1}H NMR spectrum of **10** in $CDCl_3$

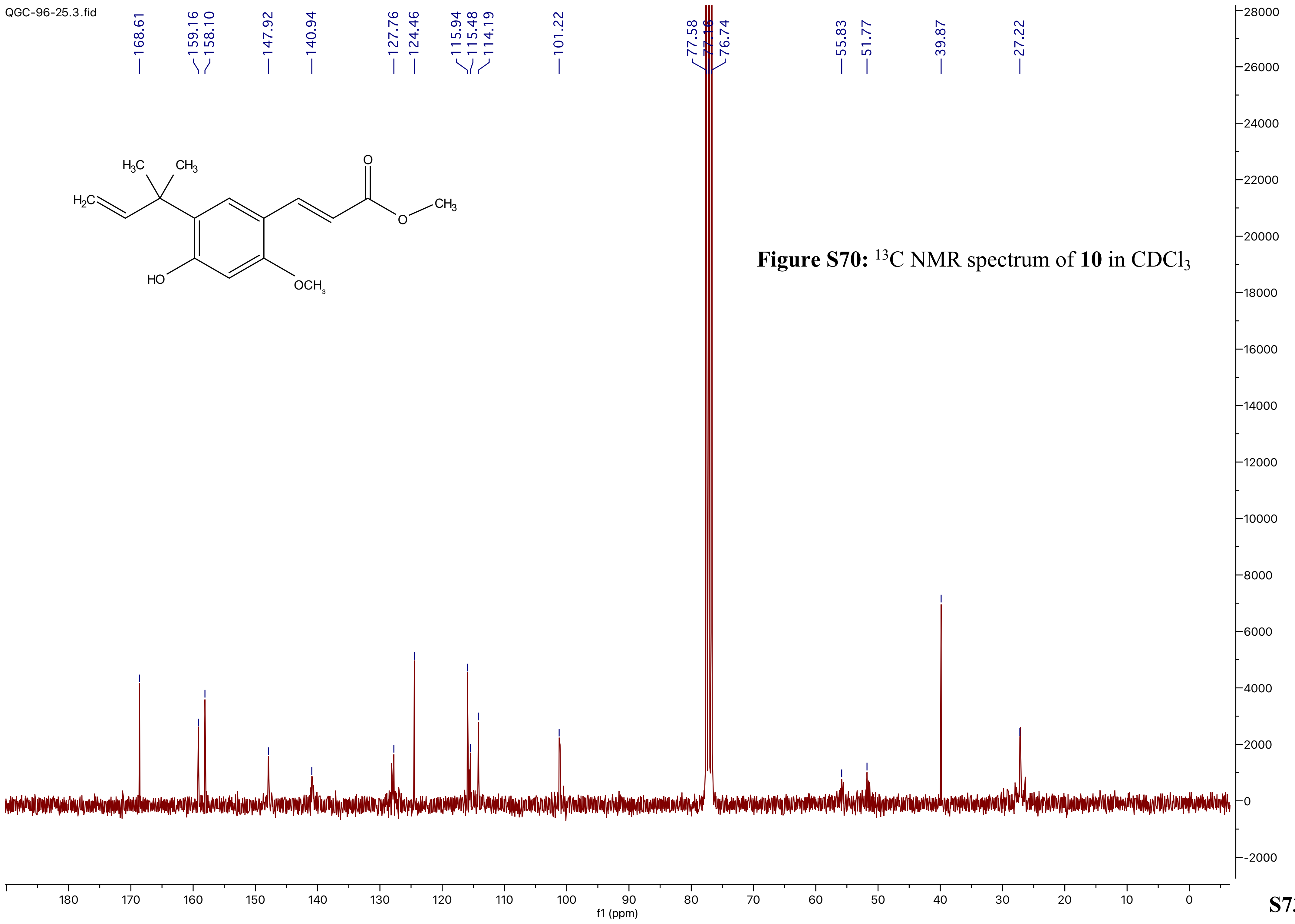

Figure S70: ^{13}C NMR spectrum of **10** in $CDCl_3$

Smple Name	Mol Fomla	MW	M+H	obsved	dlta	ppm
QGC-96-25	C16H20O4	276.1362	277.1440	277.1435	-0.0005	-1.80

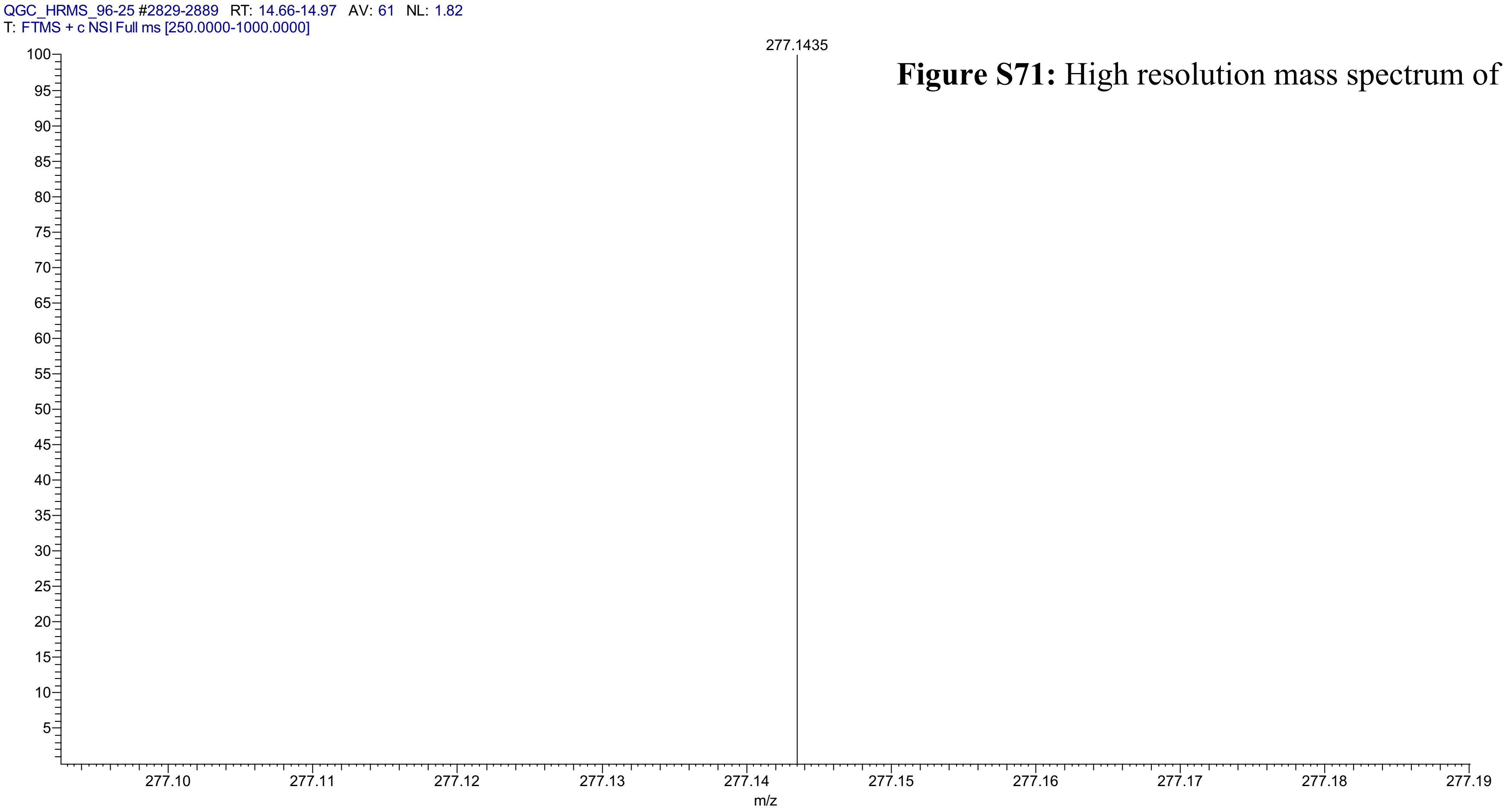

Figure S71: High resolution mass spectrum of **10**

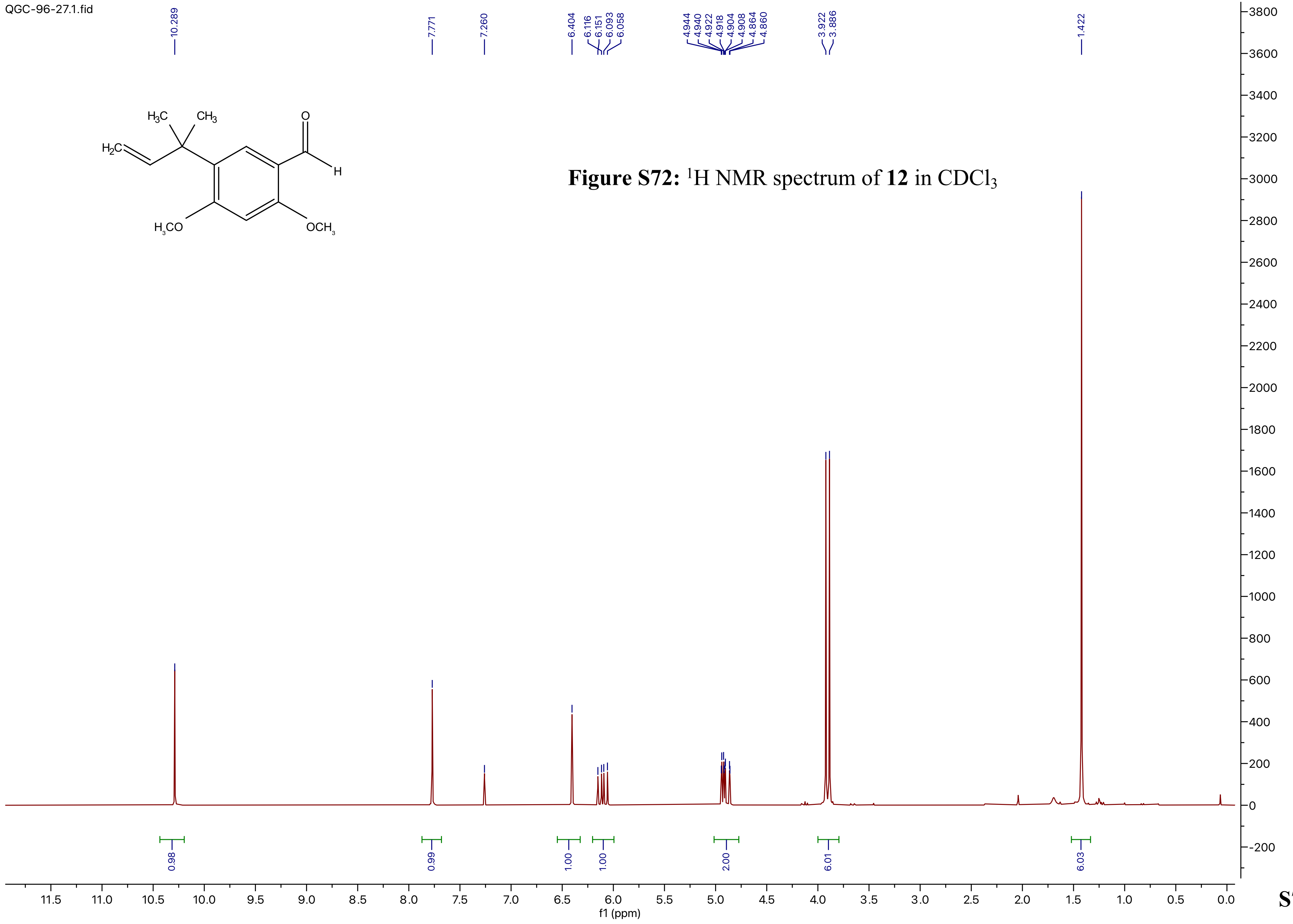

Figure S72: ^{1}H NMR spectrum of **12** in $CDCl_3$

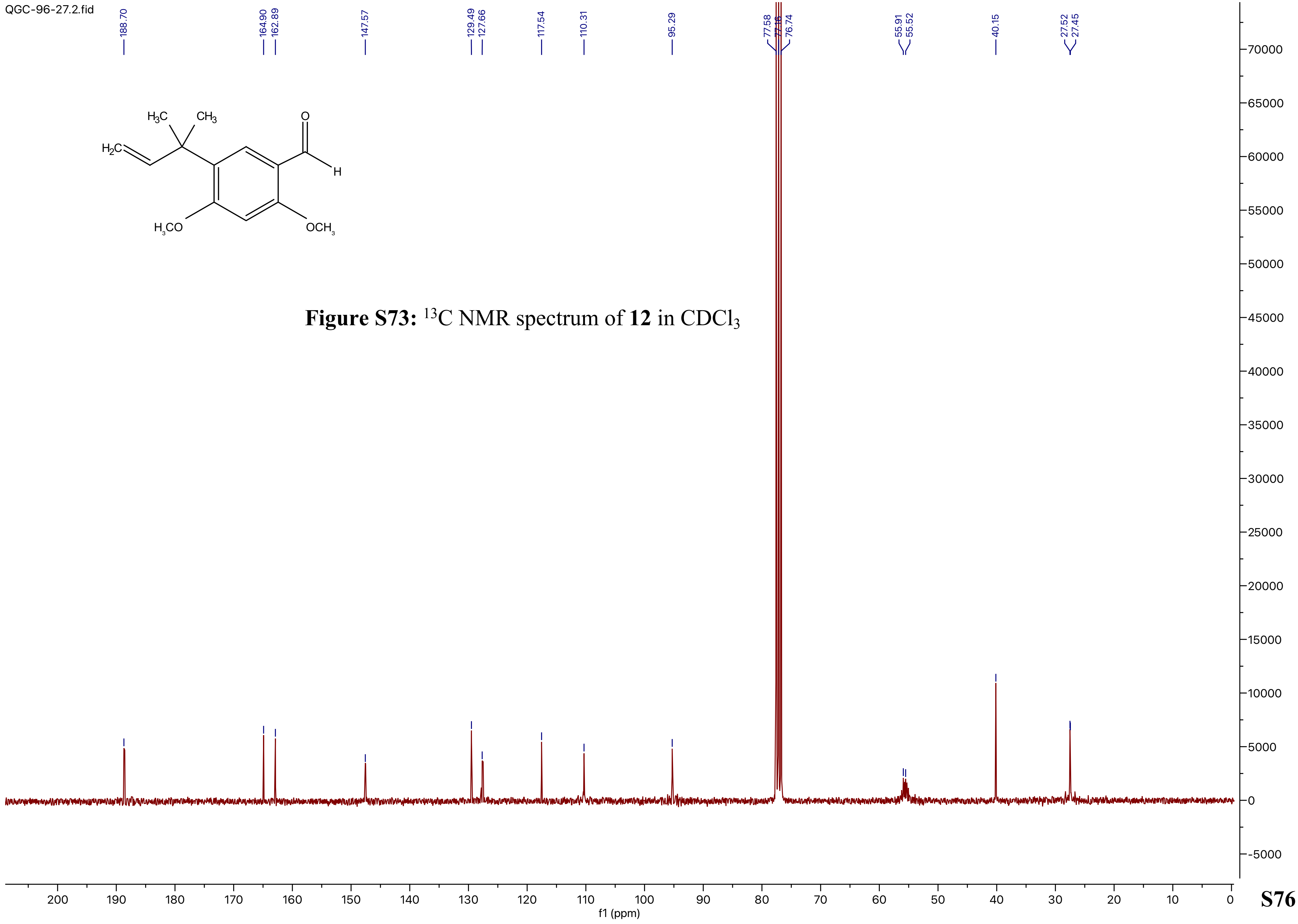

Figure S73: ^{13}C NMR spectrum of **12** in $CDCl_3$

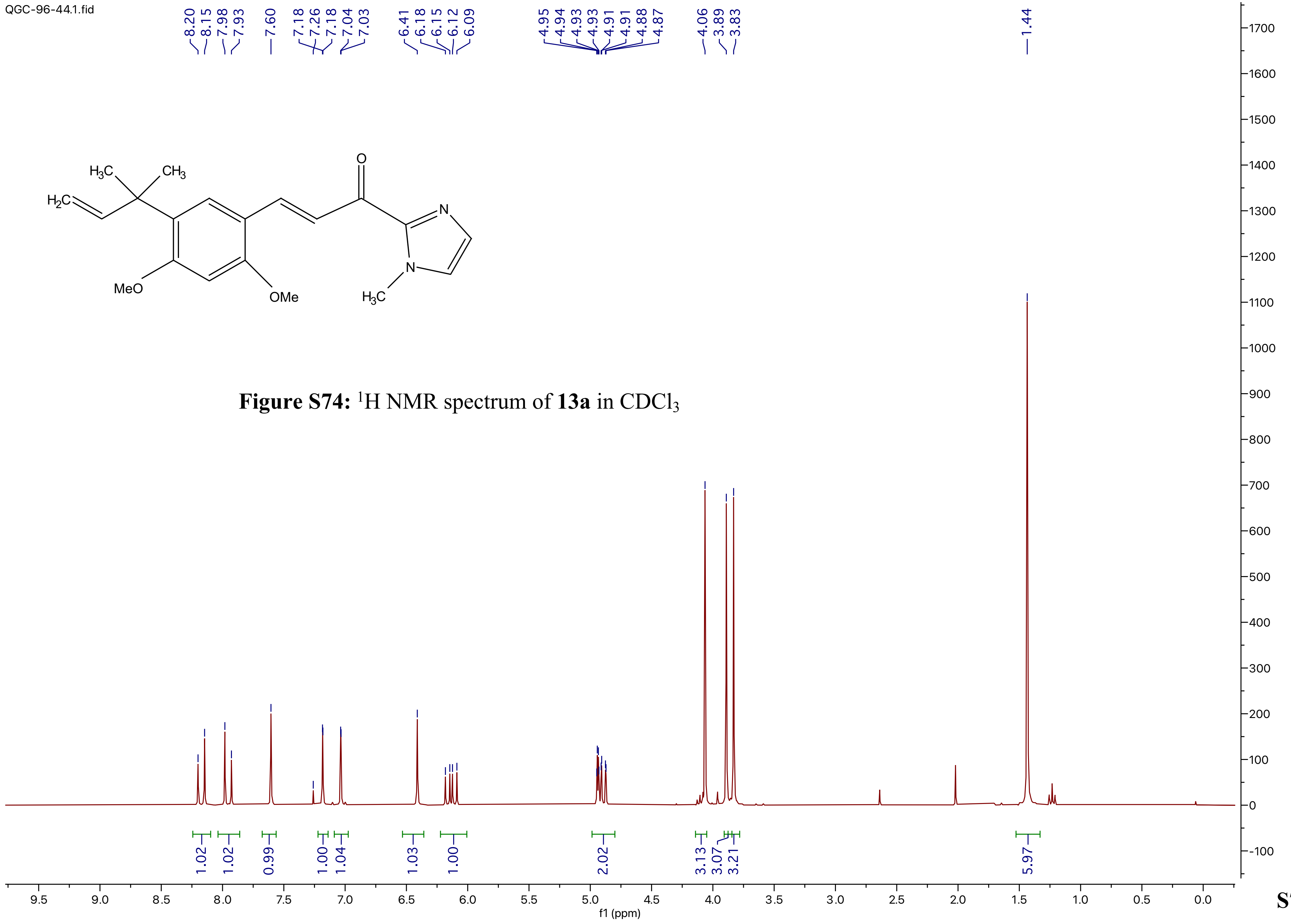

Figure S74: ^{1}H NMR spectrum of **13a** in $CDCl_3$

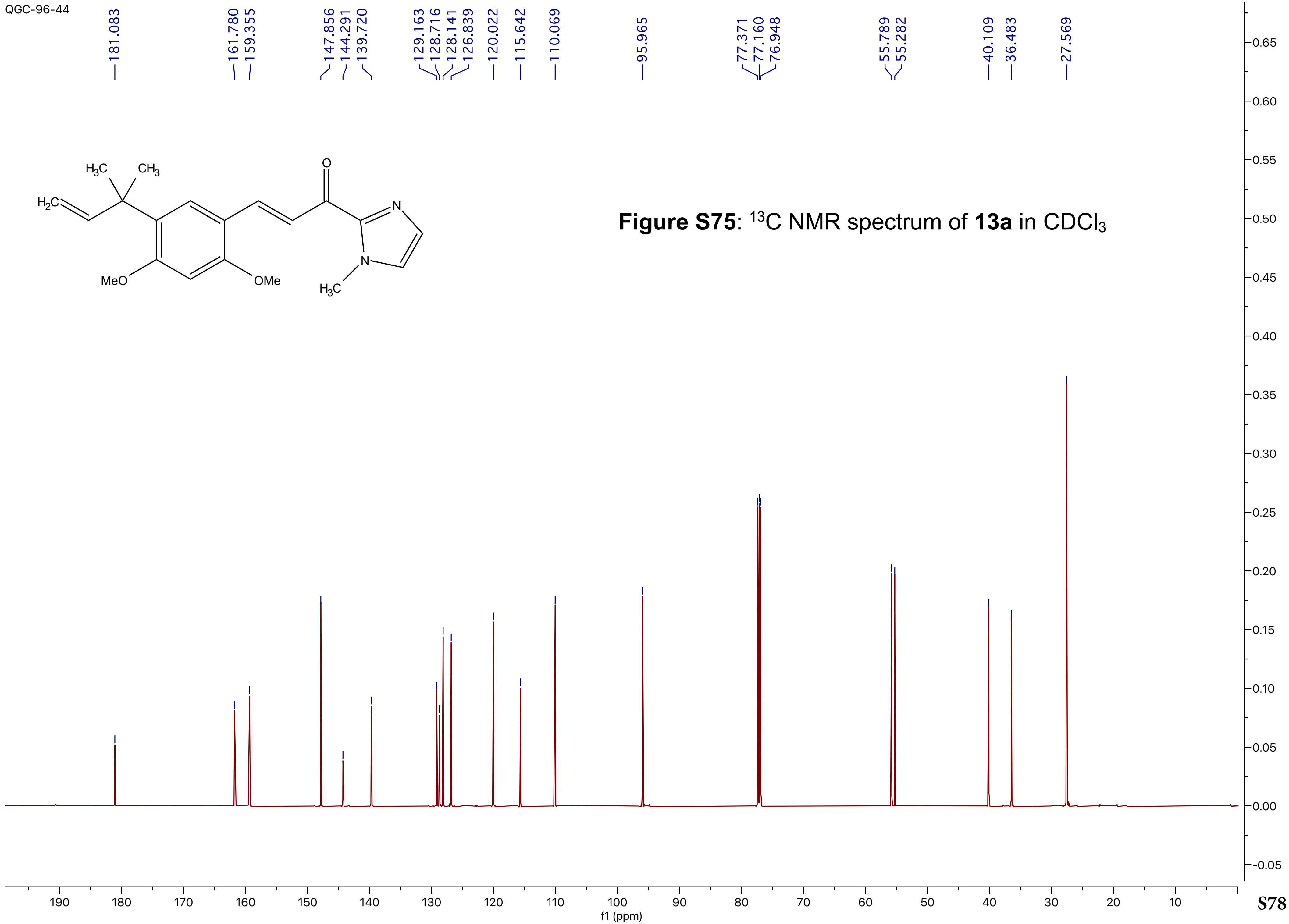

Figure S75: ^{13}C NMR spectrum of **13a** in $CDCl_3$

Smple Name	Mol Fomla	MW	M+H	obsved	dlta	ppm
QGC-96-44	C20H24N2O3	340.1787	341.1865	341.1870	0.0005	1.47

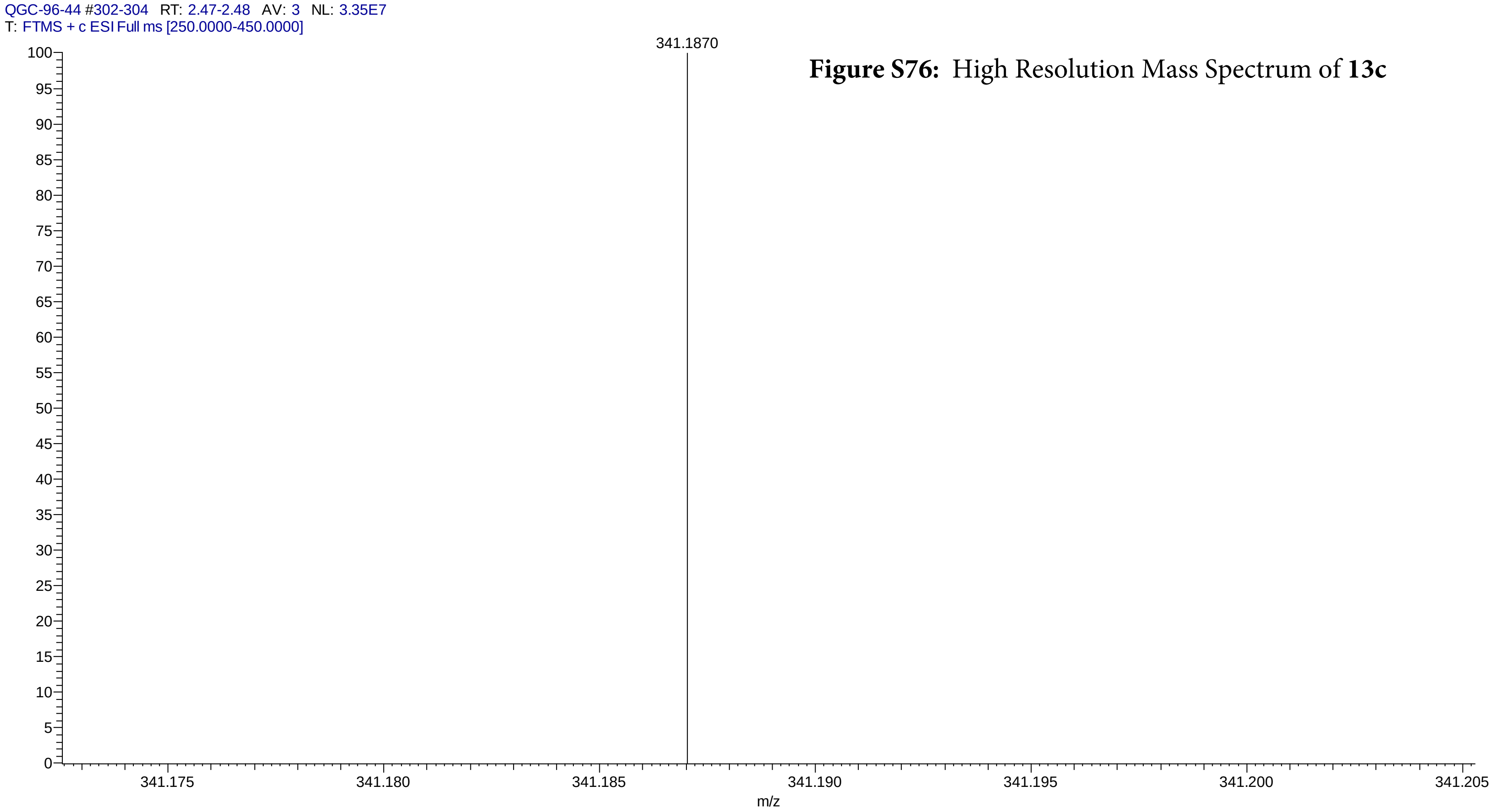

Figure S76: High Resolution Mass Spectrum of **13c**

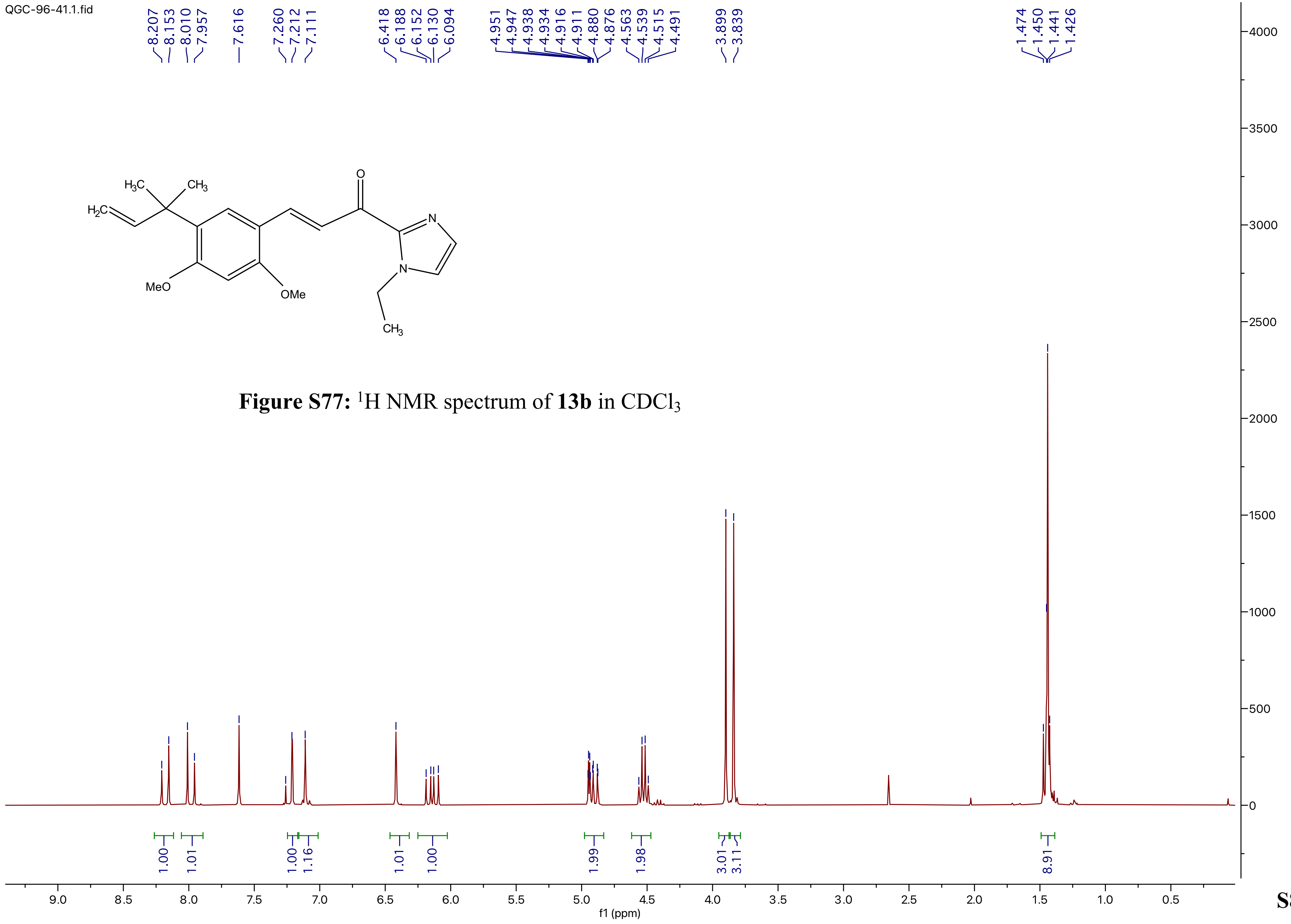

Figure S77: ^{1}H NMR spectrum of **13b** in $CDCl_3$

QGC-96-41

Figure S78: ^{13}C NMR spectrum of **13b** in $CDCl_3$

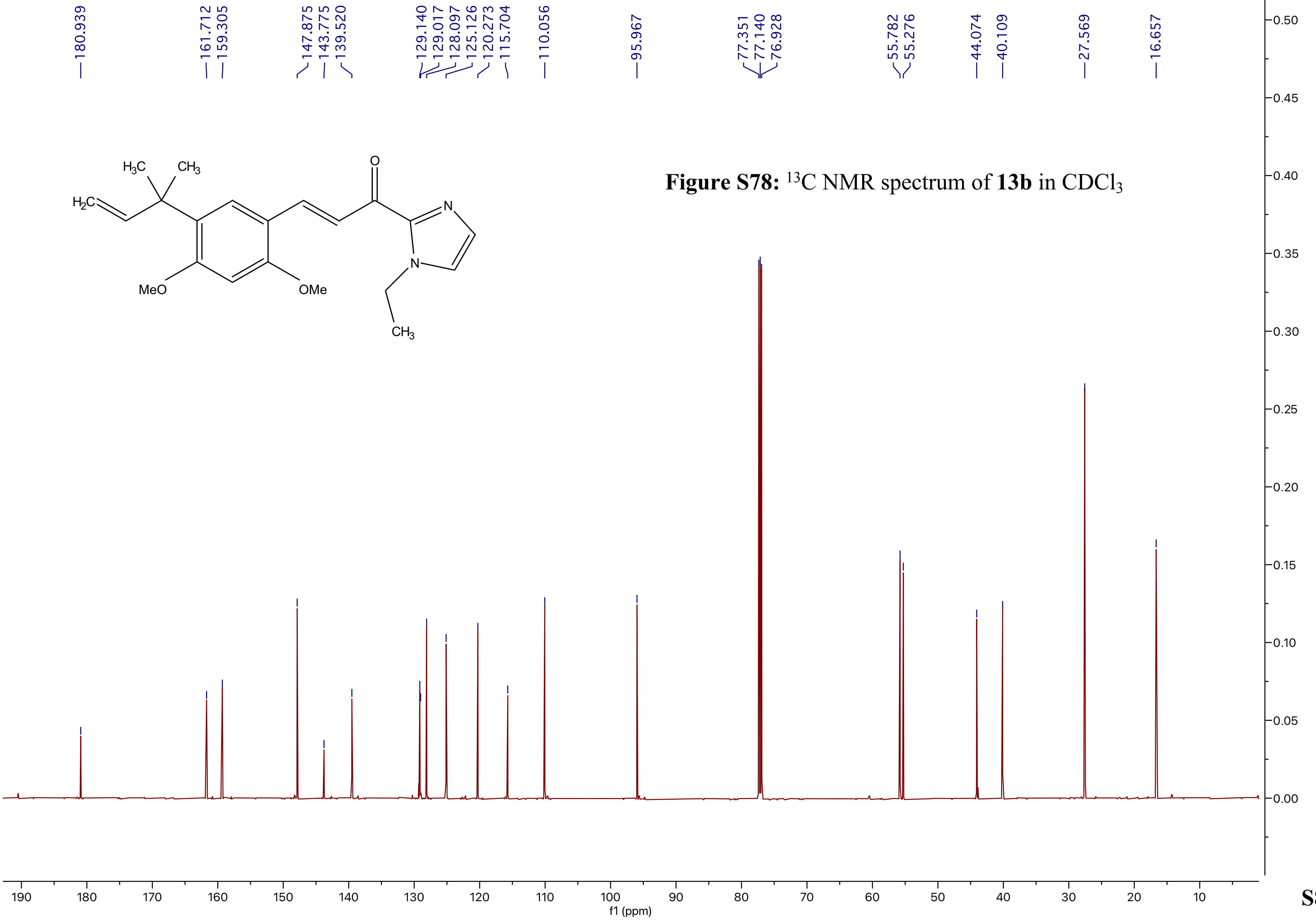

Smple Name	Mol Fomla	MW	M+H	obsved	dlta	ppm
QGC-96-41	C21H26N2O3	354.1943	355.2021	355.2027	0.0006	1.69

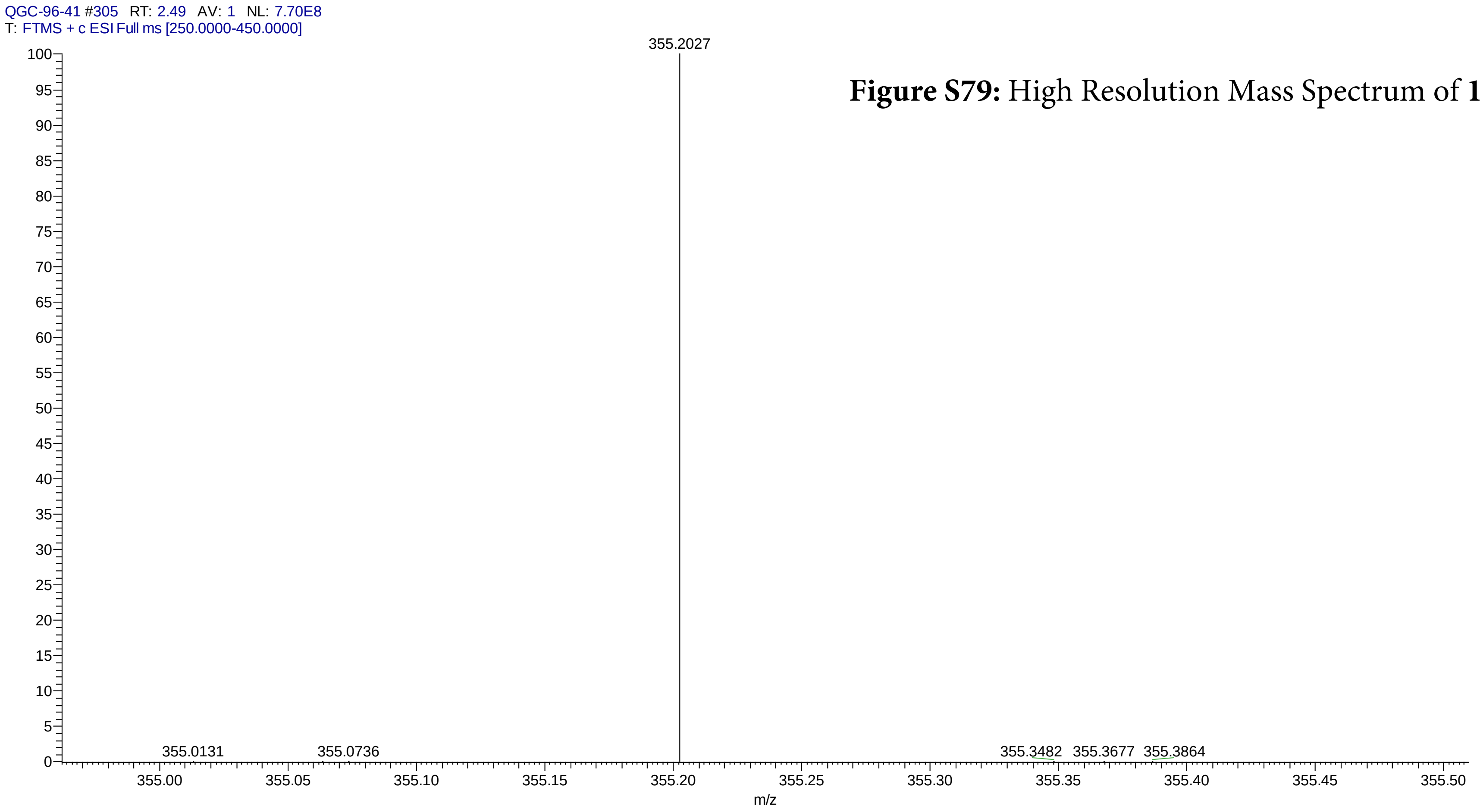

Figure S79: High Resolution Mass Spectrum of **13b**

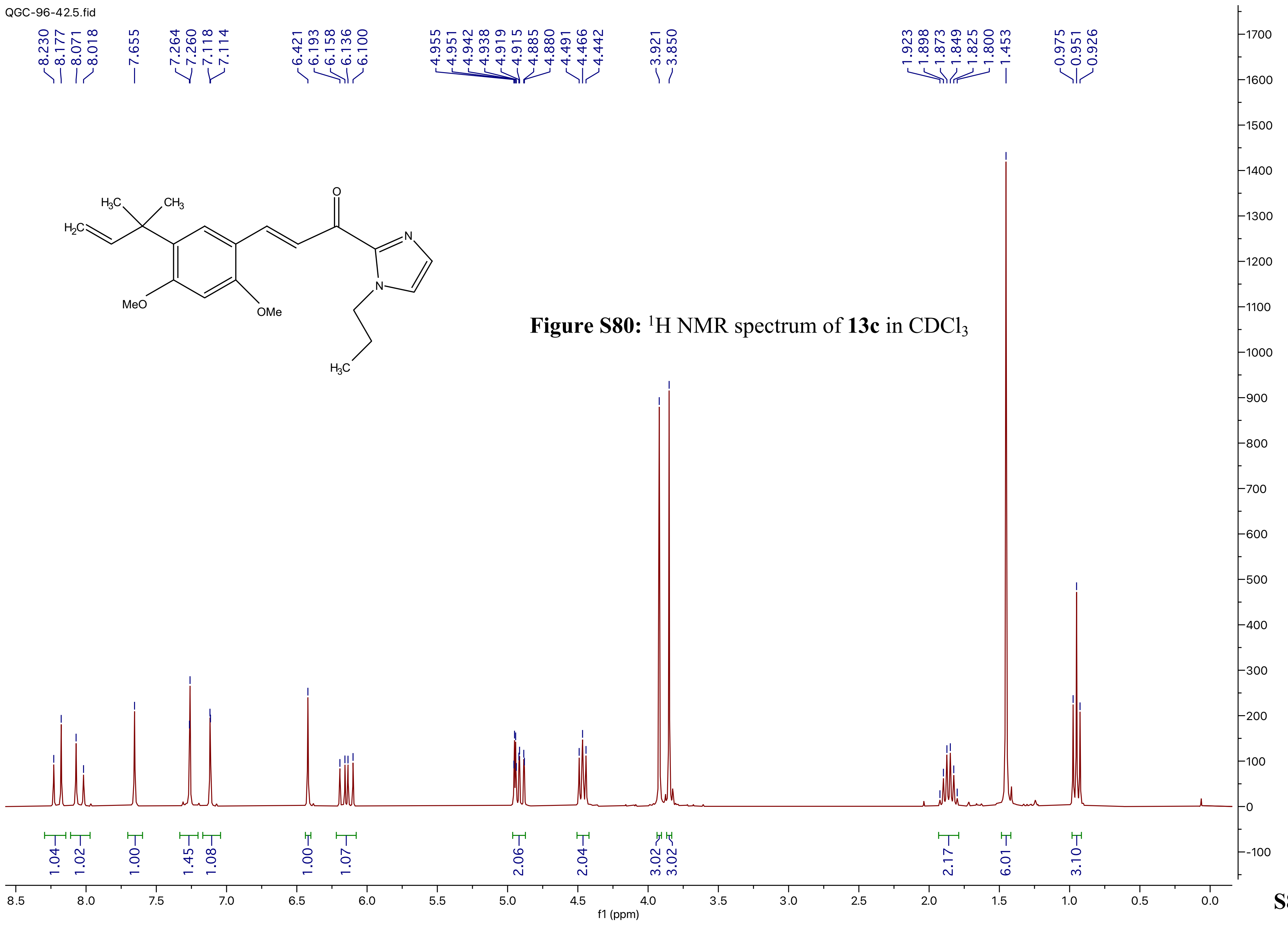

Figure S80: ^{1}H NMR spectrum of **13c** in $CDCl_3$

QGC-96-42

Figure S81: ^{13}C NMR sepctrum of **13c** in $CDCl_3$

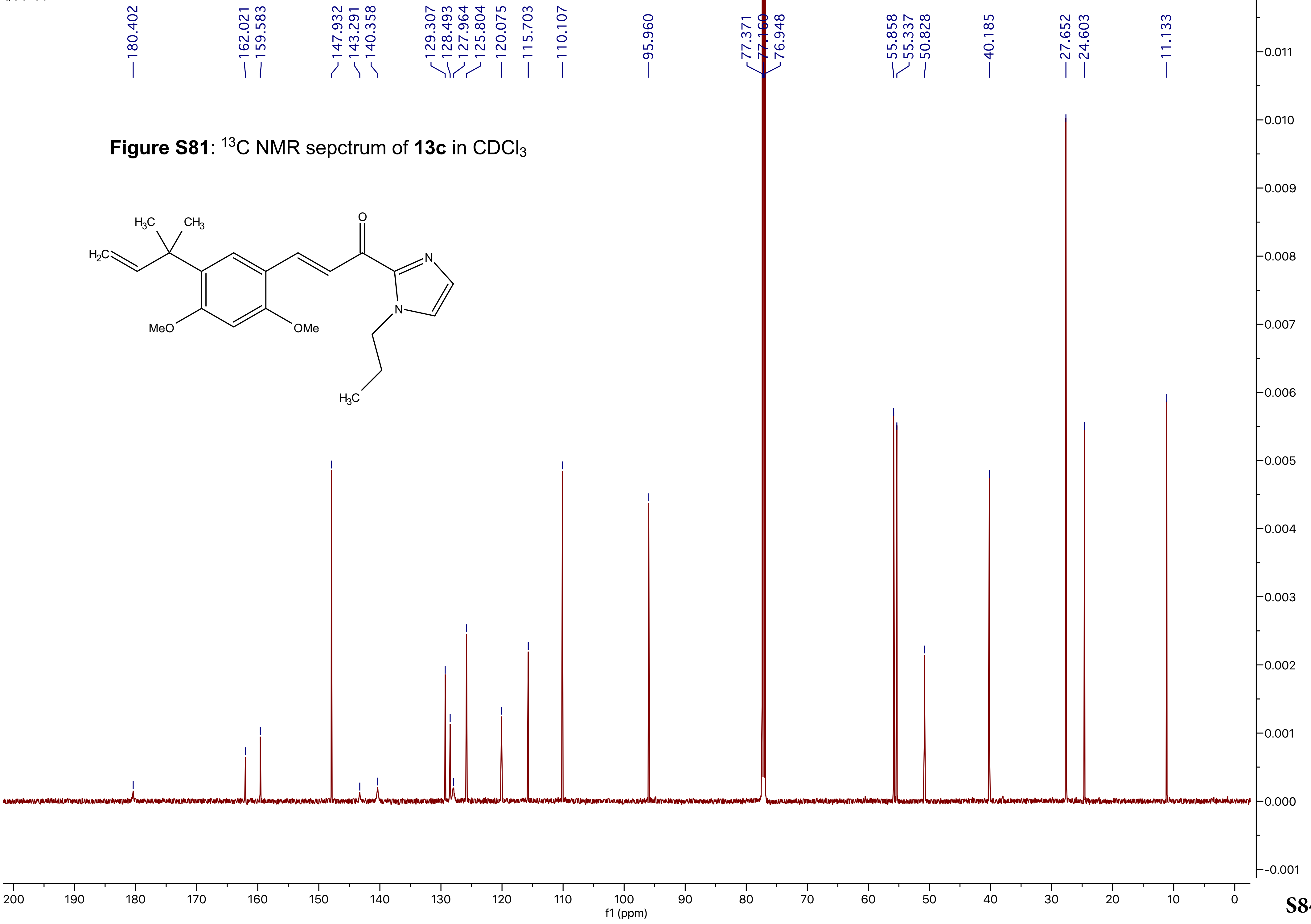

Smple Name	Mol Fomla	MW	M+H	obsved	dlta	ppm
QGC-96-42	C22H28N2O3	368.2100	369.2178	369.2184	0.0006	1.63

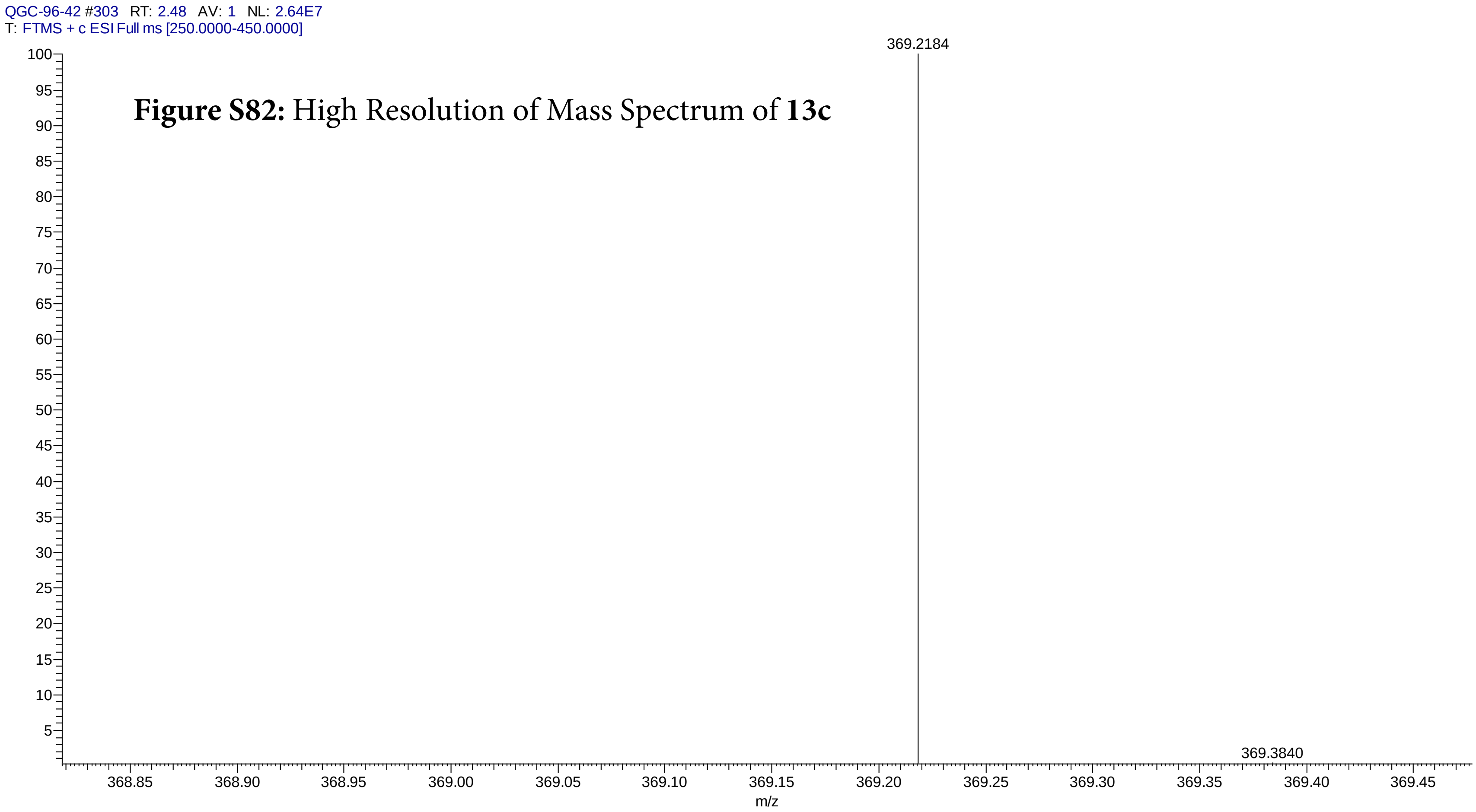

Figure S82: High Resolution of Mass Spectrum of **13c**

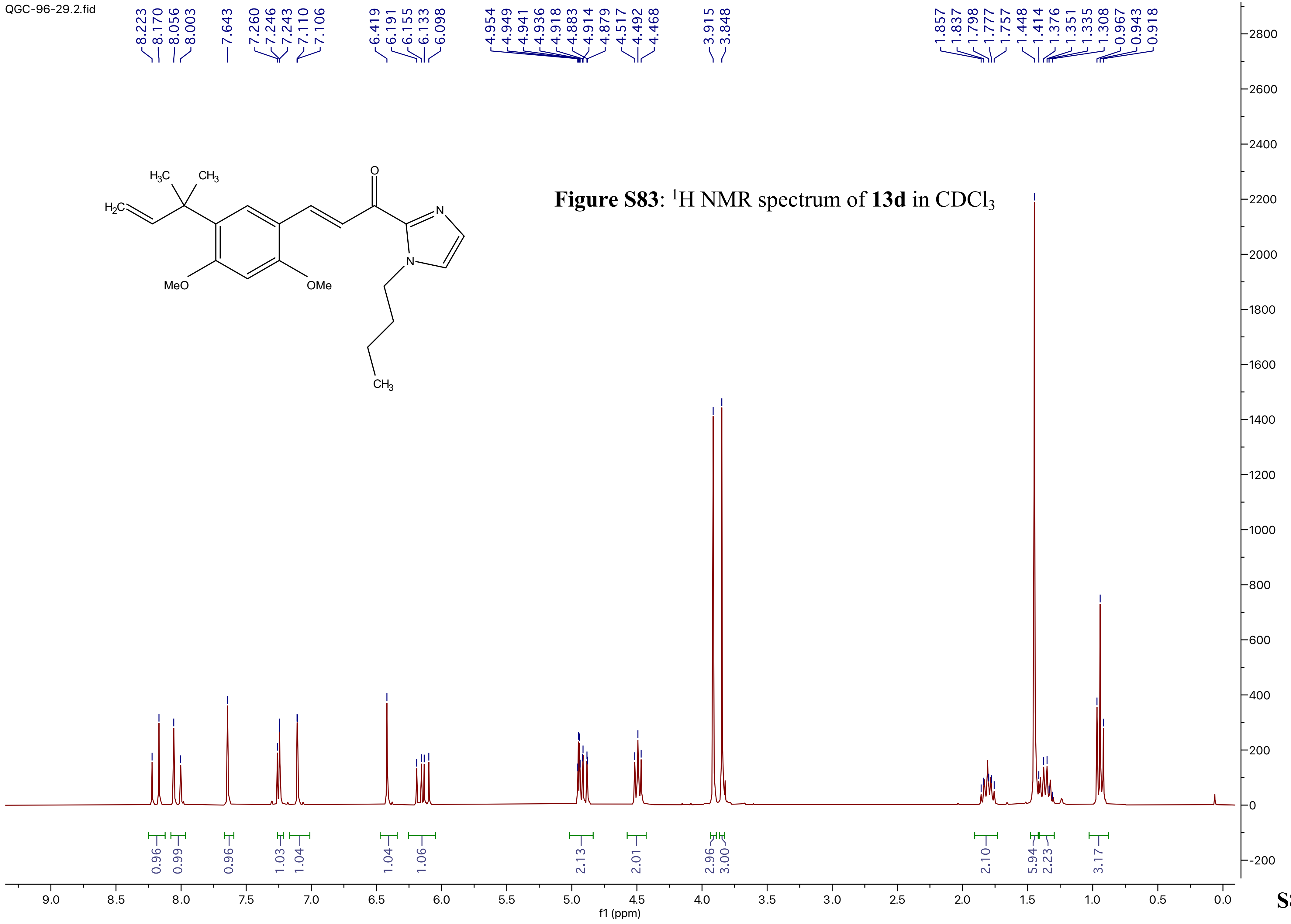

Figure S83: ^{1}H NMR spectrum of **13d** in $CDCl_3$

QGC-96-29.3.fid

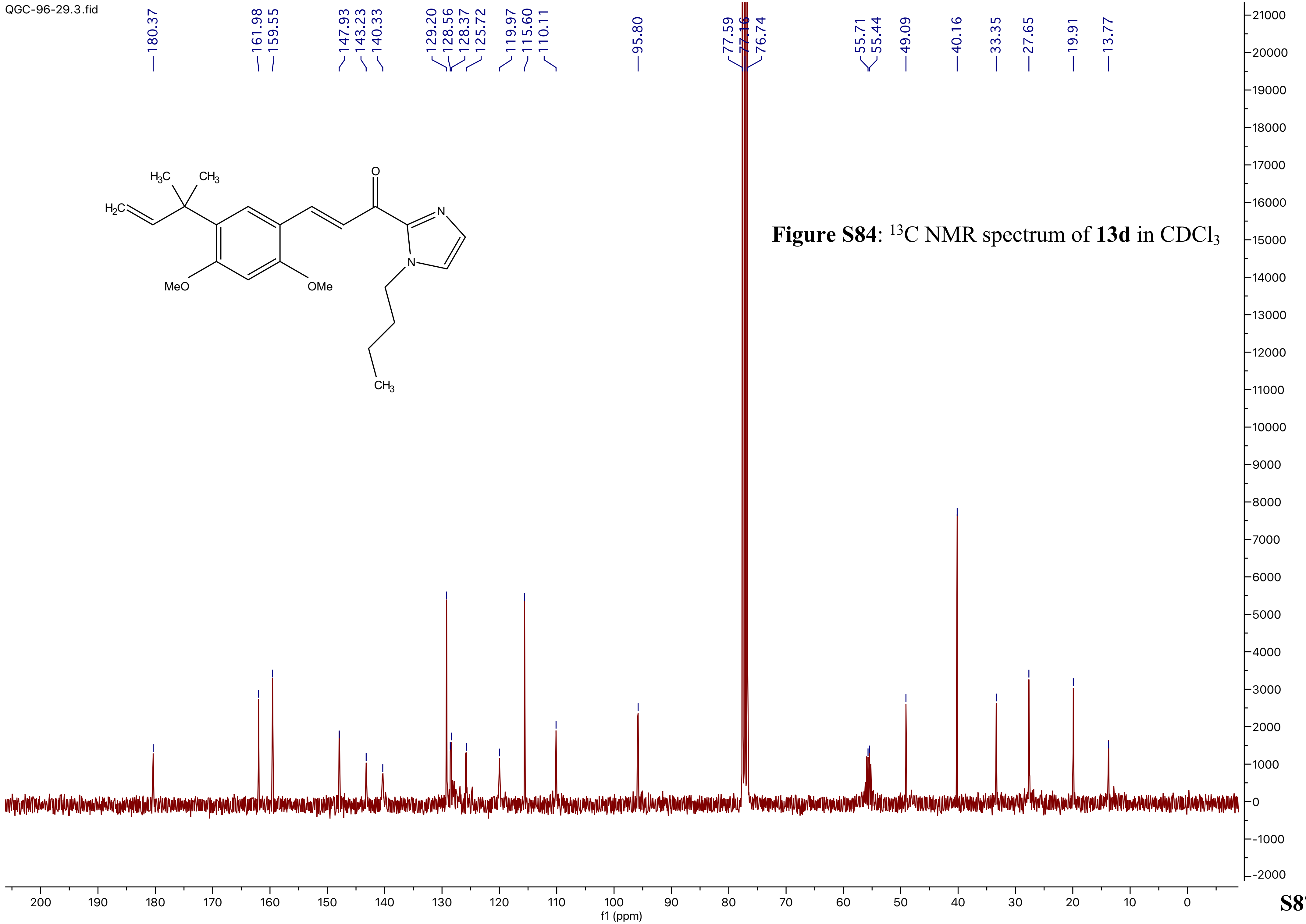

Figure S84: ^{13}C NMR spectrum of **13d** in $CDCl_3$

Smple Name	Mol Fomla	MW	M+H	obsved	dlta	ppm
QGC-96-29	C23H30N2O3	382.2256	383.2334	383.2334	0.0000	0.00

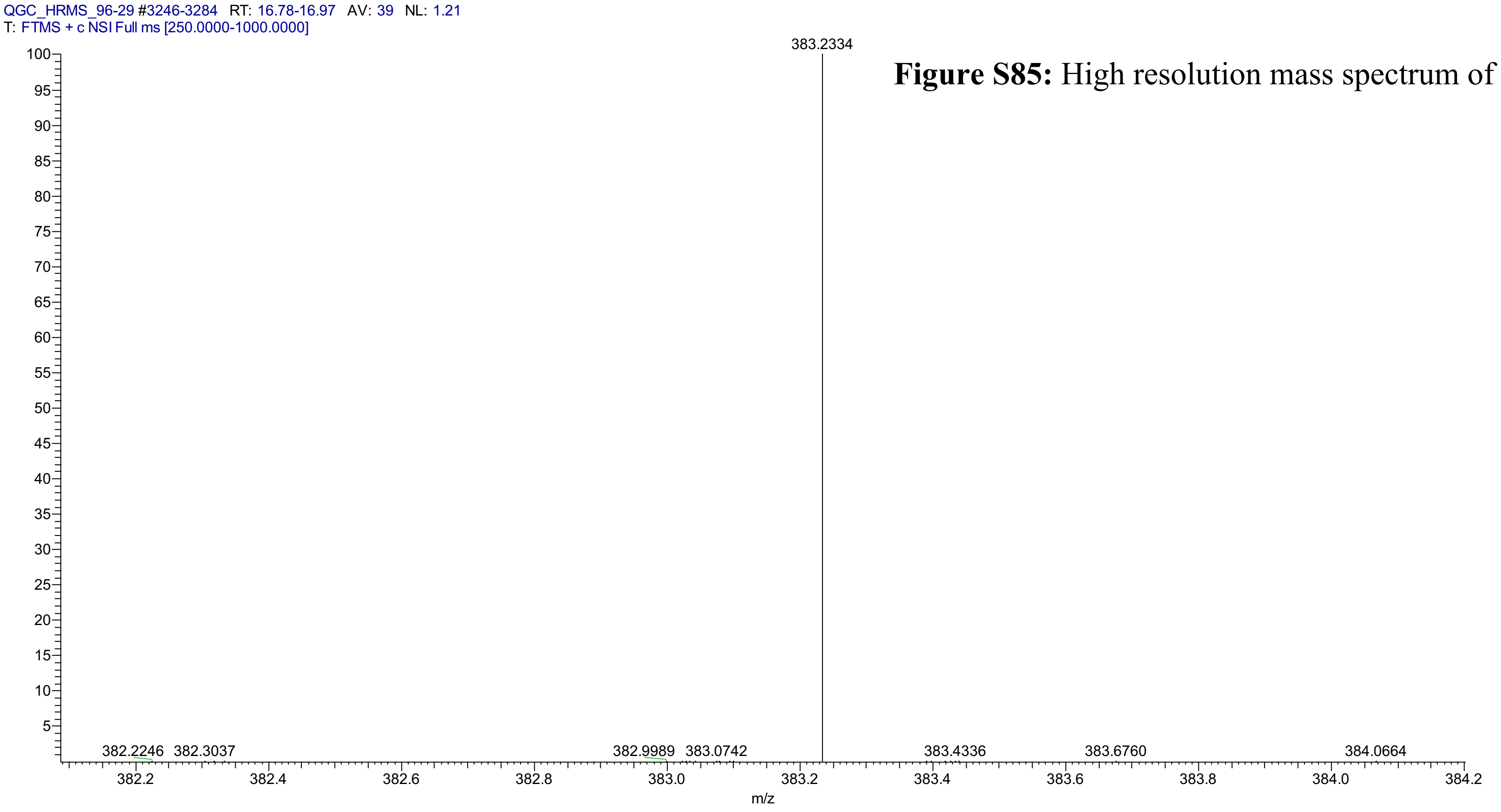

Figure S85: High resolution mass spectrum of **13d**

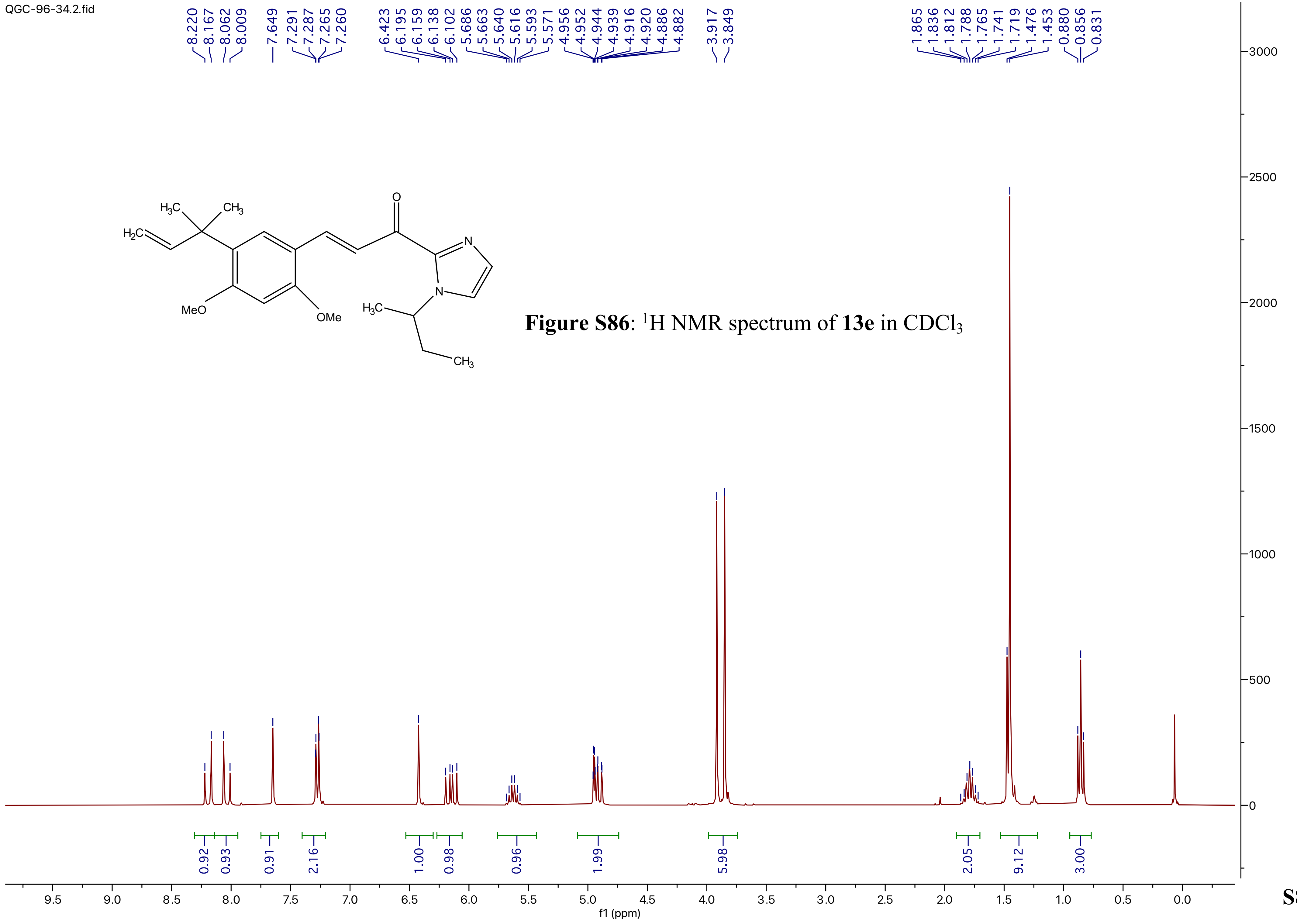

Figure S86: ^{1}H NMR spectrum of **13e** in $CDCl_3$

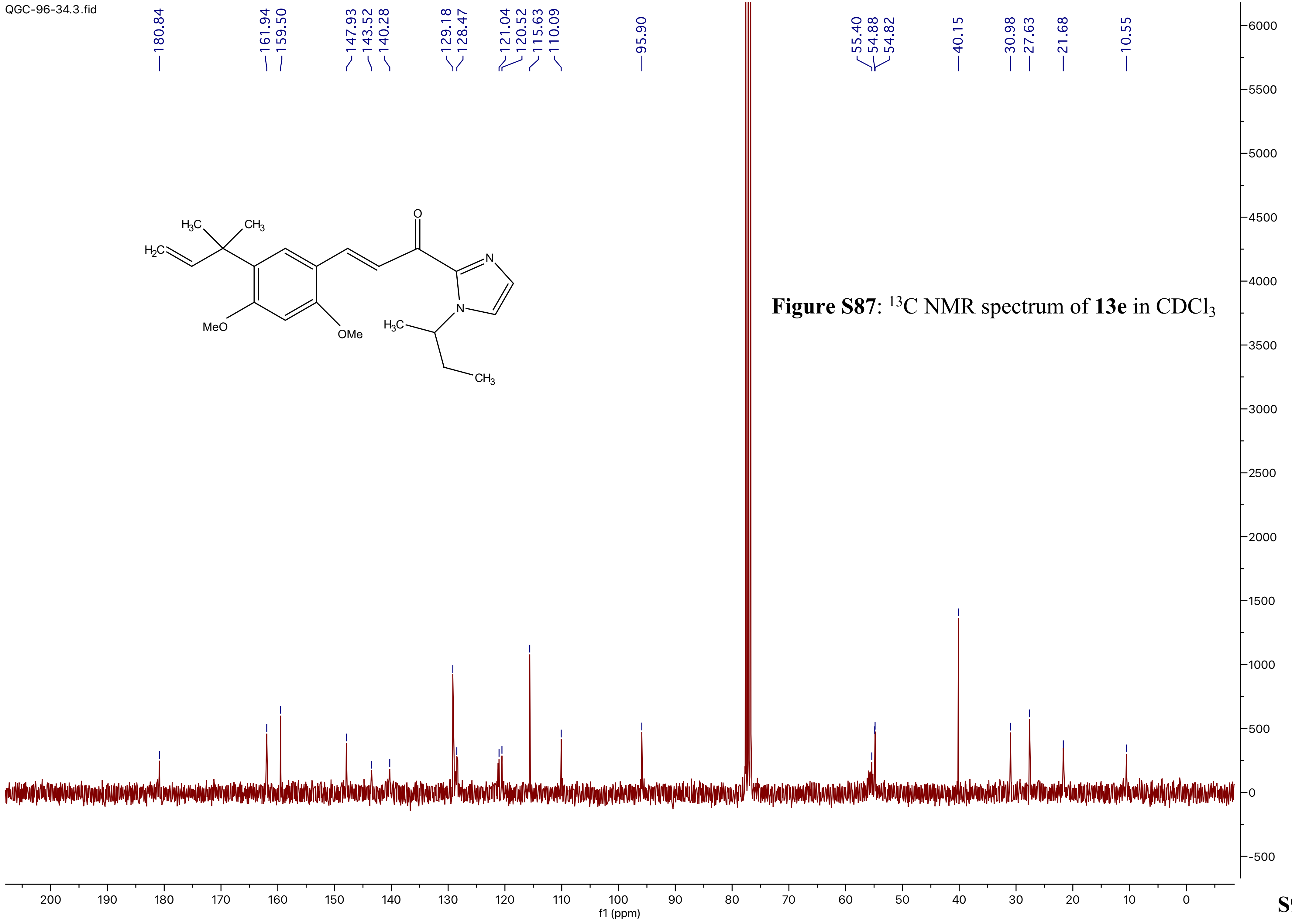

Figure S87: ^{13}C NMR spectrum of **13e** in $CDCl_3$

Smple Name	Mol Fomla	MW	M+H	obsved	dlta	ppm
QGC-96-34	C23H30N2O3	382.2256	383.2334	383.2340	0.0006	1.57

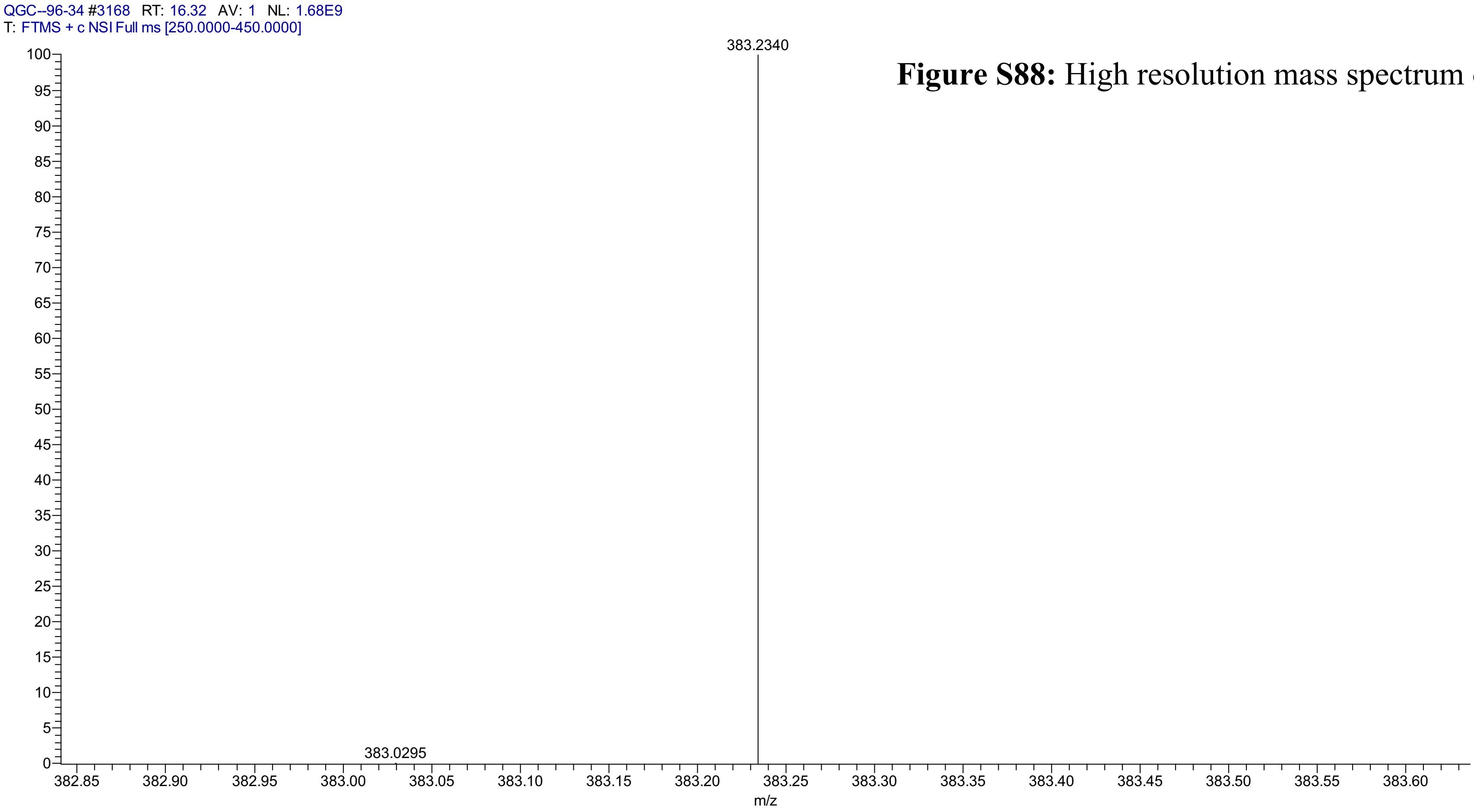

Figure S88: High resolution mass spectrum of **13e**

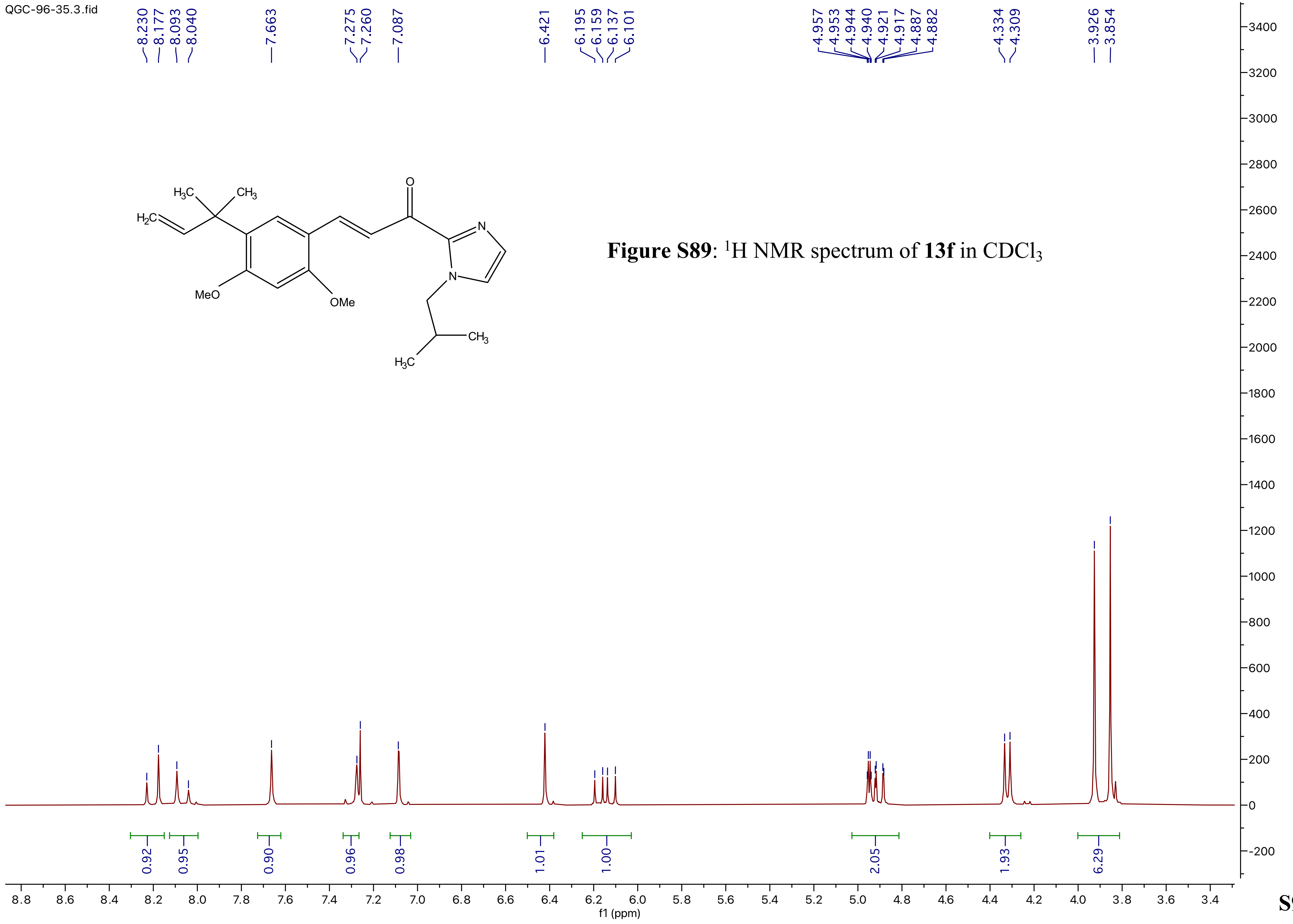

Figure S89: ^{1}H NMR spectrum of **13f** in $CDCl_3$

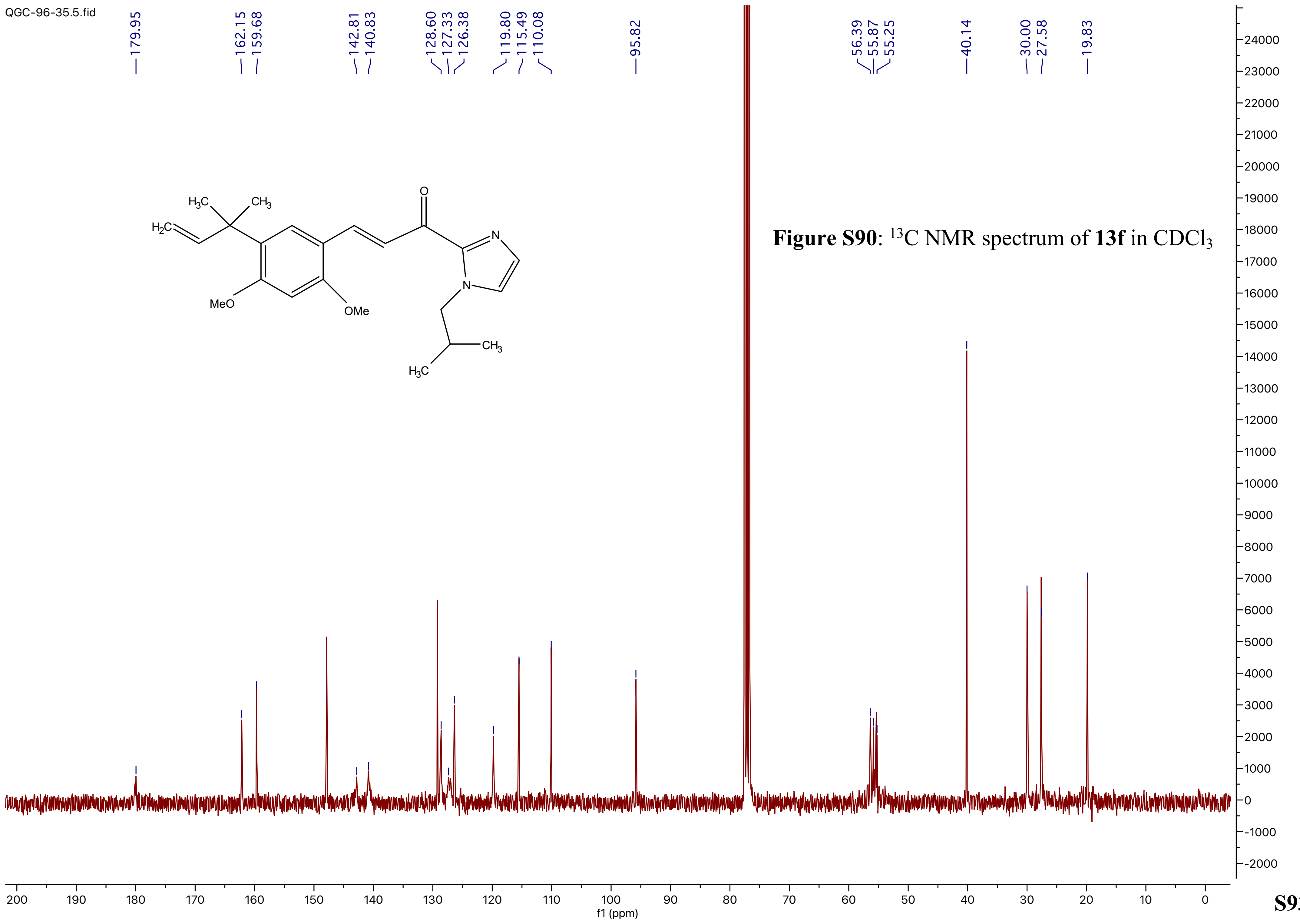

Figure S90: ^{13}C NMR spectrum of **13f** in $CDCl_3$

Smple Name	Mol Fomla	MW	M+H	obsved	dlta	ppm
QGC-96-35	C23H30N2O3	382.2256	383.2334	383.2341	0.0007	1.83

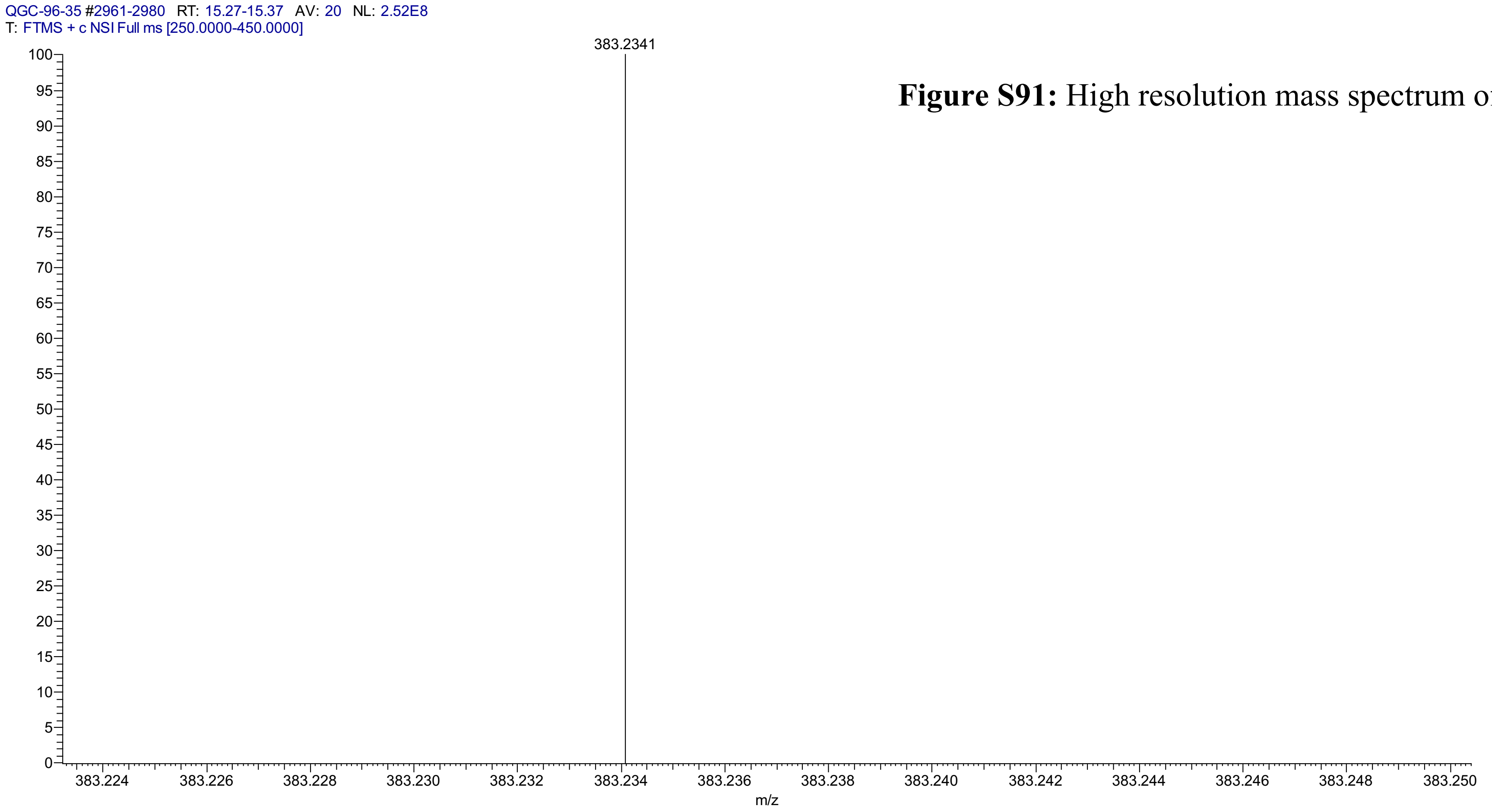

Figure S91: High resolution mass spectrum of **13f**

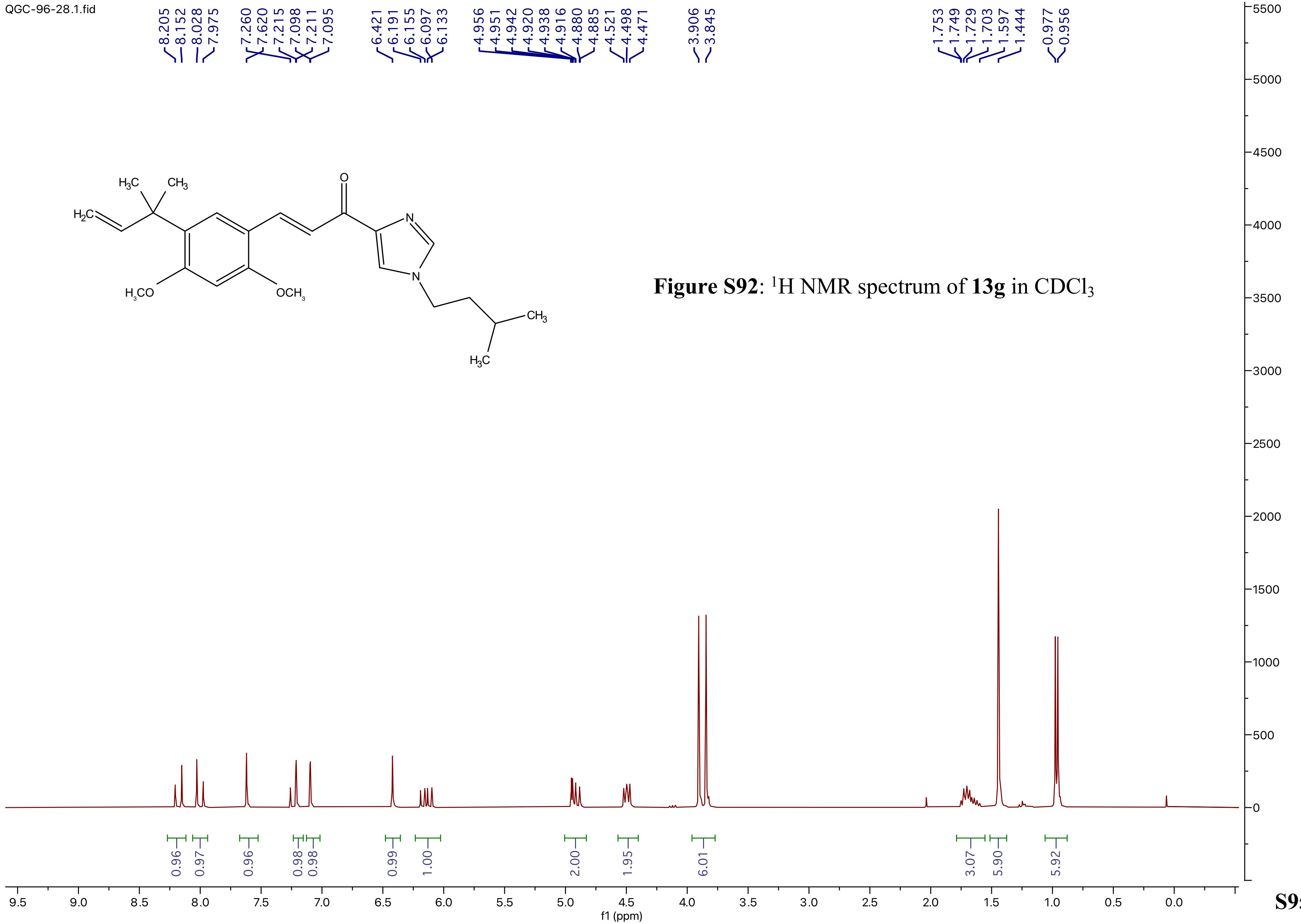

Figure S92: ^{1}H NMR spectrum of **13g** in $CDCl_3$

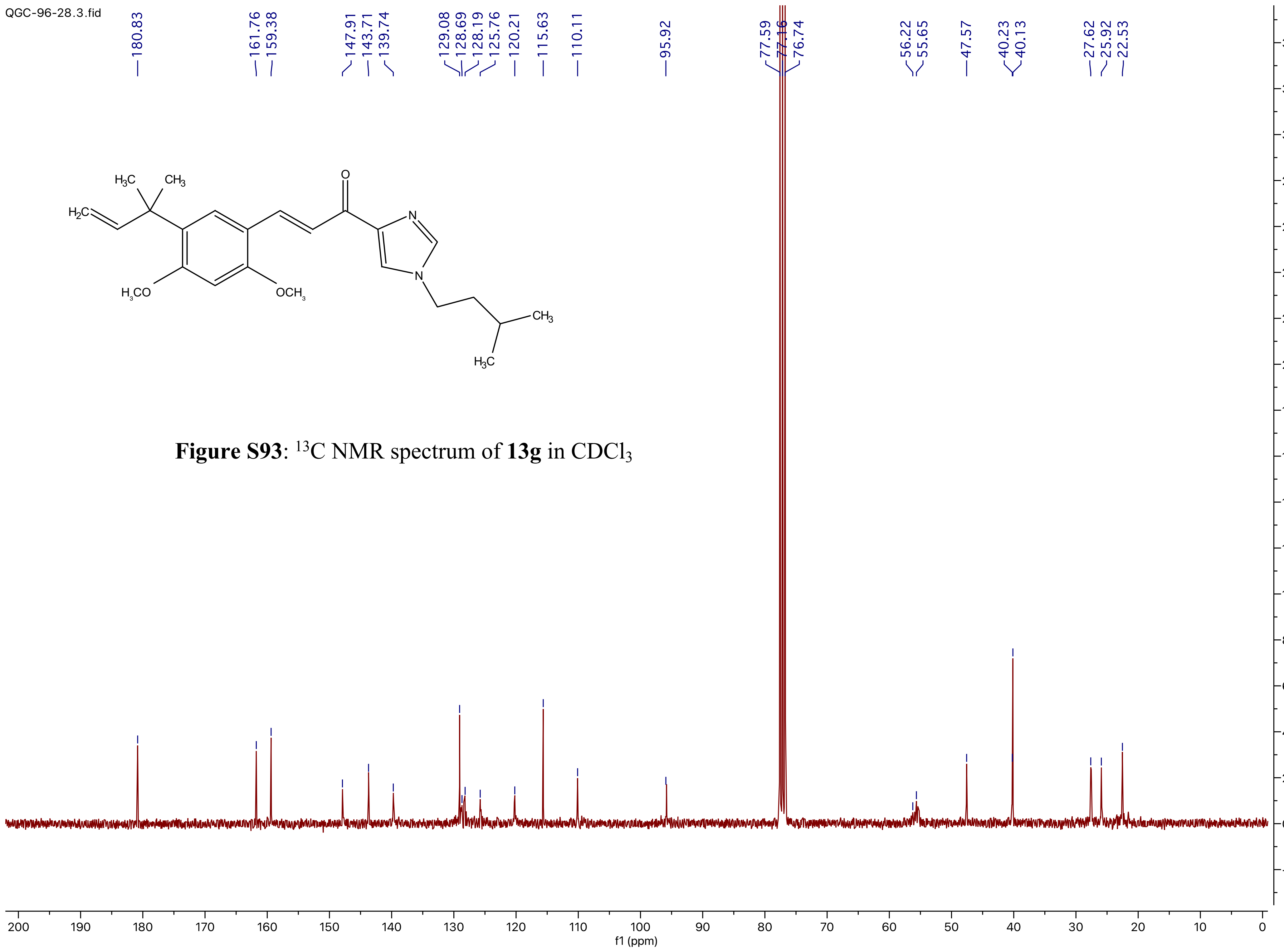

Figure S93: ^{13}C NMR spectrum of **13g** in $CDCl_3$

Smple Name	Mol Fomla	MW	M+H	obsved	dlta	ppm
QGC-96-28	C24H32N2O3	396.2413	397.2491	397.2490	-0.0001	-0.25

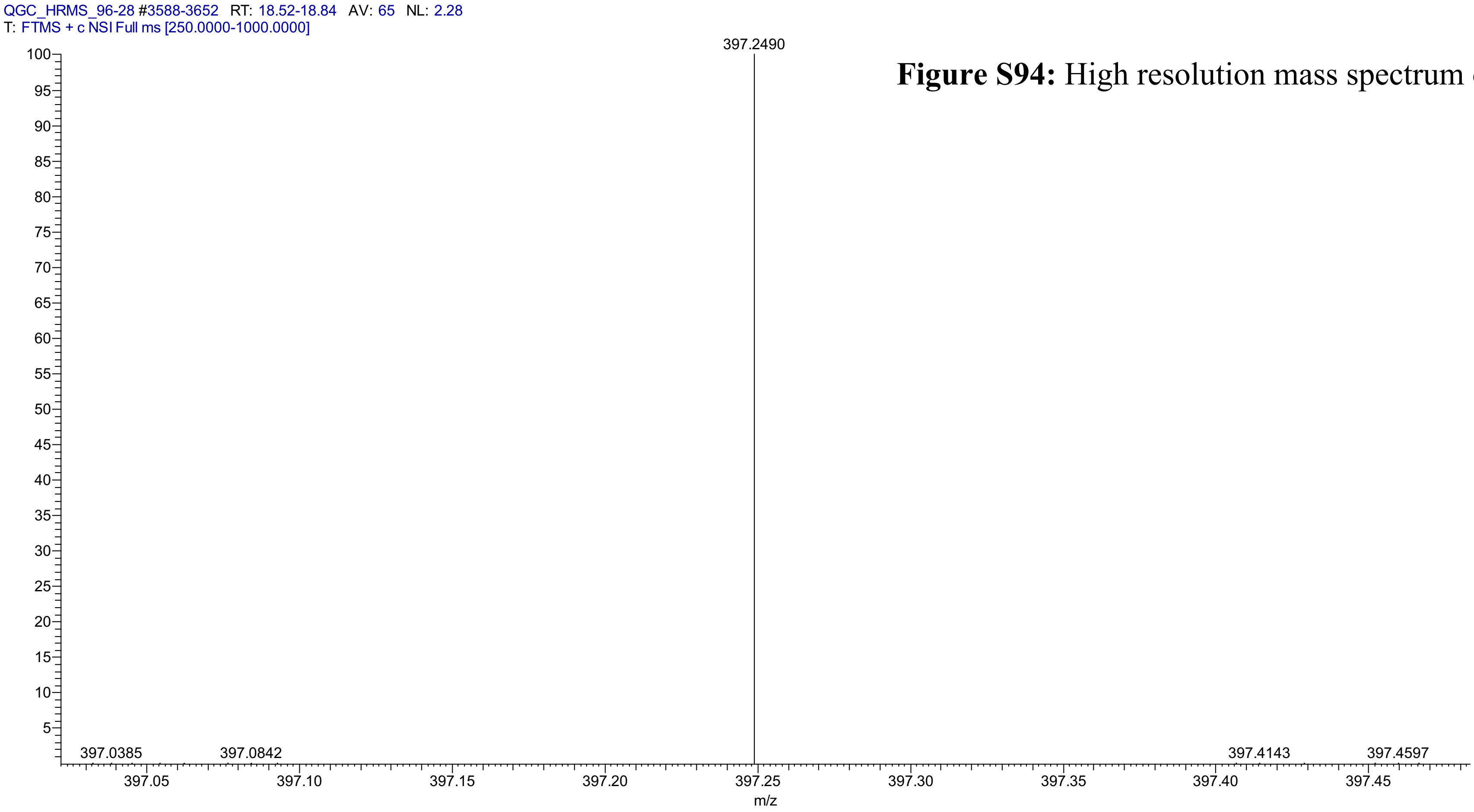

Figure S94: High resolution mass spectrum of **13g**

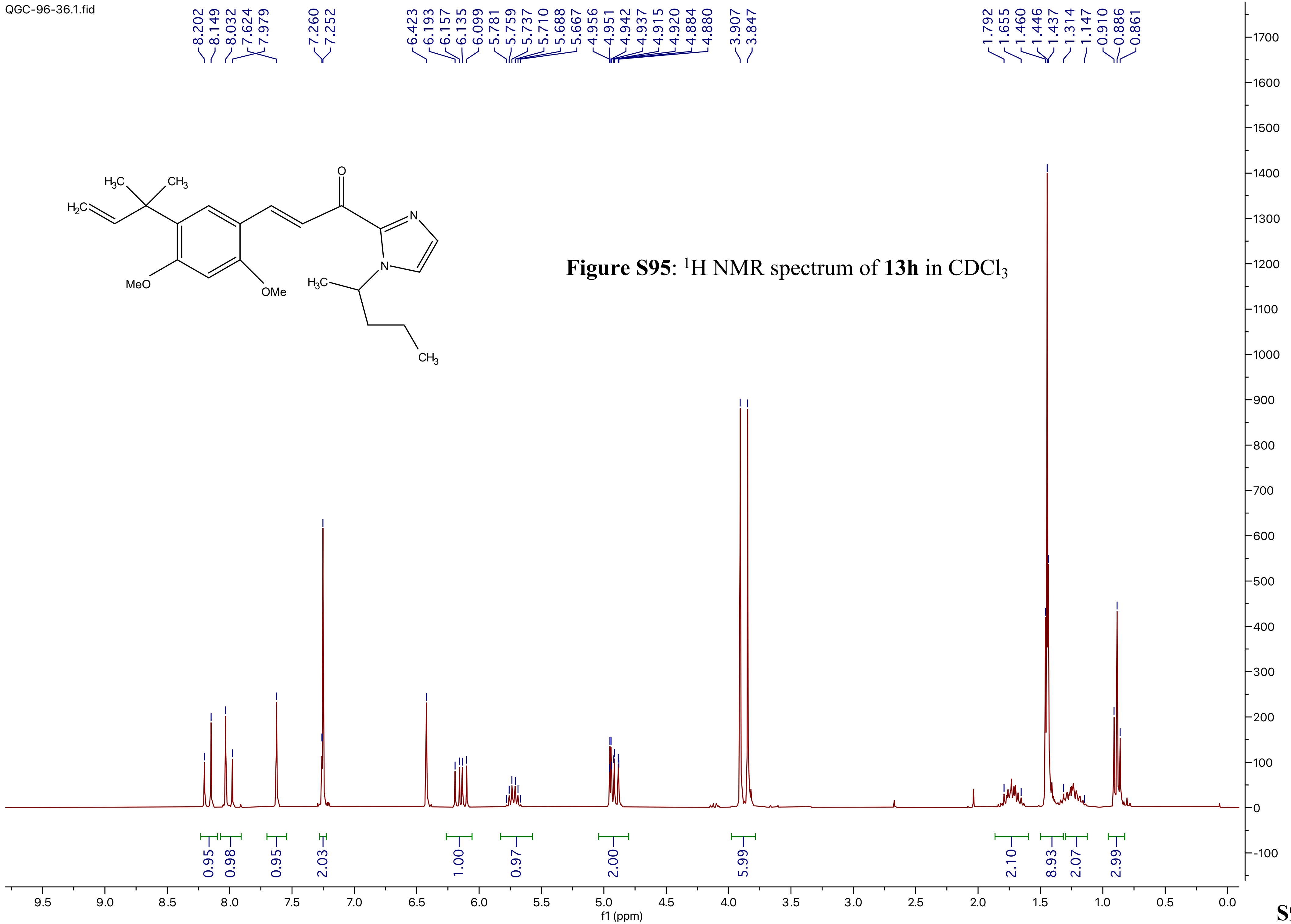

Figure S95: ^{1}H NMR spectrum of **13h** in $CDCl_3$

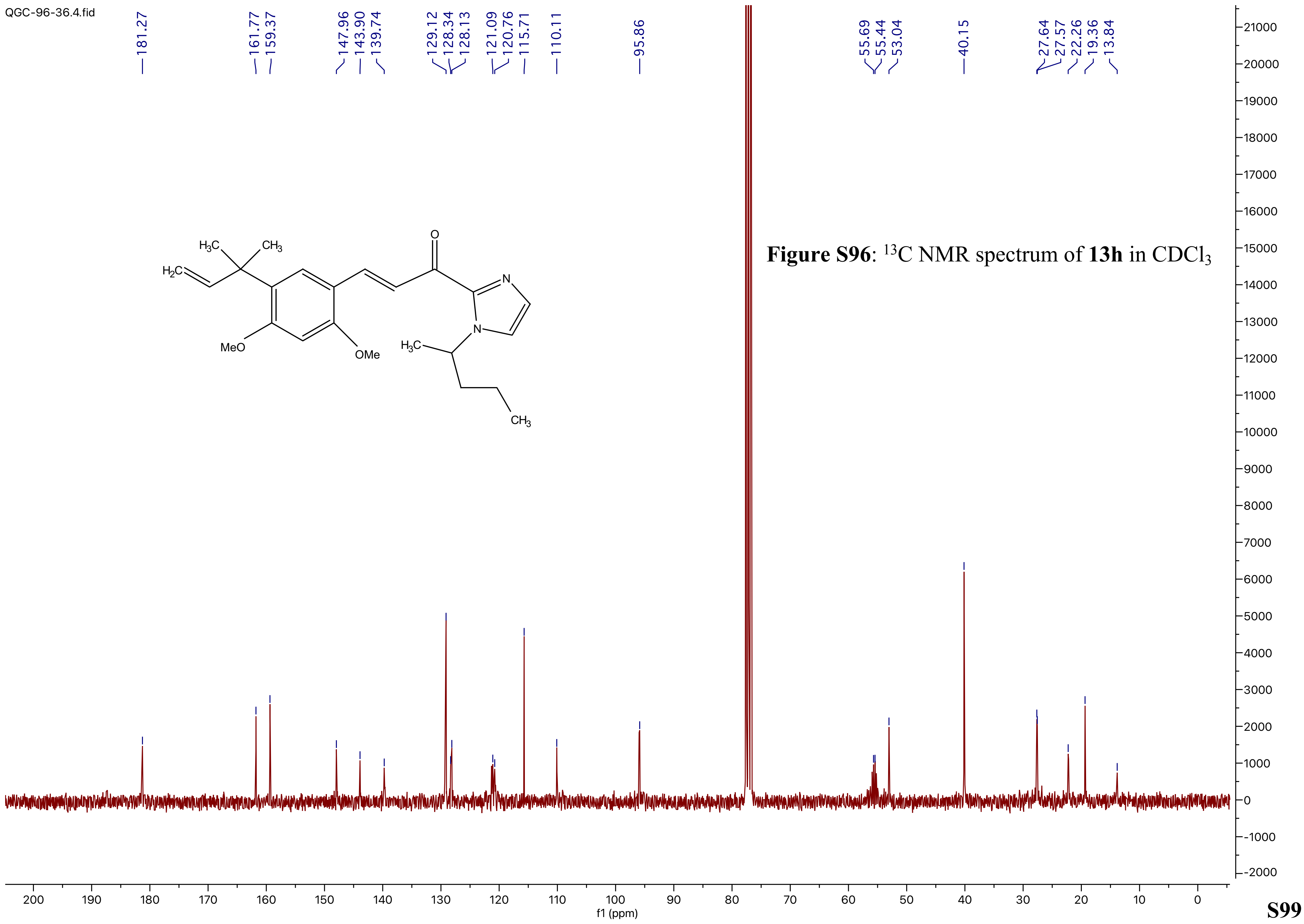

Figure S96: ^{13}C NMR spectrum of **13h** in $CDCl_3$

Smple Name	Mol Fomla	MW	M+H	obsved	dlta	ppm
QGC-96-36	C24H32N2O3	396.2413	397.2491	397.2496	0.0005	1.26

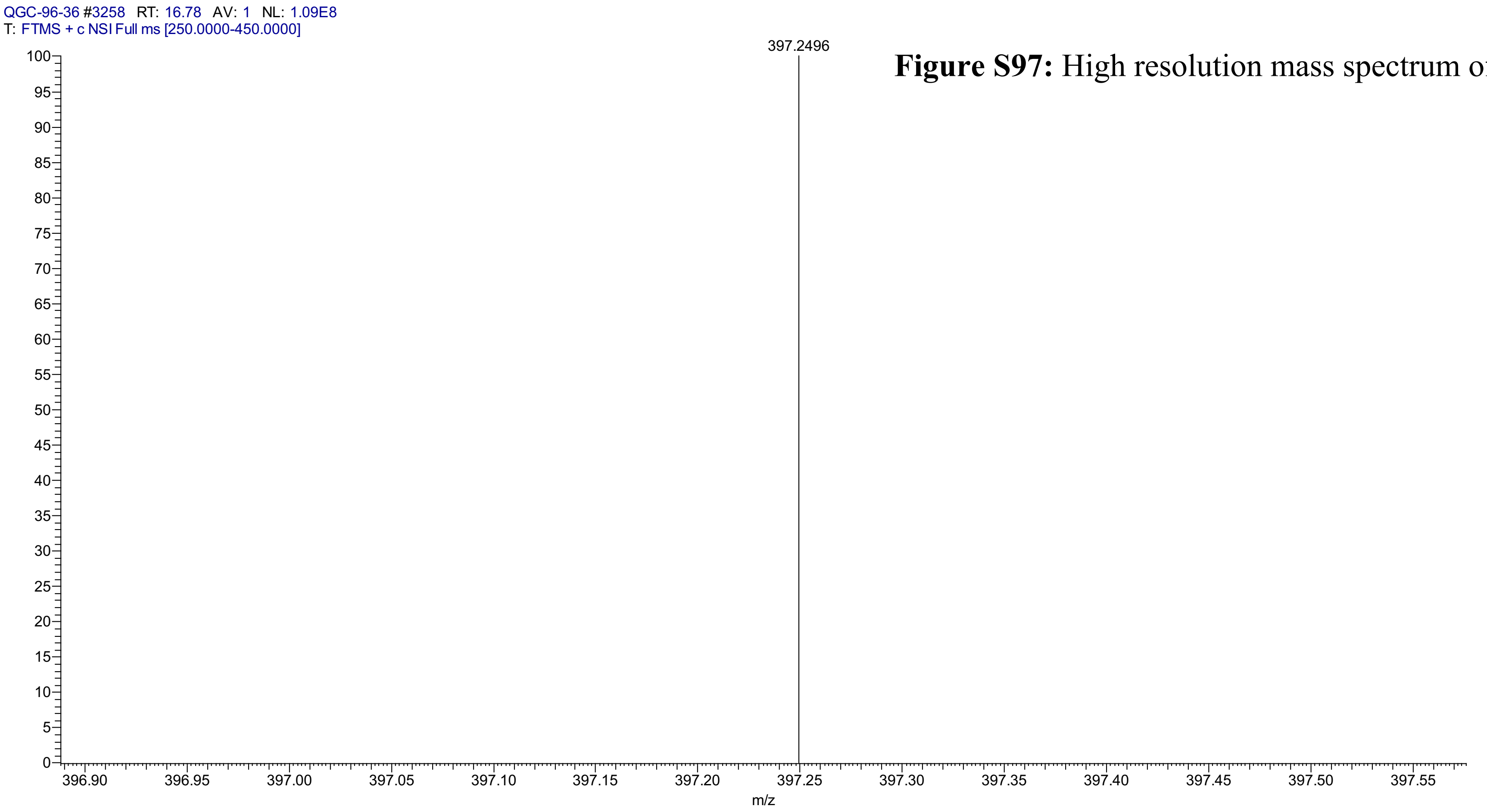

Figure S97: High resolution mass spectrum of **13h**

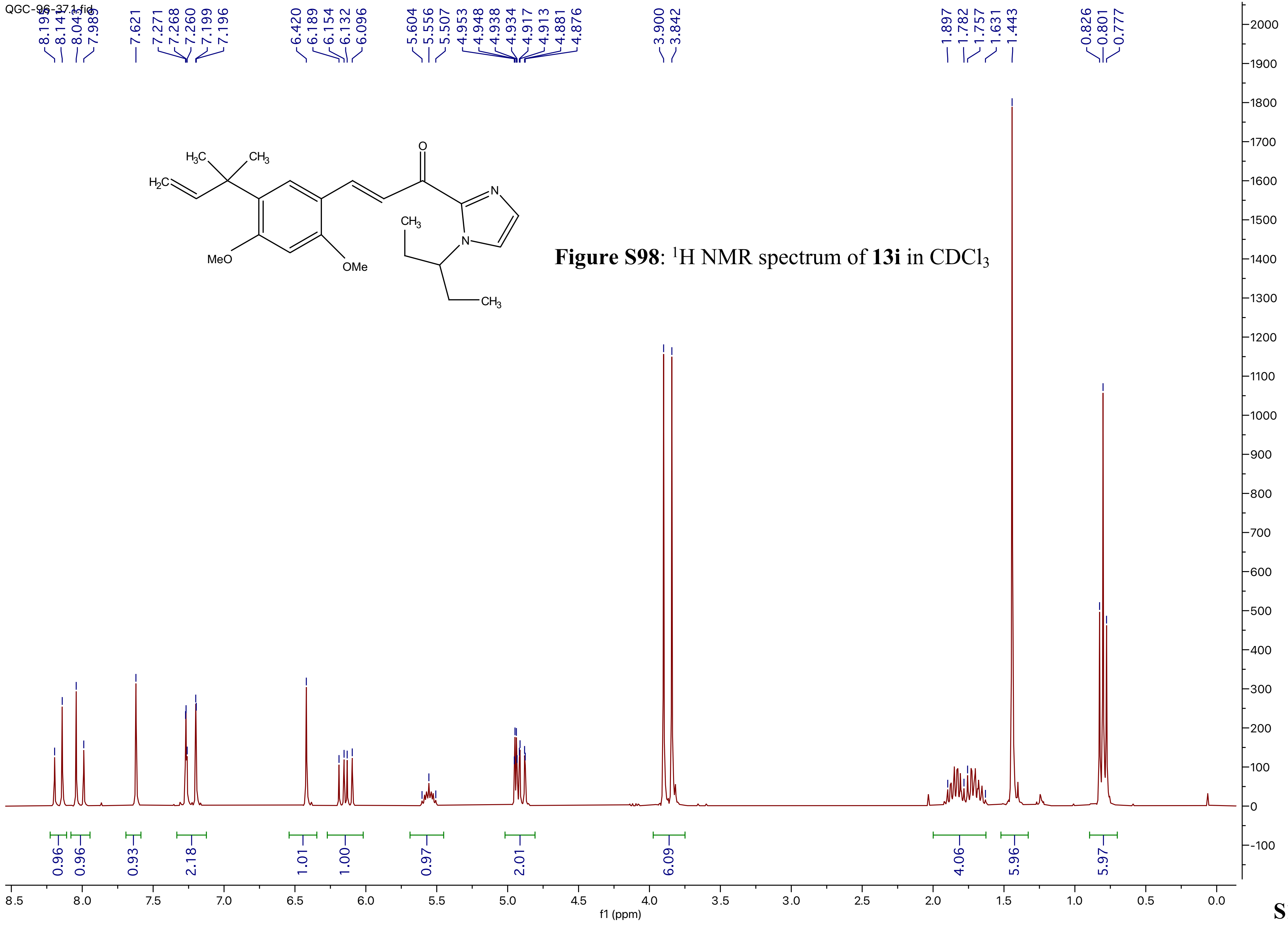

Figure S98: ^{1}H NMR spectrum of **13i** in $CDCl_3$

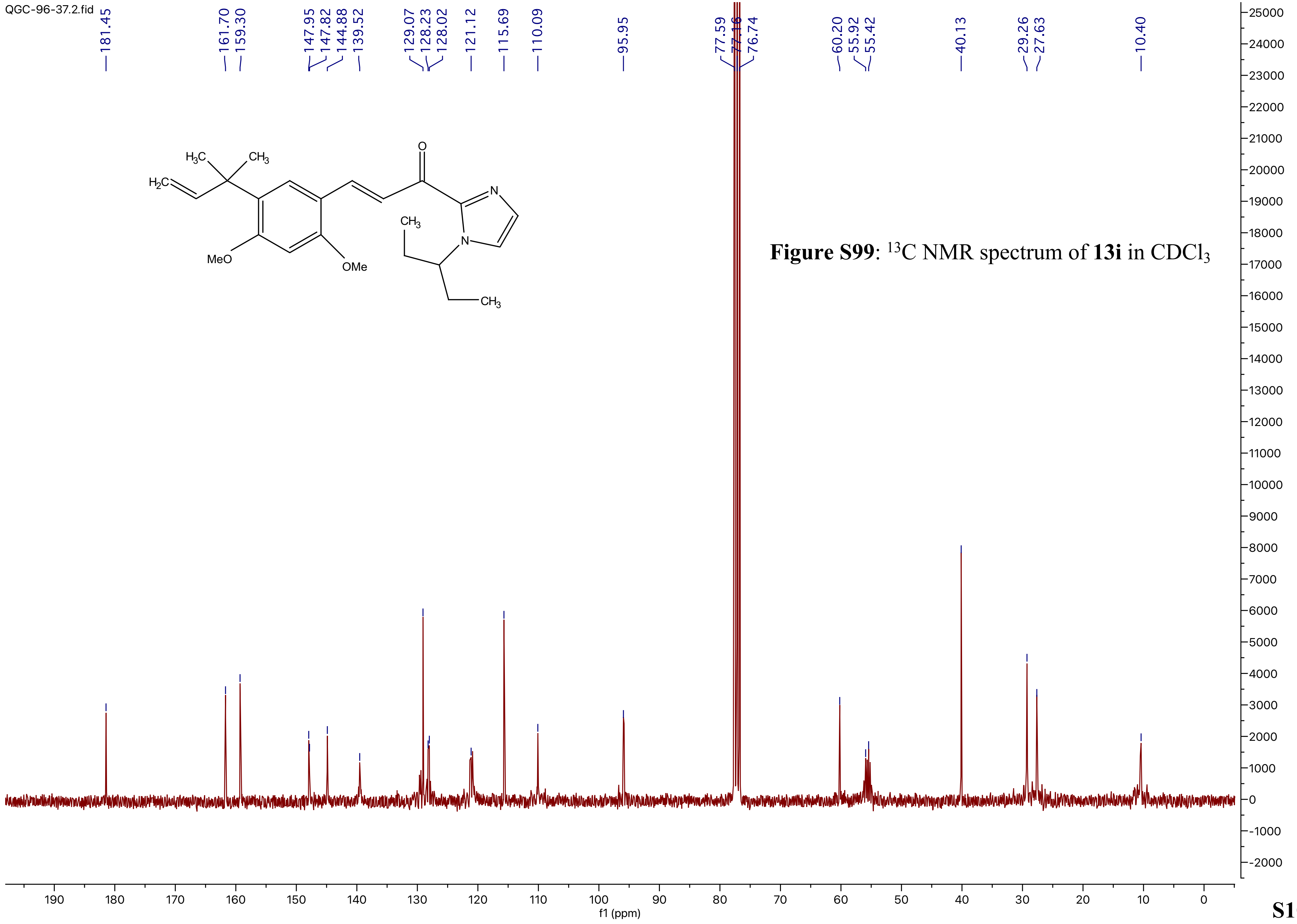

Figure S99: ^{13}C NMR spectrum of **13i** in $CDCl_3$

Smple Name	Mol Fomla	MW	M+H	obsved	dlta	ppm
QGC-96-37	C24H32N2O3	396.2413	397.2491	397.2495	0.0004	1.01

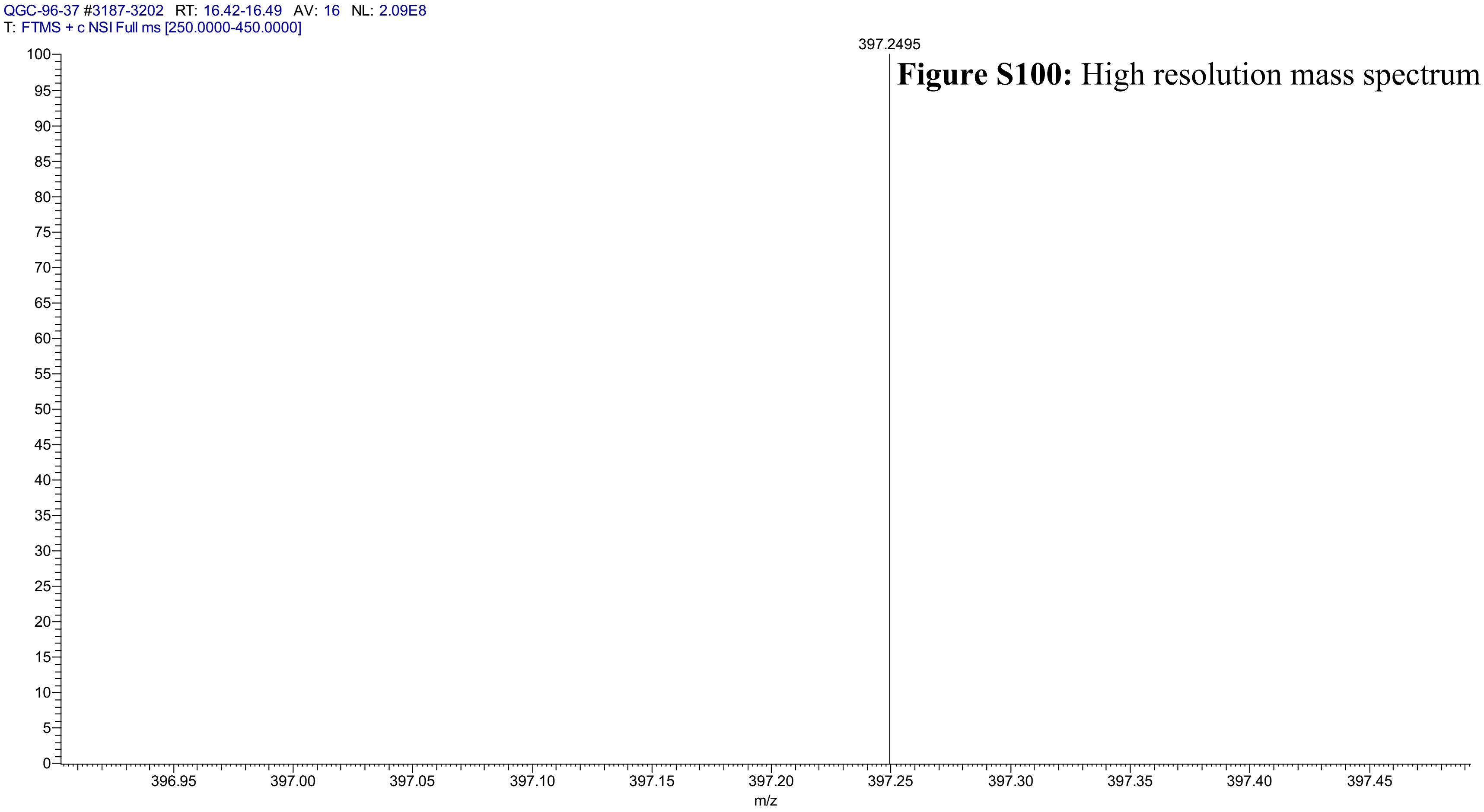

Figure S100: High resolution mass spectrum of **13i**
